# Synthesis, Reactivity and Antimicrobial Activity of a Series of 2-Arylamino-1,3-selenazoles

**DOI:** 10.3390/molecules26247695

**Published:** 2021-12-20

**Authors:** Julia Kuchar, Katharina Reinhold, Vera Rösgen, Nils Nöthling, Christian W. Lehmann, Fabian Mohr

**Affiliations:** 1 Anorganische Chemie, Fakultät für Mathematik und Naturwissenschaften, Bergische Universität Wuppertal, Gaußstr. 20, 42119 Wuppertal, Germany; kuchar@uni-wuppertal.de (J.K.); katharina.reinhold@uni-wuppertal.de (K.R.); vera.roesgen@uni-wuppertal.de (V.R.); 2 Max-Planck-Institut für Kohlenforschung, Kaiser-Wilhelm-Platz 1, 45470 Mülheim an der Ruhr, Germany; noethling@kofo.mpg.de (N.N.); lehmann@mpi-muelheim.mpg.de (C.W.L.)

**Keywords:** 1,3-selenazole, metallation reactions, X-ray structures, antimicrobial activity

## Abstract

A series of 2-arylamino-1,3-selenazoles was synthesized and their reactivity was studied. The 2-arylamino-1,3-selenazoles and their reaction products were characterized by various spectroscopic methods and X-ray diffraction. In addition, the antimicrobial activity of the 2-arylamino-1,3-selenazoles in a panel of seven bacteria and fungi was examined.

## 1. Introduction

**Scheme 1:**
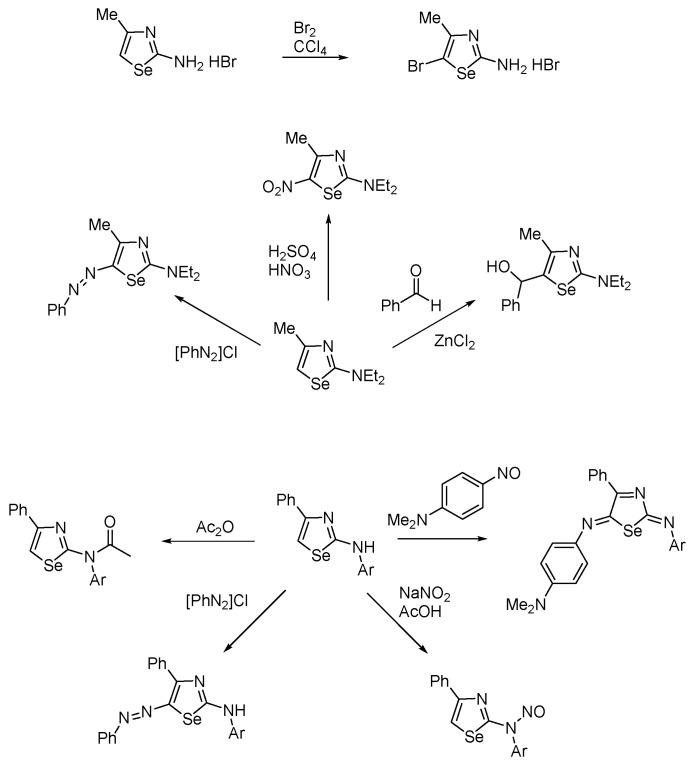
Known reactions of various 2-amino-1,3-selenazoles.

2-amino-1,3-selenazoles are five-membered, selenium-containing heterocycles, which were known for a long time. It was Hofmann (a student of Hantzsch) who first showed in 1889 that the condensation of selenourea or phenylselenourea with chloroacetone or 2-bromoacetophenone afforded the corresponding 2-amino-1,3-selenazoles [1]. The compounds were isolated as their hydrochloride or hydrobromide salts and were also converted to the corresponding *N*-acetyl derivatives and their tetrachloroplatinate salts for characterization. More than 50 years later, Backer prepared a series of alkyl-substituted 2-amino-1,3-selenazoles, which were characterized as the hydrohalide salts, *N*-acetyl-derivates or picrate salts [2]. In the 1950s, several groups began to study this class of heterocycles in a little more detail. Haginiwa published two short papers (in Japanese) on the preparation and reactions of 2-amino-1,3-selenazoles, demonstrating the high reactivity at the C5-position of the heterocycle [3,4]. Bromination of the hydrobromide salt at position 5 was accomplished using bromine in CCl_4_, nitration or diazo-coupling of the 2-diethylamino-derivative was carried out with H_2_SO_4_/HNO_3_ and phenyl diazonium chloride, respectively (Scheme 1). The 2-diethylamino-derivative also condenses with benzaldehyde in the presence of ZnCl_2_ to furnish the corresponding carbinol (Scheme 1). These papers, however, contain very few details on the reactions themselves and how the structures of the products were assigned. Around the same time, Zingaro reported various 2-amino-4-methyl-1,3-selenazoles, including the 2-phenylamino-derivative, which was reported to be an oil [5]. Metzger and Bailly showed that the reaction of 2-chloroaldehydes (2-chlorethanal and 2-chlorobutanal) with selenourea leads to 5-alkyl-substituted 2-amino-1,3-selenazoles [6]. As an alternative preparative method, King developed a one-pot reaction involving ketones, iodine and selenourea, which afforded the corresponding 2-amino-1,3-selenezoles in moderate to high yields [7]. Here, α-iodoketones are formed in situ and subsequently undergo cyclisation with selenourea. In the late 1960s the group of Bulka published two papers (in German) on the preparation of 2-arylamino-1,3-selenazoles and their reactivity [8,9]. The free amines could be acetylated in acetic anhydride and the reaction with NaNO_2_ in acetic acid (in situ formed nitrous acid) gave yellow products, which were (erroneously) proposed to be the corresponding *N*-nitrosamines (Scheme 1); functionalization at position 5 was explicitly excluded [8]. However, the reaction with nitroso anilines gave C5-substituted azomethines (Scheme 1) [8]. Phenyldiazonium chloride reacted with the selenazoles to give the C5-azo product, confirming the results of Haginiwa. In all of these reactions, the selenazole heterocycle itself remained intact. The chemistry of selenazoles was summarized in early reviews by Bulka and, more recently, by Proshin [10,11,12].

We previously reported some aspects of the chemistry of aryl-selenazoles derived from acylselenoureas, as well as the biological activity of various selenium-containing metal complexes [13,14,15]. Selenium-containing compounds in general experienced a recent renaissance, especially due to their promising medical applications [16,17]. As an example, the benzoselenazolone known as Ebselen, apart from being anti-inflammatory and an antioxidant, was also shown to be an active small molecule inhibitor against SARS-CoV-2 [18,19]. Given that much of the chemistry of 2-arylamino-1,3-selenazoles is quite old and results are at times contradictory, we wished to revisit this chemistry and study the reactivity and the structures of the products with modern spectroscopic methods and X-ray diffraction. We also report the biological activity of selected compounds in various human tumor cell lines and a variety of bacteria and fungi.

## 2. Results and Discussion

**Figure 1:**
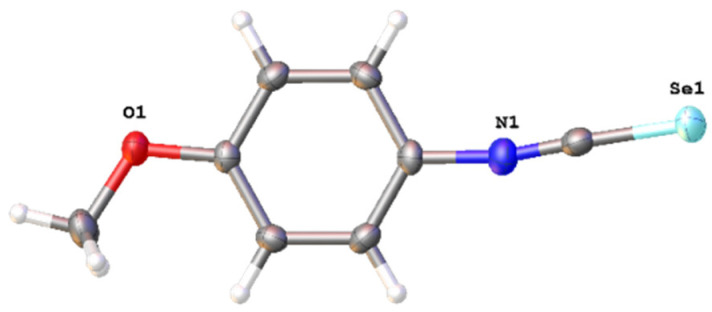
Molecular structure of compound **1c**. Ellipsoids are drawn at 50% probability level.

**Scheme 2:**
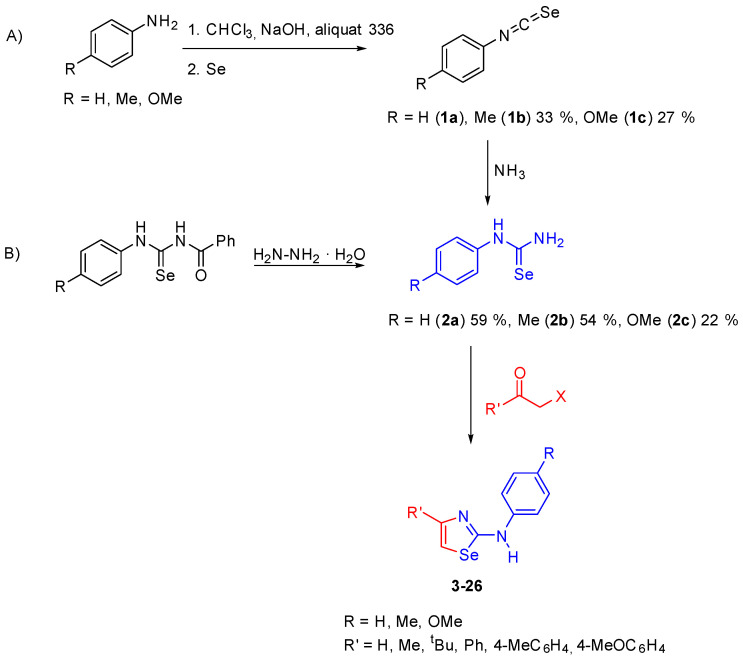
Syntheses of 2-amino-1,3-selenazoles from aryl isoselenocyanates (**A**) or acylselenoureas (**B**).

A library of functionalized 2-arylamino-1,3-selenazoles was prepared in three steps, starting from arylisoselenocyanates (Scheme 2). The required arylisoselenocyanates ArNCSe (Ar = Ph, 4-MeOC_6_H_4_, 4-MeC_6_H_4_) were synthesized in a one-pot, two-step procedure from the corresponding anilines using a modified literature procedure (Scheme 2A) [20]. The compounds are rather malodorous substances, which do not keep well and deposit red selenium. Despite of this, we managed to obtain single crystals suitable for X-ray diffraction of **1c** (Figure 1).

The subsequent reaction of the arylisoselenocyanates with methanolic ammonia solution furnished the corresponding known arylselenoureas ArNHC(Se)NH_2_ (Ar = Ph, 4-MeOC_6_H_4_, 4-MeC_6_H_4_) (Scheme 2) [21].

**Figure 2:**
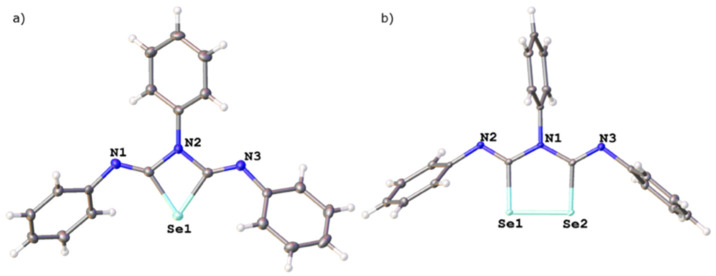
(**a**) Molecular structure of (2Z,4Z)-*N*,*N*^′^,*N*^″^-triphenyl-1,3-selenazetidine-2,4-diimine **1x**. (**b**) Molecular structure of (3Z,5Z)-*N*,*N*^′^,*N*^″^-triphenyl-1,2,4-diselenazolidine-3,5-diimine **1y**. Ellipsoids are drawn at 50% probability level.

During one preparation of PhNCSe (**1a**) we isolated a small amount of material, which was not the expected product as evident from its proton NMR spectrum. By fractional crystallization we could separate a colorless and yellow product (in 2% and 1% yield based on PhNH_2_). An X-ray diffraction study revealed the two compounds to be the 1,3-selenazetidine **1x** (colorless) and the 1,2,4-diselenazolidine **1y** (yellow) shown in Figure 2a,b, respectively. 1,3-Selenazetidines were previously prepared from the reaction of isoselenocyanates with carbodiimides [22,23]. We may explain the formation of this unusual side product by the fact that during the course of the one-pot reaction some PhNCSe reacts with PhN=C=NPh, which in turn was produced (albeit in very small quantities) from PhNC reacting with aniline. However, Koketsu failed to isolate any 1,3-selenazetidines using this reaction [23]. The formation of the 1,2,4-diselenazolidine may be explained by two sequential nucleophilic additions of aniline to PhNCSe, followed by oxidation of the intermediate. This mechanism is consistent with that proposed by Yavari for the formation of functionalized 1,2,4-diselenazolidenes from acyl isoselenocyanates [24]. A structurally similar diselenazole was isolated by Woollins as an unexpected product in the reaction of an acyl isoselenocyanate with an aromatic amine [25]. The Se–Se distance in this compound is with 2.397(3) Å longer than what is observed in our compound [Se-Se = 2.3396(3) Å]. No mechanistic details explaining how the compound possibly formed are given in the publication.

**Figure 3:**
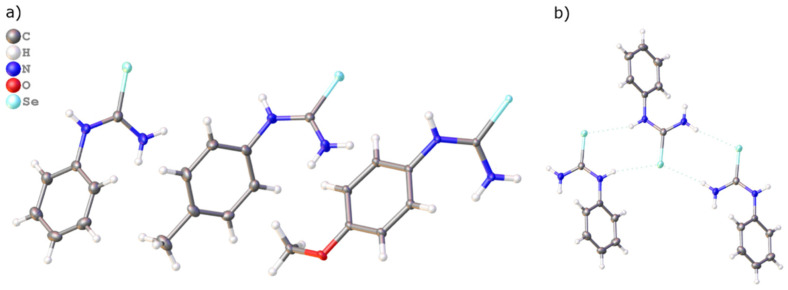
(**a**) Molecular structures of compound **2a** (left), **2b** (middle), **2c** (right). (**b**) Hydrogen bonds between molecules of **2a**. Ellipsoids are drawn at 50% probability level.

To reduce the amount of side products, an alternative synthesis for the arylselenoureas was developed. Based on the solvent-free synthesis of Kodomari et al., the *N*-aryl-*N*^′^-benzoylselenoureas were treated with an excess of hydrazine hydrate (Scheme 2 B). Upon addition of cold water, the products precipitated together with some red elemental selenium. Purification was easily achieved by recrystallization from EtOH. Overall, this method is faster and less odorous than the isoselenocyanate route [26]. Given that no X-ray crystal structures of such arylselenoureas were so far reported, we determined the molecular structures of **2a**–**2c** by X-ray diffraction (Figure 3a). In the solid-state the molecules from an infinite zig-zag chain through N-H-Se hydrogen bonds between selenium and NH_2_ groups from neighboring molecules (Figure 3b).

**Figure 4:**
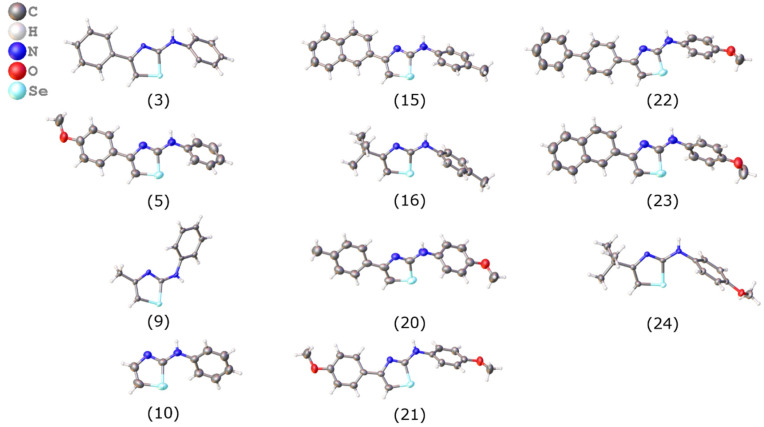
Molecular structures of compounds **3**, **5**, **9**, **10**, **15**, **16**, **20**–**24**. Ellipsoids are drawn at 50% probability level.

The 2-arylamino-1,3-selenazoles were subsequently obtained from the cyclocondensation reaction of these arylselenoureas with various 2-haloketones in the presence of Et_3_N (Scheme 2) [8,9,27]. The products and their yields are collected in Table 1. Yields are generally quite high, however, the alkyl- or proton-substituted derivatives are sometimes formed in significantly lower yields. This may be due to the generally lower stability of these compounds in solution. In the solid-state however, the compounds are stable for months. Some of the compounds were previously reported, however, characterization was restricted to melting points and elemental analysis. Thus, all of the selenium heterocycles reported here were fully characterized by NMR spectroscopy, mass spectrometry and elemental analysis. In the case of heterocycles **3**, **5**, **9**, **10**, **15**, **16**, **20**–**24**, we determined the molecular structures by single-crystal X-ray diffraction (Figure 4). In the ^1^H NMR spectra of the 1,3-selenazoles, coupling between the proton at position 5 and the selenium nucleus can be observed in form of satellites with H-Se coupling constants of around 48 Hz. This allowed us to record 2D ^1^H-^77^Se HMBC spectra, which provided ^77^Se NMR data much faster (16 min.) and with significantly less material than required for directly recorded spectra.

**Figure 5:**
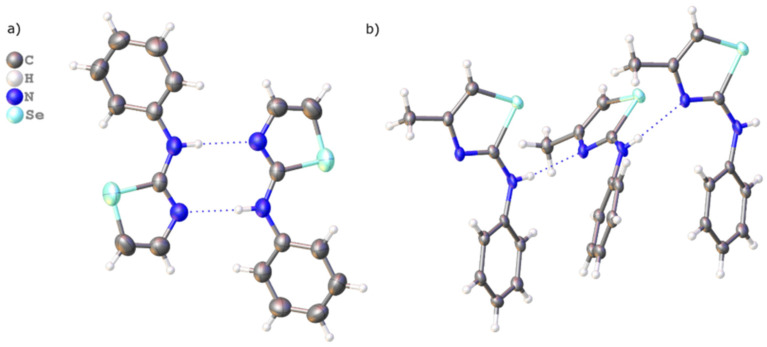
Hydrogen bonding in molecules **10**. (**a**) Dimer formation. (**b**) Chain formation.

In most cases, the X-ray crystal structures show the formation of dimers due to hydrogen bonds between the NH-group and the nitrogen atom N3 of the adjacent heterocycle (Figure 5). An exception is compound **9**, which forms an infinite polymeric chain rather than a dimer (Figure 5). This is probably due to a packing effect in the crystal.

**Scheme 3:**
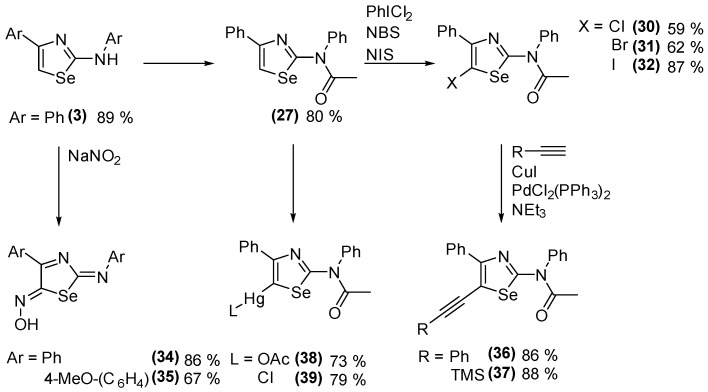
Reactions of 2-aminoaryl-4-aryl-1,3-selenazoles.

**Figure 6:**
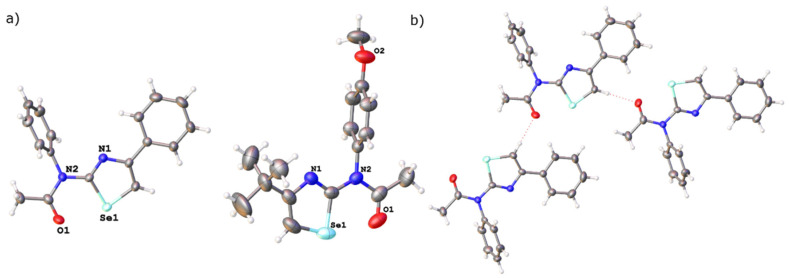
(**a**) Molecular structure of **27** (left) and **29** (right). (**b**) Hydrogen bonds between molecules of **27**.

We subsequently examined possible methods to further functionalize the 2-arylamino-1,3-selenazoles at various positions (Scheme 3). The 2-amino group was easily acetylated by heating the parent compounds in acetic anhydride for a few minutes. The presence of the acetamide is clearly confirmed by NMR spectroscopy and X-ray diffraction (Figure 6a). Between independent molecules, hydrogen bonds are formed between the CH group of the heterocycle and the acetate oxygen atom (Figure 6b). The stability of the acetylated compounds in solution seems to be higher than that of the parent compounds: the amines turn yellow and red in solution over time, whereas solutions of the acetylated derivatives remain unchanged for several days.

**Figure 7:**
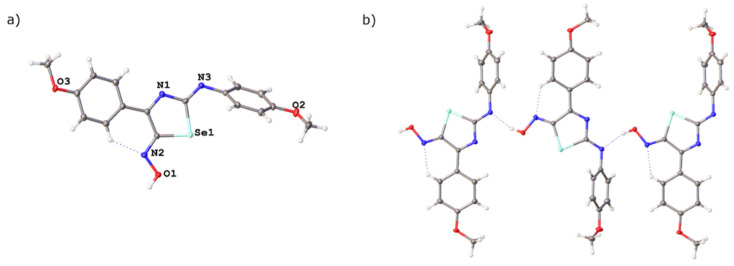
(**a**) Molecular structure of **35**. (**b**) Hydrogen bonds between molecules of **35**.

Bulka reported that 2-arylamino-1,3-selenazoles react with NaNO_2_ in acetic acid at room temperature to give the corresponding *N*-nitrosamines [8]. When we reproduced this reaction, we isolated a red-orange product, in which the resonances of both the amine proton and the proton at position 5 of the heterocycle were missing in the ^1^H NMR spectrum. Furthermore, a new signal at about 14 ppm was observed, suggesting the presence of an OH group. Through a single-crystal X-ray diffraction experiment we could confirm that the product is in fact not the *N*-nitrosamine, instead a hydroxylamine moiety is introduced at position 5 of the selenazole and, in addition, the amine is deprotonated. Hydrogen bonds are formed between N3 and the OH group of the neighboring molecule (Figure 7).

The inductive effect of the 2-arylamino group directs negative charge towards the 5-position. Thus, C5 can react with the nitrogen atom of nitrous acid to form the C-N bond. Finally, elimination of water gives the azomethine. As evident from the molecular structure, the system is extensively conjugated.

**Figure 8:**
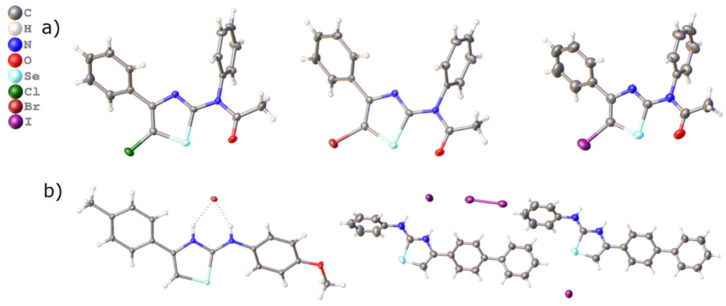
(**a**) Molecular structures of **30** (left), **31** (center) and **32** (right). (**b**) Molecular structures of HBr salt of **20** (left) and HI salt of **6** (right).

Attempted halogenation of the 2-arylamino-1,3-selenazoles with bromine or iodine only afforded the corresponding hydrohalide salts (Figure 8b). However, when reacting the *N*-acetyl compounds with iodine or bromine, poor yields of the 5-halo-selenazoles could be isolated. Gratifyingly, when we used *N*-bromo- or *N*-iodosuccinimide as halide source, the corresponding 5-halo-selenazoles were formed in high yields (Figure 8a). The 5-chloro-compound was prepared similarly using PhICl_2_. The characteristic signal for the CH proton with the ^77^Se-satelites is missing in the proton NMR spectra of the 5-halo-selenazoles, consistent with its substitution. In the carbon NMR spectra, the chemical shifts of the resonance of the carbon atom bound to the halogen atom are shifted, depending on the halide.

**Figure 9:**
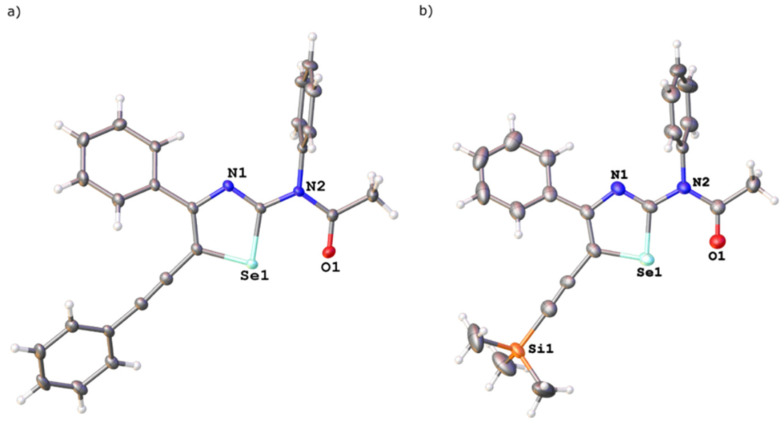
Products of Sonogashira coupling reactions. (**a**) Molecular structure of **36**. (**b**) Molecular structure of **37**.

Furthermore, we examined the ability of the iodo- and bromo-compounds to react in a Sonogashira coupling reaction with phenylacetylene or TMS-acetylene. In the ^77^Se NMR spectra of the products, signals are observed at 753 ppm (compound **36**) and 755 ppm (compound **37**), both are shifted with respect to those of their parent halo-selenazoles. In addition, X-ray crystal structures were obtained for both alkynes (Figure 9) confirming formation of the alkynes.

**Figure 10:**
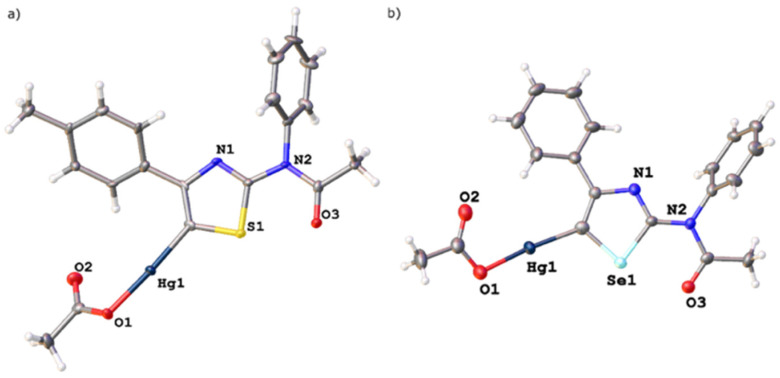
(**a**) Mercurated 2-amino-4-(*p*-tolyl)-1,3-thiazole (**38S**). (**b**) Mercurated 2-amino-4-phenyl-1,3-selenazole (**38**).

The mercuration of the sulfur-counterparts (2-amino-4-aryl-1,3-thiazoles) is described in literature several times. However, the position of the mercury atom in the products was not established with certainty. Both mercuration at position 5 of the heterocycle, as well as mercuration of the *N-*aryl rings were proposed [26,28,29]. We were therefore interested in the mercuration of selenazole derivatives, which to our knowledge have not been described at all and to numinously establish the position of mercuration. Initially, we re-examined the mercuration of 2-amino-4-(*p*-tolyl)-1,3-thiazole with Hg(OAc)_2_ and isolated a material, which based on NMR spectroscopy and X-ray diffraction was mercurated at position 5 of the heterocycle (Figure 10).

**Scheme 4:**
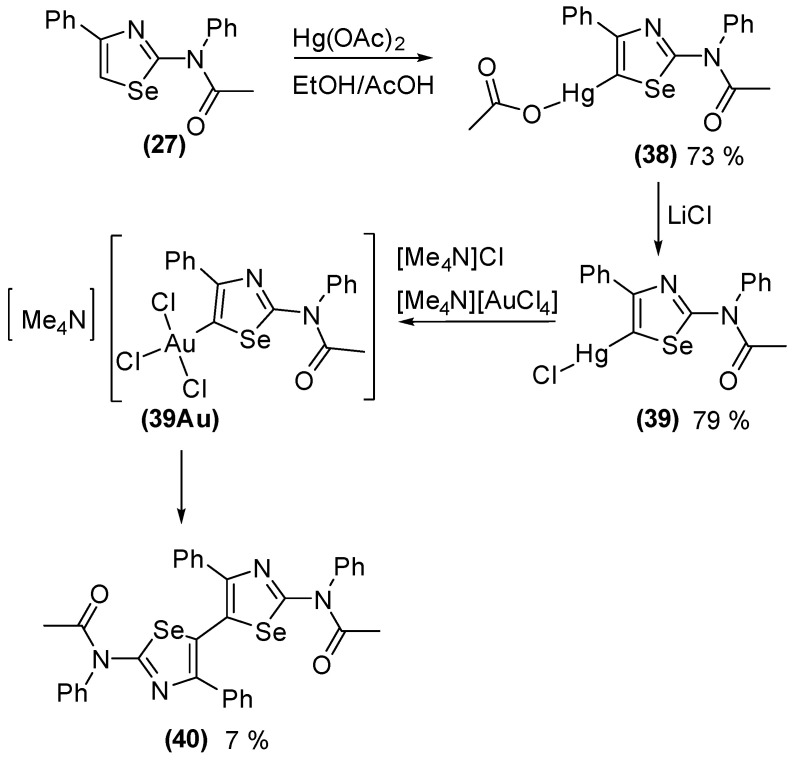
Transmetallation reactions.

**Figure 11:**
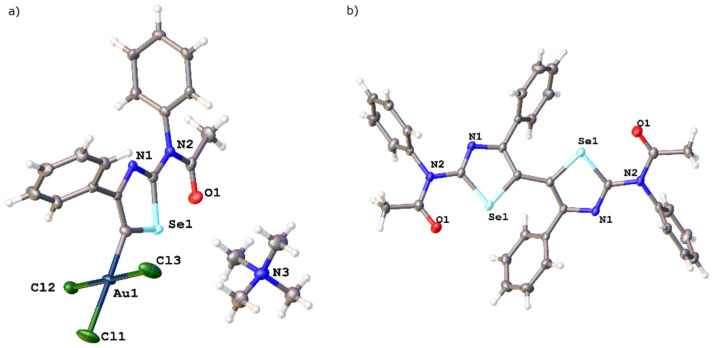
(**a**) Molecular structure of aurated 2-acetamidophenyl-4-phenyl-1,3-selenazole (**39Au**); (**b**) Molecular structure of C-C coupling product (**40**).

It was also possible to synthesize the selenium counterpart (**38**) by mercuration of 2-acetamidophenyl-4-phenyl-1,3-selenazole using mercury(II) acetate in a 1:1 mixture of EtOH and acetic acid. Similar to the halogenation reactions, it is important to protect the amino group in position 2, otherwise insoluble solids formed, which could not be characterised. In this case too, mercuration at position 5 of the heterocycle was unambiguously confirmed by NMR spectroscopy and X-ray diffraction (Figure 10b). The next goal was to attempt a transmetallation to gold using the reaction conditions reported by Vicente [30]. Indeed, stirring a solution of **38** with [Me_4_N][AuCl_4_] in the presence of [Me_4_N]Cl afforded a yellow-orange solution, which deposited a few single crystals after some time (Scheme 4). The crystals were identified as the organogold(III) salt **39Au**, in which the selenazole is bound to the metal through C5 (Figure 11a). The species seems to be rather unstable in solution; any attempts to isolate larger quantities for spectroscopic characterisation failed. If the reaction is left to stir for 3 days, a colorless solid was isolated in low (7%) yield after work-up (Scheme 4).

Based on spectroscopic data and X-ray diffraction, the material was identified as the bis(selenazole) **40**, formed by coupling of two selenazoles at position 5 (Figure 11b). The only other known examples of bis(selenazoles) are intensely colored compounds formed by FeCl_3_ mediated oxidative coupling of 2-selenazolylhydrazones [31]. In our case, conjugation is not possible, hence the C-C bond length between the two heterocycles is clearly a single bond [C-C = 1.457(4) Å]. Of interest is of course how the formation of this unexpected product can be explained. Carbon–carbon coupling by reductive elimination from organogold(III) species is a key-step in gold(III)-catalysis and was known for some time [32,33,34,35,36]. It is likely that in the reaction reported here, the initially formed mono-substituted gold(III) salt (**39Au**) reacts with a further equivalent of the mercury compound **39**, resulting in transmetallation of a second selenazolyl-moiety to gold. This species can subsequently undergo reductive elimination affording the C–C coupled product **40** and [AuCl_2_]^−^. Mass spectrometric studies (negative-ion electrospray mass spectra) of samples taken directly from the reaction mixture gave us a glimpse of some of the species involved. Initially, signals at *m/z* 642 and 306 due to **39Au** and [Hg_2_Cl_6_]^2−^, respectively were detected. The latter dianion results from the reaction of HgCl_2_ (by-product of transmetallation) with [Me_4_N]Cl and dimerization. At later stages of the reaction, an intense signal at *m/z* 266 corresponding to the gold(I) anion [AuCl_2_]^−^, which is the species remaining after reductive elimination, is seen. Unfortunately, the di-selenazolyl-substituted gold(III) anion was not detected, probably due to its tendency to rapidly undergo reductive elimination. Overall, these mass spectroscopic data, combined with X-ray diffraction data from isolated compounds, support the aforementioned reaction pathway. Key feature here is the successful sequential transfer of two selenazolyl-groups from mercury to gold.

While a detailed discussion of all the molecular structures reported herein would be too much, some general trends are worth mentioning. The geometry of the five-membered ring remains very similar, no matter what chemical modifications (acylation, halogenation, metallation, etc.) are made in the molecule. Both the angle subtended at the selenium atom (∠C-Se-C) as well as the two C-Se bond distances remain virtually constant, with values of 83.5 ± 0.5°, 1.86 ± 0.02 and 1.89 ± 0.01 Å, respectively. An exception are the structures of the three halohalide salts, in which the angle at selenium is ca. two degrees larger (∠C-Se-C = 85.5°). The C-Se and C-N bond lengths in the halohalide salts however fall in the same range as those of the neutral compounds.

### 2.1. Cytotoxicity

Cytotoxicity screening of compounds **3**–**5**, **7**, **11**, **15**, **19**, **21**, **23** and **27** was performed at a concentration of 10 µM on the NCI-60 panel of cancer cells. Neither a proliferative nor a significant antiproliferative activity could be observed. Thus, no further concentration dependent tests were performed.

### 2.2. Antimicrobial and Antifungal Activity

The antimicrobial activity of the complexes **3**–**29** was studied in seven pathogens, including the bacteria *Staphylococcus aureus*, *Escherichia coli*, *Klebsiella pneumoniae*, *Pseudomonas aeruginosa* and fungi *Candida albicans* and *Cryptococcus neoformans* var. *grubii*. Initially, the compounds were screened at a concentration of 32 µg/mL. Compounds **4**, **5**, **8**, **9**, **10**, **12**, **16**, **17**, **20**, **25** and **26** showed significant activity in at least one of the three microorganisms *S. aureus*, *C. albicans* and *C. neoformans* var. *grubii*. No activity was observed in Gram-negative bacteria. Minimum inhibitory concentrations (MIC) for *S. aureus*, *C. albicans* and *C. neoformans* var. *grubii* are shown in Table 2. In addition, the cytotoxicity to human embryonic kidney cells (HEK-293) and the hemolytic activity on human red blood cells (RBS) was studied. CC_50_ for HEK-293 and HC_10_ for RBC are also shown in Table 2.

The MIC, CC_50_, and HC_50_ values were converted to nmol/mL to allow better comparison. In general, the activity against fungi is higher than against *S. aureus*. Compounds **8**, **17**, **25,** and **26** are classified as at least partially toxic to human cells, whilst the other compounds show no significant toxicity. Especially compounds **5** and **9** show no significant toxicity but high activity against the two tested fungi. The MIC value of **5** in *C. albicans* and *C. neoformans* var. *grubii* is 12 nmol/mL. The MIC value of **9** is slightly lower with 8 nmol/mL. It is noticeable that even a small change of the structure leads to a very different activity. For example, compounds **4**, **12,** and **20** bear a 4-tolyl group in position 4 and show some activity, whilst compounds **2**, **10,** and **19** with a phenyl group in position 4 are basically inactive. It is difficult to compare these data exactly to those from other groups since different strains of microorganisms are used. Laczkowski synthesized hydrazinyl thiazole and selenazole derivatives and studied their activity in different *Candida* strains, including several strains of *C. albicans*. Laczkowski determined MIC values of 31–250 µg/mL for the selenazoles and values ranging from 0.49 to 7.81 µg/mL for the thiazoles, similar to our MIC values for the 2-amino-1,3-selenazoles. The activity against *S. aureus* was also tested by Laczkowski with MIC values for the selenazoles from 31 to 125 µg/mL, comparable to our results [37].

## 3. Materials and Methods

Reactions were carried out under aerobic conditions without protection from air or moisture unless stated otherwise. Solvents were HPLC quality stored over 3 Å molecular sieves. NMR spectroscopic data for the arylselenoureas is provided below since it was not given in the original publication. All other chemicals were obtained from commercial suppliers and were used as received. NMR spectra were recorded on Bruker Avance 400 or Bruker Avance III 600 instruments (Coventry, UK). Spectra were referenced externally to Me_4_Si (^1^H, ^13^C) and Me_2_Se (^77^Se). ^1^H-^77^Se HMBC experiments were recorded with ^1^H and ^77^Se 90° pulses of 13.5 µs and 11.9 µs, respectively. A cross magnetization relaxation time d1 of 1.5 s, a delay time of 10 ms (optimized to match a coupling constant of ca. 50 Hz), an overall evolution time of 400 µs and an acquisition time of 0.255 s were used. The spectral windows in the ^1^H and ^77^Se dimensions were 10 ppm (4001 Hz) and 200 ppm (15,271 Hz), respectively. Typically, 4 transients were accumulated for 128 increments in t1. Elemental analyses were performed by staff of the in-house elemental analysis facility using an Elementar Vario EL system. Cytotoxicity screening was performed by staff of the Developmental Therapeutics Program, Division of Cancer Treatment & Diagnosis of the National Cancer Institute (USA) using the NCI-60 panel of cells. Antimicrobial screening was performed by CO-ADD (The Community for Antimicrobial Drug Discovery), funded by the Wellcome Trust (UK) and The University of Queensland (Australia). Full experimental details are given in the Supplementary Materials.

### 3.1. Preparation of the Arylisoselenocyanates

The arylisoselenocyanates were prepared by a method reported in the literature [20]. Spectroscopic data was identical to that in the literature. Crystals suitable for X-ray diffraction of **1c** were obtained by recrystallisation from EtOH. During one preparation of **1a**, a yellow fraction was collected by column chromatography. Recrystallisation form an acetone/hexanes mixture afforded a small quantity of colorless (**1x**) and yellow crystals (**1y**) suitable for X-ray diffraction.

### 3.2. Preparation of the Arylselenoureas (via Method B)

Hydrazine hydrate (6 equiv.) was added to the solid *N*-aryl-*N*^′^-benzoylselenourea (1 equiv.) while cooling in ice-water. The ice-bath was then removed, and the mixture was stirred at room temperature for 5 min. After this time, the mixture was poured into ice-water, which precipitated the product. Recrystallization from a suitable solvent afforded the pure compounds.

### 3.3. PhNHC(Se)NH_2_ ***2a***

Method B: this was prepared as described above using hydrazine hydrate (8.0 mL, 0.160 mol) and *N*-phenyl-N^′^-benzoylselenourea (7.66 g, 0.025 mol). Recrystallization from EtOH gave the colorless product (2.9663 g, 0.01490 mol, 59%). ^1^H-NMR (400 MHz, DMSO-d_6_) δ = 7.15–7.21 (m, 1 H, *p*-Ph), 7.28–7.19 (m, 4 H, *o*-Ph, *m*-Ph), 7.56 (br. s, 1 H, NH_2_), 8.27 (br. s, 1 H, NH_2_), 10.02 (s, 1 H, NH). ^13^C-NMR (101 MHz, DMSO-d_6_) δ = 123.8 (*o*-Ph), 125.3 (*p*-Ph), 128.5 (*m*-Ph), 138.5 (*ipso*-Ph), 176.8 (CSe). ^77^Se-NMR (76 MHz, MeOH-d_4_, 248 K) δ = 183.4 (s).

### 3.4. 4-MeC_6_H_4_NHC(Se)NH_2_ ***2b***

Method B: this was prepared as described above using hydrazine hydrate (0.11 mL, 2.2 mmol) and *N*-tolyl-N^′^-benzoylselenourea (0.102 g, 0.32 mmol). Recrystallization from 1-BuOH gave the colorless product (0.023 g, 0.17 mmol, 54%). ^1^H-NMR (400 MHz, DMSO-d_6_) δ = 2.27 (s, 3 H, Me), 7.16 (s, 4 H, C_6_H_4_), 7.34 (br. s, 1 H, NH_2_), 8.17 (br. s, 1 H, NH_2_), 9.92 (s, 1 H, NH). ^13^C-NMR (101 MHz, DMSO-d_6_) δ = 20.5 (Me), 124.0 (C_6_H_4_), 129.4 (C_6_H_4_), 134.6 (Me*C*) 138.8 (CN), 176.6 (CSe). ^77^Se-NMR (76 MHz, MeOH-d_4_, 248 K) δ = 175.9 (s).

### 3.5. 4-MeOC_6_H_4_NHC(Se)NH_2_ ***2c***

Method B: this was prepared as described above using hydrazine hydrate (0.09 mL, 1.8 mmol) and *N*-methoxyphenyl-N^′^-benzoylselenourea (0.1010 g, 0.30 mmol). Recrystallization from EtOH gave the colorless product (0.0152 g, 0.07 mmol, 22%). ^1^H-NMR (600 MHz, DMSO-d_6_) δ = 3.75 (s, 3 H, Me), 6.89–6.94 (m, 2 H, C_6_H_4_), 7.17 (d, *J* = 8.5 Hz, 2 H, C_6_H_4_), 7.39 (br. s, 1 H, NH_2_), 8.11 (br. s, 1 H, NH_2_), 9.82 (s, 1H, NH). ^13^C-NMR (151 MHz, DMSO-d_6_) δ = 55.3 (Me), 114.2 (C_6_H_4_), 126.1 (C_6_H_4_), 131.2 (MeO*C*), 157.1 (CN). 176.4 (CSe).

### 3.6. Preparation of the 2-Arylamino-1,3-selenazoles

A mixture of aryl selenourea and the α-haloketone derivative (1 equiv.) in EtOH (10 mL) was heated to 80 °C for ca. 5 min. Et_3_N (1.3 equiv.) was added to the hot solution. After a further 5 min. of heating, the mixture was filtered hot to remove some elemental selenium. Addition of water to the filtrate precipitated the product, which was isolated by filtration. The products were purified by recrystallization from EtOH.

### 3.7. 2-Phenylamino-4-phenyl-1,3-selenazole ***3***

This was prepared as described above using PhNH(Se)NH_2_ (1.03 g, 5.17 mmol) and 2-bromoacetophenone (1.03 g, 5.17 mmol). The product was obtained as beige solid in 89% yield (1.36 g). ^1^H-NMR (400 MHz, DMSO-d_6_) δ = 6.99 (t, *J* = 7.3 Hz, 1 H, *p*-Ph_B_), 7.30 (t, *J* = 7.3 Hz, 1 H, *p*-Ph_A_), 7.33–7.38 (m, 2 H, *m*-Ph_B_), 7.42 (t, *J* = 7.6 Hz, 2 H, *m*-Ph_A_), 7.75 (d, *J* = 7.6 Hz, 2 H, *o-*Ph_B_), 7.78 (s, 1 H, ^2^*J*_H-Se_ = 49 Hz, CH), 7.92 (dd, *J* = 1.2 Hz, 8.31 Hz, 2 H, o-Ph_A_), 10.35 (s, 1 H, NH). ^13^C-NMR (101 MHz, DMSO-d_6_) δ = 107.9 (CH), 116.9 (*o*-Ph_B_), 121.4 (*p*-Ph_B_), 125.9 (*o*-Ph_A_), 127.3 (*p*-Ph_A_), 128.6 (*m*-Ph_A_), 129.0 (*m*-Ph_B_) 135.4 (*ipso*-Ph_A_), 141.2 (*ipso*-Ph_B_), 150.9 (C4), 164.1 (CSe). ^1^H-^77^Se-HMBC (400 MHz, 76 MHz, DMSO-d_6_) δ = 622.6 (s). High-resolution MS: calculated for [C_15_H_13_N_2_Se]^+^ 301.0244; found 301.0258. Elemental analysis for C_15_H_12_N_2_Se calculated: H 4.04, C 60.21, N 9.36; found H 3.97, C 60.19, N 9.27.

### 3.8. 2-Phenylamino-4-(4-tolyl)-1,3-selenazole ***4***

This was prepared as described above using PhNH(Se)NH_2_ (0.209 g, 1.05 mmol) and 2-bromo-4**′**-methylacetophenone (0.214 g, 1.00 mmol). The product was obtained as pale-yellow solid in 69% yield (0.218 g). ^1^H-NMR (400 MHz, DMSO-d_6_) δ = 2.32 (s, 3 H, Me), 6.99 (t, *J* = 7.4 Hz, 1 H, *p*-Ph), 7.22 (d, *J* = 8.1 Hz, 2 H, C_6_H_4_), 7.32–7.38 (m, 2 H, *m*-Ph), 7.69 (s, 1 H, CH), 7.75 (d, *J* = 7.7 Hz, 2 H, C_6_H_4_), 7.80 (d, *J* = 8.1 Hz, 2 H, o-Ph), 10.34 (s, 1 H, NH). ^13^C-NMR (101 MHz, DMSO-d_6_) δ = 20.8 (Me), 106.8 (CH), 116.9 (*o*-Ph), 121.4 (*p*-Ph), 125.8 (C_6_H_4_), 129.0 (*m*-Ph), 129.1 (C_6_H_4_), 132.8 (C_6_H_4_), 136.5 (C_6_H_4_), 141.2 (*ipso*-Ph), 150.9 (C4), 164.0 (CSe). ^1^H-^77^Se-HMBC (400 MHz, 76 MHz, DMSO-d_6_) δ = 614.9 (s). High-resolution MS: calculated for [C_16_H_15_N_2_Se]^+^ 315.0401; found 315.0370. Elemental analysis for C_16_H_14_N_2_Se calculated: H 4.50, C 61.35, N 8.94; found H 4.48, C 61.18, N 8.79.

### 3.9. 2-Phenylamino-4-(4-methoxyphenyl)-1,3-selenazole ***5***

This was prepared as described above using PhNH(Se)NH_2_ (0.193 g, 0.97 mmol) and 2-bromo-4**′**-methoxyacetophenone (0.227 g, 0.99 mmol). The product was obtained as pale-orange solid in 71% yield (0.227 g). ^1^H-NMR (400 MHz, DMSO-d_6_) δ = 3.79 (s, 3 H, OMe), 6.95–7.00 (m, 3 H, C_6_H_4_, *p*-Ph), 7.32–7.38 (m, 2 H, *m*-Ph), 7.60 (s, ^2^*J*_H-Se_ = 49 Hz, 1 H, CH), 7.74 (d, *J* = 7.6 Hz, 2 H, *o*-Ph), 7.84 (d, *J* = 8.9 Hz, 2 H, C_6_H_4_), 10.32 (s, 1 H, NH). ^13^C-NMR (101 MHz, DMSO-d_6_) δ = 55.1 (OMe), 105.5 (CH), 113.9 (C_6_H_4_), 116.9 (*o*-Ph), 121.4 (*p*-Ph), 127.2 (C_6_H_4_), 128.3 (C_6_H_4_), 129.0 (*m*-Ph), 141.3 (*ipso*-Ph), 150.7 (C4), 158.6 (C_6_H_4_), 163.9 (CSe). ^1^H-^77^Se-HMBC (400 MHz, 76 MHz, DMSO-d_6_) δ = 616.1 (s). High-resolution MS: calculated for [C_16_H_15_N_2_OSe]^+^ 331.0350; found 331.0290. Elemental analysis for C_16_H_14_N_2_OSe calculated: H 4.29, C 58.37, N 8.51; found H 4.15, C 57.81, N 8.19.

### 3.10. 2-Phenylamino-4-(4-biphenyl)-1,3-selenazole ***6***

This was prepared as described above using PhNH(Se)NH_2_ (0.198 g, 0.99 mmol) and 2-bromo-4**′**-phenylacetophenone (0.272 g, 0.99 mmol). The product was obtained as yellow solid in 74% yield (0.276 g). ^1^H-NMR (400 MHz, DMSO-d_6_) δ = 7.00 (tt, *J* = 7.3, 1.0 Hz, 1 H, *p*-Ph_B_), 7.33–7.40 (m, 3 H, *p*-Ph_A_, *m*-Ph_B_), 7.48 (t, *J* = 7.6 Hz, 2 H, *m*-Ph_A_), 7.70–7.75 (m, 4 H, C_6_H_4_, *o*-Ph_A_), 7.78 (dd, *J* = 7.6, 1,1 Hz, 2 H, *o*-Ph_B_), 7.85 (s, 1 H, CH), 8.01 (d, *J* = 8.5 Hz, 2 H, C_6_H_4_), 10.39 (s, 1 H, NH). ^13^C-NMR (101 MHz, DMSO-d_6_) δ = 108.2 (CH), 116.9 (*o*-Ph_B_), 121.5 (*p*-Ph_B_), 126.4 (C_6_H_4_), 126.5 (*o*-Ph_A_), 126.8 (C_6_H_4_), 127.4 (*p*-Ph_A_), 128.9 (*m*-Ph_B_), 129.0 (*m*-Ph_A_), 134.5 (C_6_H_4_), 138.8 (C_6_H_4_), 139.7 (*ipso*-Ph_A_), 141.2 (*ipso*-Ph_B_), 150.5 (C4), 164.11 (CSe). ^1^H-^77^Se-HMBC (400 MHz, 76 MHz, DMSO-d_6_) δ = 619.2 (s). High-resolution MS: calculated for [C_21_H_17_N_2_Se]^+^ 377.0557; found 377.0433. Elemental analysis for C_21_H_16_N_2_Se calculated: H 4.30, C 67.20, N 7.46; found H 4.24, C 67.17, N 7.92.

### 3.11. 2-Phenylamino-4-(2-naphthyl)-1,3-selenazole ***7***

This was prepared as described above using PhNH(Se)NH_2_ (0.206 g, 1.03 mmol) and 2-bromo-2**′**-acetonaphthone (0.246 g, 0.99 mmol). The product was obtained as beige solid in 76% yield (0.260 g). ^1^H-NMR (400 MHz, DMSO-d_6_) δ = 7.01 (tt, *J* = 7.4, 1.1 Hz, 1 H, *p*-Ph), 7.36–7.42 (m, 2 H, *m*-Ph), 7.46–7.54 (m, 2 H, naph), 7.78 (dd, *J* = 8.6, 1.1 Hz, 2 H, *o*-Ph), 7.85–7.99 (m, 5 H, naph, CH), 8.03 (dd, *J* = 8.6, 1.7 Hz, 1 H, naph), 8.43 (s, 1 H, naph), 10.40 (s, 1 H, NH). ^13^C-NMR (101 MHz, DMSO-d_6_) δ = 109.0 (CH), 117.4 (*o*-Ph), 122.0 (*p*-Ph), 124.5 (naph), 124.8 (naph),126.2 (naph) 126.7 (naph), 127.8 (naph), 128.4 (naph), 129.4 (*m*-Ph), 132.6 (naph), 133.1 (naph), 133.5 (naph), 141.5 (*ipso*-Ph), 151.1 (C4), 164.6 (CSe). ^1^H-^77^Se-HMBC (400 MHz, 76 MHz, DMSO-d_6_) δ = 620.3 (s). High-resolution MS: calculated for [C_19_H_15_N_2_Se]^+^ 351.0401; found 351.0328. Elemental analysis for C_19_H_14_N_2_Se calculated: H 4.04, C 65.33, N 8.02; found H 3.98, C 65.43, N 7.92.

### 3.12. 2-Phenylamino-4-(tert-butyl)-1,3-selenazole ***8***

This was prepared as described above using PhNH(Se)NH_2_ (0.102 g, 0.51 mmol) and 1-bromopinacolone (0.089 g, 0.50 mmol). The product was obtained as pink solid in 52% yield (0.074 g). ^1^H-NMR (400 MHz, DMSO-d_6_) δ = 1.27 (s, 9 H, ^t^Bu), 6.83 (s, ^2^*J*_H-Se_ = 51 Hz, 1 H, CH), 6.93 (t, *J* = 7.4 Hz, 1 H, *p*-Ph), 7.26–7.32 (m, 2 H, *m-*Ph), 7.66 (d, *J* = 7.63 Hz, 2 H, *o*-Ph), 10.16 (s, 1 H, NH). ^13^C-NMR (101 MHz, DMSO)-d_6_) δ = 29.59 (^t^Bu), 35.14 (^t^Bu), 103.3 (CH), 116.7 (*o*-Ph), 121.1 (*p*-Ph), 128.9 (*m*-Ph), 141.5 (*ipso*-Ph), 162.0 (C4), 163.6 (CSe). ^1^H-^77^Se-HMBC (400 MHz, 76 MHz, DMSO-d_6_) δ = 587.2 (s). High-resolution MS: calculated for [C_13_H_17_N_2_Se]^+^ 281.05570; found 281.0580. Elemental analysis for C_13_H_16_N_2_Se calculated: H 5.78, C 55.92, N 10.03; found H 5.71, C 55.09, N 10.04.

### 3.13. 2-Phenylamino-4-methyl-1,3-selenazole ***9***

This was prepared as described above using PhNH(Se)NH_2_ (0.194 g, 0.97 mmol) and chloroacetone (0.093 g, 1.00 mmol). The product was obtained as an orange solid in 56% yield (0.131 g). ^1^H-NMR (400 MHz, DMSO-d_6_) δ = 2.19 (d, *J* = 1.1 Hz, 3 H, Me), 6.85 (s, ^2^*J*_H-Se_ = 50 Hz, 1 H, CH), 6.95 (tt, *J* = 7.5, 1,1 Hz, 1 H, *p*-Ph), 7.27–7.32 (m, 2 H, *m*-Ph), 7.63 (d, *J* = 7.9 Hz, 2 H, *o*-Ph), 10.18 (s, 1 H, NH). ^13^C-NMR (101 MHz, DMSO-d_6_) δ = 18.4 (Me), 106.2 (CH), 116.8 (*o*-Ph), 121.2 (*p*-Ph), 128.9 (*m*-Ph), 141.3 (*ipso*-Ph), 148.3 (CSe). ^1^H-^77^Se-HMBC (400 MHz, 76 MHz, DMSO-d_6_) δ = 588.5 (s). High-resolution MS: calculated for [C_10_H_11_N_2_Se]^+^ 239.0088; found 239.0099. Elemental analysis for C_10_H_10_N_2_Se calculated: H 4.25, C 50.64, N 11.81; found H 4.26, C 50.72, N 11.77.

### 3.14. 2-Phenylamino-1,3-selenazole ***10***

This was prepared as described above using PhNH(Se)NH_2_ (0.118 g, 0.59 mmol) and chloroacetaldehyde (0.079 g, 1.00 mmol). The product was obtained as an orange solid in 32% yield (0.071 g). ^1^H-NMR (400 MHz, DMSO-d_6_) δ = 6.95 (tt, *J* = 7.5, 1.1 Hz, 1 H, *p*-Ph), 7.19 (d, *J* = 4.2 Hz, 1 H, NCH), 7.25–7.34 (m, 3 H, *m*-Ph, CH), 7.65 (dd, *J* = 8.8, 1.1 Hz, 2 H, *o*-Ph), 10.27 (s, 1 H, NH). ^13^C-NMR (101 MHz, DMSO-d_6_) δ = 113.0 (CH), 116.9 (*o*-Ph), 121.2 (*p*-Ph), 128.9 (*m*-Ph), 139.4 (NCH), 141.3 (*ipso*-Ph), 166.0 (CSe). ^1^H-^77^Se-HMBC (400 MHz, 76 MHz, DMSO-d_6_) δ = 573.0 (s). High-resolution MS: calculated for [C_9_H_9_N_2_Se]^+^ 224.9931; found 224.9943. Elemental analysis for C_9_H_8_N_2_Se calculated: H 3.61, C 48.45, N 12.55; found H 3.67, C 48.61, N 12.23.

### 3.15. 2-Amino-(4-tolyl)-4-phenyl-1,3-selenazole ***11***

This was prepared as described above using 4-MeC_6_H_4_NHC(Se)NH_2_ (0.107 g, 0.54 mmol) and 2-bromoacetophenone (0.093 g, 0.47 mmol). The product was obtained as a pink solid in 40% yield (0.067 g). ^1^H-NMR (400 MHz, DMSO-d_6_) δ [ppm] = 2.27 (s, 3H, Me), 7.16 (d, *J* = 8.2 Hz, 2H, C_6_H_4_), 7.29 (t, *J* = 7.3 Hz, 1H, *p*-Ph), 7.41 (t, *J* = 7.6 Hz, 2H, *m*-Ph), 7.62 (d, *J* = 8.5 Hz, 2H, C_6_H_4_) 7.74 (s, 1H, CH), 7.91 (d, *J* = 7.1 Hz, 2H, *o*-Ph), 10.24 (s, 1H, NH). ^13^C-NMR (101 MHz, DMSO-d_6_) δ [ppm] = 20.4 (Me), 107.4 (CH), 117.1 (C_6_H_4_), 125.9 (*o*-Ph), 127.2 (*p*-Ph), 128.5 (*m*-Ph), 129.4 (C_6_H_4_), 130.4 (C_6_H_4_), 135.4 (*ipso*-Ph), 138.9 (C_6_H_4_), 150.9 (C4), 164.3 (CSe). ^1^H-^77^Se-HMBC (400 MHz, 76 MHz, DMSO-d_6_) δ [ppm] = 613.2. High-resolution MS: calculated for [C_16_H_15_N_2_Se]^+^ 315.0401; found 315.0443. Elemental analysis for C_16_H_14_N_2_Se calculated: H 4.50, C 61.35, N 8.94; found H 4.50, C 61.52, N 8.93.

### 3.16. 2-Amino-(4-tolyl)-4-(4-tolyl)-1,3-selenazole ***12***

This was prepared as described above using 4-MeC_6_H_4_NHC(Se)NH_2_ (0.213 g, 1.08 mmol) and 2-bromo-4**′**-methylacetophenone (0.256 g, 1.20 mmol). The product was obtained as a yellow solid in 58% yield (0.205 g). ^1^H-NMR (400 MHz, DMSO-d_6_) δ [ppm] = 2.26 (s, 3H, Me_B_), 2.34 (s, 3H, Me_A_), 7.18 (d, *J* = 8.2 Hz, 2H, C_6_H_4_-B), 7.23 (d, *J* = 8.0 Hz, 2H, C_6_H_4_-A), 7.61 (d, *J* = 8.4 Hz, C_6_H_4_-B), 7.64 (s, 1H, CH), 7.77 (d, *J* = 8.1 Hz, C_6_H_4_-A), 10.32 (s, 1H, NH). ^13^C-NMR (101 MHz, DMSO-d_6_) δ [ppm] = 20.4 (Me_B_), 20.8 (Me_A_), 106.4 (CH), 117.5 (C_6_H_4_-B), 125.8 (C_6_H_4_-A), 129.1 (C_6_H_4_-A), 129.4 (C_6_H_4_-B), 130.7 (C_6_H_4_-B), 132.4 (C_6_H_4_-A), 136.6 (C_6_H_4_-A), 138.7 (C_6_H_4_-B), 150.1 (C4), 164.9 (CSe). ^1^H-^77^Se-HMBC (400 MHz, 76 MHz, DMSO-d_6_) δ [ppm] = 106.2. High-resolution MS: calculated for [C_17_H_17_N_2_Se]^+^ 329.0557; found 329.0615. Elemental analysis for C_17_H_16_N_2_Se calculated: H 4.93, C 62.39, N 8.56; found H 4.52, C 56.03, N 7.62.

### 3.17. 2-Amino-(4-tolyl)-4-(4-methoxyphenyl)-1,3-selenazole ***13***

This was prepared as described above using 4-MeC_6_H_4_NHC(Se)NH_2_ (0.226 g, 1.14 mmol) and 2-bromo-4**′**-methoxylacetophenone (0.225 g, 0.98 mmol). The product was obtained as an orange solid in 48% yield (0.162 g). ^1^H-NMR (400 MHz, DMSO-d_6_) δ [ppm] = 2.26 (s, 3H, Me), 3.78 (s, 3H, OMe), 6.97 (d, *J* = 8.8 Hz, 2H, C_6_H_4_-OMe), 7.15 (d, *J* = 8.3 Hz, 2H, C_6_H_4_-Me), 7.54 (s, 1H, CH), 7.62 (d, *J* = 8.4 Hz, 2H, C_6_H_4_-Me), 7.83 (d, *J* = 8.8 Hz, 2H, C_6_H_4_-OMe), 10.21 (s, 1H, NH). ^13^C-NMR (101 MHz, DMSO-d_6_) δ [ppm] = 20.4 (Me), 55.1 (OMe), 105.0 (CH), 113.9 (C_6_H_4_-OMe), 117.1 (C_6_H_4_-Me), 127.2 (C_6_H_4_-OMe), 128.4 (C_6_H_4_-Me), 129.4 (C_6_H_4_-Me), 130.3 (C_6_H_4_-Me), 138.9 (C_6_H_4_-Me), 150.7 (C4), 158.6 (C_6_H_4_-OMe), 164.1 (CSe). ^1^H-^77^Se-HMBC (400 MHz, 76 MHz, DMSO-d_6_) δ [ppm] = 608.7. High-resolution MS: calculated for [C_17_H_17_N_2_OSe]^+^ 345.0506; found 345.0566. Elemental analysis for C_17_H_16_N_2_OSe calculated: H 4.70, C 59.48, N 8.16; found H 4.71, C 59.25, N 8.08.

### 3.18. 2-Amino-(4-tolyl)-4-(4-biphenyl)-1,3-selenazole ***14***

This was prepared as described above using 4-MeC_6_H_4_NHC(Se)NH_2_ (0.217 g, 1.09 mmol) and 2-bromo-4**′**-phenylacetophenone (0.267 g, 0.97 mmol). The product was obtained as a yellow solid in 58% yield (0.219 g). ^1^H-NMR (400 MHz, DMSO-d_6_) δ [ppm] = 2.27 (s, 3H, Me), 7.17 (d, *J* = 8.3 Hz, 2H, C_6_H_4_-B), 7.37 (t, *J* = 7.34 Hz, 1H, *p*-Ph), 7.48 (t, *J* = 7.6 Hz, 2H, *m*-Ph), 7.65 (d, *J* = 8.4 Hz, C_6_H_4_-B), 7.72 (m, 4H, *o*-Ph, C_6_H_4_-A), 7.80 (s, 1H, CH), 8.00 (d, *J* = 8.4 Hz, 2H, C_6_H_4_-A), 10.27 (s, 1H, NH). ^13^C-NMR (101 MHz, DMSO-d_6_) δ [ppm] = 20.3 (Me), 107.7 (CH), 117.1 (C_6_H_4_-B), 126.4 (*o-*Ph/C_6_H_4_-A), 126.8 (*o-*Ph/C_6_H_4_-A), 126.4 (C_6_H_4_-A), 127.3 (*p*-Ph), 128.9 (*m*-Ph), 129.4 (C_6_H_4_-B), 130.4 (C_6_H_4_-B), 134.5 (C_6_H_4_-A), 138.7 (C_6_H_4_-A) 138.9 (C_6_H_4_-B), 139.7 (*ipso-*Ph), 150.5 (C4), 164.3 (CSe). ^1^H-^77^Se-HMBC (400 MHz, 76 MHz, DMSO-d_6_) δ [ppm] = 615.0. High-resolution MS: calculated for [C_22_H_19_N_2_Se]^+^ 391.0714; found 391.0782. Elemental analysis for C_22_H_18_N_2_Se calculated: H 4.66, C 67.87, N 7.19; found H 7.12, C 67.82, N 7.12.

### 3.19. 2-Amino-(4-tolyl)-4-(2-naphtyl)-1,3-selenazole ***15***

This was prepared as described above using 4-MeC_6_H_4_NHC(Se)NH_2_ (0.217 g, 1.09 mmol) and 2-bromo-2**′**-acetonaphthone (0.248 g, 1.00 mmol). The product was obtained as a colorless solid in 61% yield (0.221 g). ^1^H-NMR (400 MHz, DMSO-d_6_) δ [ppm] = 2.29 (s, 3H, Me), 7.20 (d, *J* = 8.1 Hz, 2H, C_6_H_4_), 7.51 (m, 2H, naph), 7.67 (d, *J* = 8.4 Hz, 2H, C_6_H_4_), 7.88–8.00 (m, 4H, CH, naph), 8.05 (m, 1H, naph), 8.44 (s, 1H, naph), 10.30 (s, 1H, NH). ^13^C-NMR (101 MHz, DMSO-d_6_) δ [ppm] = 20.4 (Me), 108.3 (CH), 117.2 (C_6_H_4_-12), 124.3 (naph), 124.5 (naph), 125.8 (naph), 126.3 (naph), 127.5 (naph), 128.0 (naph), 128.1 (naph), 129.5 (C_6_H_4_), 130.5 (C_6_H_4_), 132.3 (naph), 132.9 (naph), 133.2 (naph), 138.9 (C_6_H_4_), 150.8 (C4), 164.4 (CSe). ^1^H-^77^Se-HMBC (400 MHz, 76 MHz, DMSO-d_6_) δ [ppm] = 614.9. High-resolution MS: calculated for [C_20_H_17_N_2_Se]^+^ 365.0557; found 365.0610. Elemental analysis for C_20_H_16_N_2_Se calculated: H 4.44, C 66.12, N 7.71; found H 4.50, C 66.13, N 7.63.

### 3.20. 2-Amino-(4-tolyl)-4-(tert-butyl)-1,3-selenazole ***16***

This was prepared as described above using 4-MeC_6_H_4_NHC(Se)NH_2_ (0.163 g, 0.82 mmol) and 1-bromopinacolone (0.137 g, 0.77 mmol). The product was obtained as a colorless solid in 84% yield (0.188 g). ^1^H-NMR (400 MHz, DMSO-d_6_) δ [ppm] = 1.26 (s, 9H, *^t^*Bu), 2.24 (s, 3H, Me), 6.80 (s, 1H, CH), 7.11 (d, *J* = 8.4 Hz, 2H, C_6_H_4_), 7.53 (d, *J* = 8.4 Hz, 2H, C_6_H_4_), 10.04 (s, 1H, NH). ^13^C-NMR (101 MHz, DMSO-d_6_) δ [ppm] = 20.3 (Me), 29.6 (*^t^*Bu), 35.1 (*^t^*Bu), 102.8 (CH), 116.9 (C_6_H_4_), 129.3 (C_6_H_4_), 129.9 (C_6_H_4_), 139.2 (C_6_H_4_), 162.0 (C4), 163.8 (CSe). ^1^H-^77^Se-HMBC (400 MHz, 76 MHz, DMSO-d_6_) δ [ppm] = 582.2. High-resolution MS: calculated for [C_14_H_19_N_2_Se]^+^ 295.0714; found 295.0745. Elemental analysis for C_14_H_18_N_2_Se calculated: H 6.19, C 57.34, N 9.55; found H 6.18, C 56.99, N 9.50.

### 3.21. 2-Amino-(4-tolyl)-4-methyl-1,3-selenazole ***17***

This was prepared as described above using 4-MeC_6_H_4_NHC(Se)NH_2_ (0.215 g, 1.09 mmol) and chloroacetone (0.092 g, 1.00 mmol). The product was obtained as an orange solid in 81% yield (0.203 g). ^1^H-NMR (400 MHz, DMSO-d_6_) δ [ppm] = 2.17 (d, *J* = 1.0 Hz, 3H, Me), 2.24 (s, 3H, C_6_H_4_-*Me*), 6.79 (s, 1H, CH), 7.10 (d, *J* = 8.2 Hz, 2H, C_6_H_4_), 7.49 (d, *J* = 8.4 Hz, C_6_H_5_), 10.06 (s, 1H, NH). ^13^C-NMR (101 MHz, DMSO-d_6_) δ [ppm] = 18.5 (Me), 20.3 (C_6_H_4_-*Me*), 105.3 (CH), 117.0 (C_6_H_4_), 129.3 (C_6_H_4_), 130.1 (C_6_H_4_), 139.2 (C_6_H_4_), 148.3 (C4), 164.6 (CSe). ^1^H-^77^Se-HMBC (400 MHz, 76 MHz, DMSO-d_6_) δ [ppm] = 584.0. High-resolution MS: calculated for [C_11_H_13_N_2_Se]^+^ 253.0244; found 253.0261. Elemental analysis for C_11_H_12_N_2_Se calculated: H 4.82, C 52.60, N 11.15; found H 4.76, C 52.42, N 11.06.

### 3.22. 2-Amino-(4-tolyl)-1,3-selenazole ***18***

This was prepared as described above using 4-MeC_6_H_4_NHC(Se)NH_2_ (0.202 g, 1.02 mmol) and chloroacetaldehyde (0.079 g, 1.00 mmol). The product was obtained as a colorless solid in 60% yield (0.146 g). ^1^H-NMR (400 MHz, DMSO-d_6_) δ [ppm] = 2.24 (s, 3H, Me), 7.10 (d, *J* = 8.7 Hz, 2H, C_6_H_4_), 7.16 (d, *J* = 4.2 Hz, 1H, NCH), 7.29 (d, *J* = 4.2 Hz, 1H, CH), 7.52 (d, *J* = 8.5 Hz, C_6_H_4_), 10.16 (s, 1H, NH). ^13^C-NMR (101 MHz, DMSO-d_6_) δ [ppm] = 20.3 (Me), 112.8 (CH), 117.3 (C_6_H_2_), 129.3 (C_6_H_4_), 130.1 (C_6_H_4_), 139.5 (NCH), 138.9 (C_6_H_4_), 166.0 (CSe). ^1^H-^77^Se-HMBC (400 MHz, 76 MHz, DMSO-d_6_) δ [ppm] = 568.6. High-resolution MS: calculated for [C_10_H_11_N_2_Se]^+^ 239.0088; found 239.0100. Elemental analysis for C_10_H_10_N_2_Se calculated: H 4.25, C 50.64, N 11.81; found H 4.61, C 50.43, N 11.45.

### 3.23. 2-Amino-(4-methoxyphenyl)-4-phenyl-1,3-selenazole ***19***

This was prepared as described above using 4-MeOC_6_H_4_NHC(Se)NH_2_ (0.180 g, 0.84 mmol) and 2-bromoacetophenone (0.172 g, 0.86 mmol). The product was obtained as a beige solid in 58% yield (0.165 g). ^1^H-NMR (400 MHz, DMSO-d_6_) δ [ppm] = 3.74 (s, 3H, OMe), 6.95 (d, *J* = 9.1 Hz, 2H, C_6_H_4_), 7.29 (t, *J* = 7.3 Hz, 1H, *p*-Ph), 7.39 (t, *J* = 7.6 Hz, 2H, *m*-Ph), 7.68 (m, 3H, CH, C_6_H_4_), 7.91 (d, *J* = 9.9 Hz, 2H, *o*-Ph), 10.17 (s, 1H, NH). ^13^C-NMR (101 MHz, DMSO-d_6_) δ [ppm] = 55.2 (OMe), 106.9 (CH), 114.3 (C_6_H_4_), 118.7 (C_6_H_4_), 125.9 (*o*-Ph), 127.2 (*p*-Ph), 128.5 (*m*-Ph), 134.9 (C_6_H_4_), 135.5 (*ipso*-Ph), 150.9 (C4), 154.2 (C_6_H_4_), 164.7 (CSe). ^1^H-^77^Se-HMBC (400 MHz, 76 MHz, DMSO-d_6_) δ [ppm] = 618.5. High-resolution MS: calculated for [C_16_H_15_N_2_OSe]^+^ 331.0350; found 331.0346. Elemental analysis for C_16_H_14_N_2_OSe calculated: H 4.29, C 58.37, N 8.51; found: H 4.26, C 58.55, N 8.45.

### 3.24. 2-Amino-(4-methoxyphenyl)-4-(4-tolyl)-1,3-selenazole ***20***

This was prepared as described above using 4-MeOC_6_H_4_NHC(Se)NH_2_ (0.200 g, 0.93 mmol) and 2-bromo-4**′**-methylacetophenone (0.195 g, 0.91 mmol). The product was obtained as a colorless solid in 58% yield (0.182 g). ^1^H-NMR (400 MHz, CDCl_3_) δ [ppm] = 2.37 (s, 3H, Me), 3.83 (s, 3H, OMe), 6.90 (d, *J* = 9.0 Hz, 2H, MeOC_6_H_4_), 7.17 (d, *J* = 7.9 Hz, 2H, MeC_6_H_4_), 7.22 (s, 1H, CH), 7.29 (d, *J* = 9.0 Hz, 2H, MeOC_6_H_4_), 7.72 (d, *J* = 8.1 Hz, 2H, MeC_6_H_4_). ^13^C-NMR (101 MHz, CDCl_3_) δ [ppm] = 21.2 (Me), 55.5 (OMe), 104.5 (CH), 114.8 (MeOC_6_H_4_), 122.9 (MeOC_6_H_4_), 126.3 (MeC_6_H_4_), 129.2 (MeC_6_H_4_), 132.8 (MeC_6_H_4_), 134.4 (MeOC_6_H_4_), 137.5 (MeC_6_H_4_), 152.2 (C4), 156.9 (MeOC_6_H_4_), 169.8 (CSe). ^1^H-^77^Se-HMBC (400 MHz, 76 MHz, DMSO-d_6_) δ [ppm] = 586.8. High-resolution MS: calculated for [C_17_H_17_N_2_OSe]^+^ 345.0506; found 345.0534. Elemental analysis for C_17_H_16_N_2_OSe calculated: H 4.70, C 59.48, N 8.16; found H 4.66, C 59.51, N 8.08.

### 3.25. 2-Amino-(4-methoxyphenyl)-4-(4-methoxyphenyl)-1,3-selenazole ***21***

This was prepared as described above using 4-MeOC_6_H_4_NHC(Se)NH_2_ (0.212 g, 0.99 mmol) and 2-bromo-4**′**-methoxyacetophenone (0.226 g, 0.99 mmol). The product was obtained as a beige solid in 65% yield (0.231 g). ^1^H-NMR (400 MHz, DMSO-d_6_) δ [ppm] = 3.74 (s, 3H, OMe_B_), 3.78 (s, 3H, OMe_A_), 6.95 (m, 4H, C_6_H_4_-A, C_6_H_4_-B), 7.52 (s, 1H, CH), 7.65 (d, *J* = 9.0 Hz, 2H, C_6_H_4_-B), 7.83 (d, *J* = 8.8 Hz, 2H, C_6_H_4_-A), 10.12 (s, 1H, NH). ^13^C-NMR (101 MHz, DMSO-d_6_) δ [ppm] = 55.1 (OMe_B_), 55.2 (OMe_A_), 104.6 (CH), 113.9 (C_6_H_4_), 114.2 (C_6_H_4_), 118.7 (C_6_H_4_-B), 127.2 (C_6_H_4_-A), 128.4 (C_6_H_4_-A), 134.9 (C_6_H_4_-B), 150.7 (C4), 154.2 (C_6_H_4_-B), 158.5 (C_6_H_4_-A), 164.5 (CSe). ^1^H-^77^Se-HMBC (400 MHz, 76 MHz, DMSO-d_6_) δ [ppm] = 603.0. High-resolution MS: calculated for [C_17_H_17_N_2_O_2_Se]^+^ 361.0455; found 361.0484. Elemental analysis for C_17_H_16_N_2_O_2_Se calculated: H 4.49, C 56.83, N 7.80; found H 4.51, C 56.87, N 7.74.

### 3.26. 2-Amino-(4-methoxyphenyl)-4-(4-biphenyl)-1,3-selenazole ***22***

This was prepared as described above using 4-MeOC_6_H_4_NHC(Se)NH_2_ (0.203 g, 0.95 mmol) and 2-bromo-4**′**-phenylacetophenone (0.291 g, 1.06 mmol). The product was obtained as a beige solid in 68% yield (0.260 g). ^1^H-NMR (400 MHz, DMSO-d_6_) δ [ppm] = 3.75 (s, 3H, OMe), 6.96 (d, *J* = 9.1 Hz, 2H, MeOC_6_H_4_), 7.37 (t, *J* = 7.3 Hz, 1H, *p*-Ph), 7.48 (t, *J* = 7.6 Hz, 2H, *m*-Ph), 7.69 (m, 6H, MeOC_6_H_4_, C_6_H_4_, *o*-Ph), 7.77 (s, 1H, CH), 7.99 (d, *J* = 8.0 Hz, 2H, C_6_H_4_), 10.18 (s, 1H, NH). ^13^C-NMR (101 MHz, DMSO-d_6_) δ [ppm] = 55.2 (OMe), 107.2 (CH), 114.3 (MeOC_6_H_4_), 118.7 (MeOC_6_H_4_), 126.4 (C_6_H_4_), 126.8 (*o-*Ph), 127.4 (p-Ph), 128.9 (*m*-Ph), 134.6 (MeOC_6_H_4_), 134.9 (C_6_H_4_) 138.7 (C_6_H_4_), 139.7 (*ipso*-Ph), 150.5 (C4), 154.2 (MeOC_6_H_4_), 164.7 (CSe). ^1^H-^77^Se-HMBC (400 MHz, 76 MHz, DMSO-d_6_) δ [ppm] = 609.3. High-resolution MS: calculated for [C_22_H_19_N_2_OSe]^+^ 407.0663; found 407.0717. Elemental analysis for C_22_H_18_N_2_OSe calculated: H 4.48, C 65.19, N 6.91; found: H 4.45, C 65.62, N 6.86.

### 3.27. 2-Amino-(4-methoxyphenyl)-4-(2-naphtyl)-1,3-selenazole ***23***

This was prepared as described above using 4-MeOC_6_H_4_NHC(Se)NH_2_ (0.207 g, 0.96 mmol) and 2-bromo-2**′**-acetonaphthone (0.239 g, 0.96 mmol). The product was obtained as a beige solid in 68% yield (0.248 g). ^1^H-NMR (400 MHz, DMSO-d_6_) δ [ppm] = 3.77 (s, 3H, OMe), 7.00 (d, J = 9.0 Hz, 2H, MeOC_6_H_4_), 7.51 (m, 2H, naph), 7.71 (d, *J* = 9.0 Hz, 2H, MeOC_6_H_4_), 7.87 (s, 1H, CH), 7.99 (m, 4H, naph), 8.45 (s, 1H, naph), 10.22 (s, 1H, NH). ^13^C-NMR (101 MHz, DMSO-d_6_) δ [ppm] = 55.2 (OMe), 107.8 (CH), 114.4 (MeOC_6_H_4_), 118.9 (MeOC_6_H_4_), 124.3 (naph), 124.5 (naph), 125.8 (naph), 126.3 (naph), 127.5 (naph), 128.0 (naph), 128.1 (naph), 132.3 (naph), 132.9 (naph), 133.2 (naph), 134.9 (MeOC_6_H_4_), 150.9 (C4), 154.3 (MeOC_6_H_4_), 164.8 (CSe). ^1^H-^77^Se-HMBC (400 MHz, 76 MHz, DMSO-d_6_) δ [ppm] = 609.2. High-resolution MS: calculated for [C_20_H_17_N_2_OSe]^+^ 381.0506; found 381.0532. Elemental analysis for C_20_H_16_N_2_OSe calculated.: H 4.25, C 63.33, N 7.39; found: H 4.27, C 63.41, N 7.29.

### 3.28. 2-Amino-(4-methoxyphenyl)-4-(tert-butyl)-1,3-selenazole ***24***

This was prepared as described above using 4-MeOC_6_H_4_NHC(Se)NH_2_ (0.221 g, 1.03 mmol) and 1-bromopinacolone (0.179 g, 1.00 mmol). The product was obtained as an orange solid in 35% yield (0.108 g). ^1^H-NMR (400 MHz, DMSO-d_6_) δ [ppm] = 1.26 (s, 9H, *^t^*Bu), 3.72 (s, 3H, OMe), 6.76 (s, 1H, CH), 6.90 (d, *J* = 9.0 Hz, 2H, C_6_H_4_), 7.57 (d, *J* = 9.0 Hz, 2H, C_6_H_4_), 9.95 (s, 1H, NH). ^13^C-NMR (101 MHz, DMSO-d_6_) δ [ppm] = 29.6 (*^t^*Bu), 35.1 (*^t^*Bu), 55.1 (OMe), 102.3 (CH), 114.1 (C_6_H_4_), 118.5 (C_6_H_4_), 135.2 (C_6_H_4_), 154.0 (C_6_H_4_), 162.0 (C4), 164.2 (CSe). ^1^H-^77^Se-HMBC (400 MHz, 76 MHz, DMSO-d_6_) δ [ppm] = 576.5. High-resolution MS: calculated for [C_14_H_19_N_2_OSe]^+^ 311.0663; found 311.0694. Elemental analysis for C_14_H_18_N_2_OSe calculated: H 5.87, C 54.37, N 9.06; found H 5.78, C 53.65, N 8.81.

### 3.29. 2-Amino-(4-methoxyphenyl)-4-methyl-1,3-selenazole ***25***

This was prepared as described above using 4-MeOC_6_H_4_NHC(Se)NH_2_ (0.225 g, 1.05 mmol) and chloroacetone (0.093 g, 1.00 mmol). The product was obtained as a colorless solid in 79% yield (0.212 g). ^1^H-NMR (400 MHz, DMSO-d_6_) δ [ppm] = 2.16 (d, *J* = 1.1 Hz, 3H, Me), 3.72 (s, 3H, OMe), 6.75 (d, *J* = 1.1 Hz, 1H, CH), 6.90 (d, *J* = 9.1 Hz, 2H, C_6_H_4_), 7.53 (d, *J* = 9.1 Hz, 2H, C_6_H_4_), 9.94 (s, 1H, NH). ^13^C-NMR (101 MHz, DMSO-d_6_) δ [ppm] = 18.4 (Me), 55.2 (OMe), 105.0 (CH), 114.1 (C_6_H_4_), 118.8 (C_6_H_4_), 135.1 (C_6_H_4_), 148.2 (C4), 154.1 (C_6_H_4_), 165.0 (CSe). ^1^H-^77^Se-HMBC (400 MHz, 76 MHz, DMSO-d_6_) δ [ppm] = 577.2. High-resolution MS: calculated for [C_11_H_13_N_2_OSe]^+^ 269.0193; found 269.0206. Elemental analysis for C_11_H_13_N_2_OSe calculated: H 4.53, C 49.45, N 10.48; found: H 4.58, C 49.59, N 10.48.

### 3.30. 2-Amino-(4-methoxyphenyl)-1,3-selenazole ***26***

This was prepared as described above using 4-MeOC_6_H_4_NHC(Se)NH_2_ (0.221 g, 1.03 mmol) and chloroacetaldehyde (0.079 g, 1.00 mmol). The product was obtained as a colorless solid in 34% yield (0.085 g). ^1^H-NMR (400 MHz, DMSO-d_6_) δ [ppm] = 3.73 (s, 3H, OMe), 6.90 (d, *J* = 9.1 Hz, 2H, C_6_H_4_), 7.13 (d, *J* = 4.17 Hz, 1H, NCH), 7.25 (d, *J* = 4.2 Hz, 1H, CH), 7.55 (d, *J* = 9.1 Hz, 2H, C_6_H_4_), 10.07 (s, 1H, NH). ^13^C-NMR (101 MHz, DMSO-d_6_) δ [ppm] = 55.2 (OMe), 112.0 (CH), 114.1 (C_6_H_4_), 118.7 (C_6_H_4_), 135.0 (C_6_H_4_), 139.4 (NCH), 154.1 (C_6_H_4_), 166.7 (CSe). ^1^H-^77^Se-HMBC (400 MHz, 76 MHz, DMSO-d_6_) δ [ppm] = 562.5. High-resolution MS: calculated for [C_10_H_11_N_2_OSe]^+^ 255.0037; found 255.0003. Elemental analysis for C_10_H_10_N_2_OSe calculated: H 3.98, C 47.44, N 11.07; found H 4.07, C 47.53, N 10.90.

### 3.31. Acetylation Reactions

The 2-amino-1,3-selenazol was heated in acetic anhydride (5 mL) at 100 °C for ca. 5 min. The solution was treated with water to precipitate the product. The solid was isolated by filtration and was subsequently recrystallized from EtOH.

### 3.32. 2-Acetamidophenyl-4-phenyl-1,3-selenazole ***27***

This was prepared as described above using 2-aminophenyl-4-phenyl-1,3-selenazole (0.121 g, 0.400 mmol). A colorless product was obtained in 80% yield (0.110 g). ^1^H-NMR (400 MHz, DMSO-d_6_) δ = 2.37 (s, 3 H, Me), 7.21 (t, *J* = 7.3 Hz, 1 H, *p*-Ph_A_), 7.28 (t, *J* = 7.4 Hz, 2 H, *m*-Ph_A_), 7.50–7.65 (m, 7 H, *o*-Ph_A_, Ph_B_), 8.17 (s, ^2^*J*_H-Se_ = 43 Hz, 1 H, CH). ^13^C-NMR (101 MHz, DMSO-d_6_) δ = 23.6 (Me), 114.6 (CH), 125.7 (Ph_B_), 127.3 (*p*-Ph_A_), 128.4 (*m-*Ph_A_), 128.8 (Ph_B_), 128.9 (*p-*Ph_B_), 129.6 (*o*-Ph_A_), 135.2 (*ipso*-Ph_A_), 140.2 (*ipso*-Ph_B_), 148.6 (C4), 160.7 (CSe), 170.0 (CO). ^1^H-^77^Se-HMBC (400 MHz, 76 MHz, DMSO-d_6_) δ = 694.2 (s). High-resolution MS: calculated for [C_17_H_15_N_2_OSeNa]^+^ 365.0169; found 365.0107. Elemental analysis for C_17_H_15_N_2_OSe calculated: H 4.14, C 59.83, N 8.21; found H 4.12, C 59.96, N 8.17.

### 3.33. 2-Acetamido-(4-tolyl)-4-methyl-1,3-selenazole ***28***

This was prepared as described above using 2-amino-(4-tolyl)-4-methyl-1,3-selenazol (0.019 g, 0.70 mmol). A colorless product was obtained in 45% yield (0.099 g). ^1^H-NMR (400 MHz, MeOH-d_4_) δ = 2.03 (s, 3 H, Me), 2.17 (d, *J* = 1.1 Hz, 3 H, Me), 2.47 (s, 3 H, Me), 7.21 (m, 2 H, C_6_H_4_), 7.24 (s, 1 H, CH), 7.40 (m, 2 H, C_6_H_4_). ^13^C-NMR (101 MHz, MeOH-d_4_) δ = 18.4 (Me), 21.2 (Me), 23.8 (Me), 114.6 (CH), 129.7 (C_6_H_4_), 131.6 (C_6_H_4_), 139.2 (C_6_H_4_), 140.6 (C_6_H_4_), 148.5 (C4), 163.2 (CSe), 172.1 (CO). ^1^H-^77^Se-HMBC (400 MHz, 76 MHz, MeOH-d_4_) δ = 666.6 (s). High-resolution MS: calculated for [C_13_H_14_N_2_OSeNa]^+^ 317.0169; found 317.0184. Elemental analysis for C_13_H_14_N_2_OSe calculated: H 4.81, C 53.25, N 9.55; found H 4.88, C 52.67, N 9.41.

### 3.34. 2-Acetamido-(4-methoxyphenyl)-4-(t-butyl)-1,3-selenazole ***29***

This was prepared as described above using 2-amino-(4-methoxyphenyl)-4-(t-butyl)-1,3-selenazol (0.065 g, 0.20 mmol). An orange product was obtained in 85% yield (0.063 g). ^1^H-NMR (400 MHz, DMSO-d_6_) δ = 1.05 (s, 9 H, ^t^Bu), 1.99 (s, 3 H, Me), 3.80 (s, 3 H, OMe), 7.07 (d, *J* = 8.9 Hz, 2 H, C_6_H_4_), 7.24 (s, ^2^*J*_H-Se_ = 45 Hz, 1 H, CH), 7.31 (d, *J* = 8.9 Hz, 2 H, C_6_H_4_). ^13^C-NMR (101 MHz, DMSO-d_6_) δ = 23.6 (Me), 29.5 (^t^Bu), 34.9 (^t^Bu), 55.3 (OMe), 110.1 (CH), 114.4 (C_6_H_4_), 129.8 (C_6_H_4_), 133.1 (C_6_H_4_), 158.9 (C_6_H_4_), 159.8 (C4), 159.9 (NC) 169.9 (CO). ^1^H-^77^Se-HMBC (400 MHz, 76 MHz, DMSO-d_6_) δ = 664.5 (s). High-resolution MS: calculated for [C_16_H_20_N_2_O_2_SeNa]^+^ 375.0588; found 375.0480. Elemental analysis for C_16_H_20_N_2_O_2_Se calculated: H 5.74, C 54.70, N 7.97; found H 5.69, C 54.88, N 7.97.

### 3.35. Halogenation Reactions

To a solution of the *N*-acetyl selenazole in CH_2_Cl_2_ (5 mL) was added NBS or NIS (1 equiv.). After stirring for 1 h at room temperature, the solvent was removed and the remaining solid was dissolved in EtOH. Addition of water precipitated the product, which was isolated by filtration and was subsequently recrystallized from EtOH. The chloro-compound was prepared similarly using PhICl_2_ and Et_2_O as solvent.

### 3.36. 2-Acetamidophenyl-5-chloro-4-phenyl-1,3-selenazole ***30***

This was prepared as described above from 2-acetamidophenyl-4-phenyl-1,3-selenazole (0.031 g, 0.09 mmol) and PhICl_2_ (0.031 g, 0.11 mmol) in Et_2_O. A colorless product was obtained in 59% yield (0.025 g). ^1^H-NMR (600 MHz, DMSO-d_6_) δ = 2.05 (s, 3 H, Me), 7.27–7.32 (m, 1 H, *p*-Ph_A_), 7.32–7.37 (m, 2 H, *m-*Ph_A_), 7.50–7.55 (m, 3 H, *p*-Ph_B_, Ph), 7.52–7.60 (m, 4 H, Ph). ^13^C-NMR (151 MHz, DMSO-d_6_) δ = 23.3 (Me), 119.7 (CCl), 127.8 (Ph), 127.9 (*p*-Ph_A_), 128.2 (*m*-Ph_A_), 128.9 (Ph), 129.2 (*p*-Ph_B_), 129.7 (Ph), 133.4 (*ipso*-Ph_A_), 138.8 (*ipso-*Ph_B_), 144.3 (C4), 156.5 (CSe), 170.8 (CO). ^77^Se-NMR (114 MHz, DMSO-d_6_) δ = 720.9 (s). High-resolution MS: calculated for [C_17_H_13_N_2_ClOSeNa]^+^ 398.9779; found 398.9637. Elemental analysis for C_17_H_13_N_2_ClOSe calculated: H 3.49, C 54.35, N 7.46; found H 3.68, C 54.35, N 7.22.

### 3.37. 2-Acetamidophenyl-5-bromo-4-phenyl-1,3-selenazole ***31***

This was prepared as described above from 2-acetamidophenyl-4-phenyl-1,3-selenazole (0.043 g, 0.12 mmol) and NBS (0.027 g, 0.10 mmol). A colorless product was obtained in 62% yield (0.033 g). ^1^H-NMR (600 MHz, CDCl_3_) δ = 2.13 (s, 3 H, Me), 7.28–7.30 (m, 1 H, *p*-Ph_A_), 7.30–7.35 (m, 4 H, *o*-Ph_B_, m-Ph_A_), 7.50–7.53 (m, 1 H, *p*-Ph_B_), 7.54–7.58 (m, 2 H, *m*-Ph_B_), 7.74–7.76 (m, 2 H, *o*-Ph_A_). ^13^C-NMR (151 MHz, CDCl_3_) δ = 23.5 (Me), 105.0 (CBr), 127.7 (p-Ph_A_), 127.9 (*m*-Ph_A_), 128.6 (*o*-Ph_A/B_) 128.7 (*o*-Ph_A/B_), 129.3 (p-Ph_B_), 129.9 (*m*-Ph_B_), 134.8 (ipso-Ph_A_), 139.4 (*ipso*-Ph_B_), 147.2 (C4), 159.4 (CSe), 170.6 (CO). ^77^Se-NMR (114 MHz, DMSO-d_6_) δ = 737.5 (s). High-resolution MS: calculated for [C_17_H_13_N_2_BrOSeNa]^+^ 442.9274; found 442.9142. Elemental analysis for C_17_H_13_N_2_BrOSe calculated: H 3.12, C 48.60, N 6.67; found H 3.09, C 48.74, N 6.66.

### 3.38. 2-Acetamidophenyl-5-iodo-4-phenyl-1,3-selenazole ***32***

This was prepared as described above from 2-acetamidophenyl-4-phenyl-1,3-selenazole (0.070 g, 0.21 mmol) and NIS (0.051 g, 0.22 mmol). A colorless product was obtained in 87% yield (0.091 g). ^1^H-NMR (600 MHz, DMSO-d_6_) δ [ppm] = 2.03 (s, 3H, Me), 7.31 (m, 3H, *p*-Ph_A_, *m*-Ph_A_), 7.55 (m, 7H, Ph). ^13^C-NMR (151 MHz, DMSO-d_6_) δ [ppm] = 23.3 (Me), 127.8 (Ph), 127.9 (Ph), 128.8 (Ph), 129.7 (Ph), 135.7 (*ipso*-Ph_A_), 139.5 (*ipso*-Ph_B_), 151.3 (C4), 164.4 (CSe), 170.6 (CO). ^77^Se-NMR (114 MHz, DMSO-d_6_) δ [ppm] = 776.2. High-resolution MS: calculated for [C_17_H_13_N_2_IOSeNa]^+^ 490.9136; found 490.9042. Elemental analysis for C_17_H_13_N_2_IOSe calculated: H 2.80, C 43.71, N 6.00; found H 2.76, C 43.67, N 5.93.

### 3.39. 2-Acetamido-(4-tolyl)-5-bromo-4-methyl-1,3-selenazole ***33***

This was prepared as described above from 2-acetamidophenyl-4-methyl-1,3-selenazole (0.019 g, 0.06 mmol) and NBS (0.025 g, 0.14 mmol). A beige product was obtained in 58% yield (0.013 g). ^1^H-NMR (400 MHz, DMSO-d_6_) δ [ppm] = 1.97 (s, 3H, CO*Me*, 2.06 (s, 3H, Me), 2.37 (s, 3H, C_6_H_4_-*Me*), 7.33 (m, 4H, C_6_H_4_). ^13^C-NMR (101 MHz, DMSO-d_6_) δ [ppm] = 16.4 (Me), 20.7 (C_6_H_4_-*Me*), 23.3 (CO*Me*), 103.3 (CBr), 128.6 (C_6_H_4_), 130.3 (C_6_H_4_), 136.6 (C_6_H_4_), 138.6 (C_6_H_4_), 146.0 (C4), 159.4 (CSe), 170.7 (CO). ^77^Se-NMR (76 MHz, DMSO-d_6_) δ [ppm] = 704.9. High-resolution MS: calculated for [C_13_H_13_N_2_BrOSeNa]^+^ 394.9274; found 3949288. Elemental analysis for C_13_H_13_N_2_BrOSe calculated: H 3.52, C 41.96, N 7.53; found H 3.56, C 42.32, N 7.44.

### 3.40. Hydroxylamine Derivatives

The 2-amino-1,3-selenazol was dissolved in glacial acetic acid and treated with sodium nitrite (1.5 equiv.). The reaction mixture was stirred for 10 min at room temperature. Addition of water precipitated the product, which was isolated by filtration and subsequently recrystallized from EtOH.

### 3.41. 2-Aminophenyl-5-hydroxylamine-4-phenyl-1,3-selenazole ***34***

This was prepared as described above from 2-aminophenyl-4-phenyl-1,3-selenazole (0.054 g, 0.18 mmol) and NaNO_2_ (0.019 g, 0.28 mmol). A yellow product was obtained in 86% yield (0.051 g). ^1^H-NMR (400 MHz, DMSO-d_6_) δ [ppm] = 7.08 (d, *J* = 7.3 Hz, 2H, *o*-Ph_B_), 7.28 (t, *J* = 7.5 Hz, 1H, *p*-Ph_B_), 7.48 (m, 2H, *m*-Ph_B_), 7.59 (t, *J* = 7.6 Hz, 2H, *m*-Ph_A_), 7.69 (t, *J* = 7.4 Hz, 1H, *p*-Ph_A_), 8.22 (d, *J* = 7.1 Hz, 2H, *o*-Ph_A_), 13.74 (s, 1H, OH). ^13^C-NMR (101 MHz, DMSO-d_6_) δ [ppm] = 119.5 (*o*-Ph_B_), 125.9 (*p*-Ph_B_), 128.6 (*m*-Ph_A_), 129.6 (*m*-Ph_B_), 130.7 (*o*-Ph_A_) 132.8 (*p*-Ph_A_), 150.9 (*ipso*-Ph_B_), 154.1 (C5), 174.6 (C4). ^77^Se-NMR (76 MHz, DMSO-d_6_) δ [ppm] = 407.0. High-resolution MS: calculated for [C_15_H_11_N_3_OSeNa]^+^ 351.9965; found 351.9899. Elemental analysis for C_15_H_11_N_3_OSe calculated: H 3.38, C 54.89, N 12.80; found H 3.86, C 54.46, N 12.46.

### 3.42. 2-Amino-(4-methoxyphenyl)-5-hydroxylamine-4-(4-methoxyphenyl)-1,3-selenazole **35**

This was prepared as described above from 2-amino-(4-methoxyphenyl)-4-(4-methoxyphenyl)-1,3-selenazole (0.045 g, 0.13 mmol) and NaNO_2_ (0.067 g, 0.98 mmol). A red product was obtained in 67% yield (0.033 g). ^1^H-NMR (600 MHz, DMSO-d_6_) δ [ppm] = 3.80 (s, 3H, OMe_B_), 3.89 (s, 3H, OMe_A_), 7.05 (m, 4H, C_6_H_4_-B), 7.15 (d, *J* = 8.99 Hz, 2H, C_6_H_4_-A), 8.33 (d, *J* = 8.98 Hz, 2H, C_6_H_4_-A), 13.62 (s, 1H, OH). ^13^C-NMR (151 MHz, DMSO-d_6_) δ [ppm] = 55.3 (OMe), 55.6 (OMe), 114.2 (C_6_H_4_-A), 114.7 (C_6_H_4_-B), 121.2 (C_6_H_4_-B), 123.6 (C_6_H_4_-A) 132.9 (C_6_H_4_-A),143.9 (C_6_H_4_-B) 154.6 (C5), 157.3 (C_6_H_4_-B), 163.1 (C_6_H_4_-A), 172.5 (C4). ^77^Se-NMR (114 MHz, DMSO-d_6_) δ [ppm] = 408.8. High-resolution MS: calculated for [C_17_H_14_N_3_O_3_Se]^-^ 388.0200; found 388.0234. Elemental analysis for C_17_H_15_N_3_O_3_Se calculated: H 3.89, C 52.59, N 10.82; found H 3.86, C 52.54, N 10.75.

### 3.43. Sonogashira Coupling Reactions

#### 3.43.1. 2-Acetamidophenyl-4-phenyl-5-phenylethinyl-1,3-selenazole **36**

Under a dinitrogen atmosphere a mixture of 2-acetamidophenyl-5-iodo-4-phenyl-1,3-selenazole (0.065 g, 0.14 mmol), CuI (0.010 g, 0.05 mmol), PdCl_2_(PPh_3_)_2_ (0.006 g, 0.01 mmol) and phenylacetylene (0.040 g, 0.39 mmol) in triethylamine was stirred for three days at room temperature. The solution was treated with water and CH_2_Cl_2_. The organic phase was separated, dried over MgSO_4_ and the filtrate was passed through silica gel. The crude product was recrystallized from EtOH. A beige product was obtained in 86% yield (0.053 g). ^1^H-NMR (600 MHz, DMSO-d_6_) δ [ppm] = 2.06 (s, 3H, Me), 7.31 (t, *J* = 7.3 Hz, *p*-Ph_A_), 7.38 (t, *J* = 7.6 Hz, *m*-Ph_A_), 7.44 (m, 3H, *p*-Ph_B_, *m*-Ph_B_), 7.58 (m, 7H, Ph_c_, *o*-Ph_B_), 7.90 (d, *J* = 7.3 Hz, *o*-Ph_A_). ^13^C-NMR (151 MHz, DMSO-d_6_) δ [ppm] = 23.4 (Me), 83.4 (alkyne), 98.9 (alkyne), 110.7 (C5), 122.4 (*ipso*-Ph_B_), 127.2 (*o*-Ph_A_), 128.2 (*p*-Ph_A_), 128.3 (*m*-Ph_A_), 128.8 (Ph_C_, Ph_B_), 129.2 (Ph_C_) 129.7 (Ph_C_), 130.9 (*o*-Ph_B_),134.9 (*ipso*-Ph_A_), 139.5 (*ipso*-Ph_C_), 150.9 (C4), 158.7 (CSe), 170.8 (CO). ^77^Se-NMR (76 MHz, DMSO-d_6_) δ [ppm] = 752.6. High-resolution MS: calculated for [C_25_H_18_N_2_OSeNa]^+^ 465.0482; found 465.0371. Elemental analysis for C_25_H_18_N_2_OSe calculated: H 4.11, C 68.03, N 6.35; found H 4.04, C 68.05, N 6.38.

#### 3.43.2. 2-Acetamidophenyl-4-phenyl-5-(trimethylsilyl)ethinyl-1,3-selenazole **37**

This was prepared as described above out 2-acetamidophenyl-5-bromo-4-phenyl-1,3-selenazole (0.1010 g, 0.240 mmol), CuI (0.0074 g, 0.039 mmol), PdCl_2_(PPh_3_)_2_ (0.0194 g, 0.042 mmol) and trimethylsilylacetylene (0.1 mL, 0.702 mmol) in 6 mL triethylamine. The crude product was purified by column chromatography (silica gel, 3:7, acetone/hexane) affording 0.0931 g (88%) beige product. ^1^H-NMR (400 MHz, DMSO) δ [ppm] = 0.25 (s, 9H, TMS), 2.05 (s, 3H, Me_Ac_), 7.31 (m, 3H, Ph_A_), 7.58 (m, 5H, Ph_B_), 7.86 (m, 2H, Ph_A_). ^13^C-NMR (101 MHz, DMSO) δ [ppm] = 0.4 (TMS), 23.3 (Me_Ac_), 98.8 (alkyne), 105.6 (alkyne), 110.6 (C5), 127.1 (Ph_A_), 128.1 (Ph_A_), 128.3 (*p*-Ph_A_), 128.8 (Ph_B_), 129.2 (*p*-Ph_B_), 129.7 (Ph_B_), 134.7 (*ipso*-Ph_A_), 139.5 (*ipso*-Ph_B_), 151.8 (C4), 158.4 (CSe), 170.9 (CO). ^77^Se-NMR (76 MHz, DMSO) δ [ppm] = 755.1. High-resolution MS: calculated for [C_22_H_22_N_2_OSeSiNa]^+^ 461.0564; found 461.0570. Elemental analysis for C_22_H_22_N_2_OSeSi calculated: H 5.07, C 60.40, N 6.40; found H 4.95, C 59.20, N 6.28.

### 3.44. Metallation Reactions

#### 3.44.1. Acetoxy(2-acetamidophenyl-4-phenyl-1,3-selenazole-5yl)mercury **38**

2-Acetamidophenyl-4-phenyl-1,3-selenazole (0.0506 g, 0.12 mmol) was dissolved in a mixture of 1:1 EtOH/glacial acetic acid. A solution of mercury(II) acetate (0.0404 g, 0.13 mmol) in water/EtOH was added and the mixture stirred for 10 min at room temperature. Addition of water precipitated the colorless product (0.0521 g, 0.09 mmol, 73%), which was isolated by filtration and dried in air. ^1^H-NMR (400 MHz, DMSO) δ [ppm] = 1.94 (s, 3H, Me_OAc_), 2.02 (s, 3H, Me_Ac_), 7.24 (m, 3H, *m*-Ph_A_, *p*-Ph_A_), 7.55 (m, 5H, Ph_B_), 7.73 (dd, 2H, *J* = 7.9 Hz, *J* = 1.6 Hz, *o*-Ph_A_). ^13^C-NMR (101 MHz, DMSO) δ [ppm] = 22.8 (Me_OAc_), 23.7 (Me_Ac_), 126.7 (*o*-Ph_A_), 127.1 (*p*-Ph_A_), 128.2 (*m*-Ph_A_), 128.7 (*p*-Ph_B_), 128.8 (Ph_B_), 129.6 (Ph_B_), 138.5 (*ipso*-Ph_A_), 140.5 (*ipso*-Ph_B_), 153.2 (C4), 169.8 (CO_Ac_), 174.6 (CO_OAc_). No CHg signal was observed. ^77^Se-NMR (76 MHz, DMSO) δ [ppm] = 766.6. Elemental analysis for C_19_H_16_N_2_O_3_SeHg calculated: H 2.69, C 38.04, N 4.67; found H 2.60, C 37.75, N 4.60. Crystals suitable for X-ray diffraction were obtained by slow evaporation of an acetone solution of the compound.

#### 3.44.2. Acetoxy(2-acetamido-(4-tolyl)-4-phenyl-1,3-thiazole-5yl)mercury **38S**

This was synthesized by a method described in literature [24]. ^1^H-NMR (400 MHz, CDCl_3_) δ [ppm] = 1.96 (s, 3H, Me_OAc_), 2.00 (s, 3H, Me_Ac_), 2.36 (s, 3H, Me 4-tol), 7.10 (d, *J* = 7.1 Hz, 2H, 4-tol), 7.58–7.47 (m, 5H, Ph), 7.65 (d, *J* = 7.6 Hz, 2H, 4-tol). ^13^C-NMR (101 MHz, CDCl_3_) δ [ppm] = 20.6 (Me 4-tol), 22.8 (Me_OAc_), 23.6 (Me_Ac_), 126.4 (4-tol), 128.1 (Ph), 128.9 (4-tol), 129.6 (Ph), 134.1 (4-tol), 136.7 (4-tol), 140.4 (Ph), 152.7 (C4), 163.2 (C2), 169.4 (CO_Ac_), 174.5 (CO_OAc_). No CHg signal was observed. Crystals suitable for X-ray diffraction were obtained by slow diffusion of hexanes into an acetone solution of the compound.

#### 3.44.3. Chlorido(2-acetamidophenyl-4-phenyl-1,3-selenazole-5yl)mercury **39**

2-Acetamidophenyl-4-phenyl-1,3-selenazole (0.1557 g, 0.263 mmol) was dissolved in a mixture of 1:1 EtOH/glacial acetic acid. A solution of mercury(II) acetate (0.087 g, 272 mmol) in water/EtOH was added and the mixture stirred for 10 min at room temperature. Solid LiCl (0,0441 g, 1.040 mmol) was added and the resulting colorless product (0.1197 g, 0.208 mmol, 79%) precipitated. The solid was isolated by filtration and dried in air. ^1^H-NMR (400 MHz, DMSO) δ [ppm] = 2.01 (s, 3H, Me_Ac_), 7.26 (m, 3H, *m-*Ph_A_, *p*-Ph_A_), 7.52 (m, 2H, Ph_B_), 7.65 (d, ^3^J_HH_ = 8.1 Hz, *o*-Ph_A_). ^13^C-NMR (101 MHz, DMSO) δ [ppm] = 23.7 (Me_Ac_), 126.7 (*o-*Ph_A_), 127.2 (*p-*Ph_A_), 128.3 (*m-*Ph_A_), 128.8 (*p-*Ph_B_), 128.8 (Ph_B_), 129.6 (Ph_B_), 139.2 (*ipso*-Ph_A_), 140.4 (*ipso*-Ph_B_), 153.2 (C4), 164.4 (C2), 169.8 (CO_Ac_). No CHg signal was observed. ^77^Se-NMR (76 MHz, DMSO) δ [ppm] = 768.2 Elemental analysis for C_17_H_13_N_2_OSeHgCl calculated: H 2.27, C 35.43, N 4.86; found H 2.18, C 35.79, N 4.84.

#### 3.44.4. 2,2′-Diacetamidophenyl-4,4′-diphenyl-5,5‘-bis(1,3-selenazole) **40**

Chlorido(2-acetamidophenyl-4-phenyl-1,3-selenazole-5yl)mercury (0.256 g, 0.44 mmol), [Me_4_N]Cl (0.034 g, 0.63 mmol) and [Me_4_N][AuCl_4_] (0.180 g, 0.09 mmol) were dissolved in acetone. The mixture was stirred for three days at room temperature. The solvent was allowed to evaporate without heating. The remaining solid was extracted with CH_2_Cl_2_ and filtered. Evaporation of the filtrate in vacuum afforded a solid, which was washed with diethyl ether, recrystallized from EtOH and dried. The pure colorless product was obtained in 7% (0.010 g, 0.02 mmol) yield. ^1^H-NMR (400 MHz, DMSO) δ [ppm] = 1.99 (s, 6H, Me), 7.22 (m, 6H, Ph), 7.38 (m, 4H, Ph), 7.55 (m, 10H, Ph). ^13^C-NMR (101 MHz, DMSO) δ [ppm] = 23.4 (Me), 127.6 (Ph), 128.1 (Ph), 128.3 (Ph), 128.9 (Ph), 129.6 (Ph), 135.0 (*ipso*-Ph), 139.9 (*ipso*-Ph), 146.2 (C4) 170.4 (CO). High-resolution MS: calculated for [C_34_H_26_N_4_O_2_Se_2_H]^+^ 683.0464 found 683.0498. Elemental analysis for C_34_H_26_N_4_O_2_Se_2_ calculated: H 3.85, C 60.01, N 8.23; found H 3.91, C 59.93, N 8.17. A few crystals of the gold(III) salt 39Au deposited when sample of the reaction mixture was left to slowly evaporate.

## 4. Conclusions

A variety of 2-amino-1,3-selenazoles was synthesized and characterized with various spectroscopic methods as well as X-ray diffraction. Furthermore, their reactivity was studied in detail. The reaction of a 2-amino-1,3-selenazole with NaNO_2_ gave a hydroxylamine, not a nitrosamine as claimed by Bulka in 1963. An efficient method to halogenate the 5-position of the selenazole is reported, and Sonogashira coupling of the iodo-derivatives was found to be possible. The 5-position can be readily mercurated with Hg(OAc)_2_ and the resulting organomercury reagents can be used to transfer a selenozolyl-group to gold(III). The species are reactive, readily undergoing reductive elimination affording a bis(selenazole). Furthermore, biological studies with some of the compounds were performed. No cytotoxic effects against a panel of 60 cancer cells was observed. Little activity against *S. aureus* but good activity against *C. albicans* and *C. neoformans* var. *grubii* was observed in some compounds. The most promising compounds are **5** and especially **9**, since they show relatively low toxicity and high activity against the fungal strains.

## Figures and Tables

**Table 1:** Prepared 2-amino-1,3-selenazole derivatives.

Compound	NHR	R‘	% Yield ^a^	Reference
**3**	Ph	Ph	89	[9]
**4**	Ph	4-MeC_6_H_4_	69	[9]
**5**	Ph	4-MeOC_6_H_4_	71	[9]
**6**	Ph	4-PhC_6_H_4_	74	This work
**7**	Ph	2-Nap	76	This work
**8**	Ph	^t^Bu	52	This work
**9**	Ph	Me	56	[5]
**10**	Ph	H	32	[9]
**11**	4-MeC_6_H_4_	Ph	40	[9]
**12**	4-MeC_6_H_4_	4-MeC_6_H_4_	58	[9]
**13**	4-MeC_6_H_4_	4-MeOC_6_H_4_	48	This work
**14**	4-MeC_6_H_4_	4-PhC_6_H_4_	58	This work
**15**	4-MeC_6_H_4_	2-Nap	61	This work
**16**	4-MeC_6_H_4_	^t^Bu	84	This work
**17**	4-MeC_6_H_4_	Me	81	[9]
**18**	4-MeC_6_H_4_	H	60	[9]
**19**	4-MeOC_6_H_4_	Ph	58	[9]
**20**	4-MeOC_6_H_4_	4-MeC_6_H_4_	58	[9]
**21**	4-MeOC_6_H_4_	4-MeOC_6_H_4_	65	This work
**22**	4-MeOC_6_H_4_	4-PhC_6_H_4_	68	This work
**23**	4-MeOC_6_H_4_	2-Nap	68	This work
**24**	4-MeOC_6_H_4_	^t^Bu	35	This work
**25**	4-MeOC_6_H_4_	Me	79	[9]
**26**	4-MeOC_6_H_4_	H	34	[9]

^a^ Yields refer to the final step of the reaction sequence.

**Table 2:** MIC, CC_50_, HC_10_ ([µg/mL], and [nmol/mL]) of compounds **4**, **5**, **8**, **9**, **10**, **12**, **16**, **17**, **20**, **25**, **26**.

	***S. aureus***	***C. albicans***	***C. neoformans***	**HEK-293**	**RBC**
**Compound**	**MIC [µg/mL]**			**CC_50_ [µg/mL]**	**HC_10_ [µg/mL]**
**4**	>32	16	4	>32	>32
**5**	32	4	4	>32	>32
**8**	>32	4	16	28.10	>32
**9**	32	2	2	>32	>32
**10**	>32	32	>32	>32	>32
**12**	>32	16	8	>32	>32
**16**	>32	4	>32	>32	>32
**17**	32	2	2	31.69	>32
**20**	>32	4	>32	>32	>32
**25**	32	2	8	>32	>32
**26**	32	16	16	30.99	>32
**Compound**	**MIC [nmol/mL]**			**CC_50_ [nmol/mL]**	**HC_10_ [nmol/mL]**
**4**	>102	51	13	>102	>102
**5**	97	12	12	>97	>97
**8**	>115	14	57	100.63	>115
**9**	135	8	8	>135	>135
**10**	>143	143	>143	>143	>143
**12**	>98	49	24	>98	>98
**16**	>109	14	>109	>109	>109
**17**	127	8	8	126.16	>127
**20**	>93	12	>93	>93	>93
**25**	120	7	30	>120	>120
**26**	126	63	63	122.41	>126

## Data Availability

Not applicable.

## Supplementary Materials

The following are available online: Figures S1–S124: NMR spectra of the compounds. Table S1: Crystallographic and refinement details for all X-ray structures reported herein. Full experimental details of the biological studies and X-ray crystallography.

## Sample Availability

Samples of the compounds are available from the authors upon request.
