# Supplementary material for: Synthesis, Reactivity and Antimicrobial Activity of a Series of 2-Arylamino-1,3-selenazoles
Source: Molecules. 2021 Dec 20;26(24):7695. doi: 10.3390/molecules26247695 (PMC8706435; doi:10.3390/molecules26247695)

Supplementary Material

Synthesis, Reactivity and Antimicrobial Activity of a Series of 2-Arylamino-1,3-Selenazoles.

Julia Kuchar [1], Katharina Reinhold [1], Vera Rösgen [1], Nils Nöthling [2], Christian W. Lehmann [2] and Fabian Mohr [1,*]

[1] Anorganische Chemie, Fakultät für Mathematik und Naturwissenschaften, Bergische Universität Wuppertal, Gaußstr. 20, 42119 Wuppertal, Germany.

[2] Max-Planck-Institut für Kohlenforschung, Kaiser-Wilhelm-Platz 1, 45470 Mülheim an der Ruhr, Germany.

Contents

Figures S 1-S 124. Proton, carbon and selenium NMR spectra of the compounds.

Table S 1. Crystallographic and refinement details.

Experimental details of the X-ray structure determinations

Experimental details of the biological studies

Results of the NCI 60 screening

Compound 2a

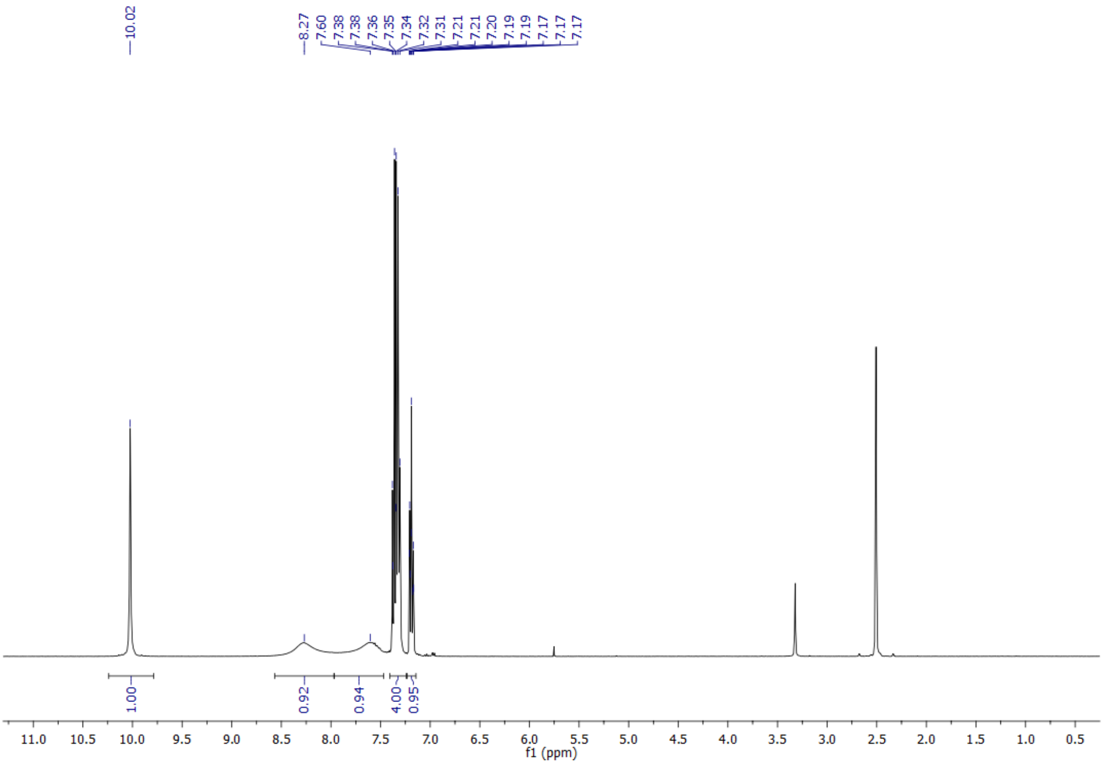

Figure S 1: ^{1}H-NMR (400 MHz, DMSO-d_6) of compound 2a

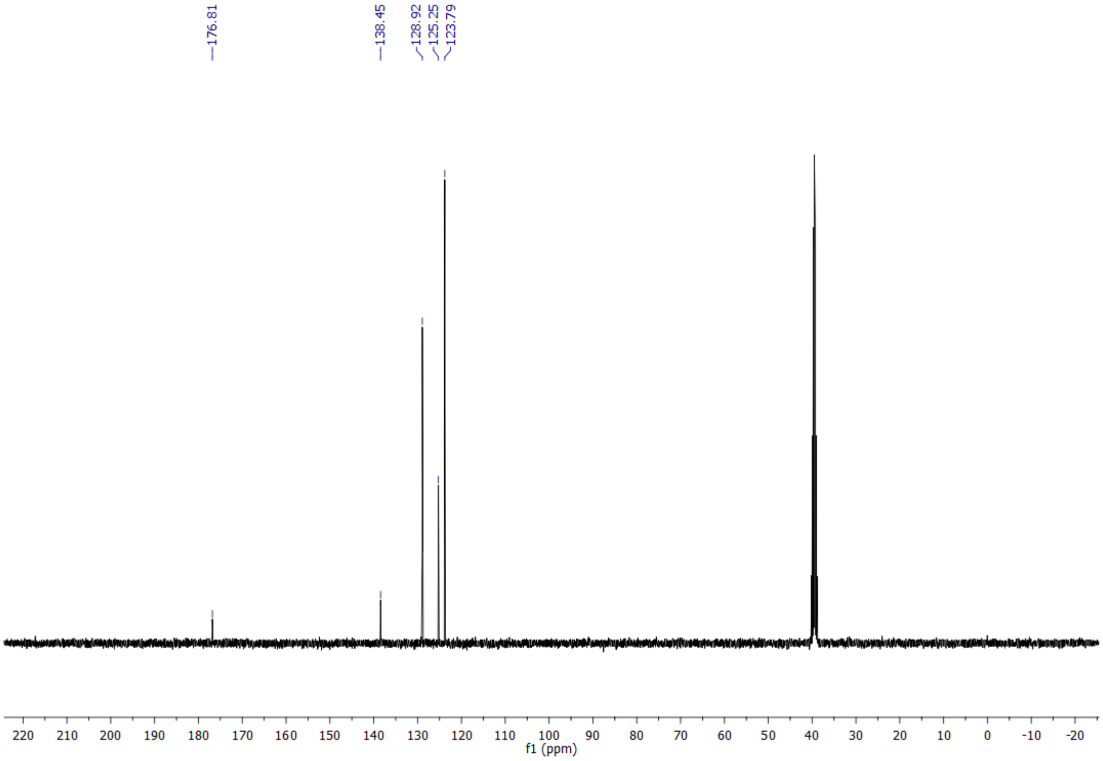

Figure S 2: ^{13}C-NMR (101 MHz, DMSO-d_6) of compound 2a

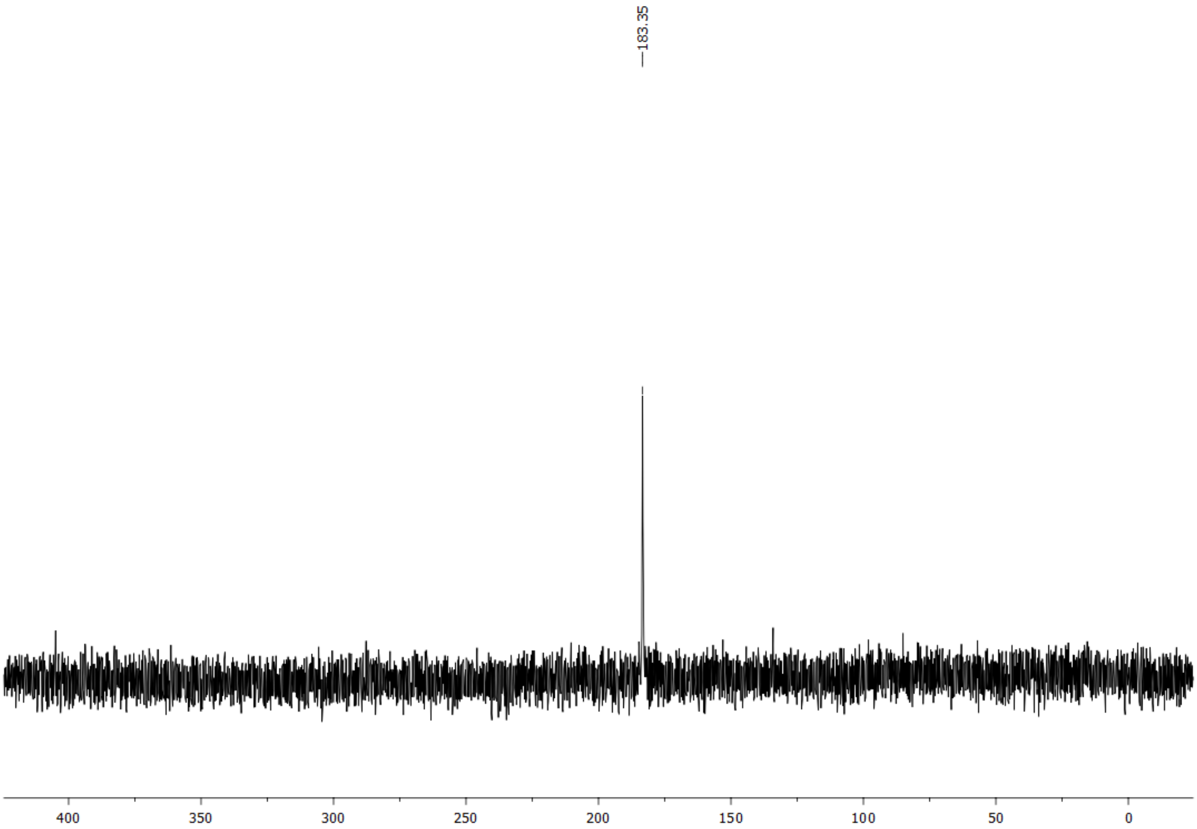

Figure S 3: ^{77}Se-NMR (76 MHz, MeOD, 248 K) of compound 2a

Compound 2b

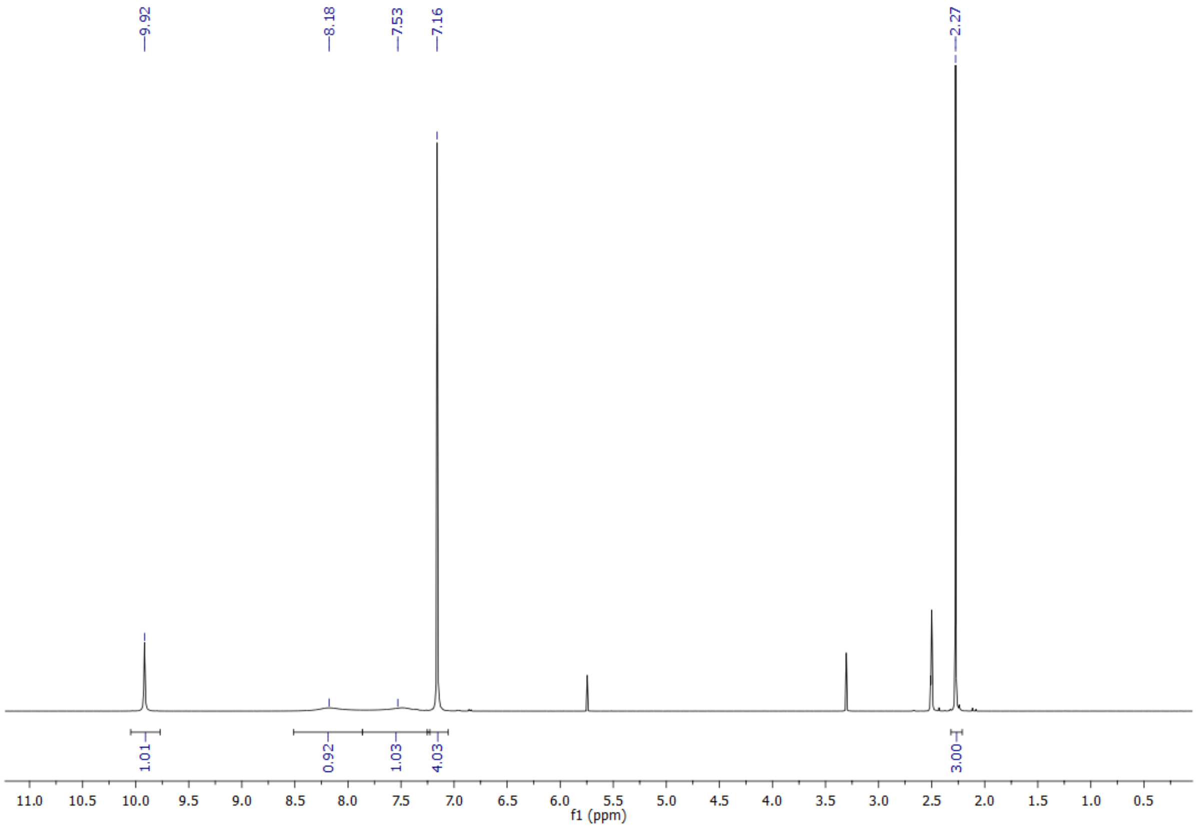

Figure S 4: ^{1}H-NMR (400 MHz, DMSO-d_6) of compound 2b

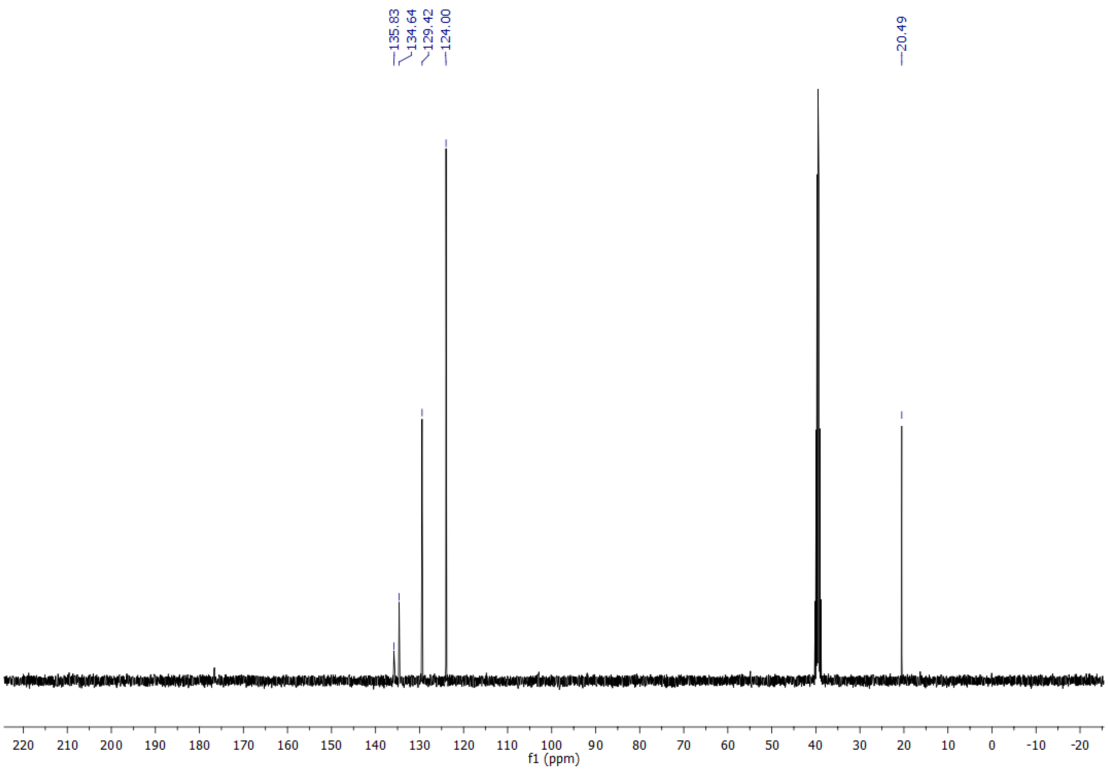

Figure S 5: ^{13}C-NMR (101 MHz, DMSO-d$_6$) of compound 2b

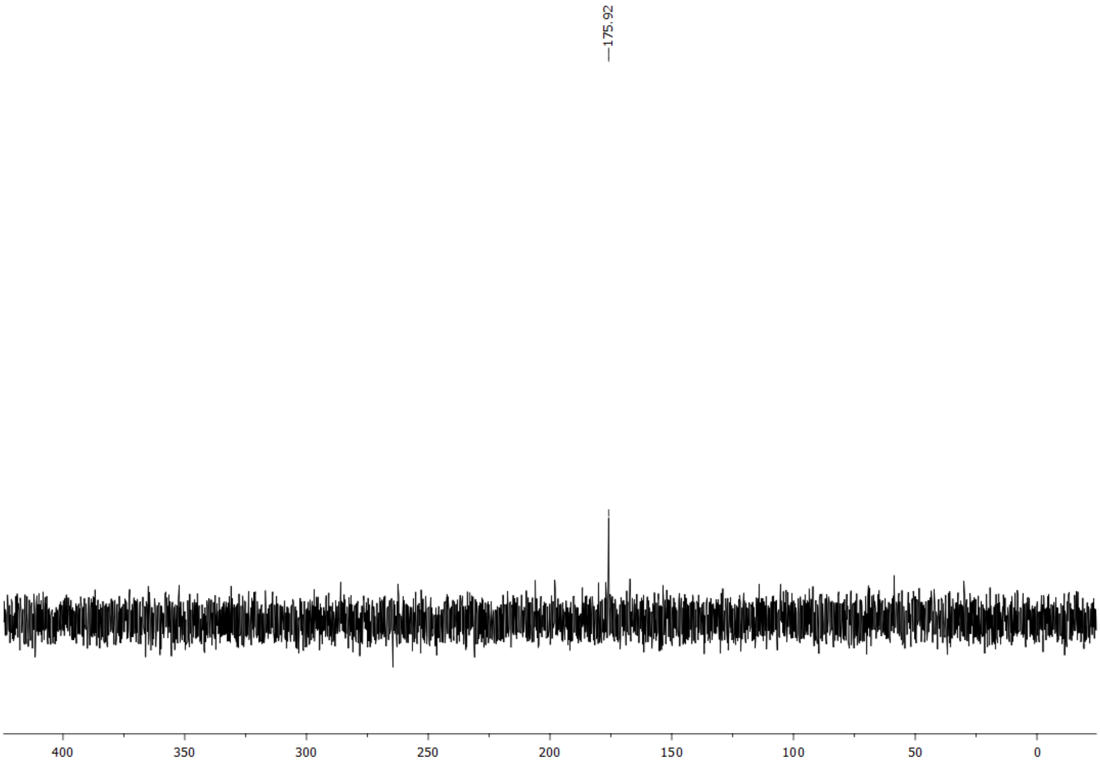

Figure S 6: ^{77}Se-NMR (76 MHz, MeOD, 248K) of compound 2b

Compound 2c

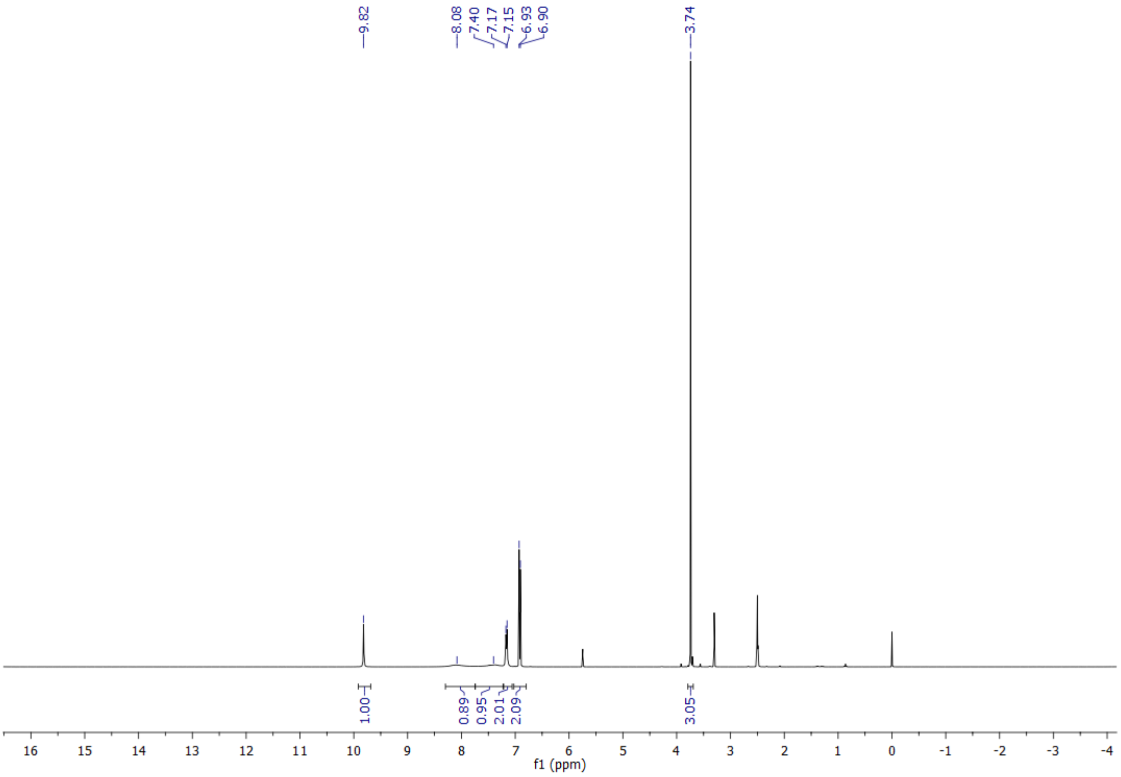

Figure S 7: ¹H-NMR (600 MHz, DMSO-d_6) of compound 2c

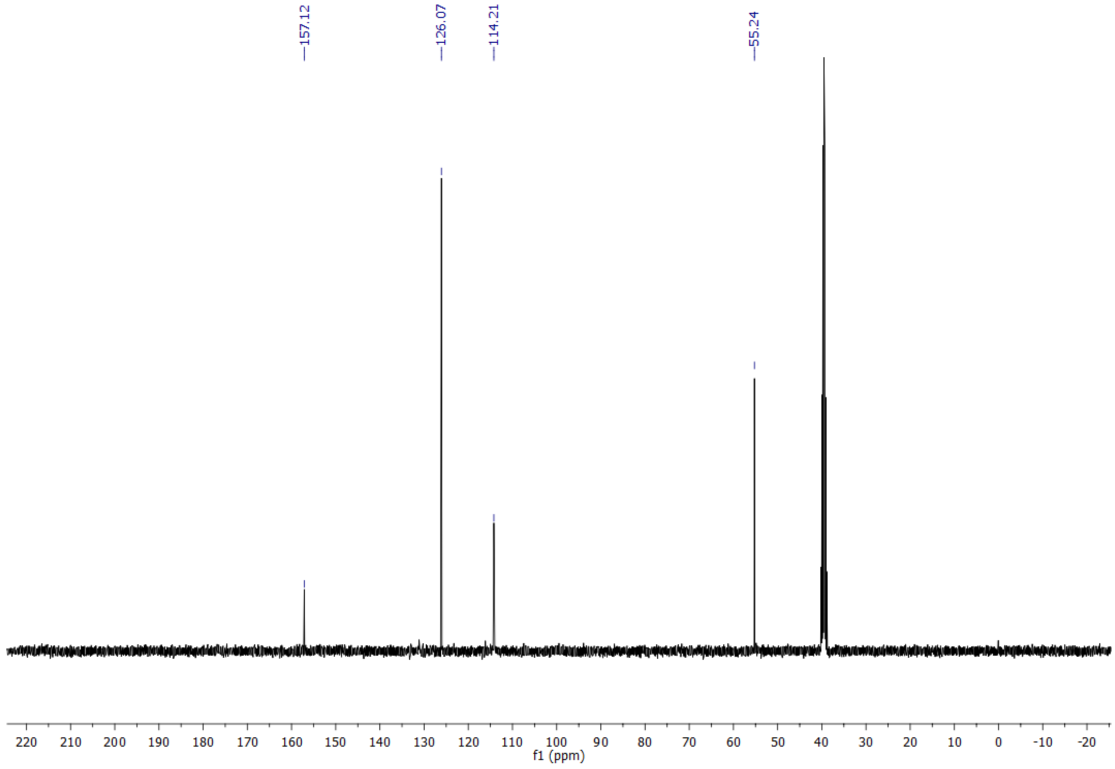

Figure S 8: ¹³C-NMR (151 MHz, DMSO-d_6) of compound 2c

Compound 3

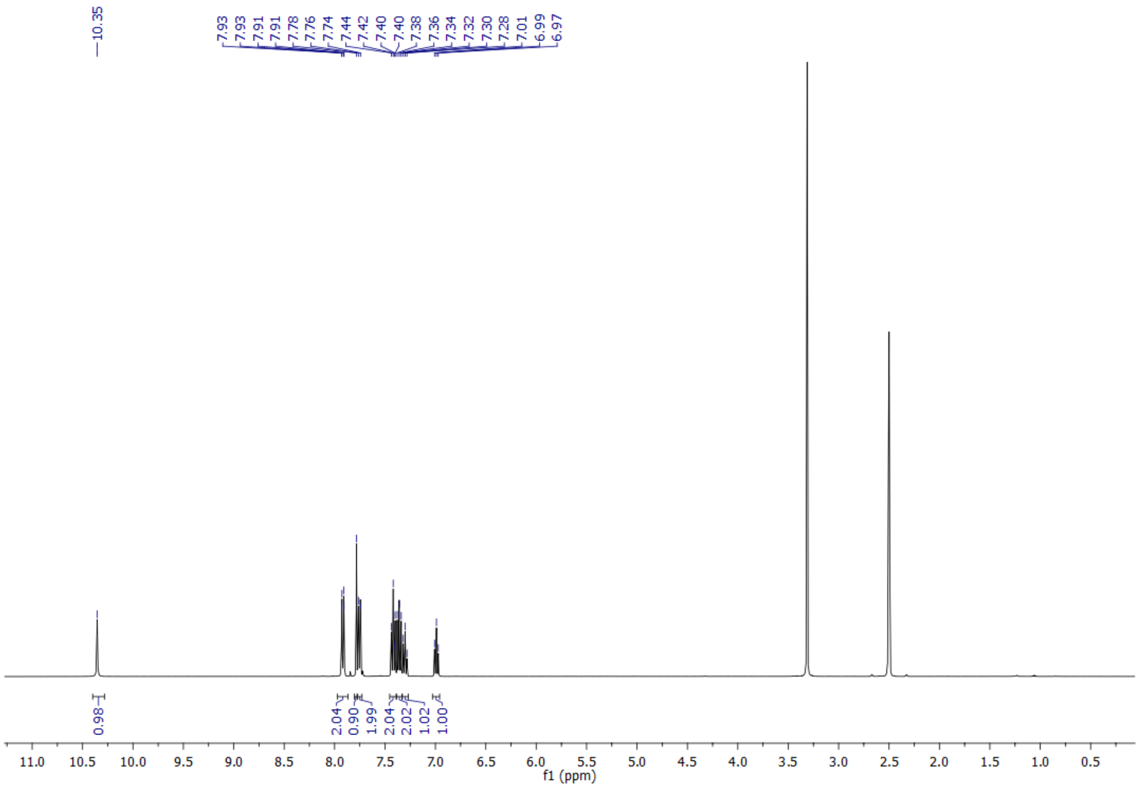

Figure S 9: ^{1}H-NMR (400 MHz, DMSO-d_6) of compound 3

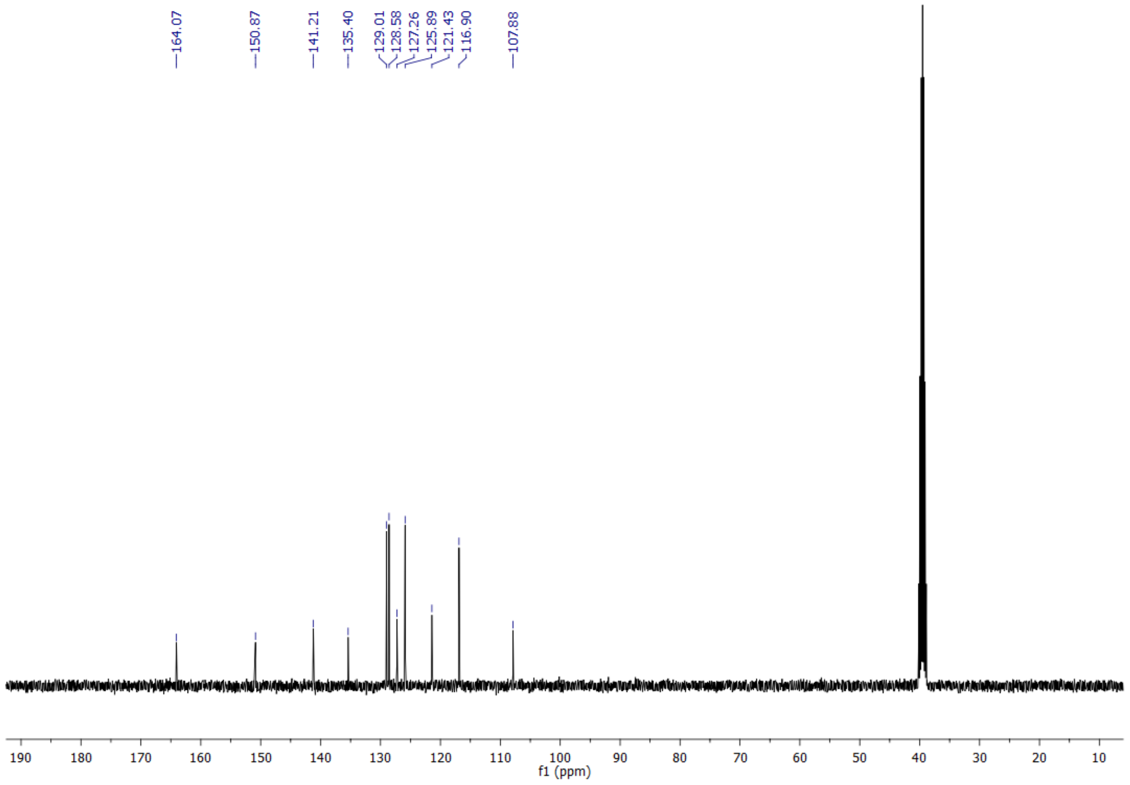

Figure S 10: ^{13}C-NMR (101 MHz, DMSO-d_6) of compound 3

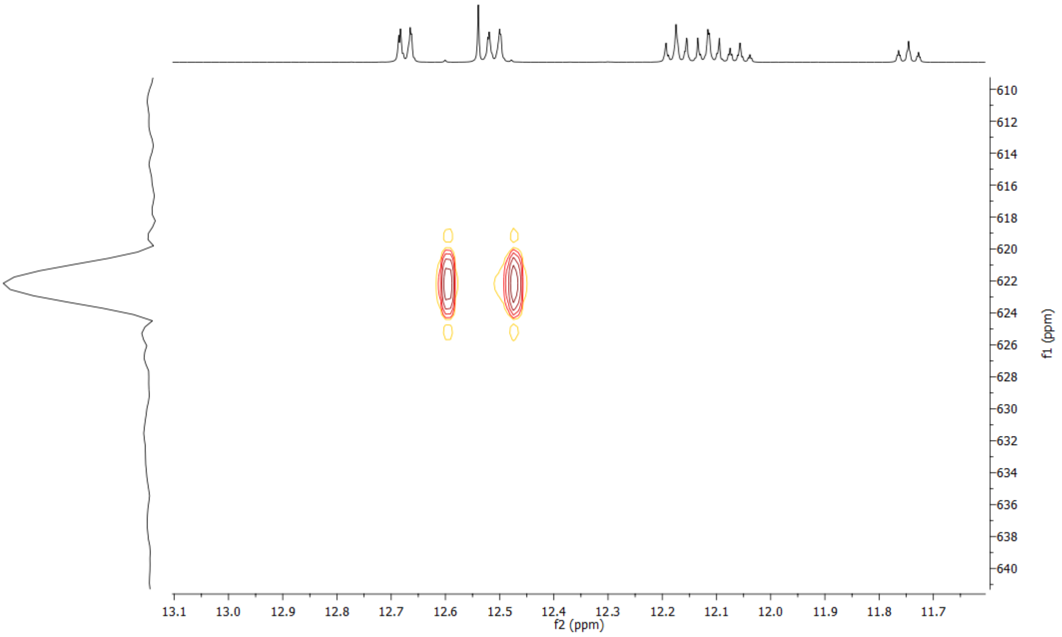

Figure S 11: ^{1}H-^{77}Se-HMBC (400 MHz, 76 MHz, DMSO-d$_6$) of compound 3

Compound 4

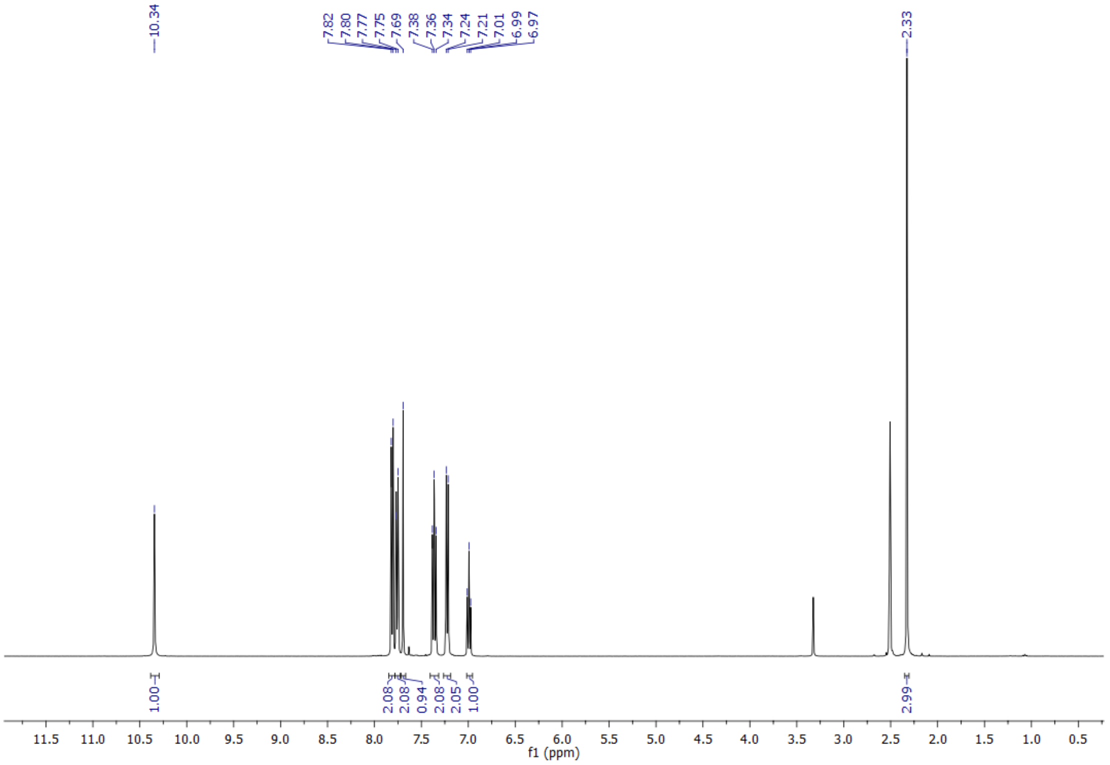

Figure S 12: ^{1}H-NMR (400 MHz, DMSO-d$_6$) of compound 4

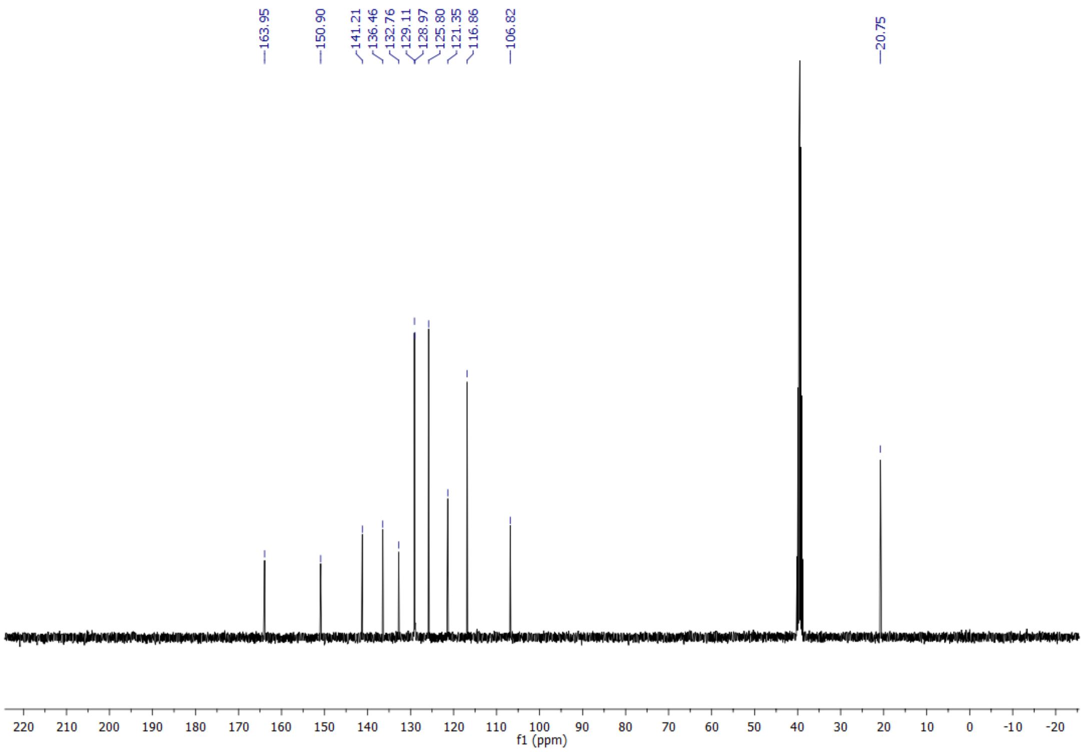

Figure S 13: ^{13}C-NMR (101 MHz, DMSO-d$_6$) of compound 4

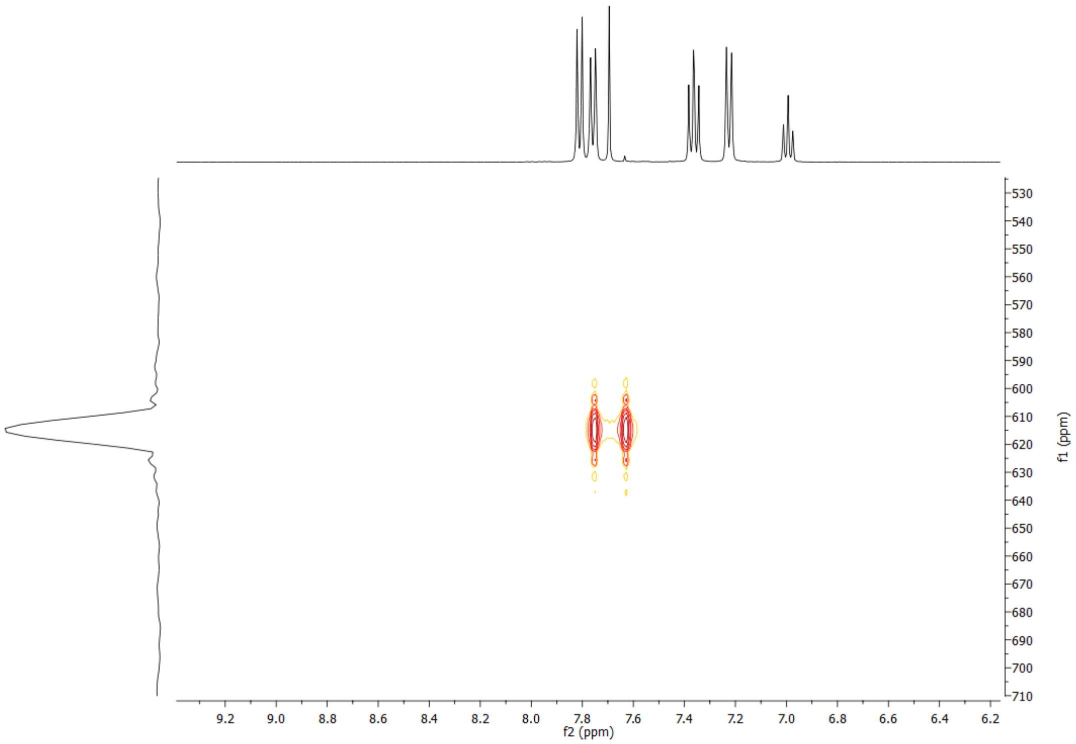

Figure S 14: ^{1}H-^{77}Se-HMBC (400 MHz, 76 MHz, DMSO-d$_6$) of compound 4

Compound 5

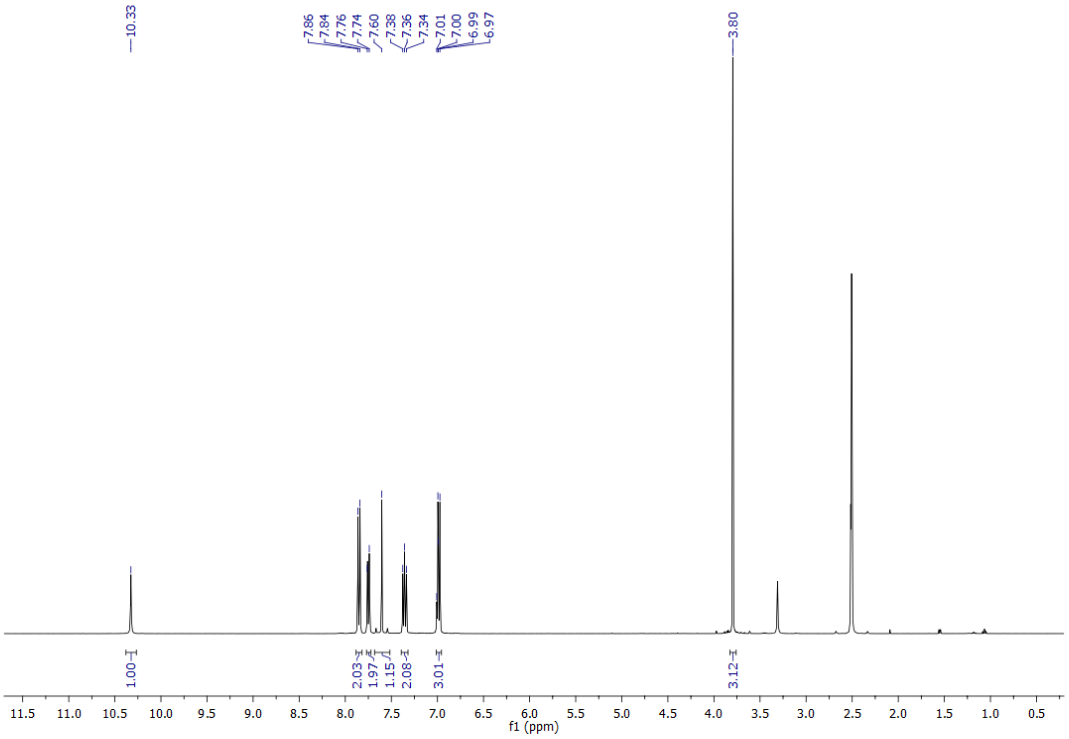

Figure S 15: ^{1}H-NMR (400 MHz, DMSO-d_6) of compound 5

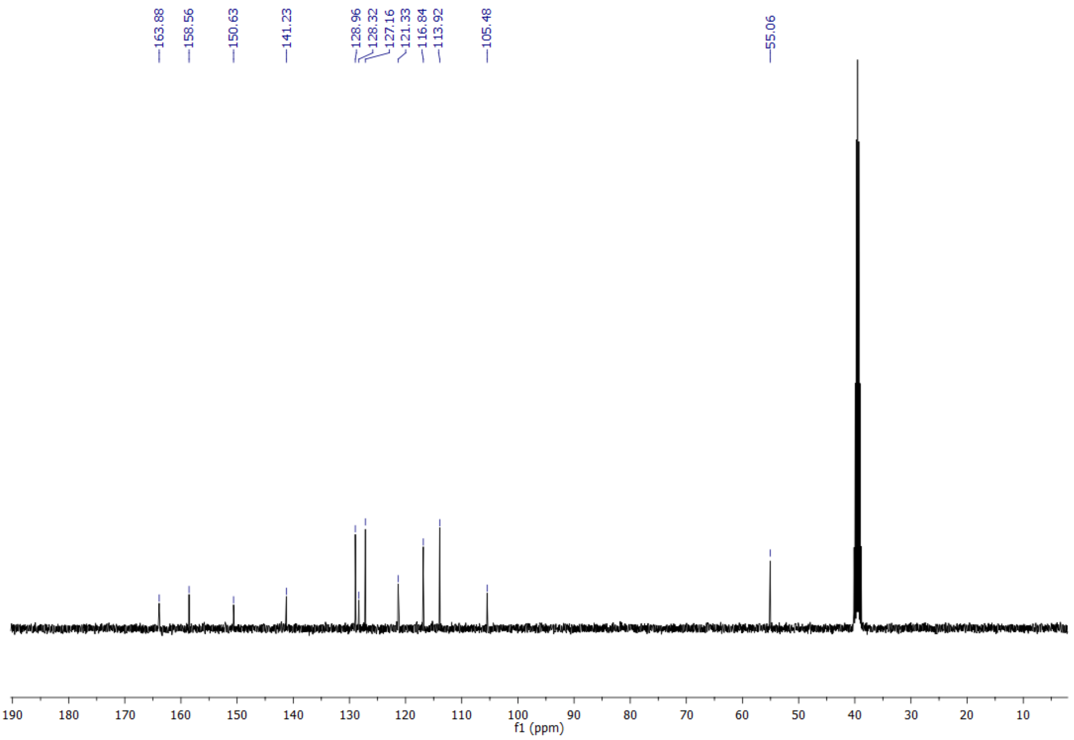

Figure S 16: ^{13}C-NMR (101 MHz, DMSO-d_6) of compound 5

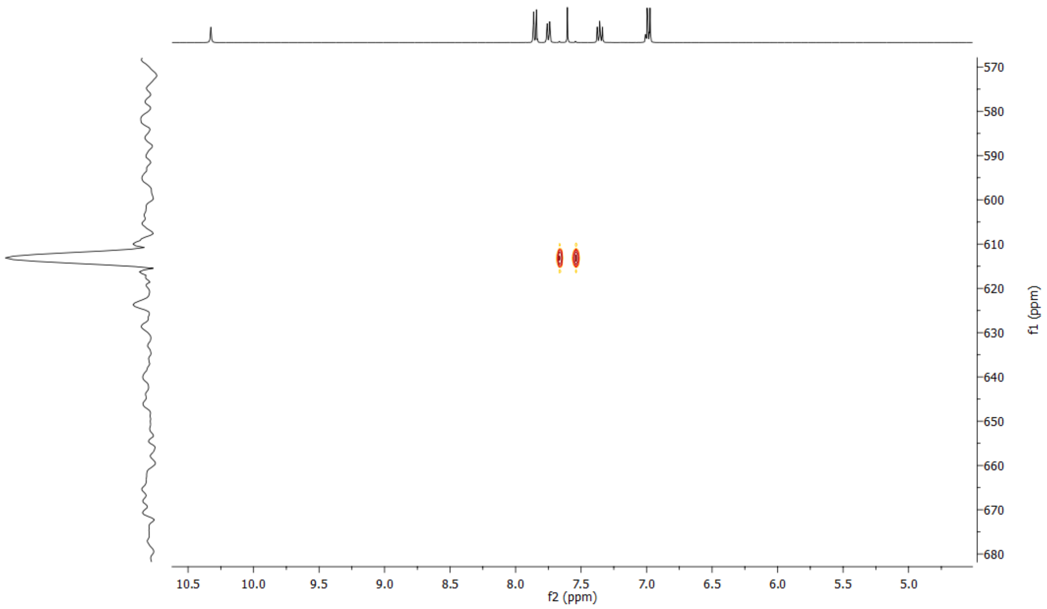

Figure S 17: ^{1}H-^{77}Se-HMBC (400 MHz, 76 MHz, DMSO-d_6) of compound 5

Compound 6

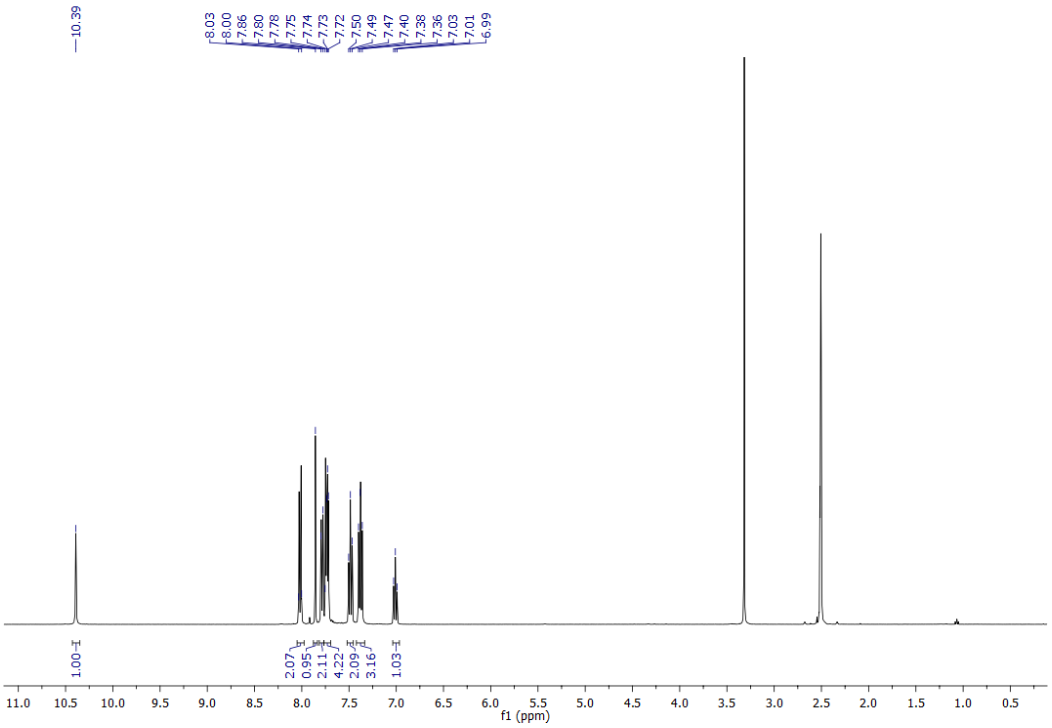

Figure S 18: ^{1}H-NMR (400 MHz, DMSO-d_6) of compound 6

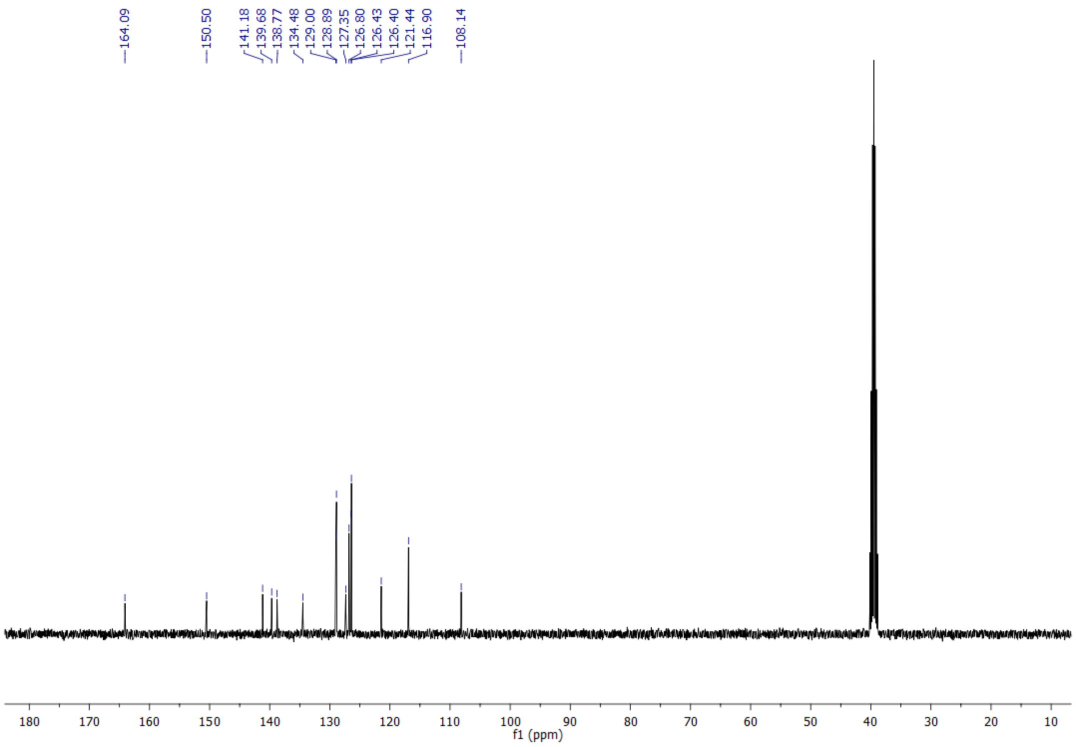

Figure S 19: ^{13}C-NMR (101 MHz, DMSO-d_6) of compound 6

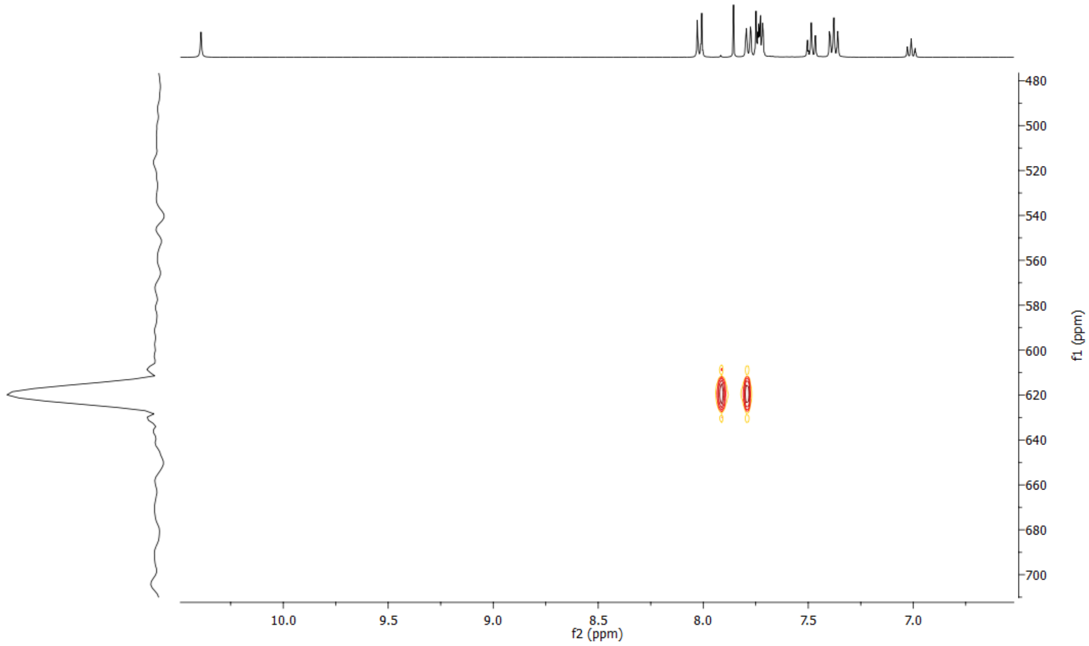

Figure S 20: ^{1}H-^{77}Se-HMBC (400 MHz, 76 MHz, DMSO-d_6) of compound 6

Compound 7

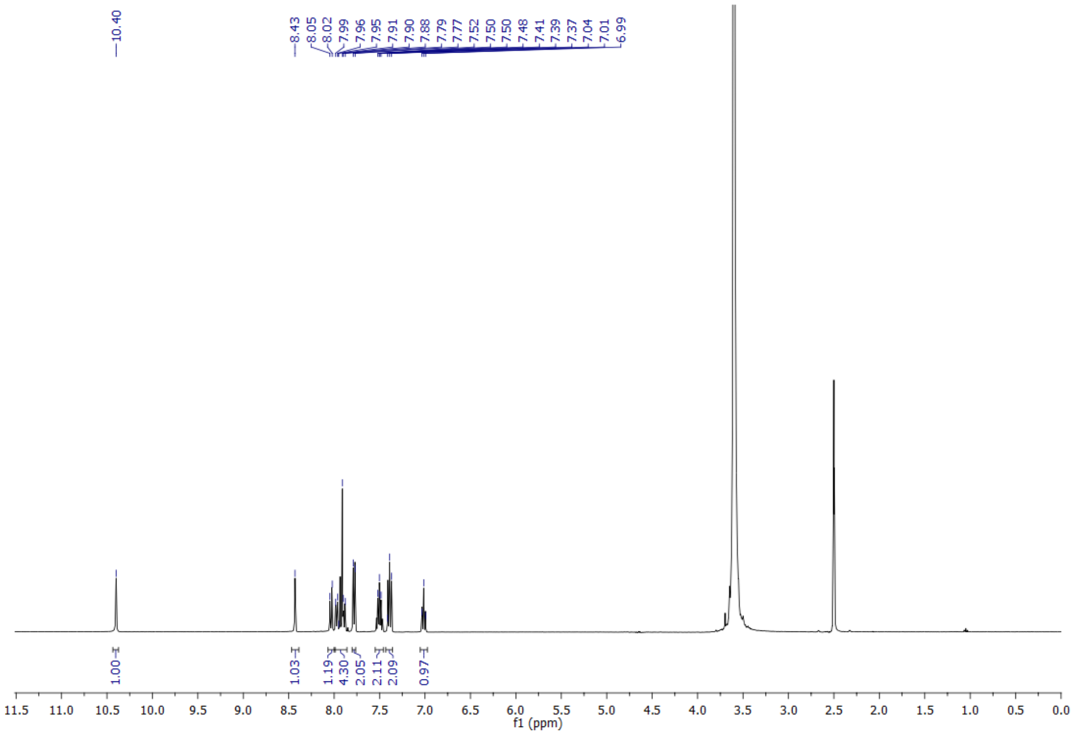

Figure S 21: ^{1}H-NMR (400 MHz, DMSO-d_6) of compound 7

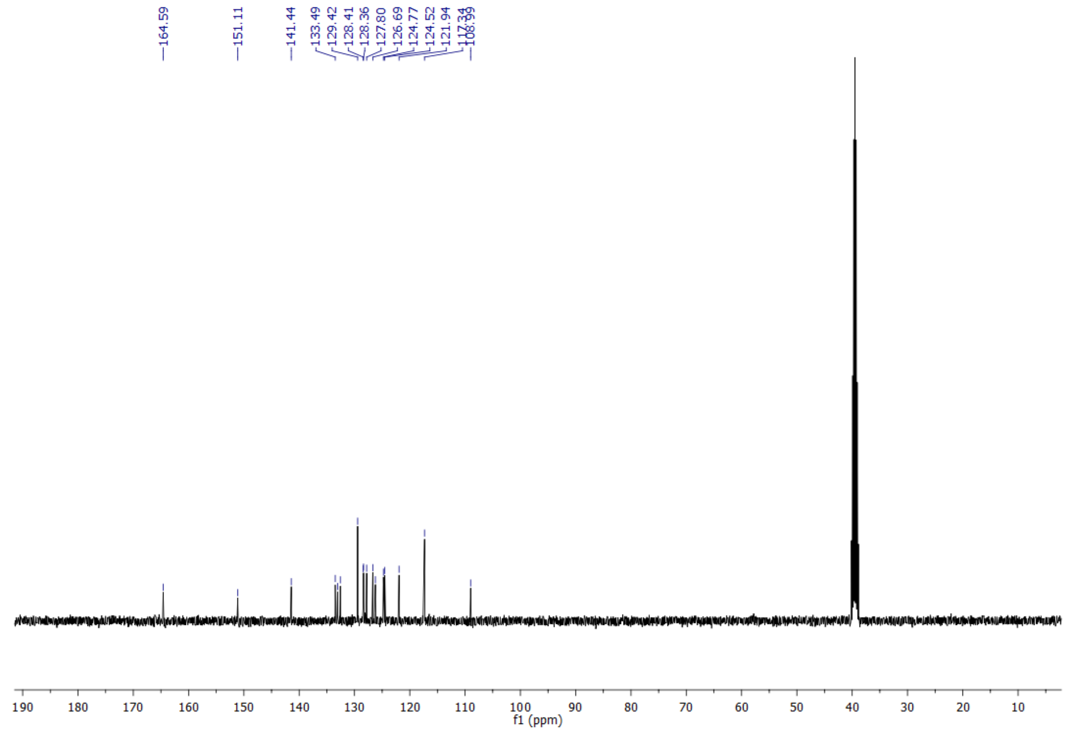

Figure S 22: ^{13}C-NMR (101 MHz, DMSO-d_6) of compound 7

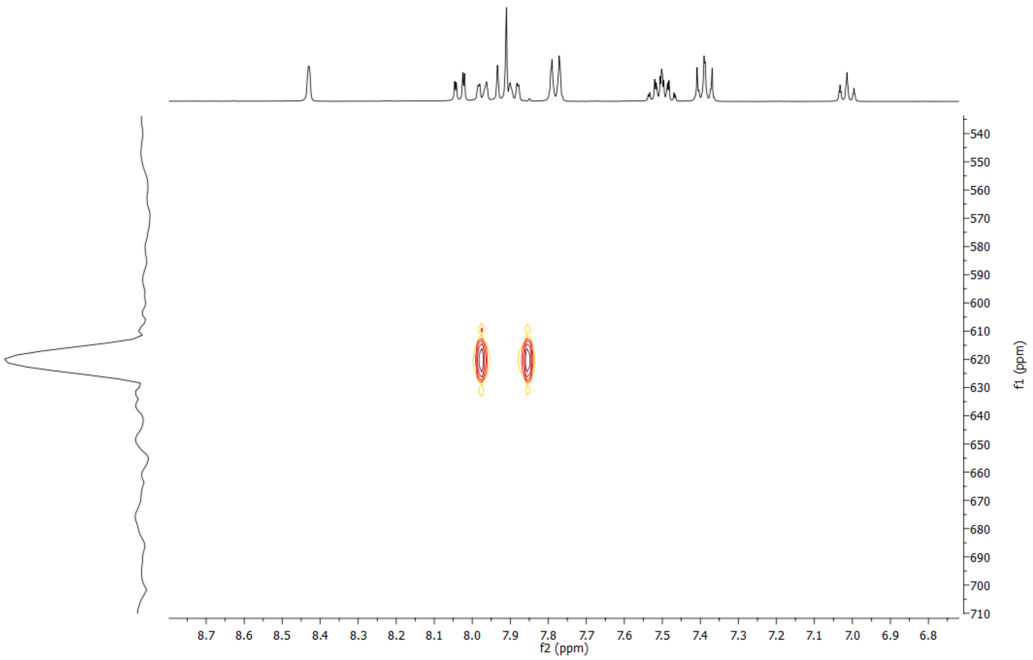

Figure S 23: ^{1}H-^{77}Se-HMBC (400 MHz, 76 MHz, DMSO-d_6) of compound 7

Compound 8

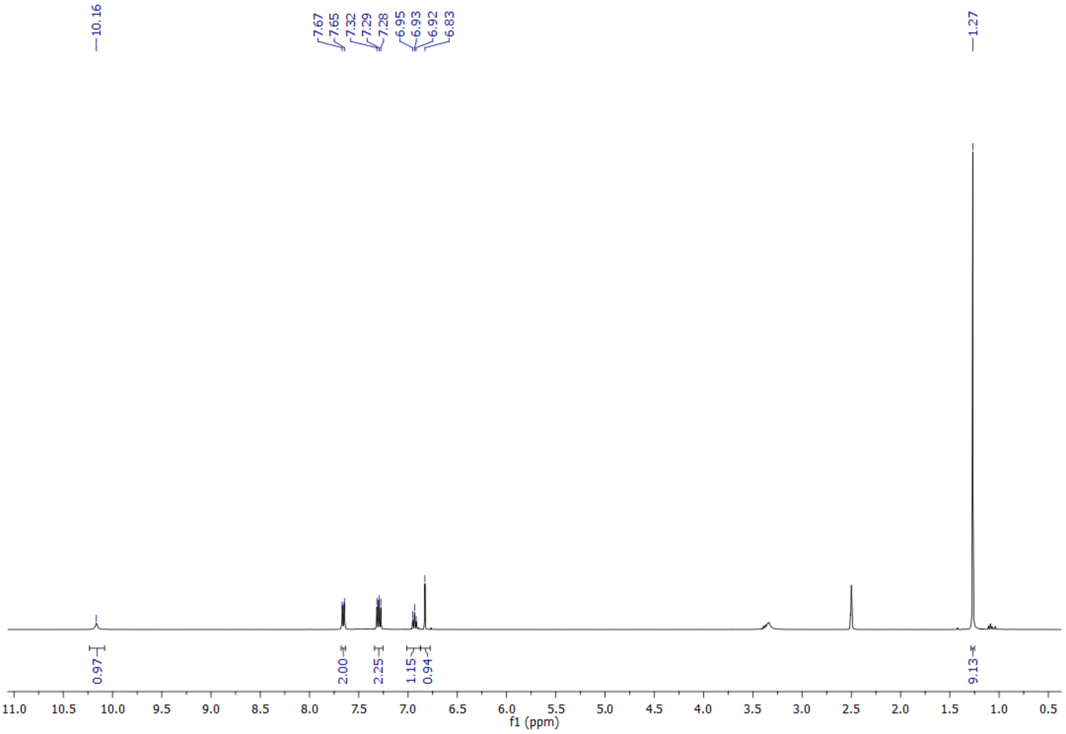

Figure S 24: ^{1}H-NMR (400 MHz, DMSO-d_6) of compound 8

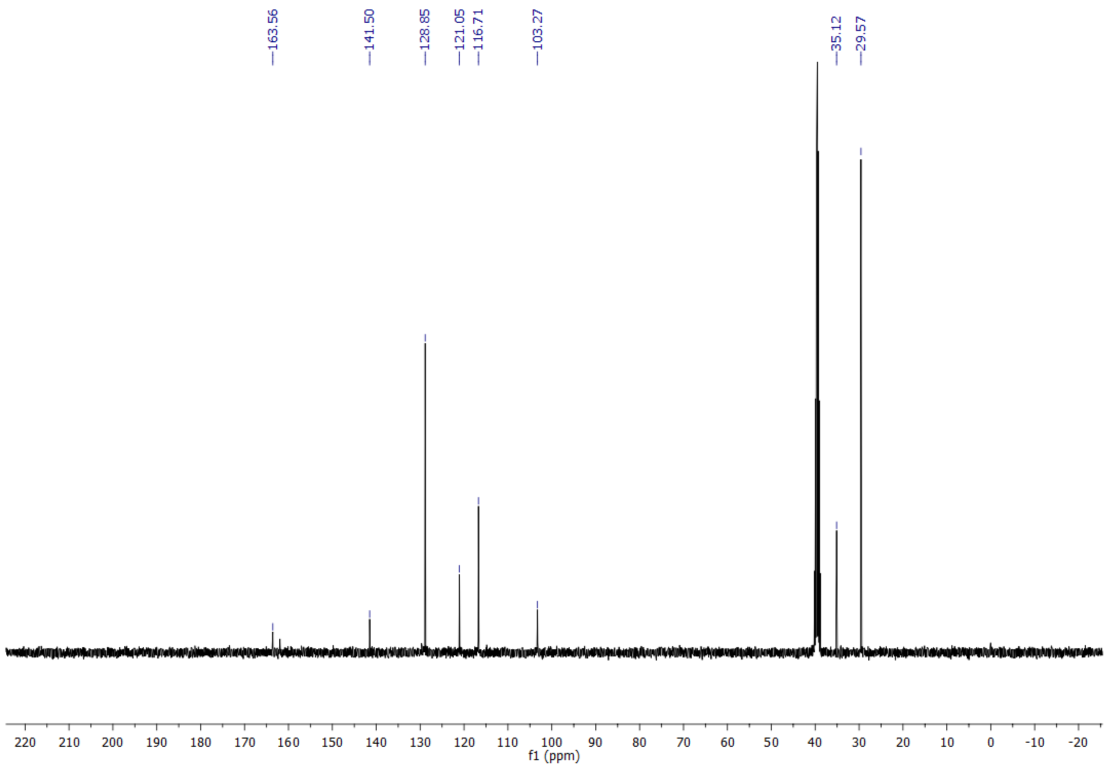

Figure S 25: ^{13}C-NMR (101 MHz, DMSO-d_6) of compound 8

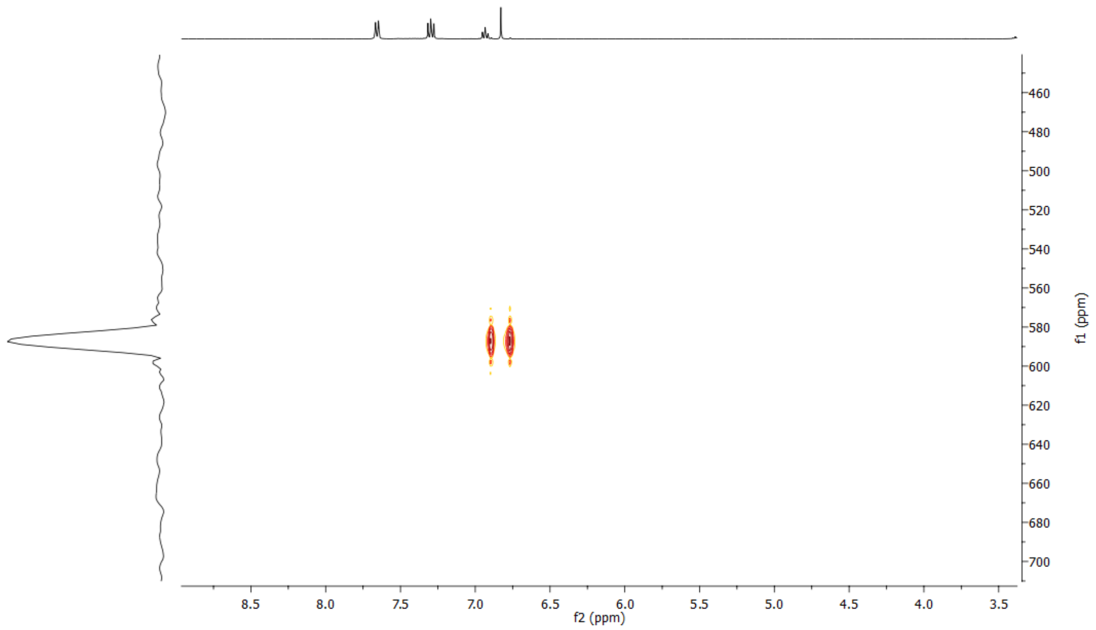

Figure S 26: ^{1}H-^{77}Se-HMBC (400 MHz, 76 MHz, DMSO-d_6) of compound 8

Compound 9

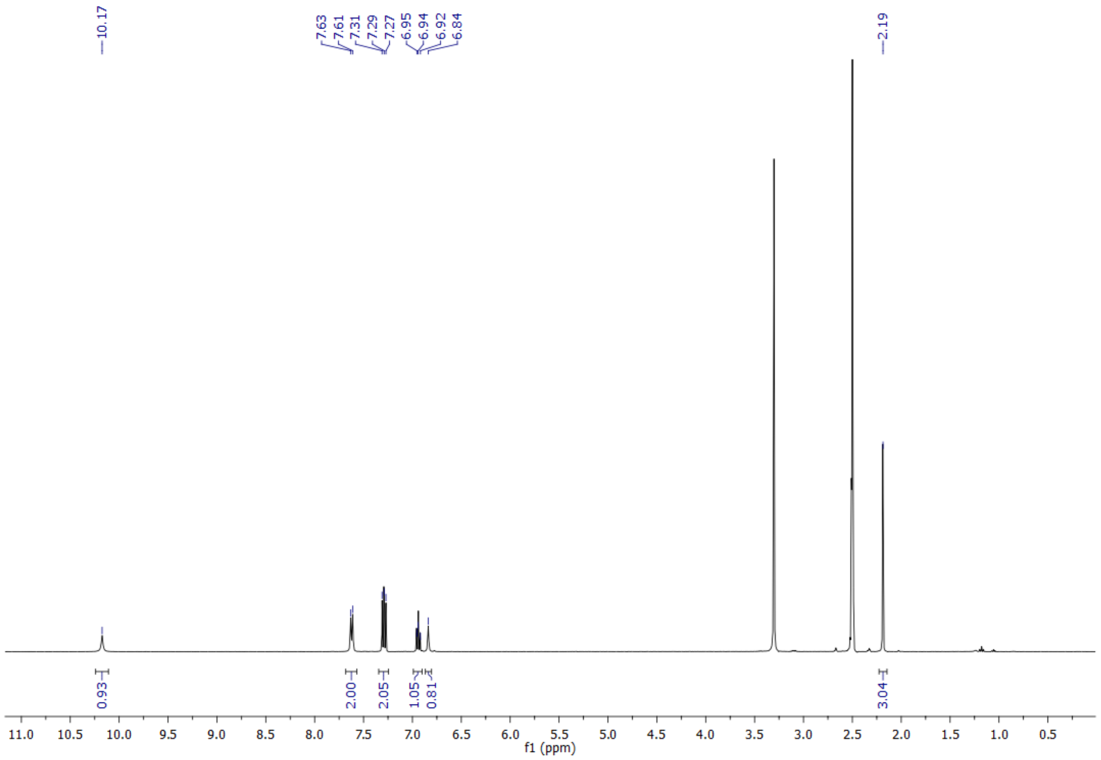

Figure S 27: ^{1}H-NMR (400 MHz, DMSO-d_6) of compound 9

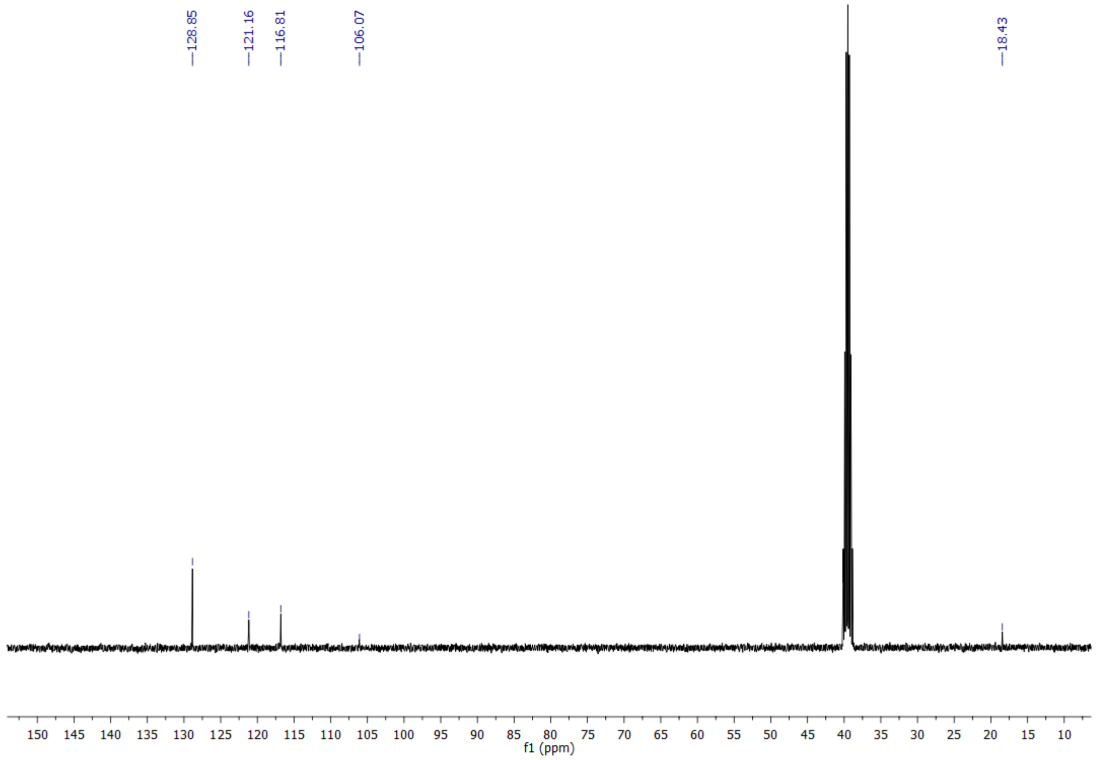

Figure S 28: ^{13}C-NMR (101 MHz, DMSO-d_6) of compound 9

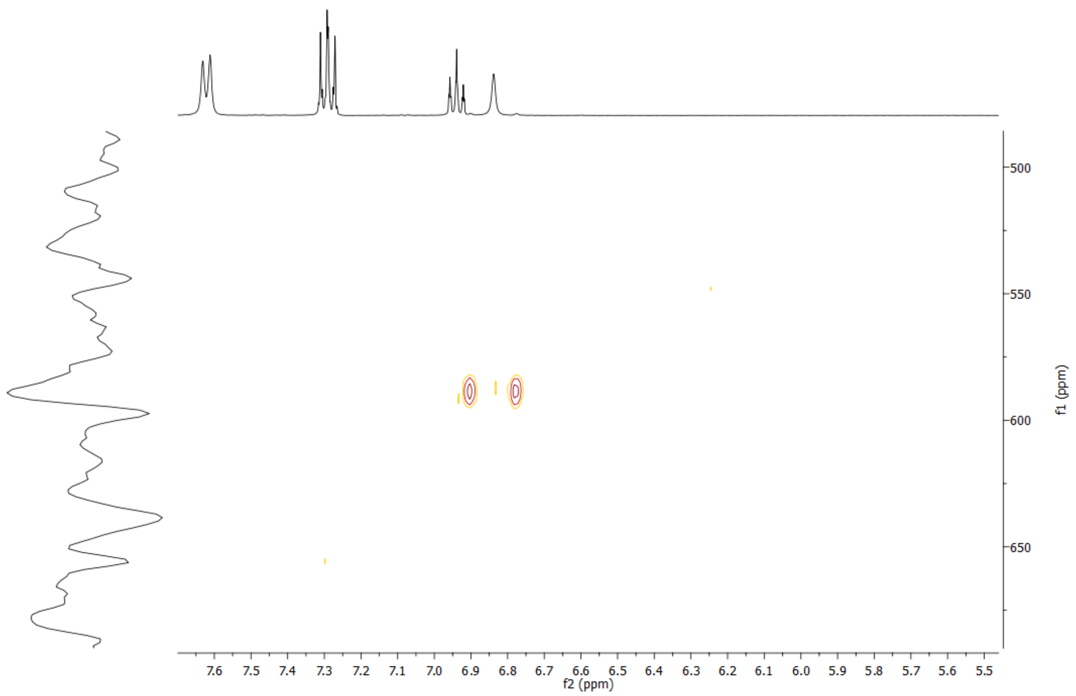

Figure S 29: ^{1}H-^{77}Se-HMBC (400 MHz, 76 MHz, DMSO-d_6) of compound 9

Compound 10

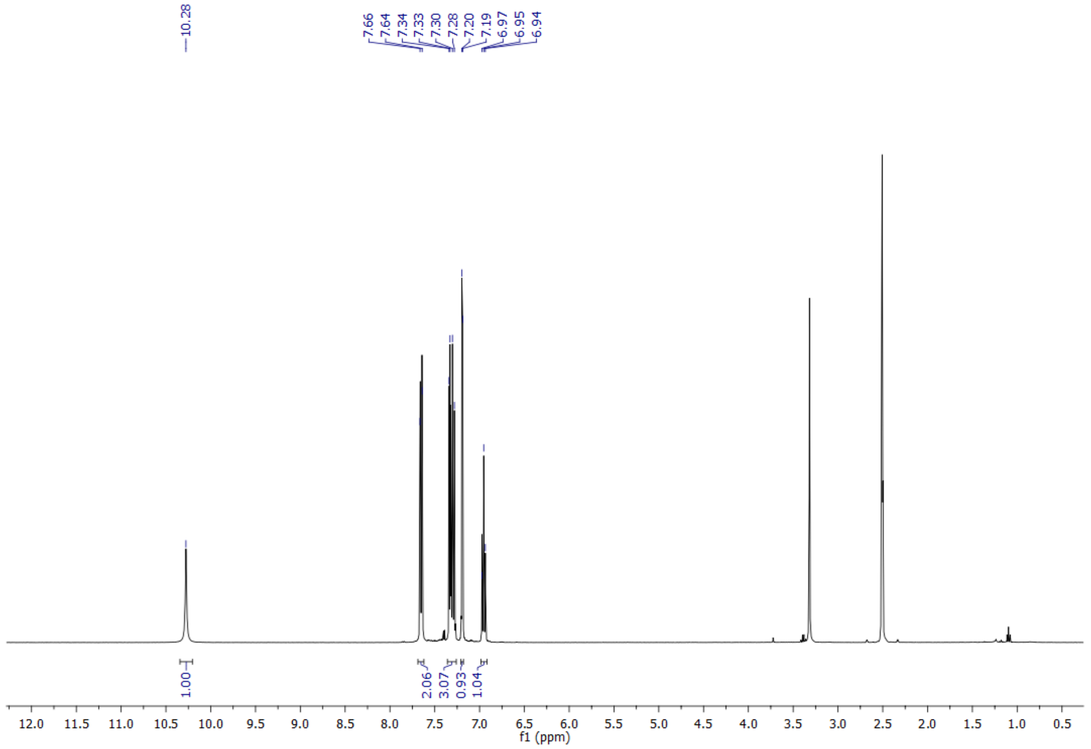

Figure S 30: ^{1}H-NMR (400 MHz, DMSO-d_6) of compound 10

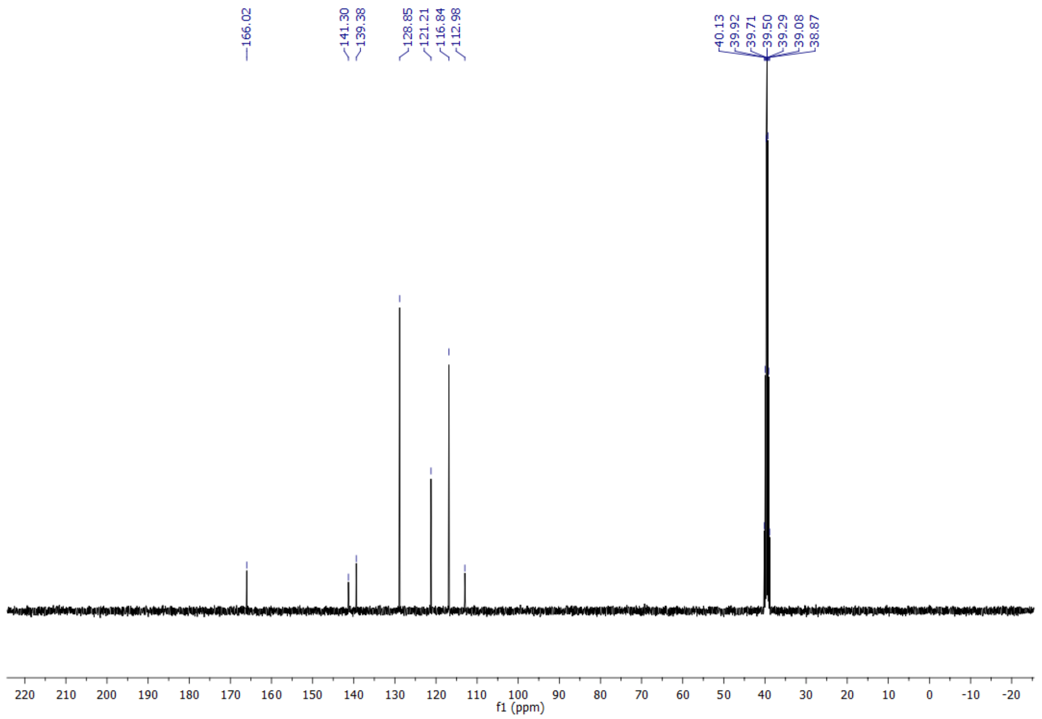

Figure S 31: ^{13}C-NMR (101 MHz, DMSO-d_6) of compound 10

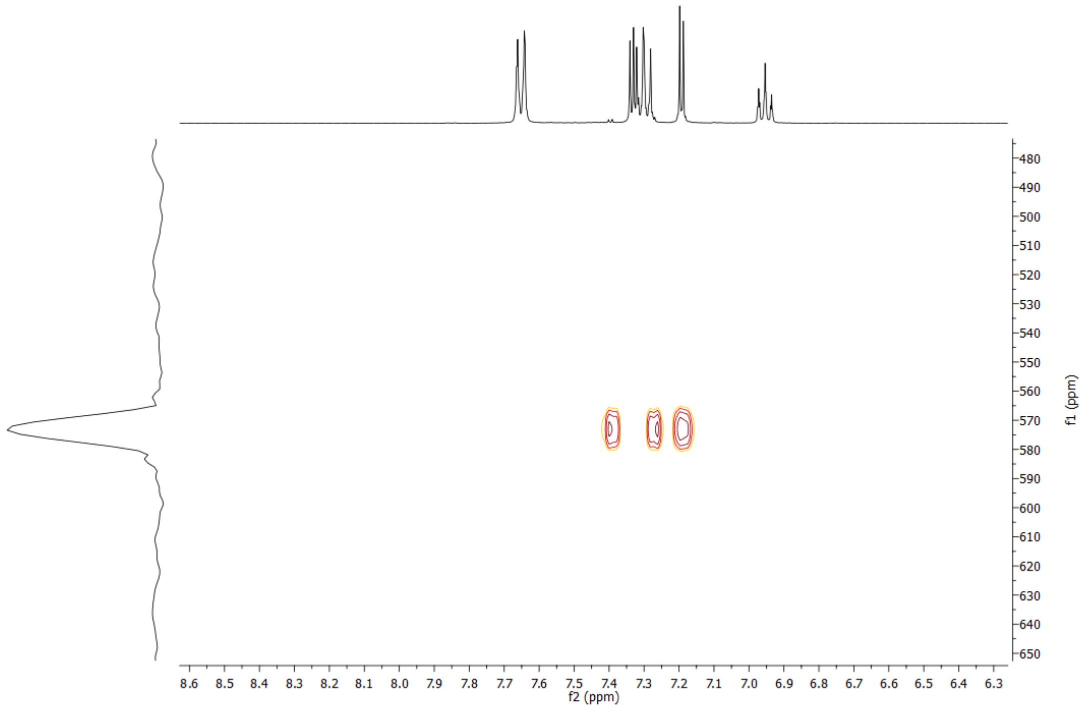

Figure S 32: ^{1}H-^{77}Se-HMBC (400 MHz, 76 MHz, DMSO-d_6) of compound 10

Compound 11

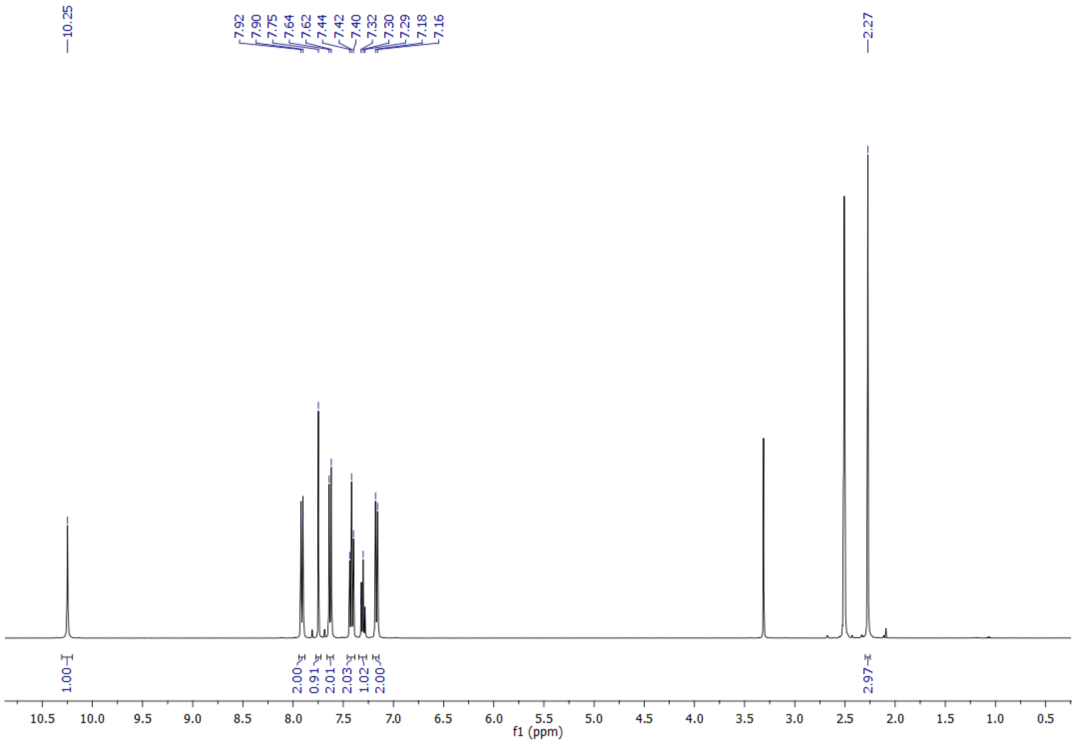

Figure S 33

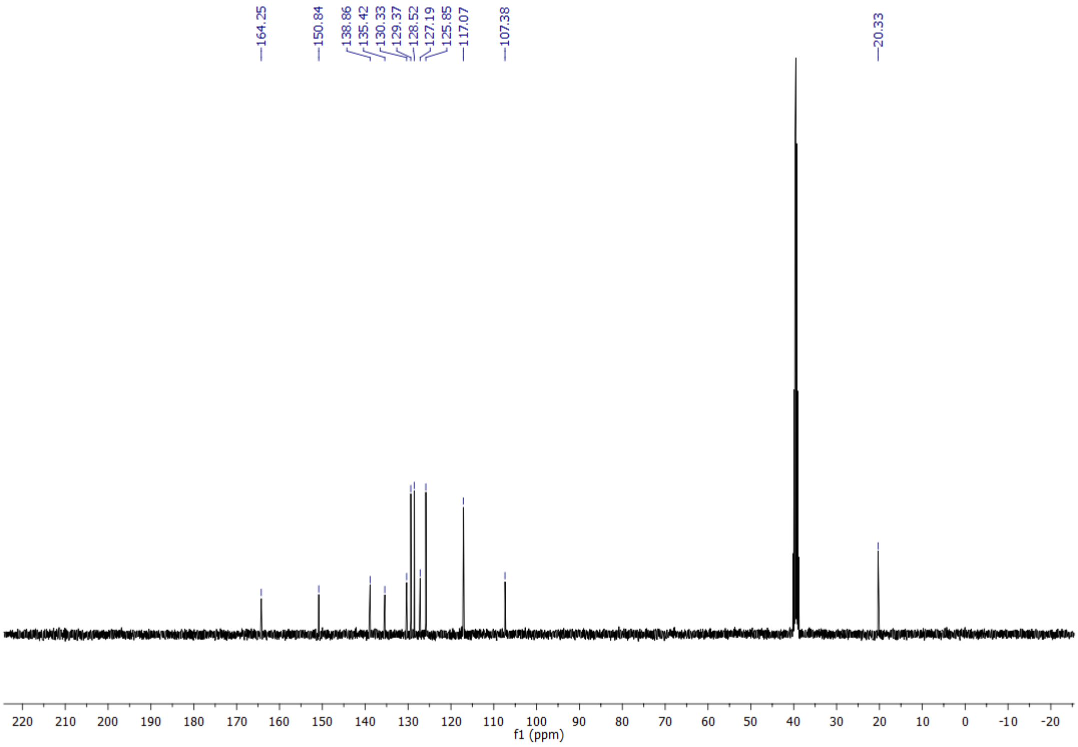

Figure S 34: ^{13}C-NMR (101 MHz, DMSO-d_6) of compound 11

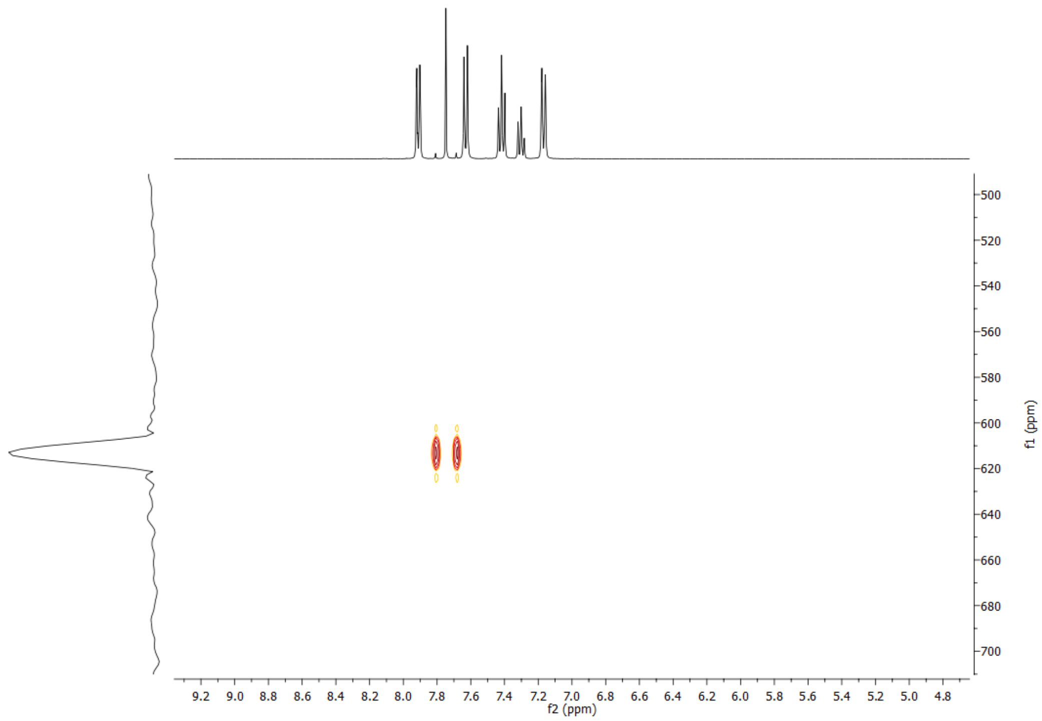

Figure S 35: ^{1}H-^{77}Se-HMBC (400 MHz, 76 MHz, DMSO-d_6) of compound 11

Compound 12

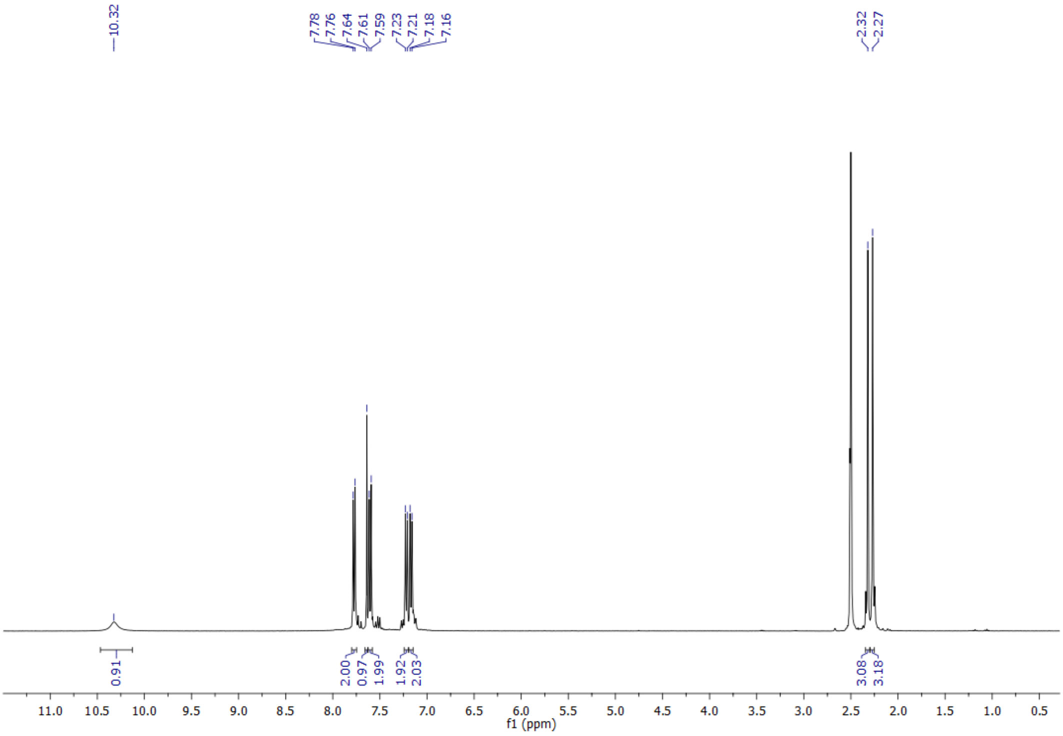

Figure S 36: ^{1}H-NMR (400 MHz, DMSO-d_6) of compound 12

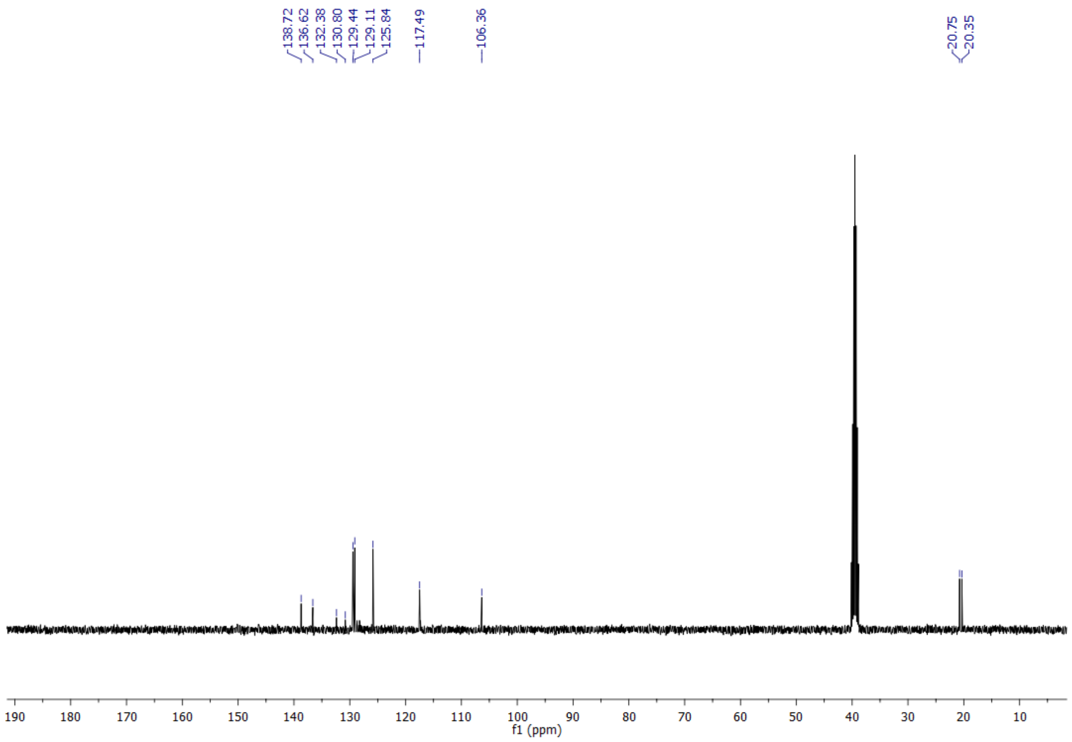

Figure S 37: ^{13}C-NMR (101 MHz, DMSO-d_6) of compound 12

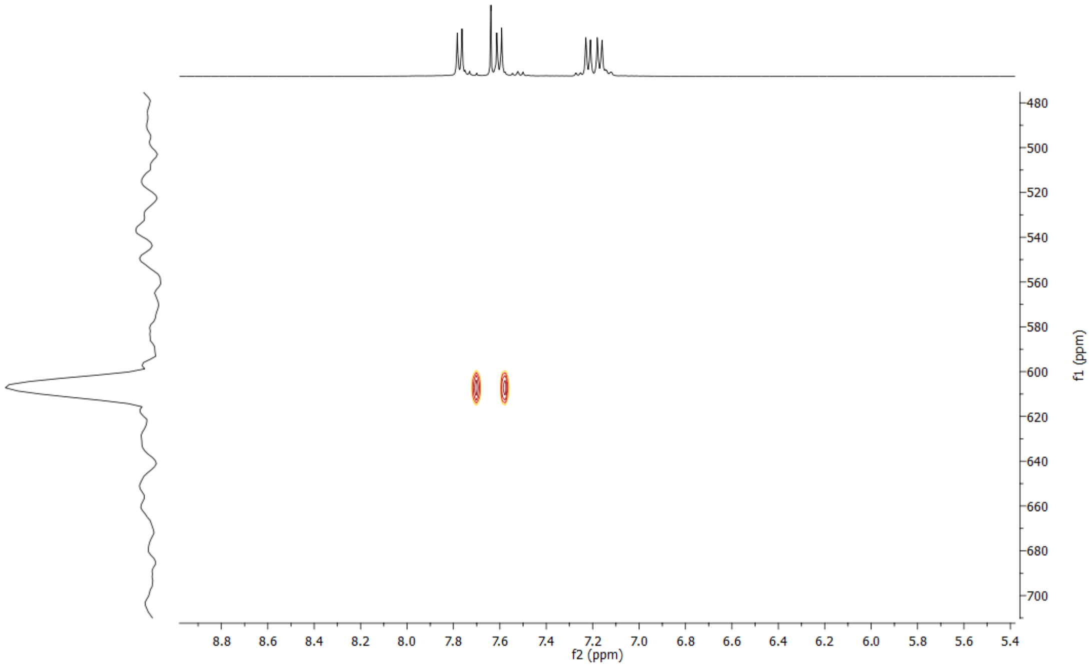

Figure S 38: ^{1}H-^{77}Se-HMBC (400 MHz, 76 MHz, DMSO-d_6) of compound 12

Compound 13

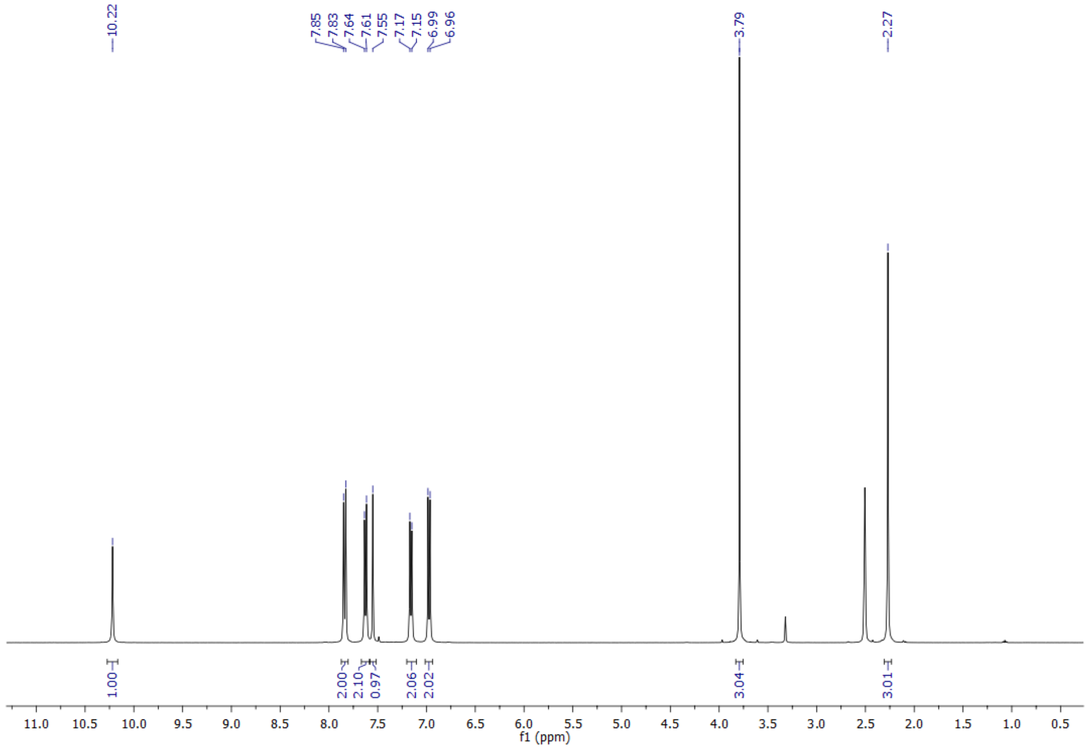

Figure S 39: ^{1}H-NMR (400 MHz, DMSO-d_6) of compound 13

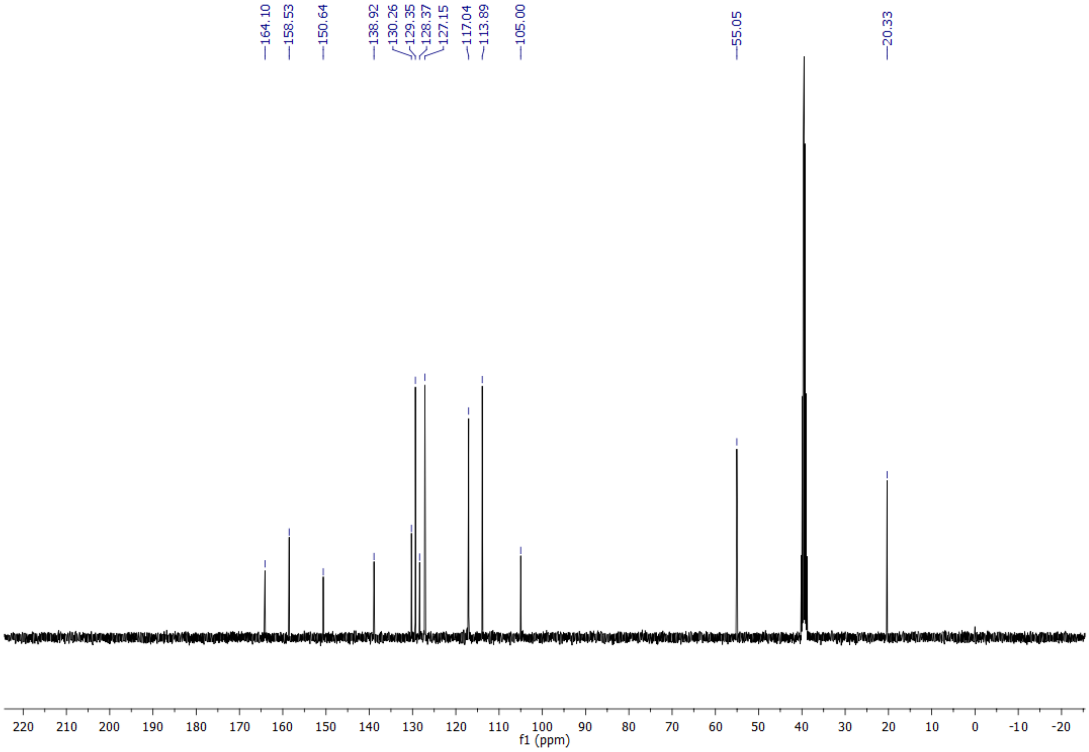

Figure S 40: ^{13}C-NMR (101 MHz, DMSO-d_6) of compound 13

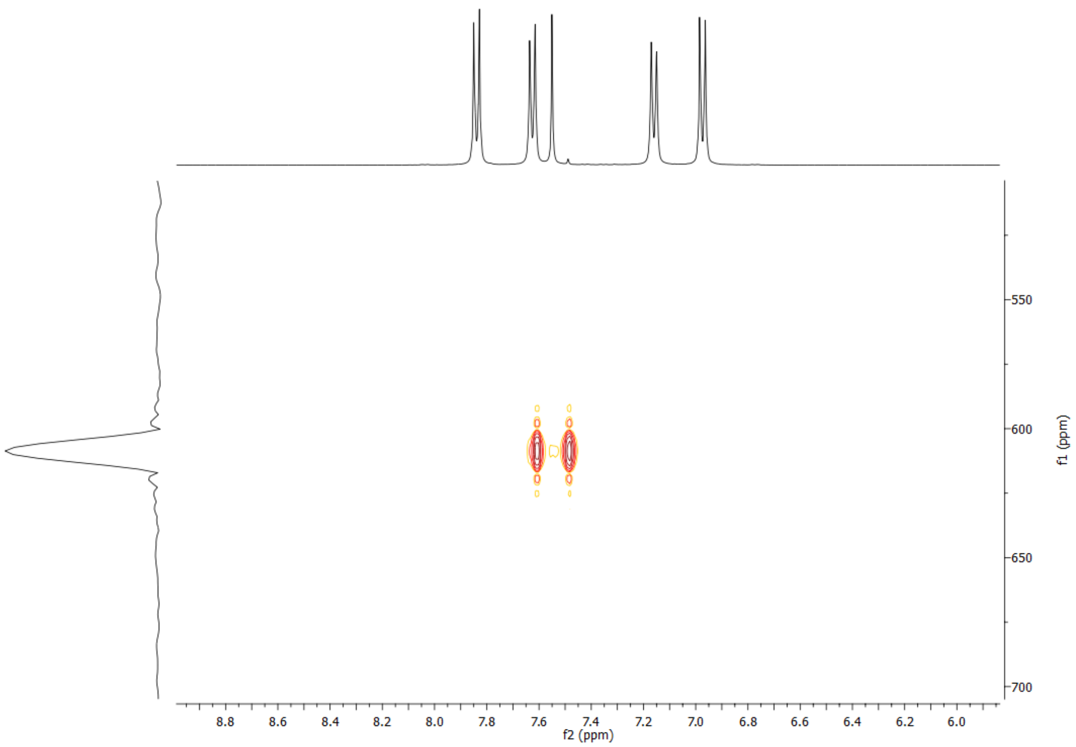

Figure S 41: ^{1}H-^{77}Se-HMBC (400 MHz, 76 MHz, DMSO-d6) of compound 13

Compund 14

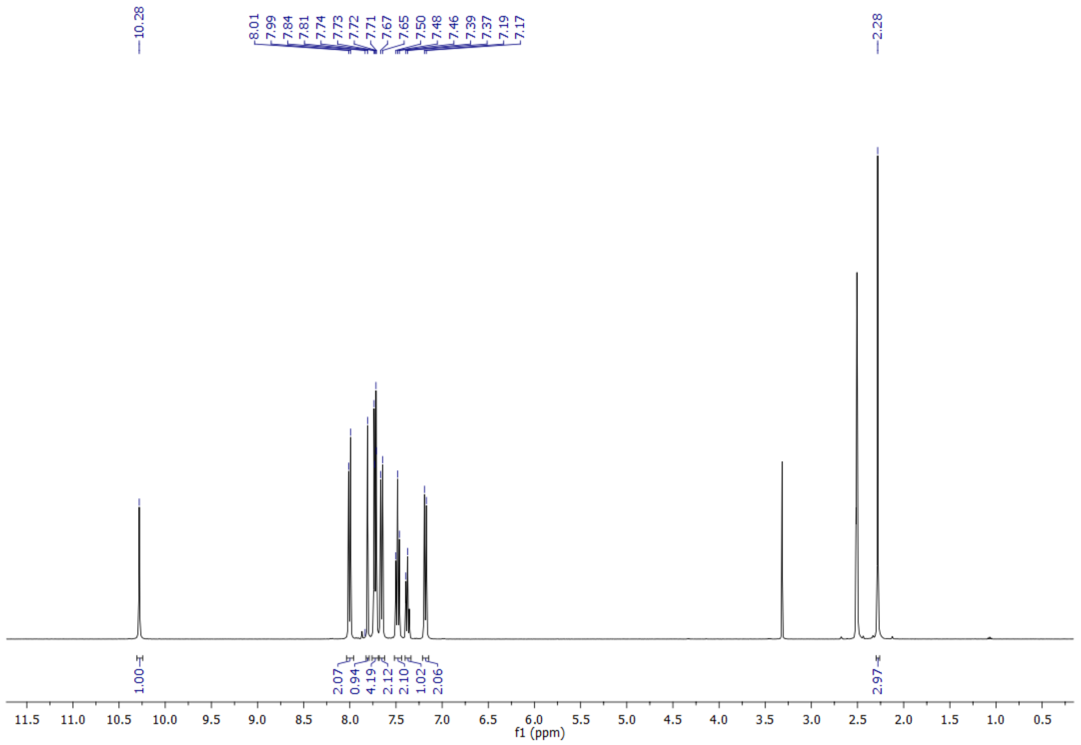

Figure S 42: ^{1}H-NMR (400 MHz, DMSO-d6) of compound 14

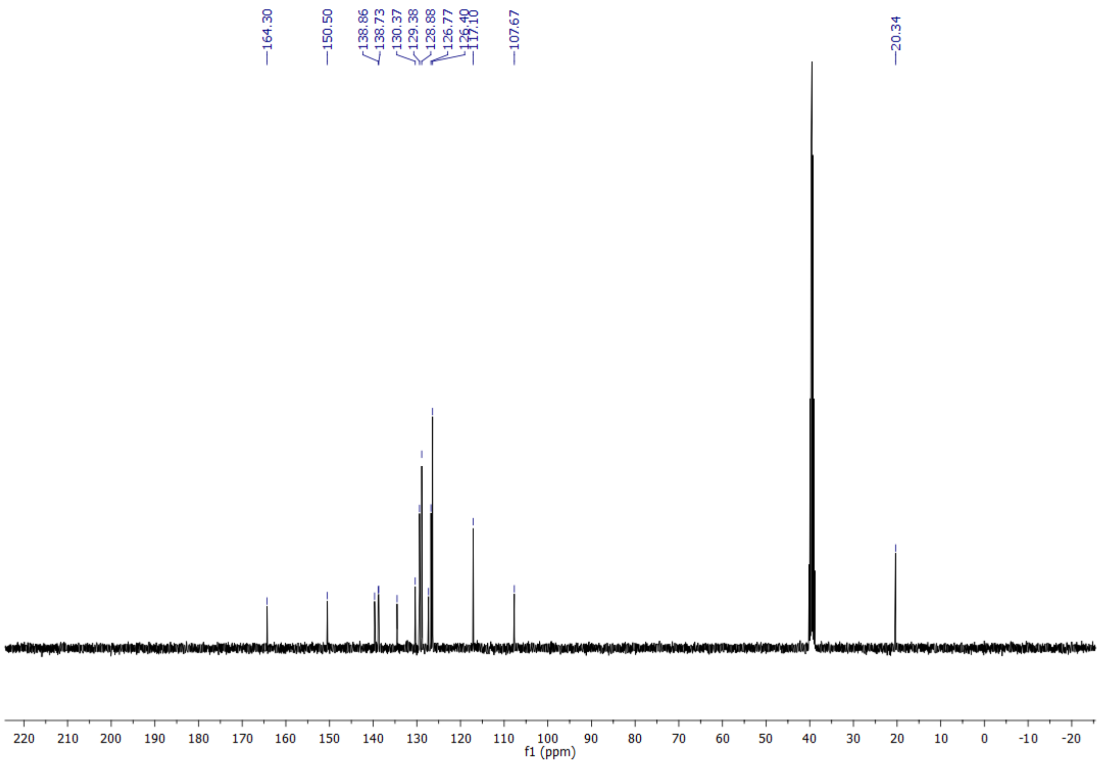

Figure S 43: ^{13}C-NMR (101 MHz, DMSO-d_6) of compound 14

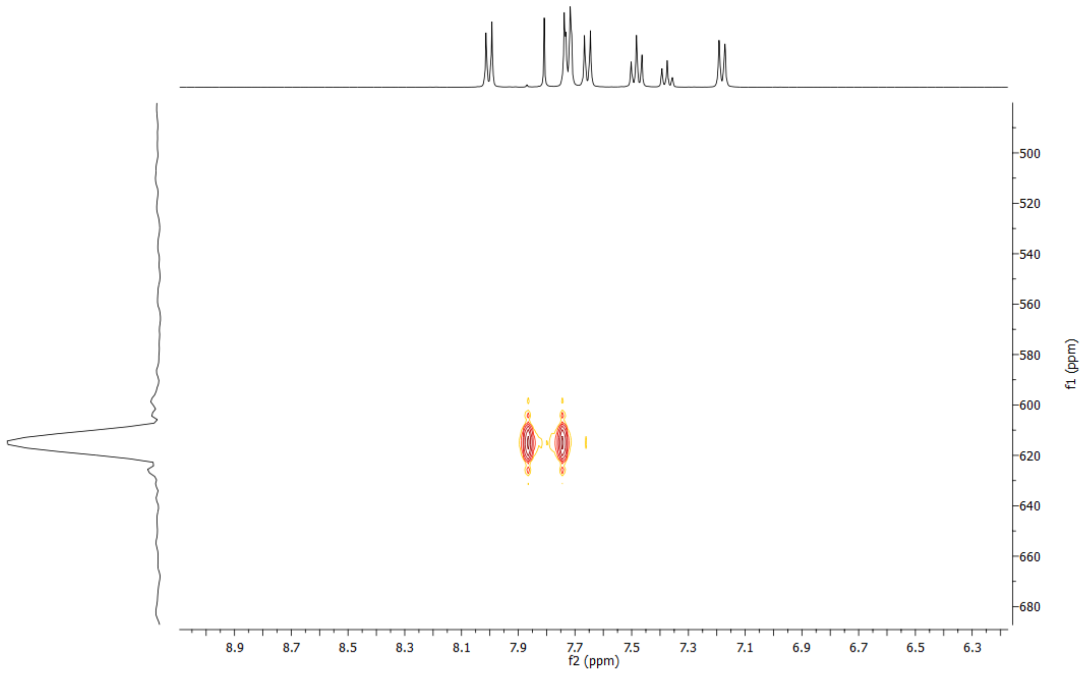

Figure S 44: ^{1}H-^{77}Se-HMBC (400 MHz, 76 MHz, DMSO-d_6) of compound 14

Compound 15

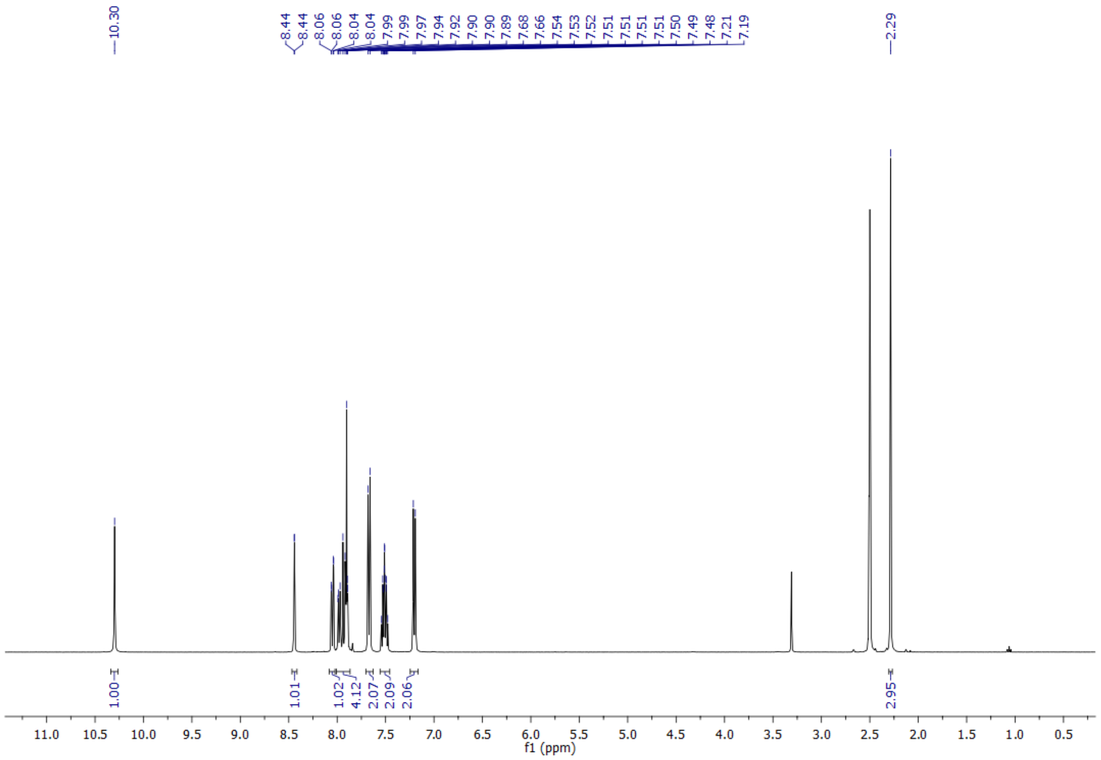

Figure S 45: ^{1}H-NMR (400 MHz, DMSO-d_6) of compound 15

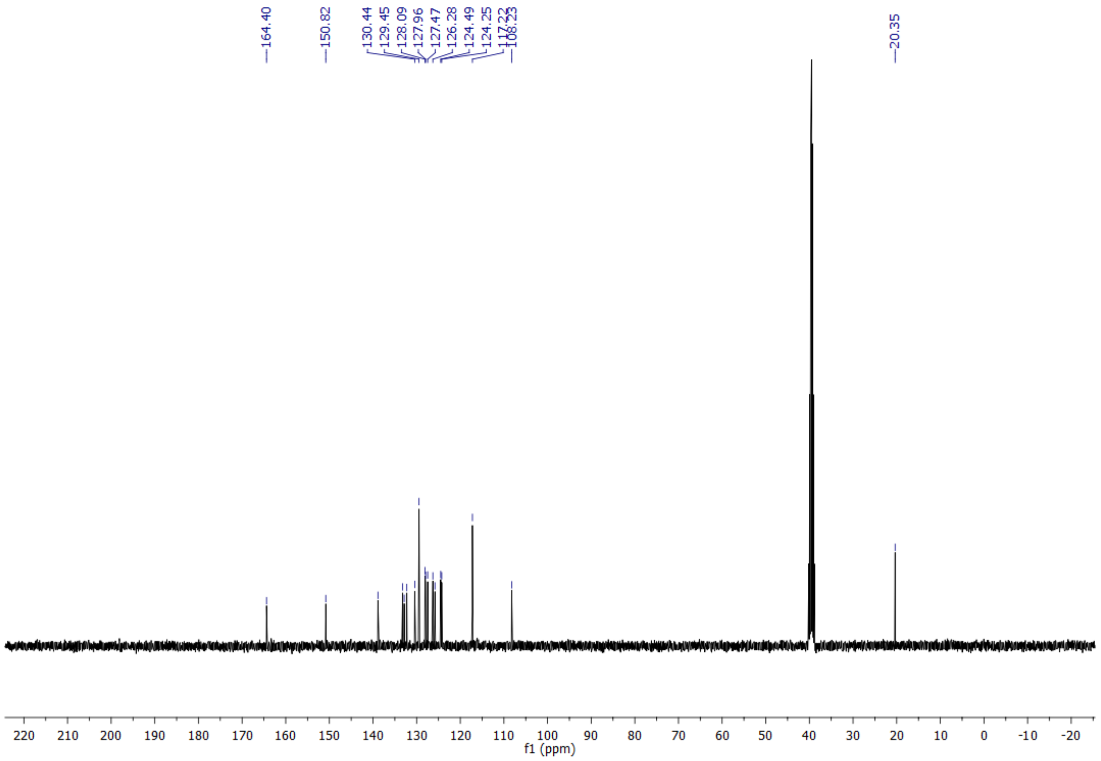

Figure S 46: ^{13}C-NMR (101 MHz, DMSO-d_6) of compound 15

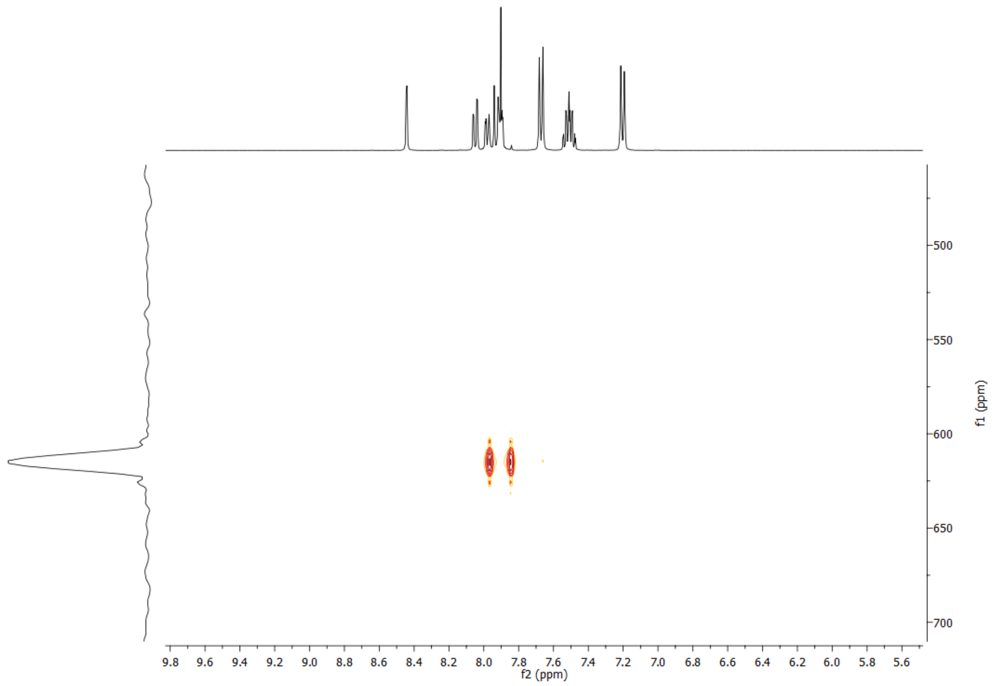

Figure S 47: ^{1}H-^{77}Se-HMBC (400 MHz, 76 MHz, DMSO-d_6) of compound 15

Compound 16

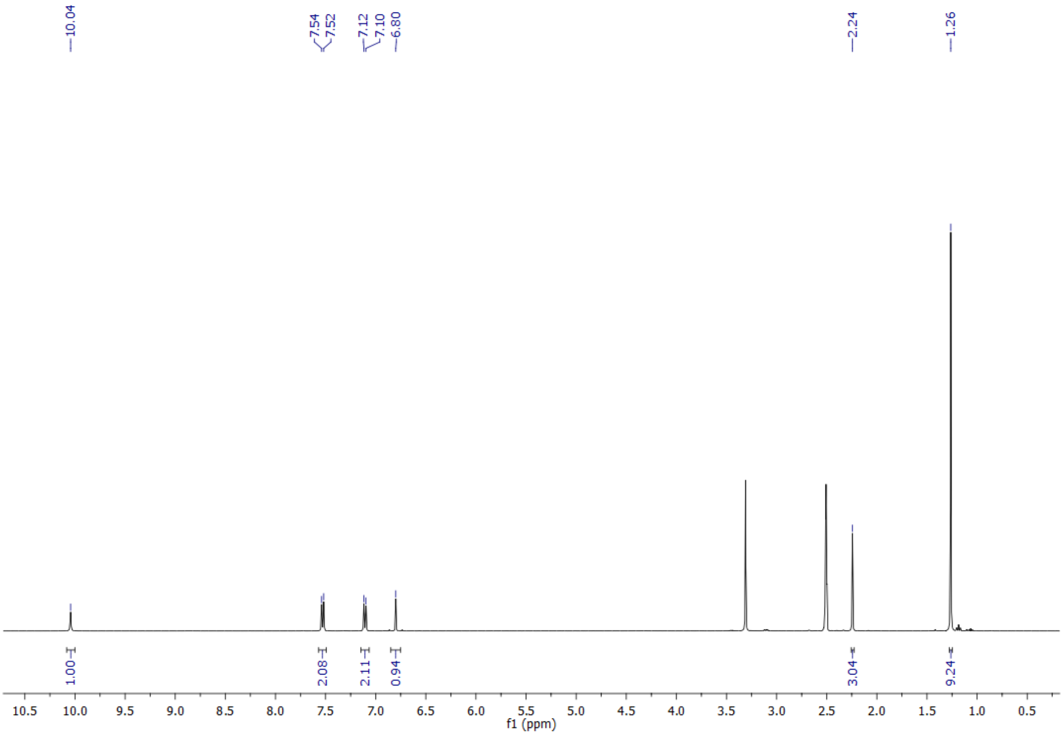

Figure S 48: ^{1}H-NMR (400 MHz, DMSO-d_6) of compound 16

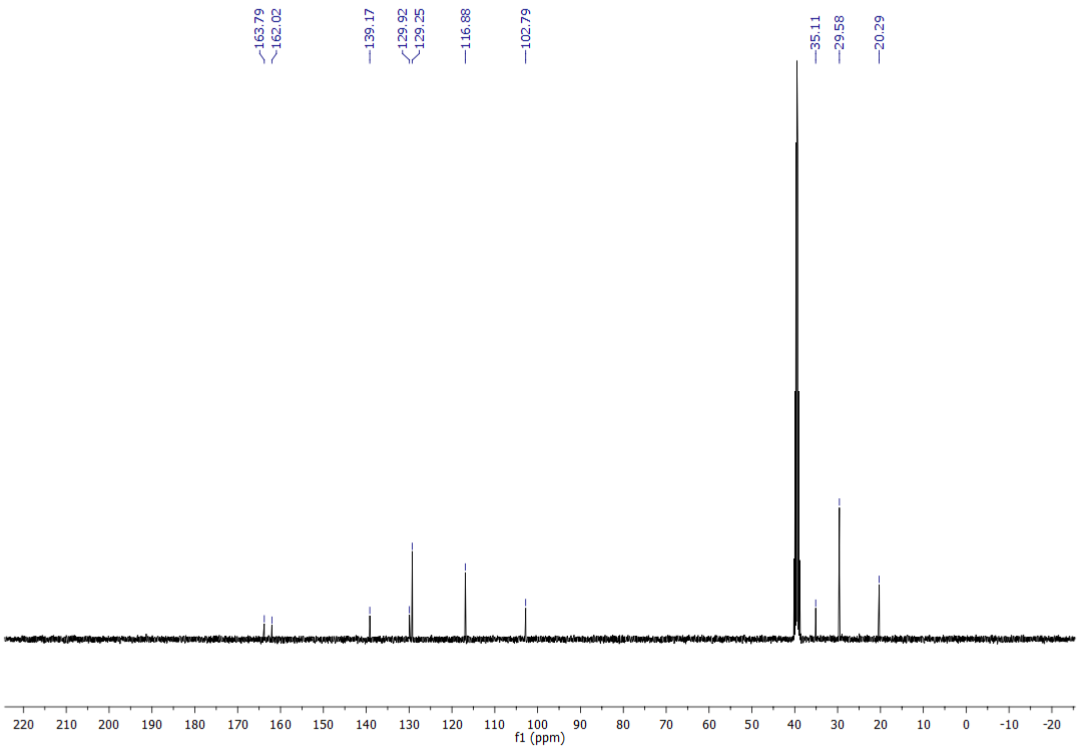

Figure S 49: ^{13}C-NMR (101 MHz, DMSO-d$_6$) of compound 16

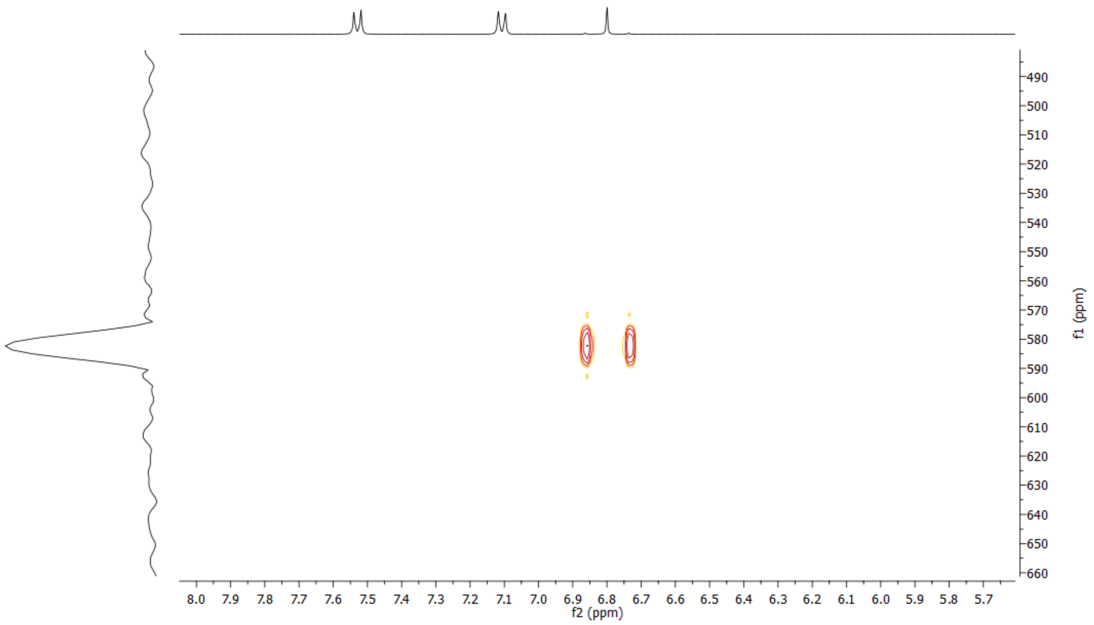

Figure S 50: ^{1}H-^{77}Se-HMBC (400 MHz, 76 MHz, DMSO-d$_6$) of compound 16

Compound 17

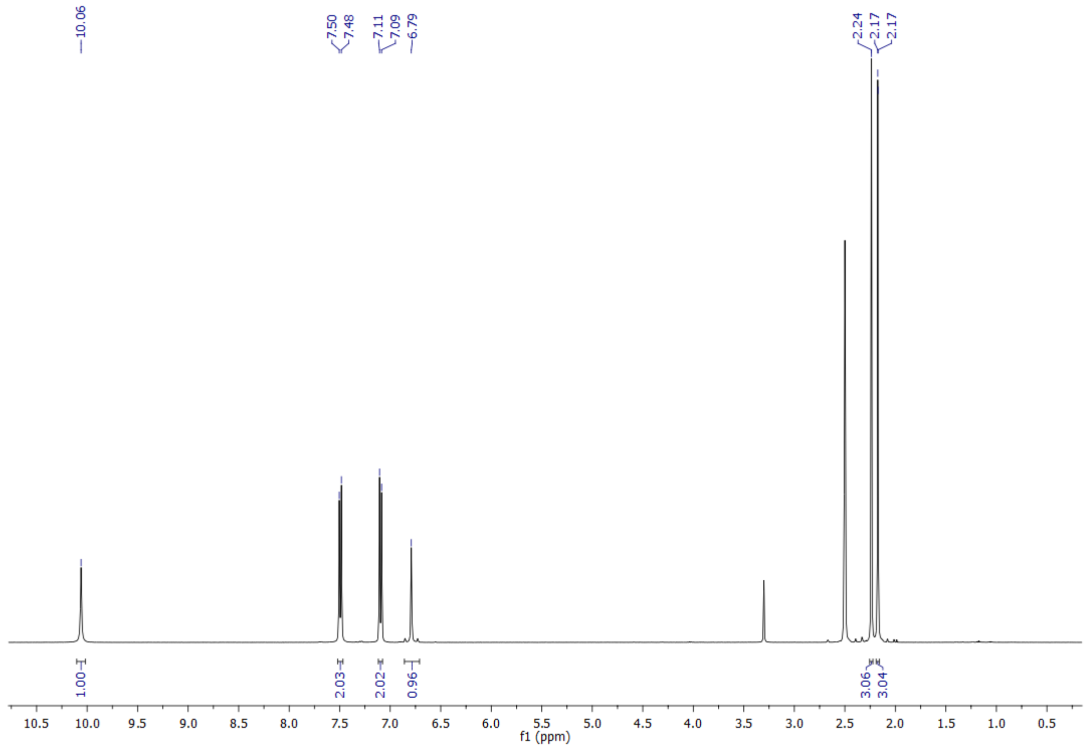

Figure S 51: ^{1}H-NMR (400 MHz, DMSO-d_6) of compound 17

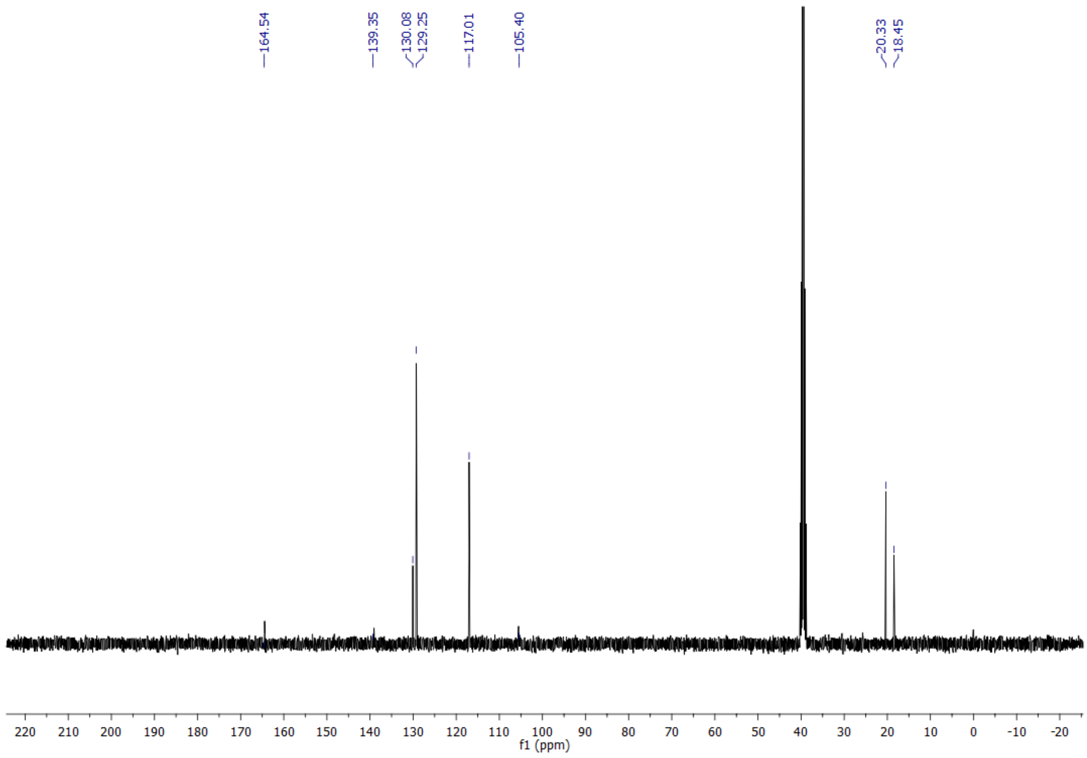

Figure S 52: ^{13}C-NMR (101 MHz, DMSO-d_6) of compound 17

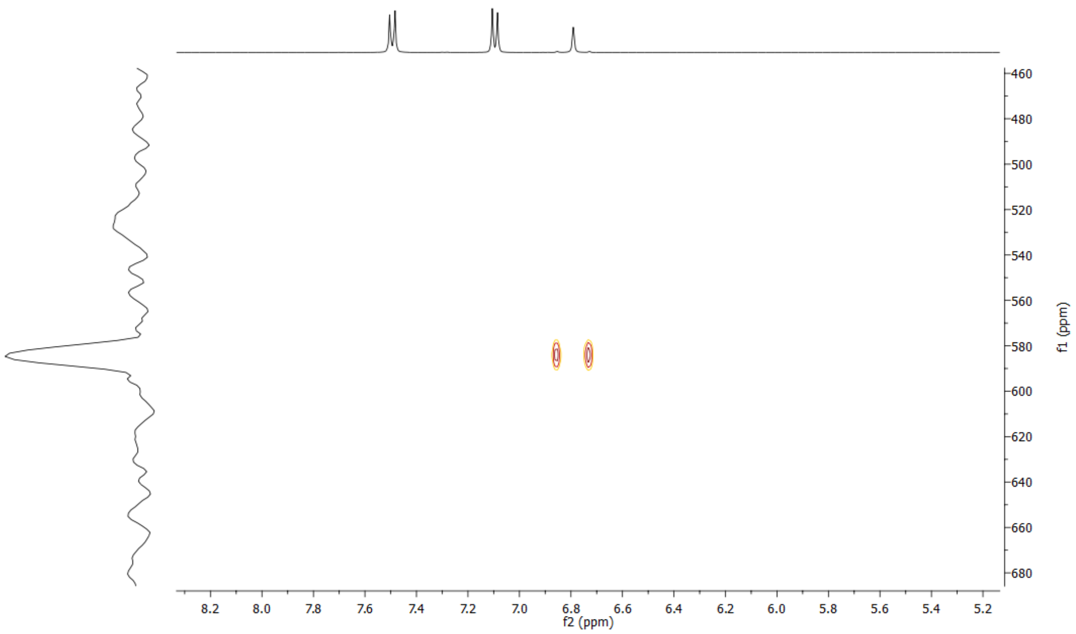

Figure S 53: ¹H-⁷⁷Se-HMBC (400 MHz, 76 MHz, DMSO-d₆) of compound 17

Compound 18

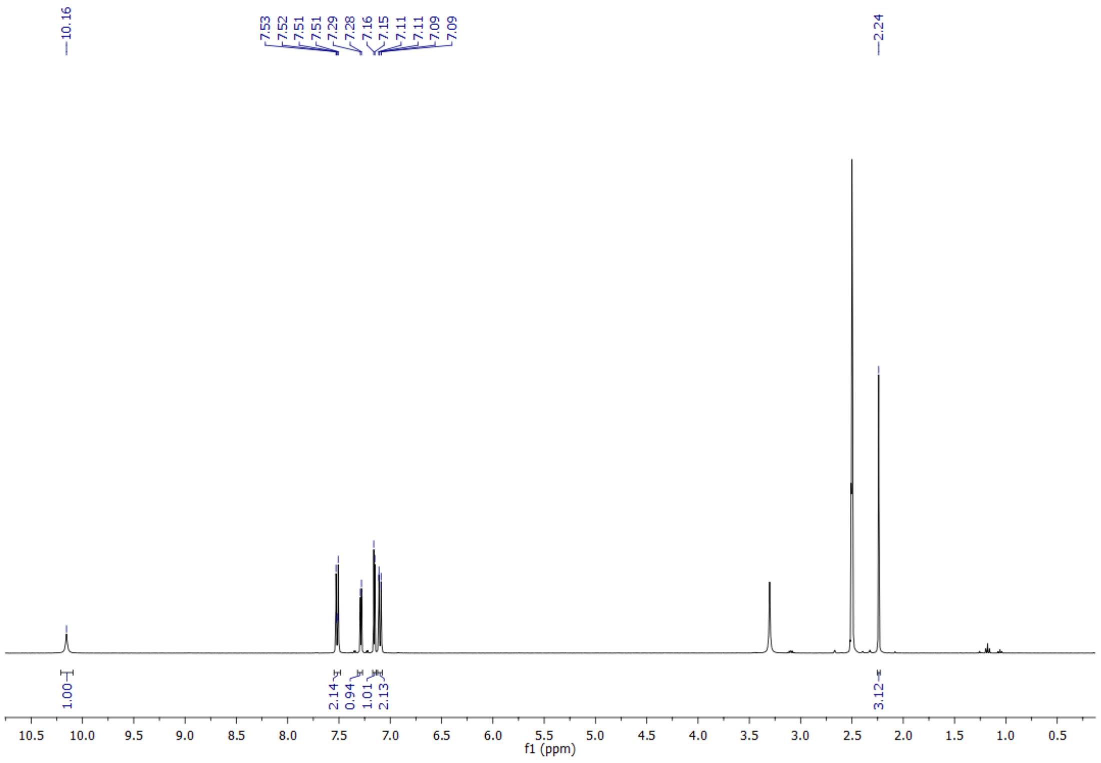

Figure S 54: ¹H-NMR (400 MHz, DMSO-d₆) of compound 18

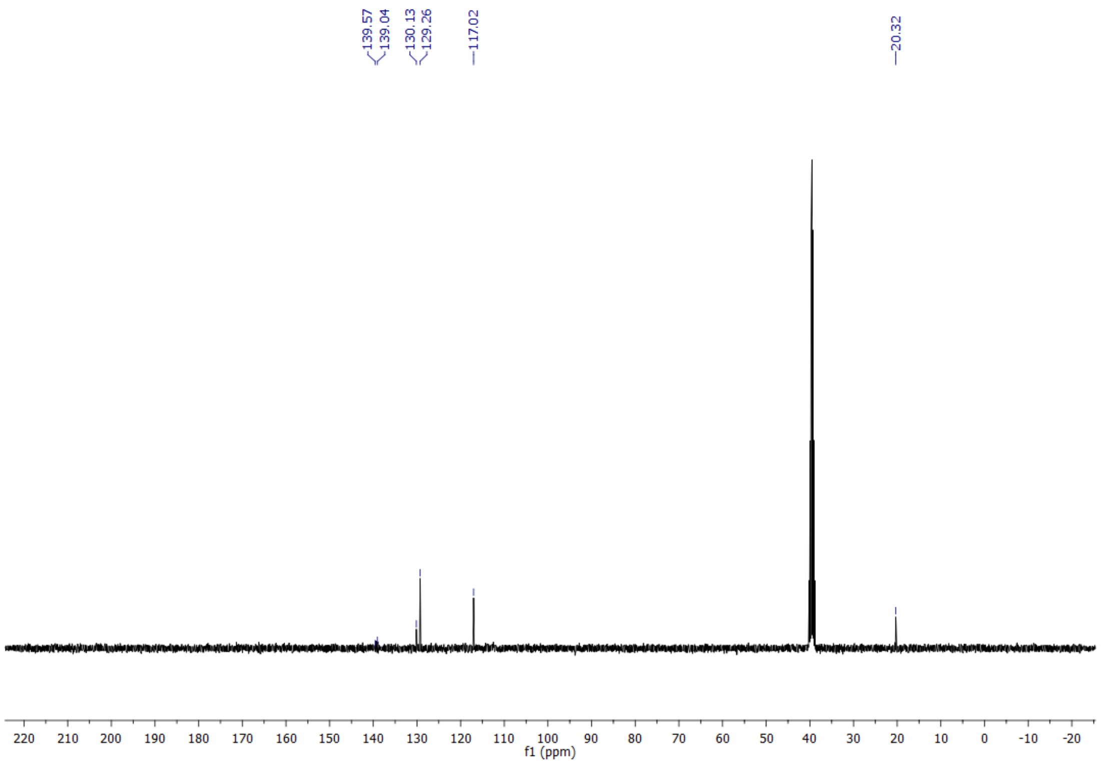

Figure S 55: ^{13}C-NMR (101 MHz, DMSO-d_6) of compound 18

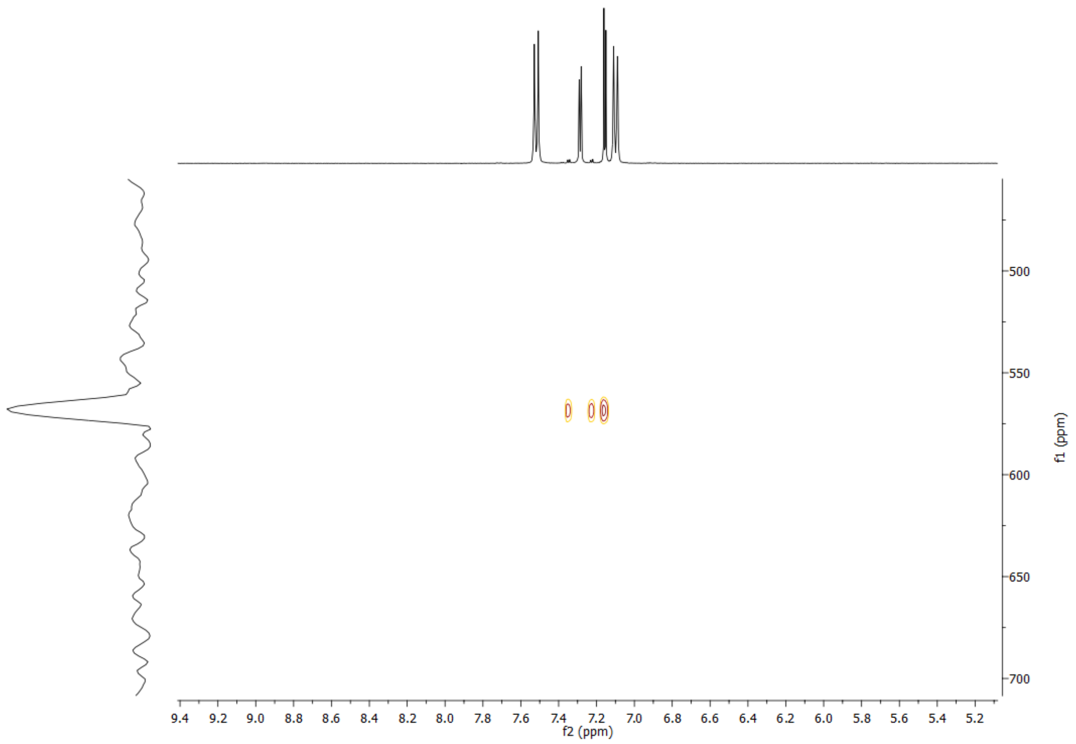

Figure S 56: ^{1}H-^{77}Se-HMBC (400 MHz, 76 MHz, DMSO-d_6) of compound 18

Compound 19

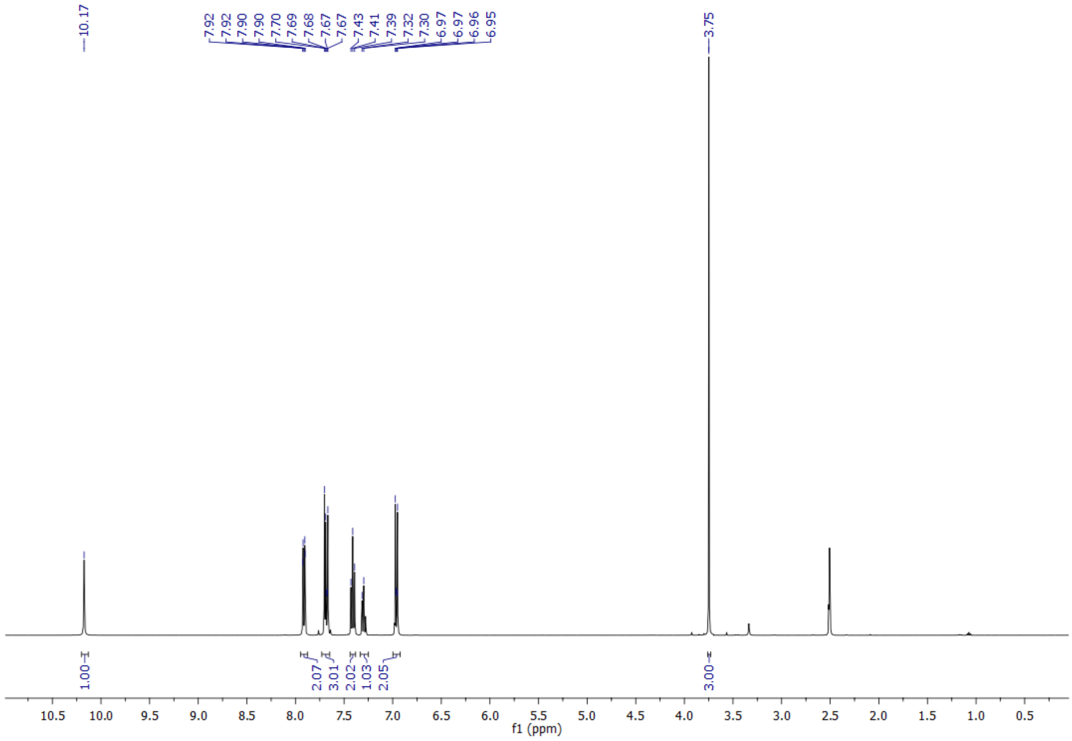

Figure S 57: ^{1}H-NMR (400 MHz, DMSO-d_6) of compound 19

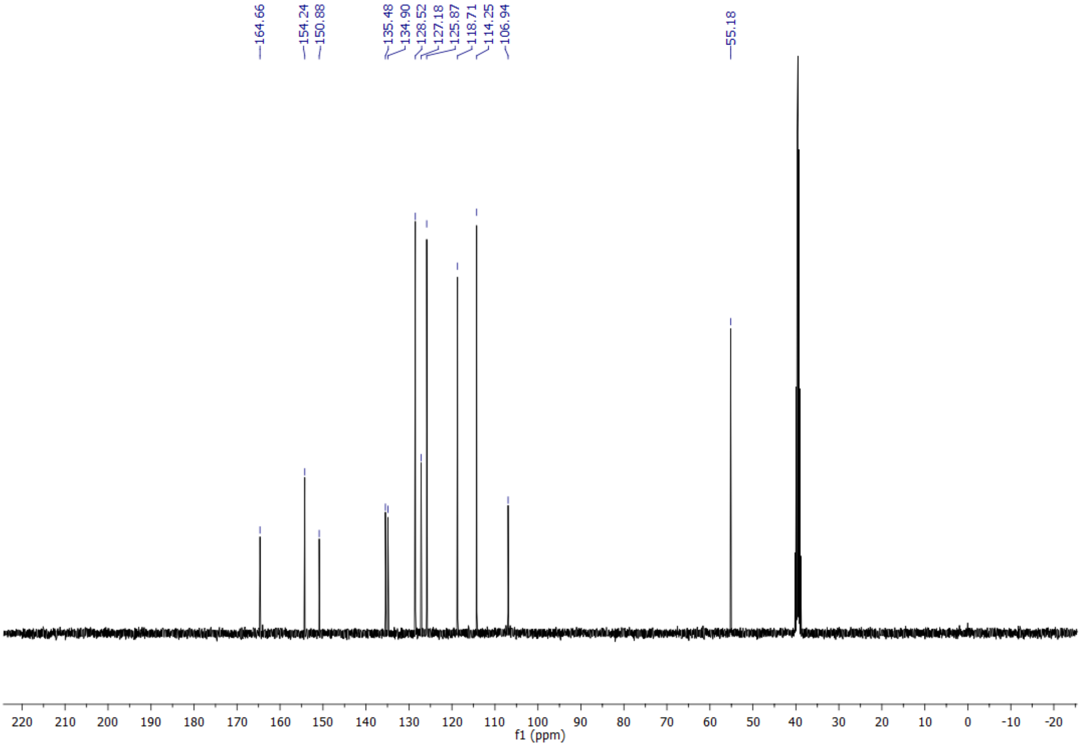

Figure S 58: ^{13}C-NMR (101 MHz, DMSO-d_6) of compound 19

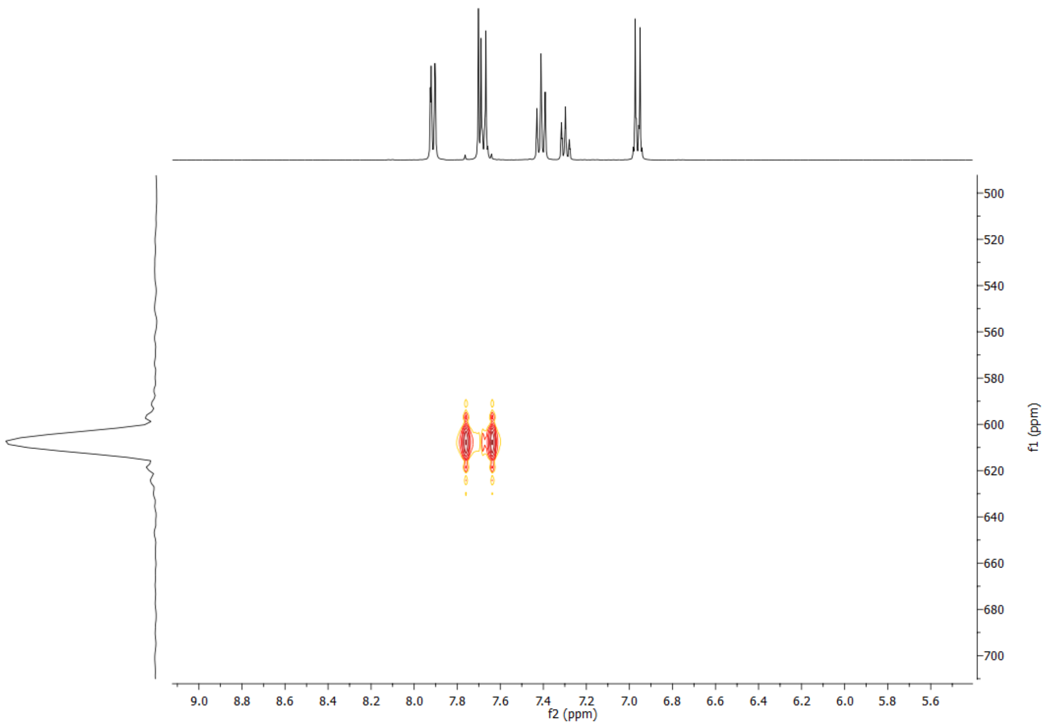

Figure S 59: ¹H-⁷⁷Se-HMBC (400 MHz, 76 MHz, DMSO-d₆) of compound 19

Compound 20

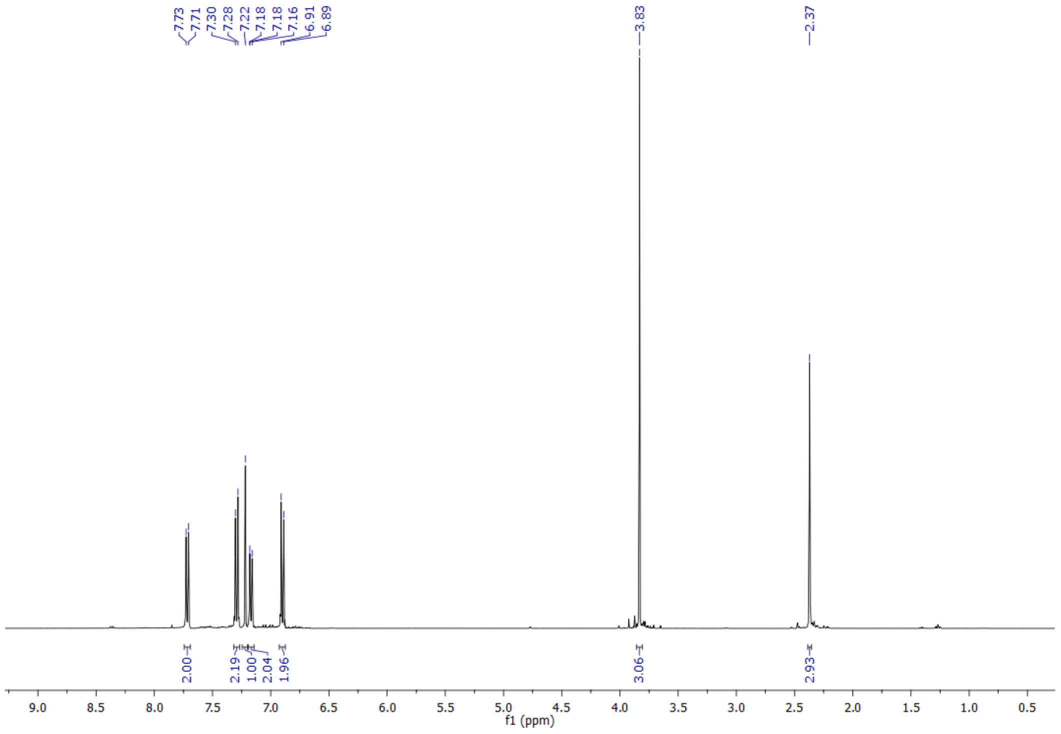

Figure S 60: ¹H-NMR (400 MHz, DMSO-d₆) of compound 20

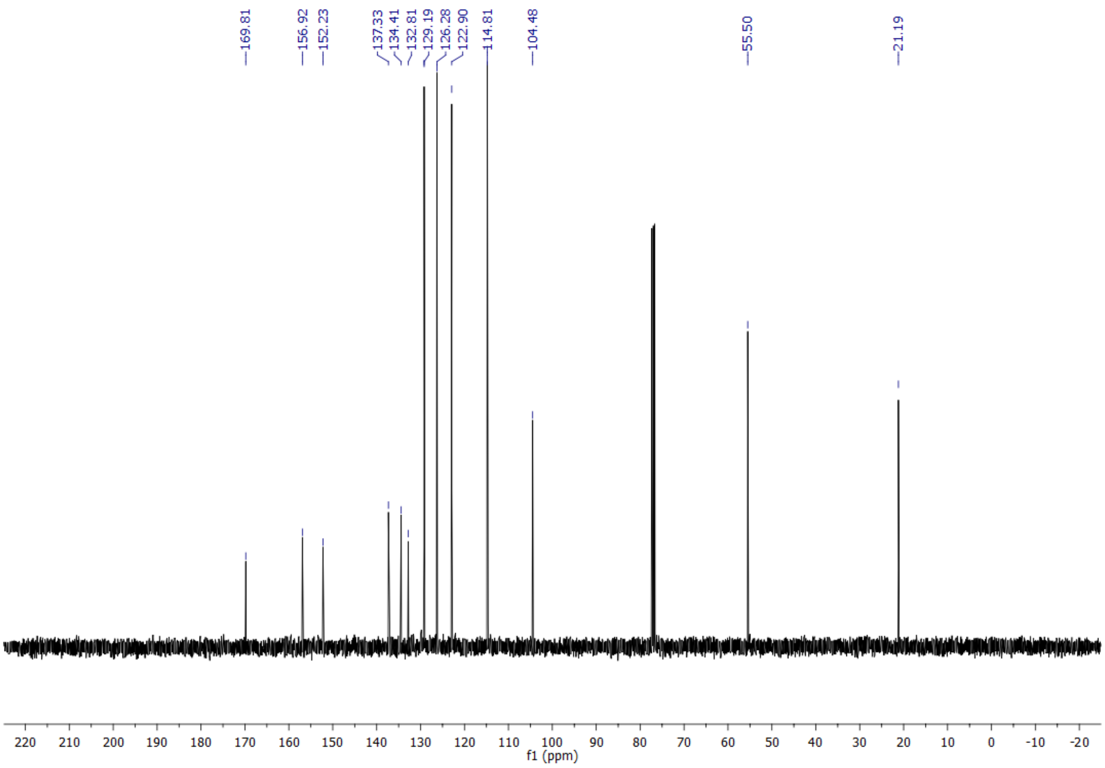

Figure S 61: ^{13}C-NMR (101 MHz, DMSO-d_6) of compound 20

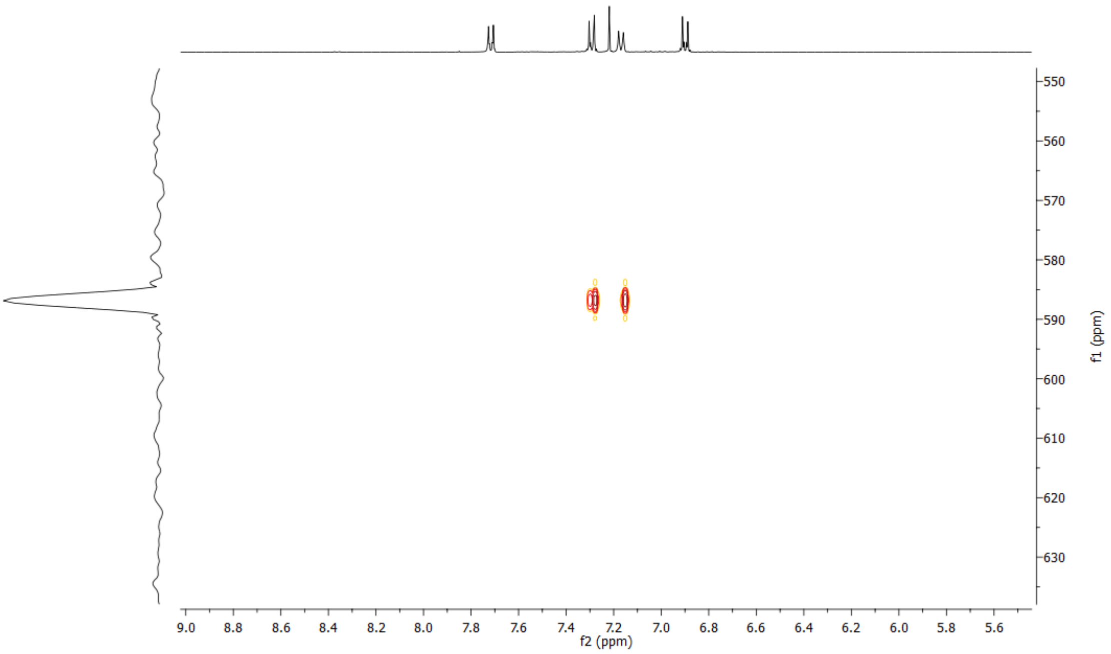

Figure S 62: ^{1}H-^{77}Se-HMBC (400 MHz, 76 MHz, DMSO-d_6) of compound 20

Compound 21

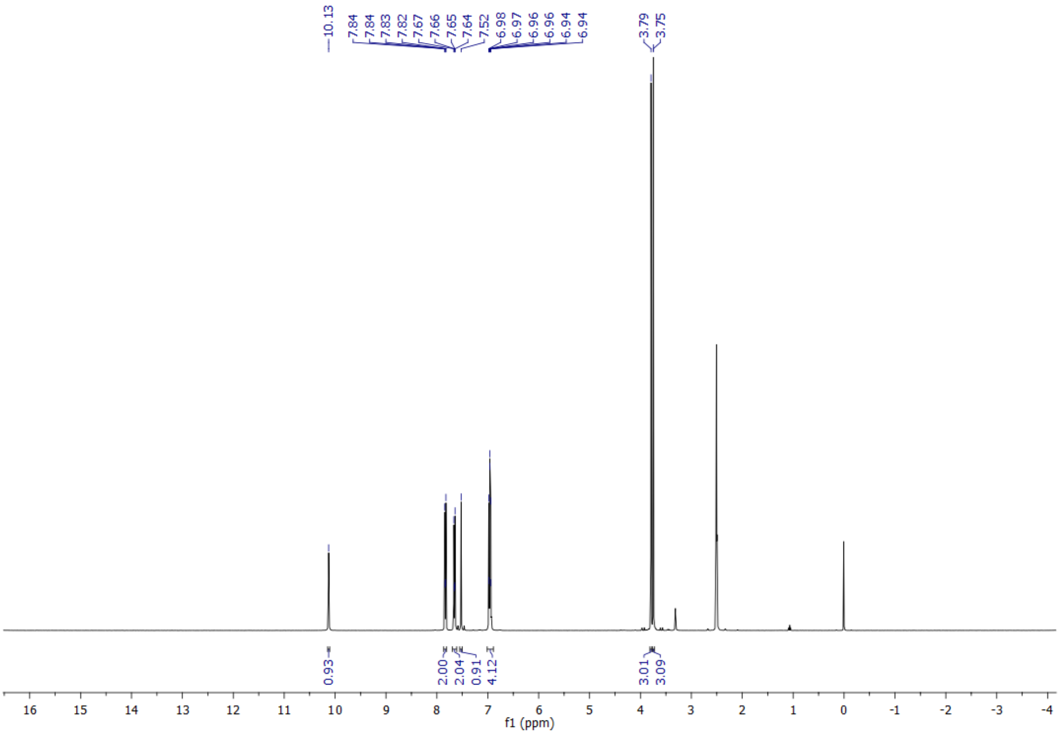

Figure S 63: ^{1}H-NMR (400 MHz, DMSO-d_6) of compound 21

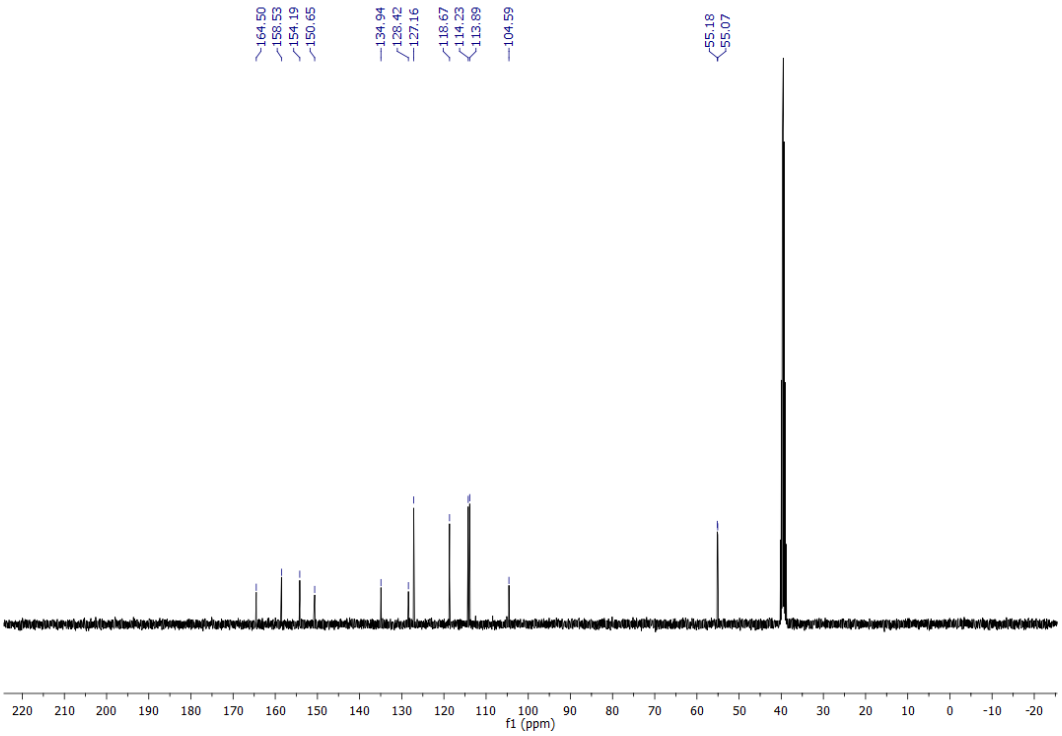

Figure S 64: ^{13}C-NMR (101 MHz, DMSO-d_6) of compound 21

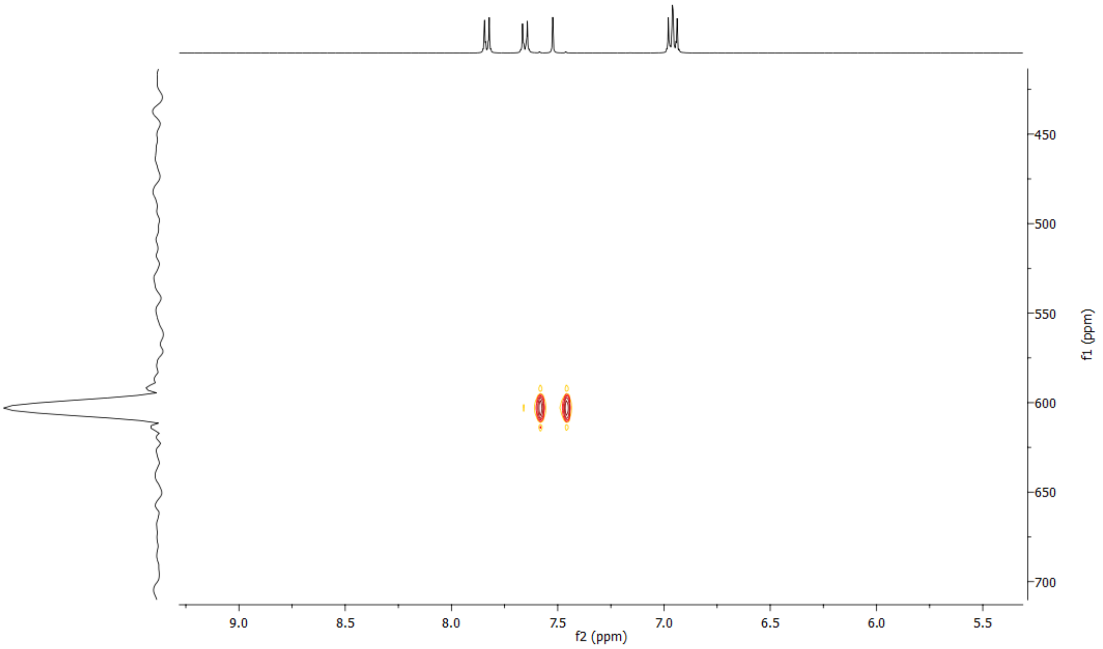

Figure S 65: ^{1}H-^{77}Se-HMBC (400 MHz, 76 MHz, DMSO-d_6) of compound 21

Compound 22

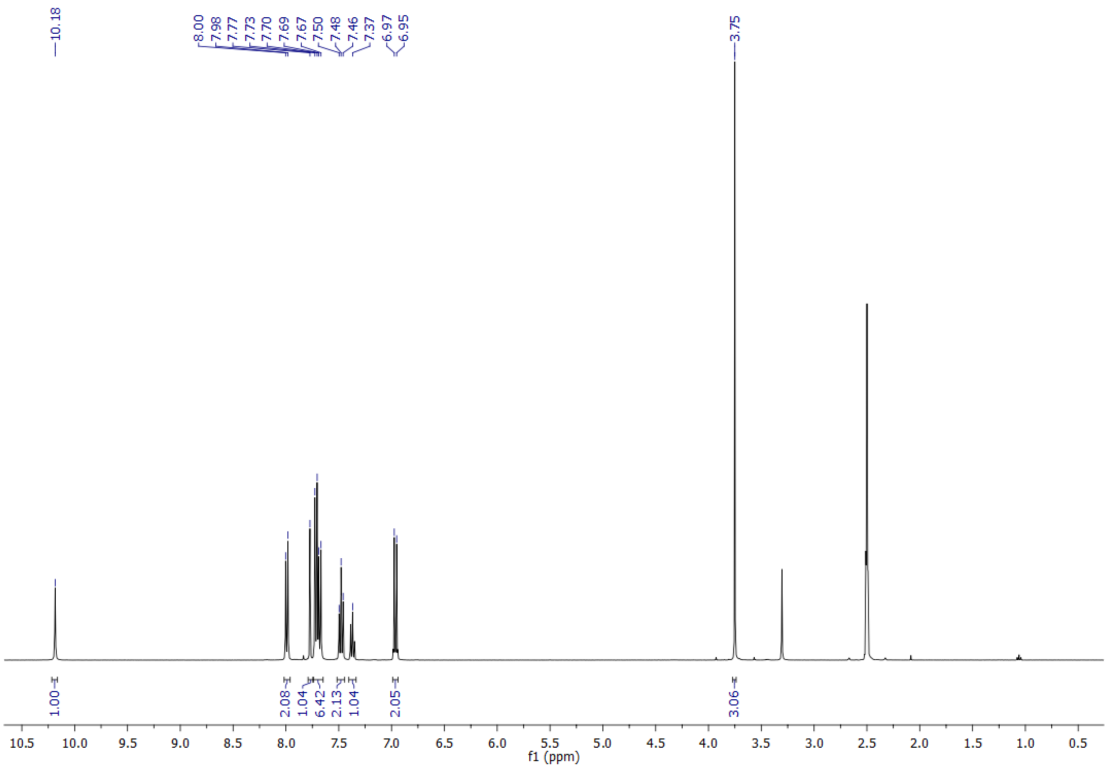

Figure S 66: ^{1}H-NMR (400 MHz, DMSO-d_6) of compound 22

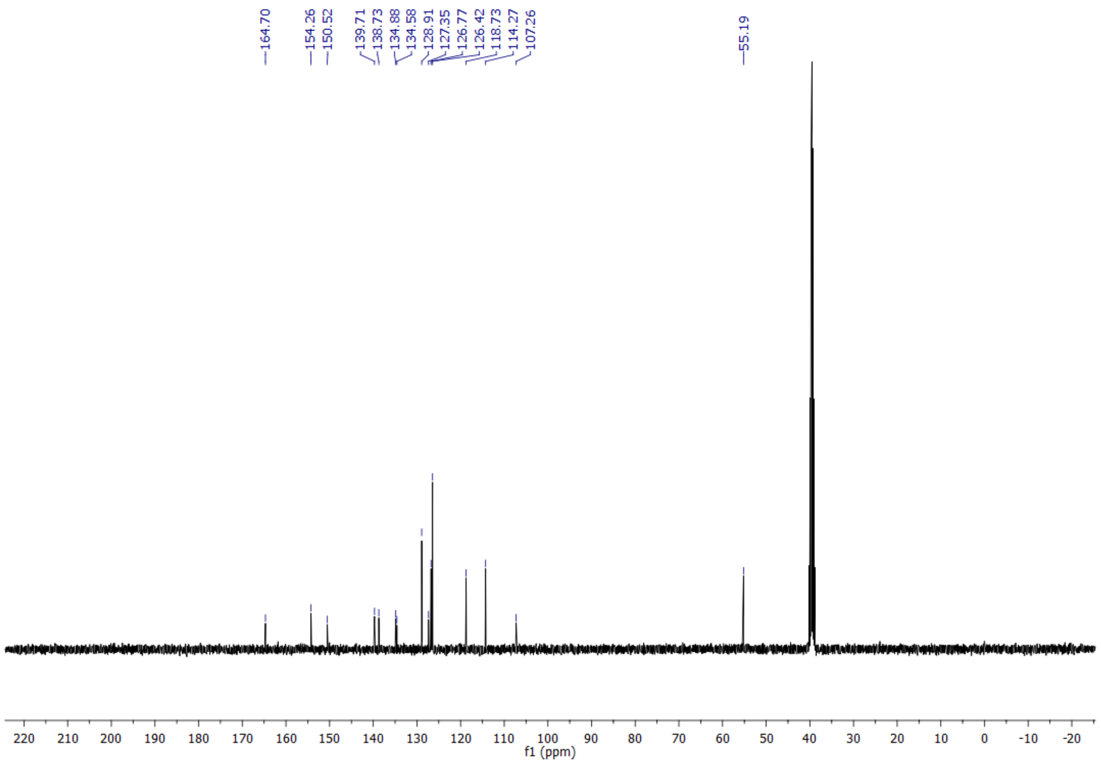

Figure S 67: ^{13}C-NMR (101 MHz, DMSO-d_6) of compound 22

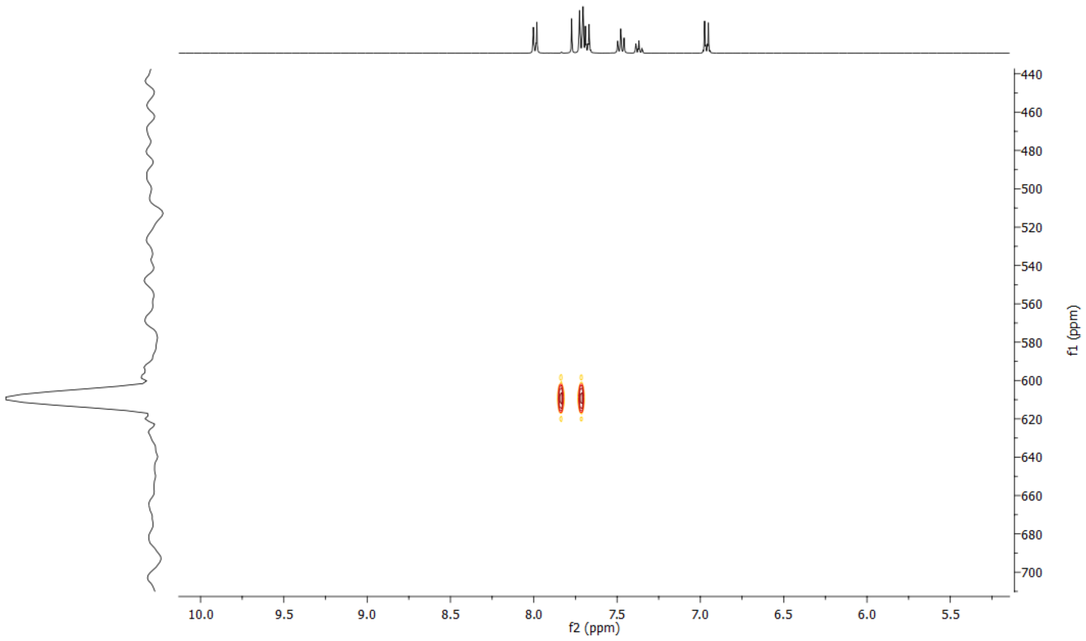

Figure S 68: ^{1}H-^{77}Se-HMBC (400 MHz, 76 MHz, DMSO-d_6) of compound 22

Compound 23

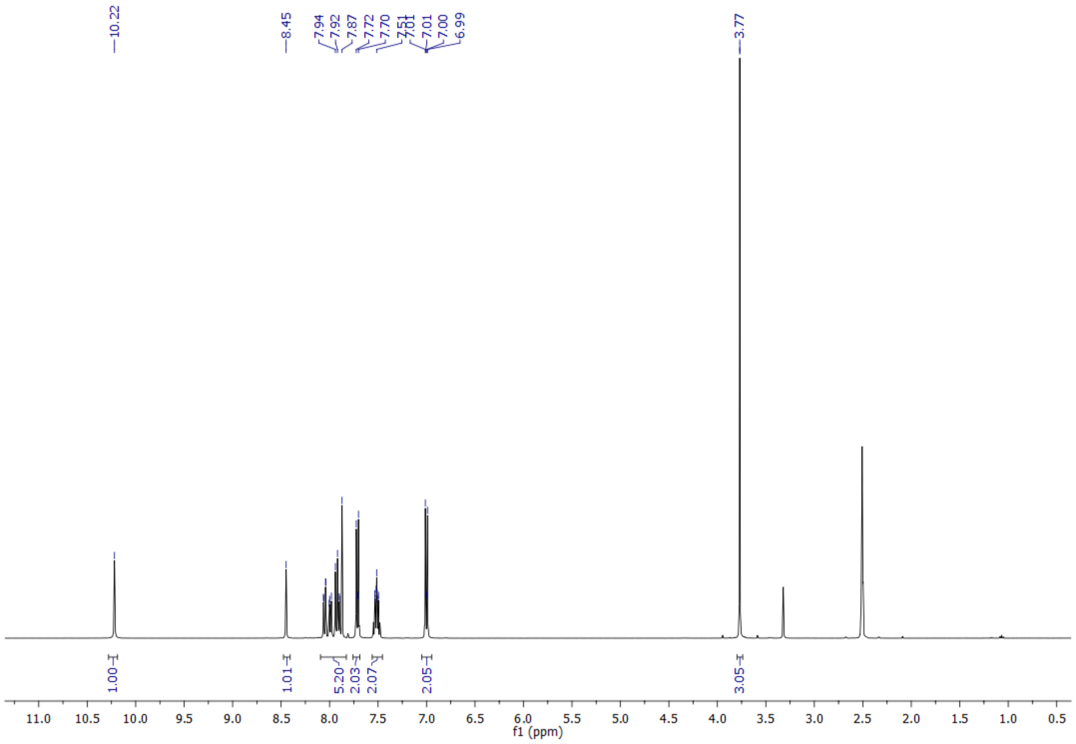

Figure S 69: ¹H-NMR (400 MHz, DMSO-d$_6$) of compound 23

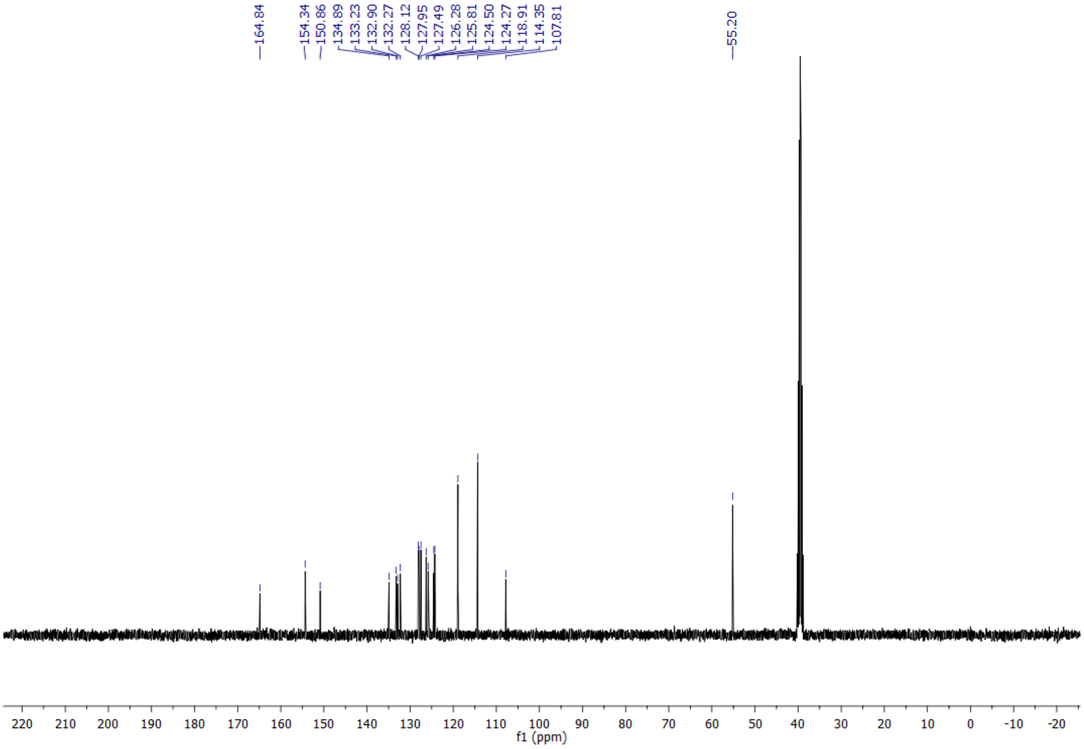

Figure S 70: ¹³C-NMR (101 MHz, DMSO-d$_6$) of compound 23

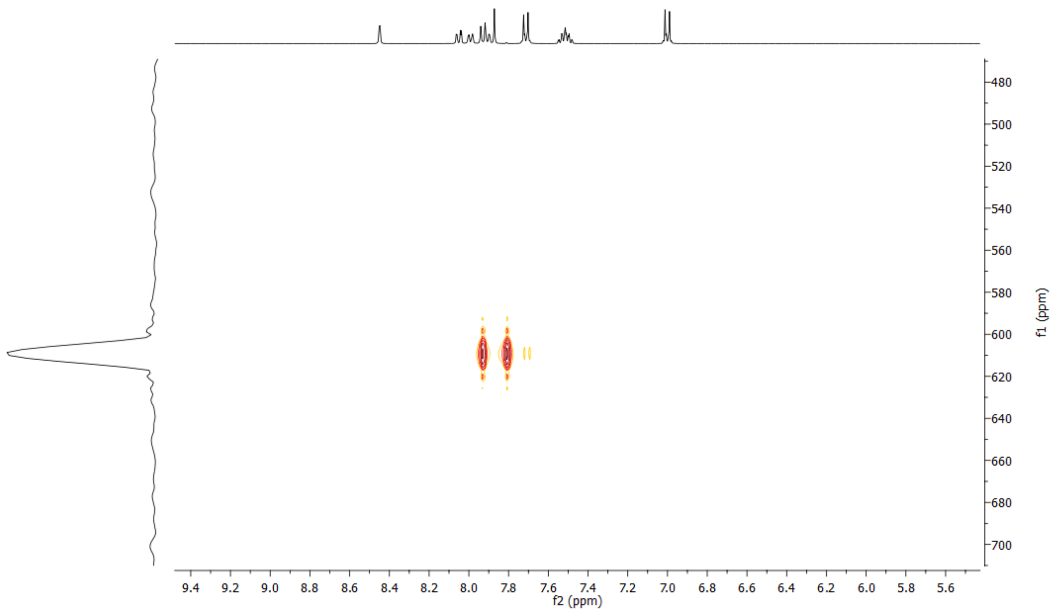

Figure S 71: ^{1}H-^{77}Se-HMBC (400 MHz, 76 MHz, DMSO-d_6) of compound 23

Compound 24

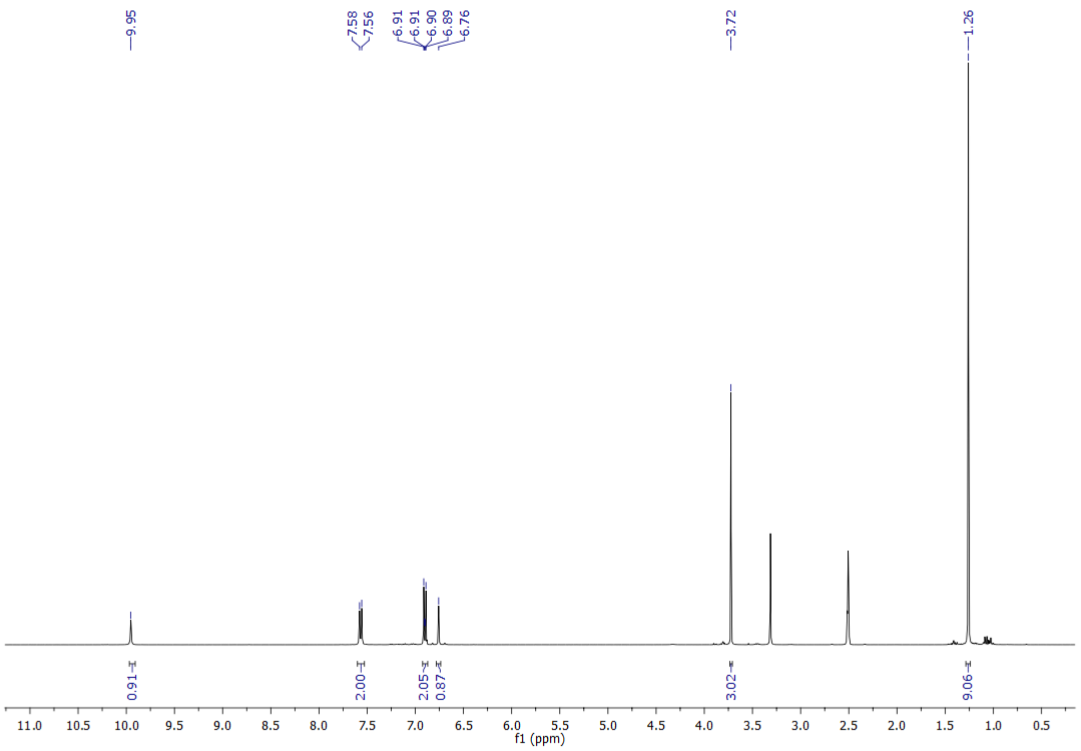

Figure S 72: ^{1}H-NMR (400 MHz, DMSO-d_6) of compound 24

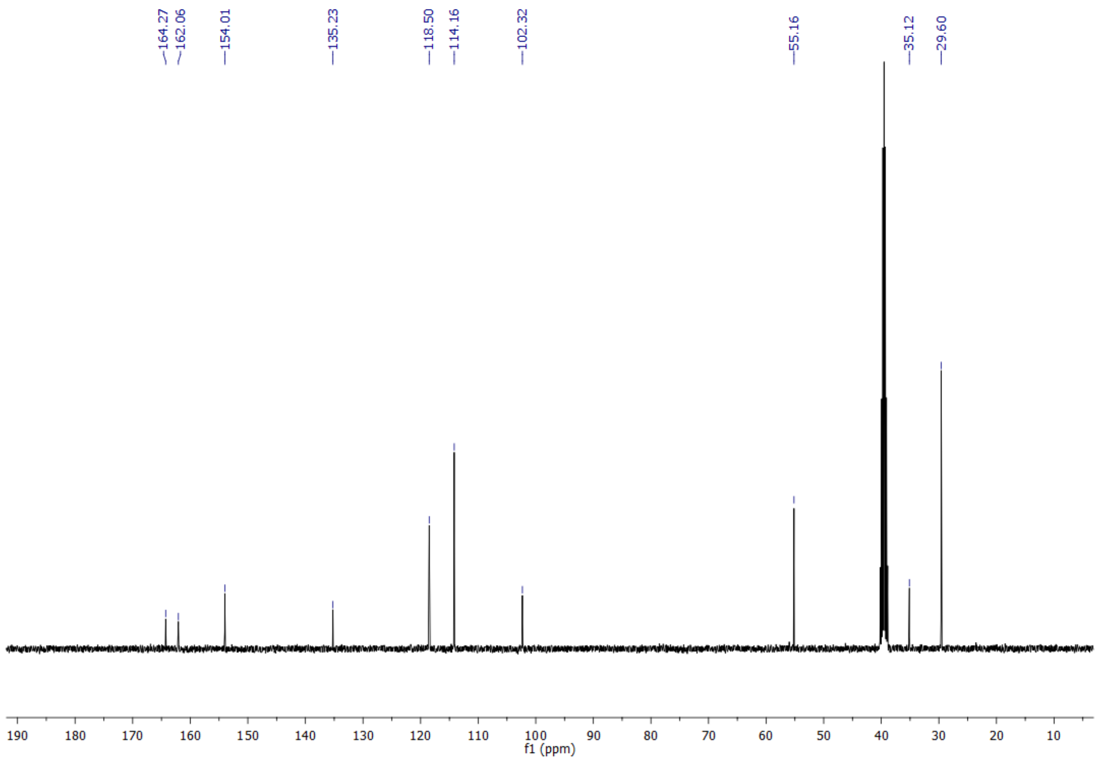

Figure S 73: ^{13}C-NMR (101 MHz, DMSO-d_6) of compound 24

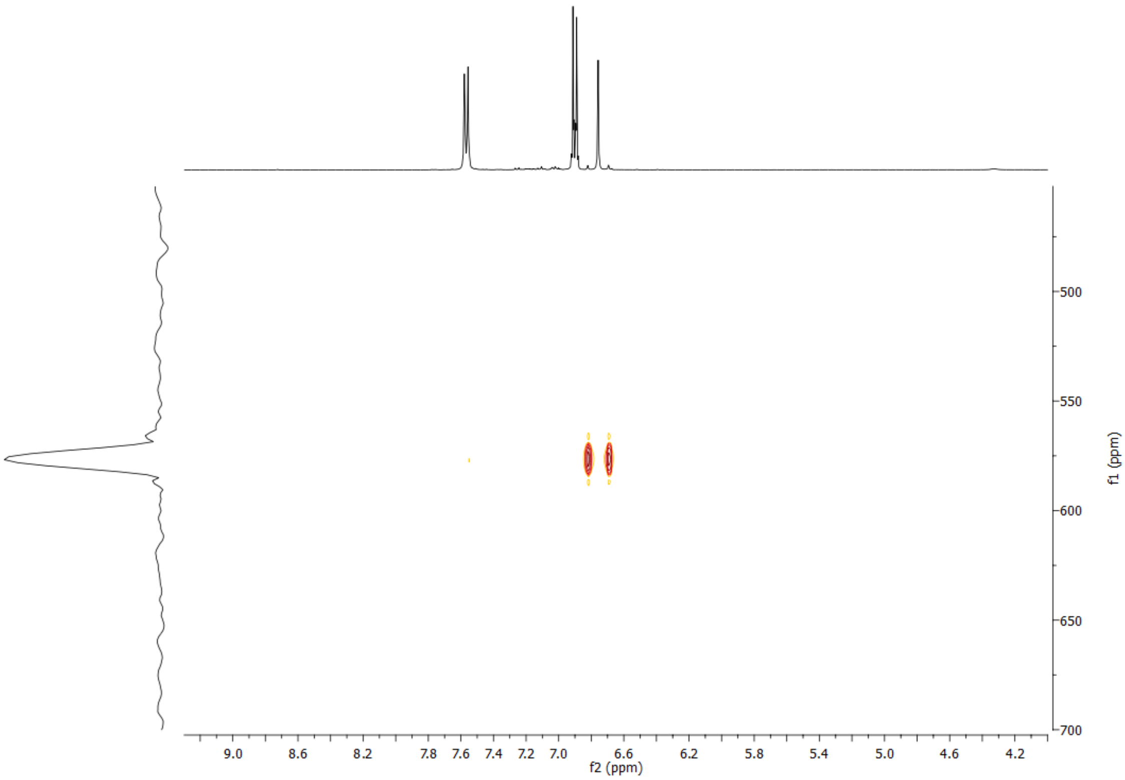

Figure S 74: ^{1}H-^{77}Se-HMBC (400 MHz, 76 MHz, DMSO-d_6) of compound 24

Compound 25

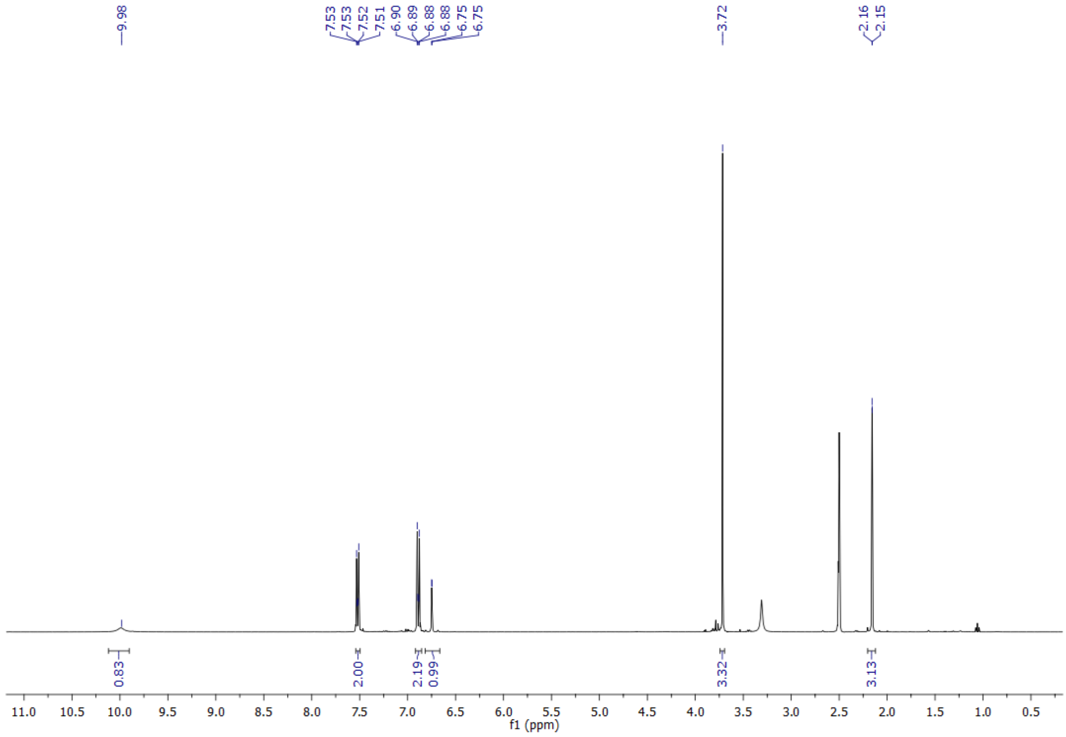

Figure S 75: ^{1}H-NMR (400 MHz, DMSO-d_6) of compound 25

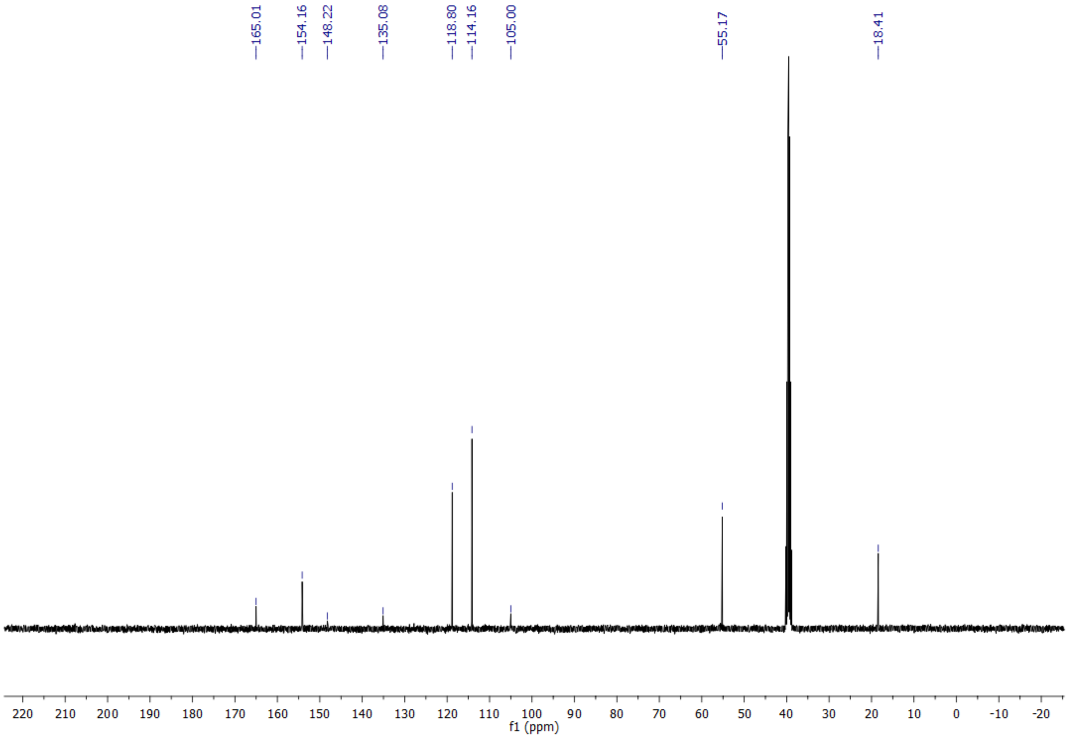

Figure S 76 : ^{13}C-NMR (101 MHz, DMSO-d_6) of compound 25

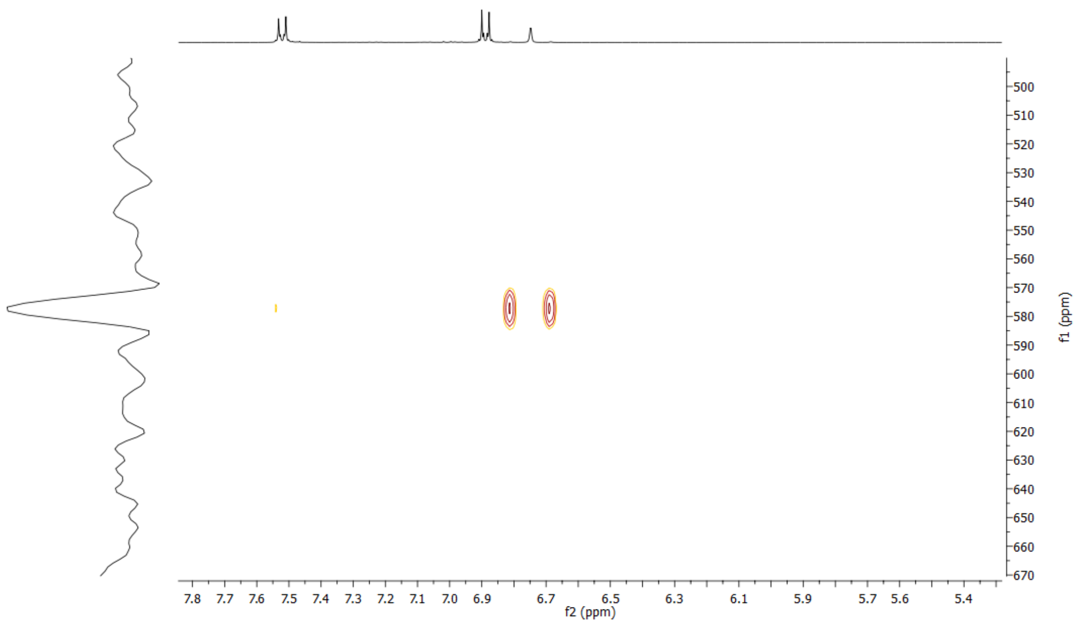

Figure S 77: ^{1}H-^{77}Se-HMBC (400 MHz, 76 MHz, DMSO-d_6) of compound 25

Compound 26

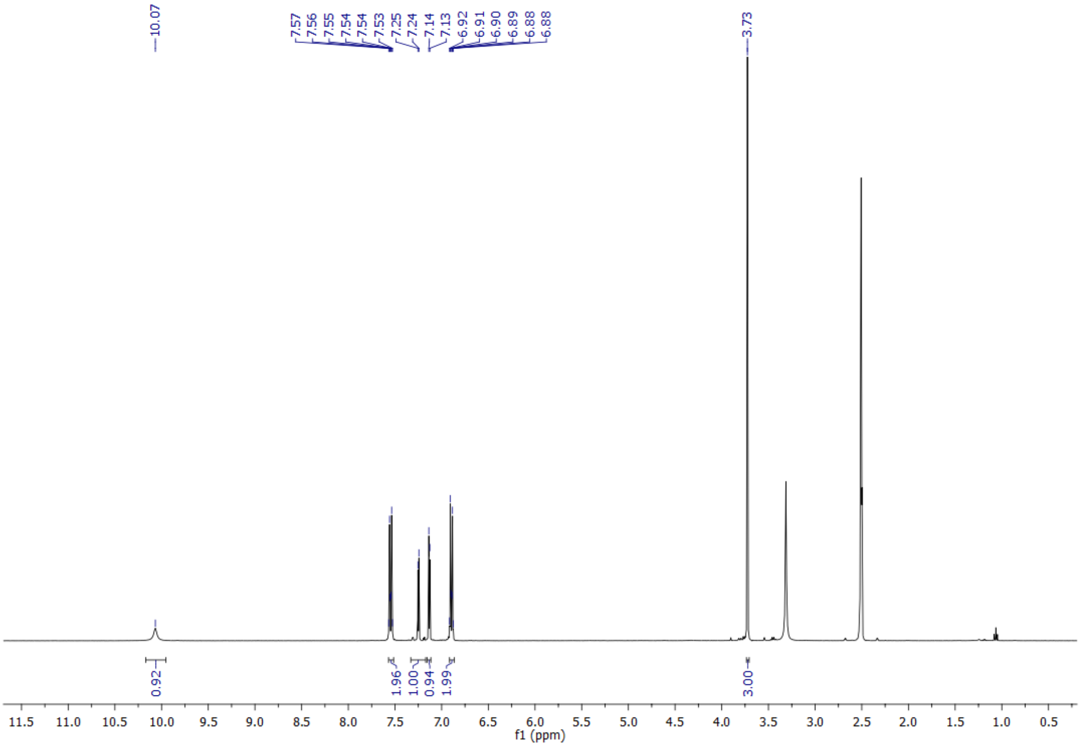

Figure S 78: ^{1}H-NMR (400 MHz, DMSO-d_6) of compound 26

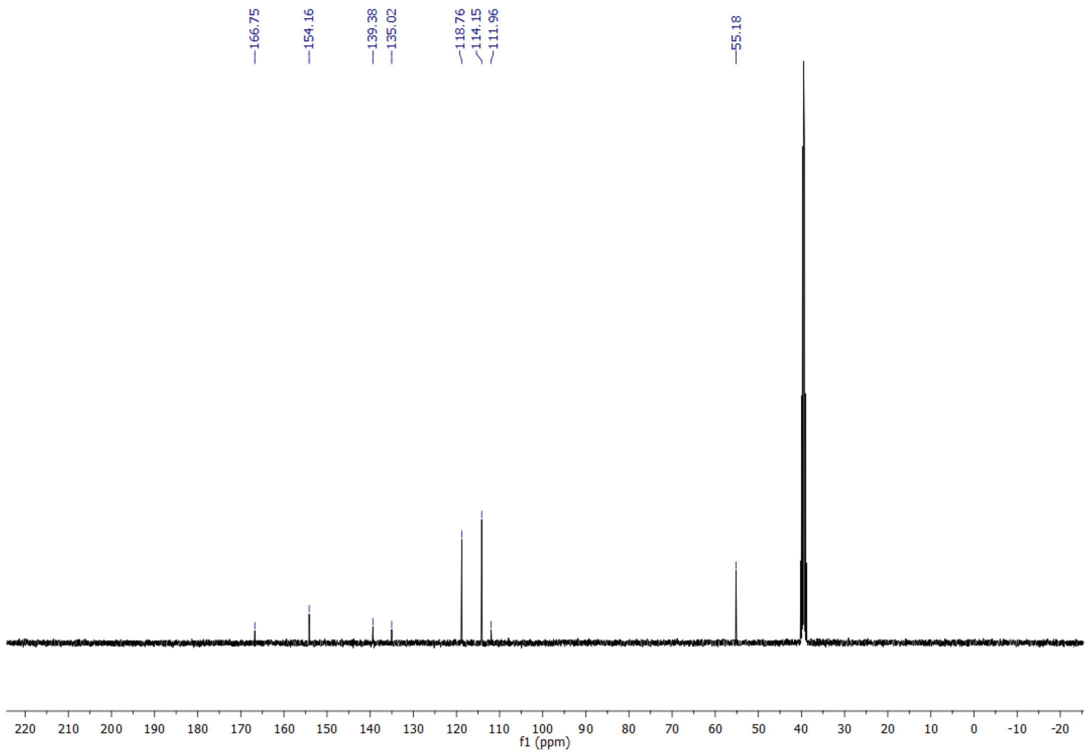

Figure S 79: ^{13}C-NMR (101 MHz, DMSO-d_6) of compound 26

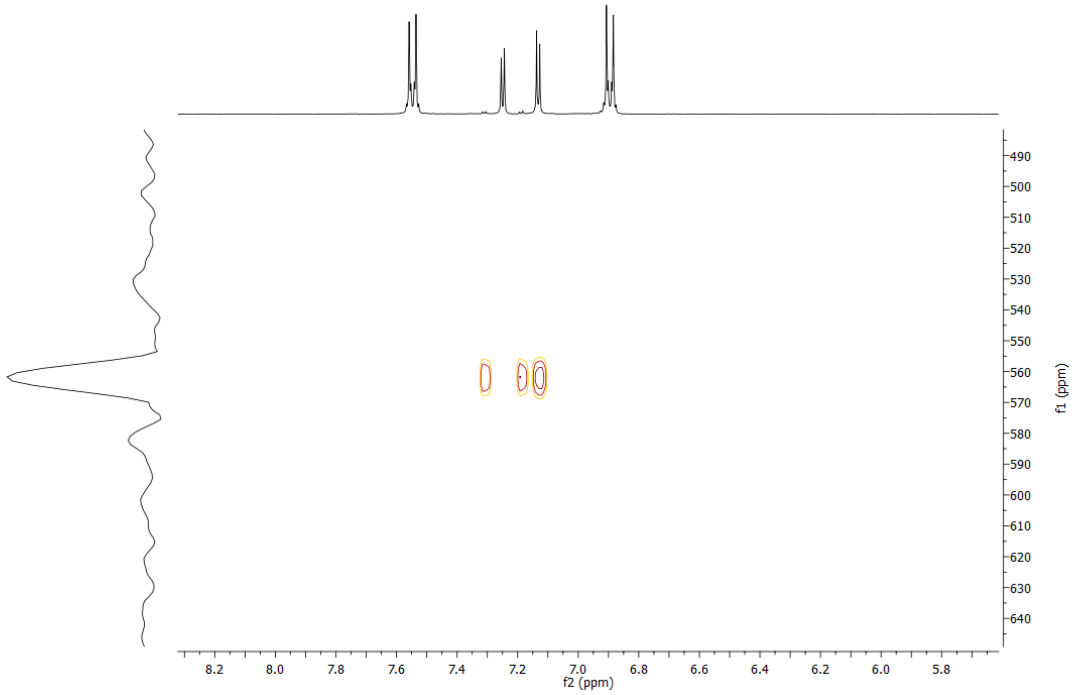

Figure S 80: ^{1}H-^{77}Se-HMBC (400 MHz, 76 MHz, DMSO-d_6) of compound 26

Compound 27

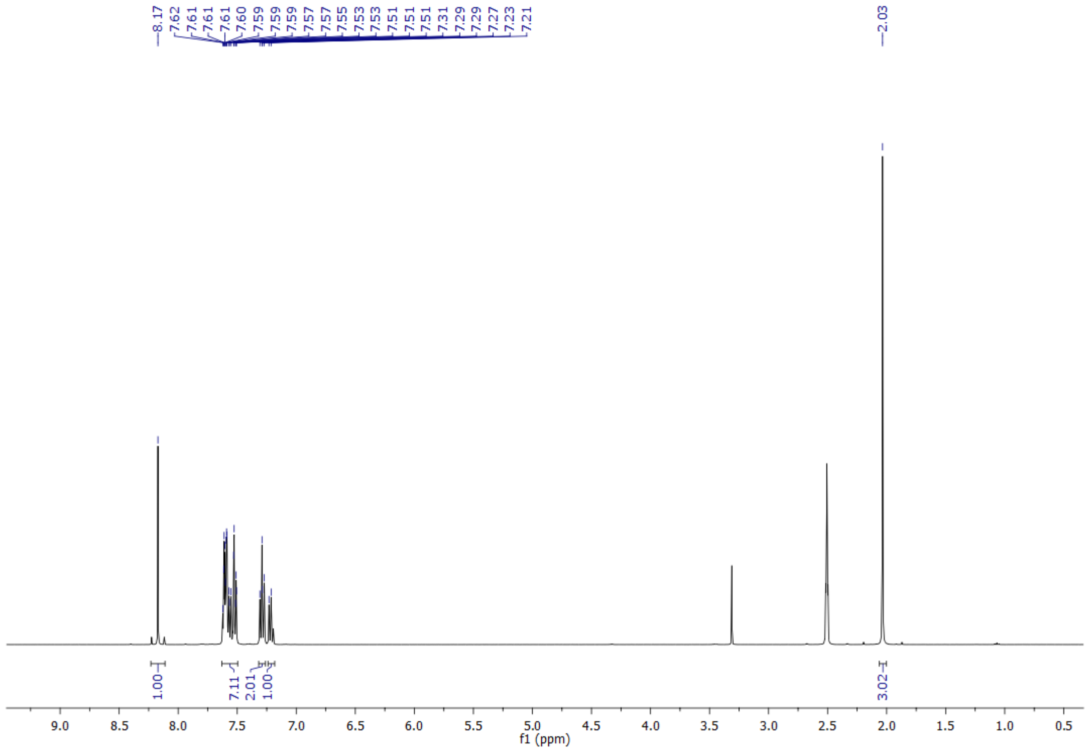

*Figure S 81: *

Figure S 81: ^{1}H-NMR (400 MHz, DMSO-d_6) of compound 27

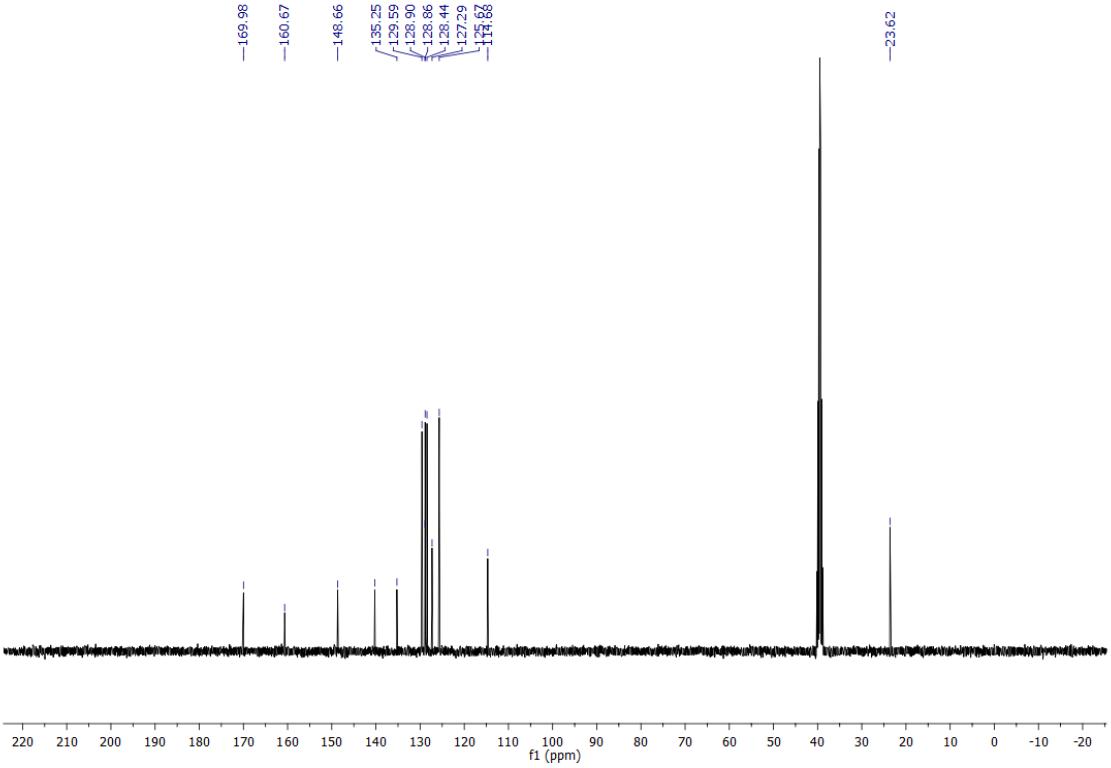

Figure S 82: ^{13}C-NMR (101 MHz, DMSO-d_6) of compound 27

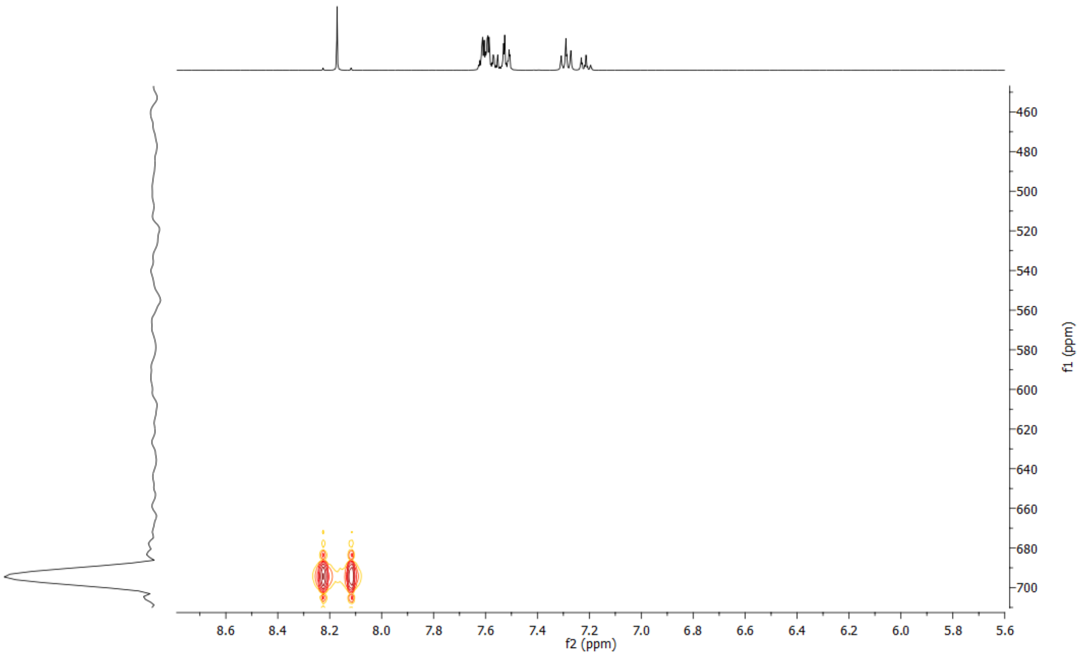

Figure S 83: ^{1}H-^{77}Se-HMBC (400 MHz, 76 MHz, DMSO-d_6) of compound 27

Compound 28

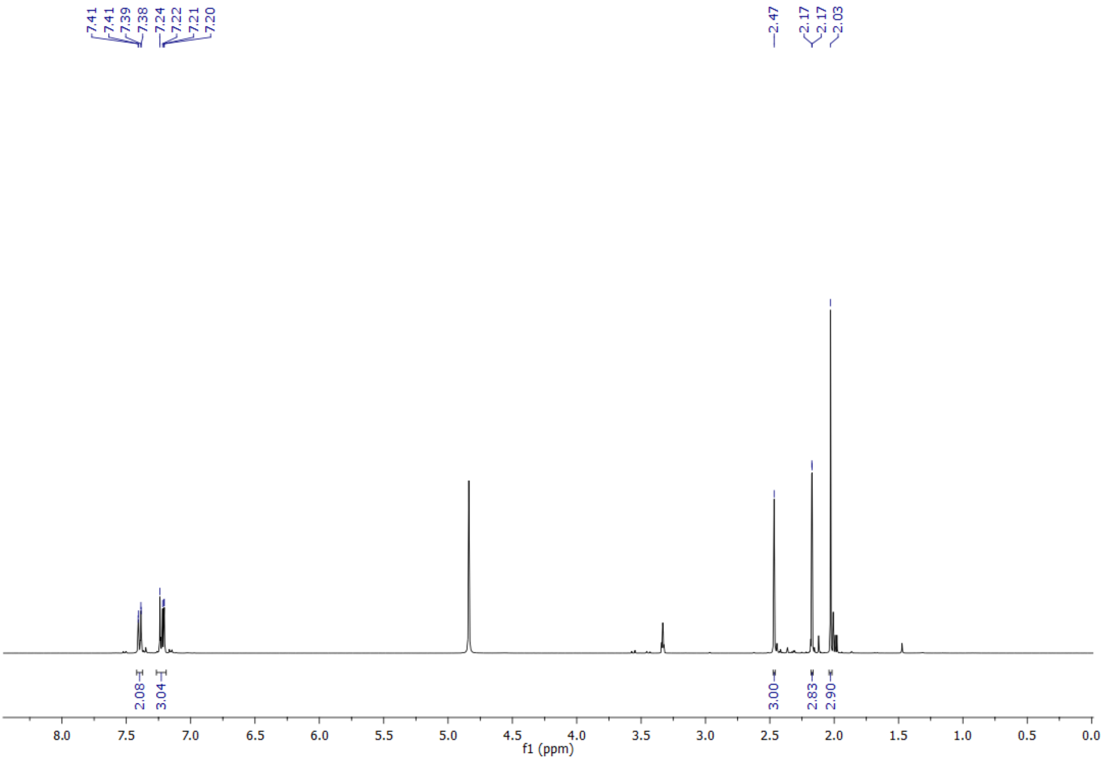

Figure S 84: ^{1}H-NMR (400 MHz, MeOD) of compound 28

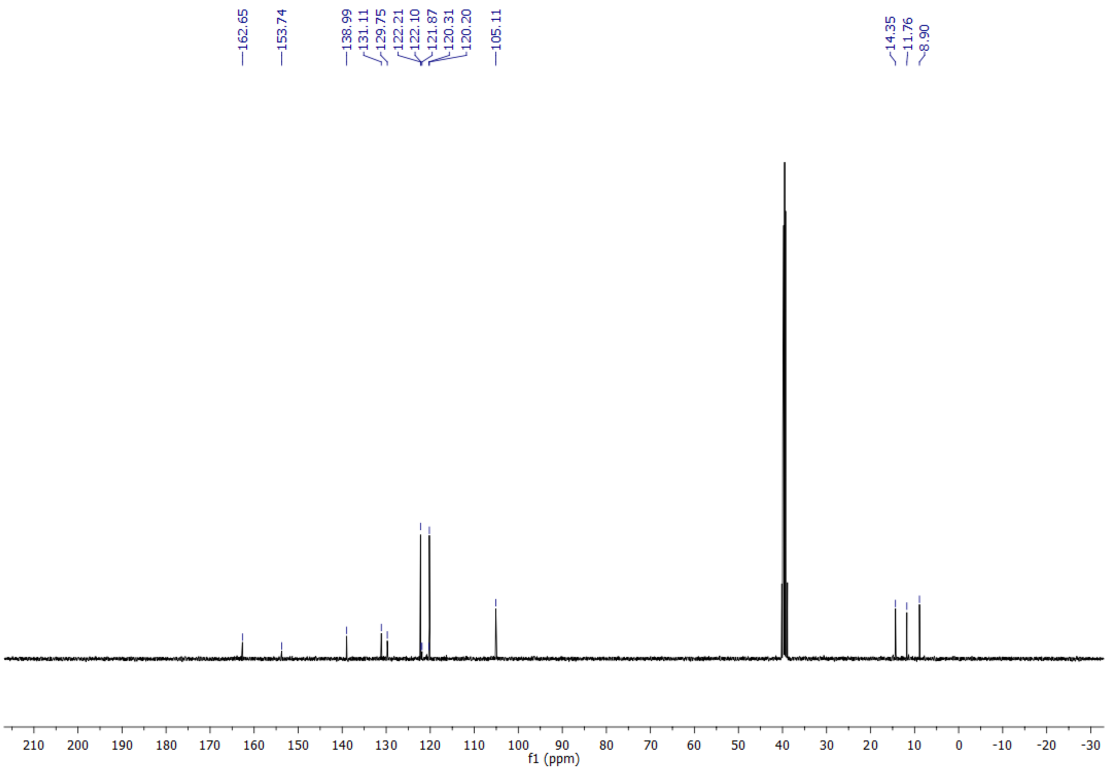

Figure S 85: ^{13}C-NMR (101 MHz, MeOD) of compound 28

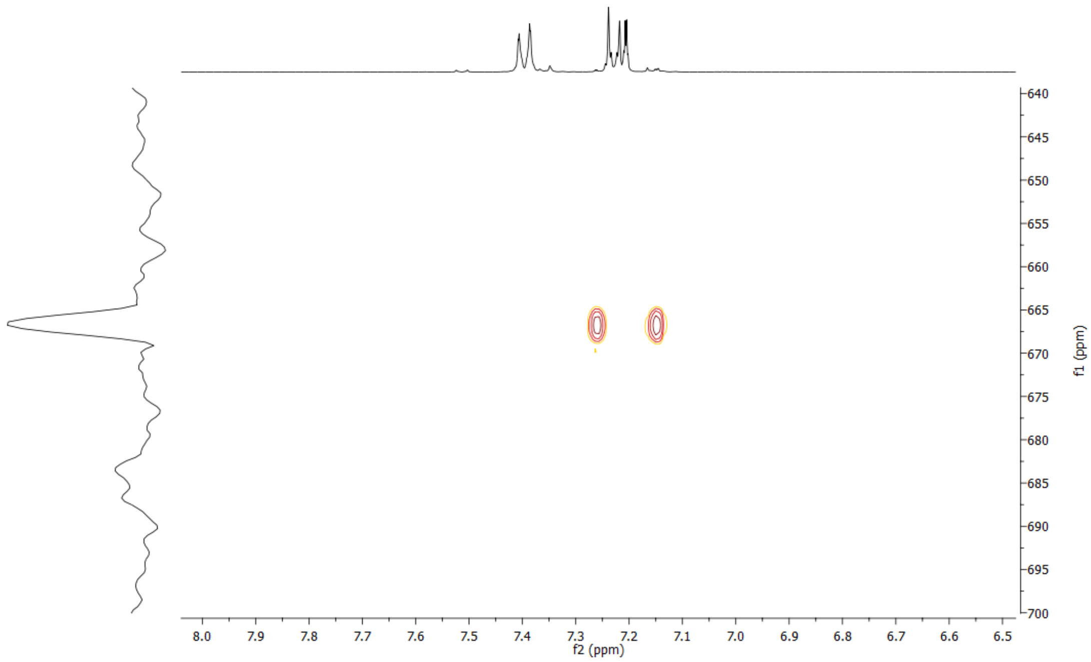

Figure S 86: ^{1}H-^{77}Se-HMBC (400 MHz, 76 MHz, MeOD) of compound 28

Compound 29

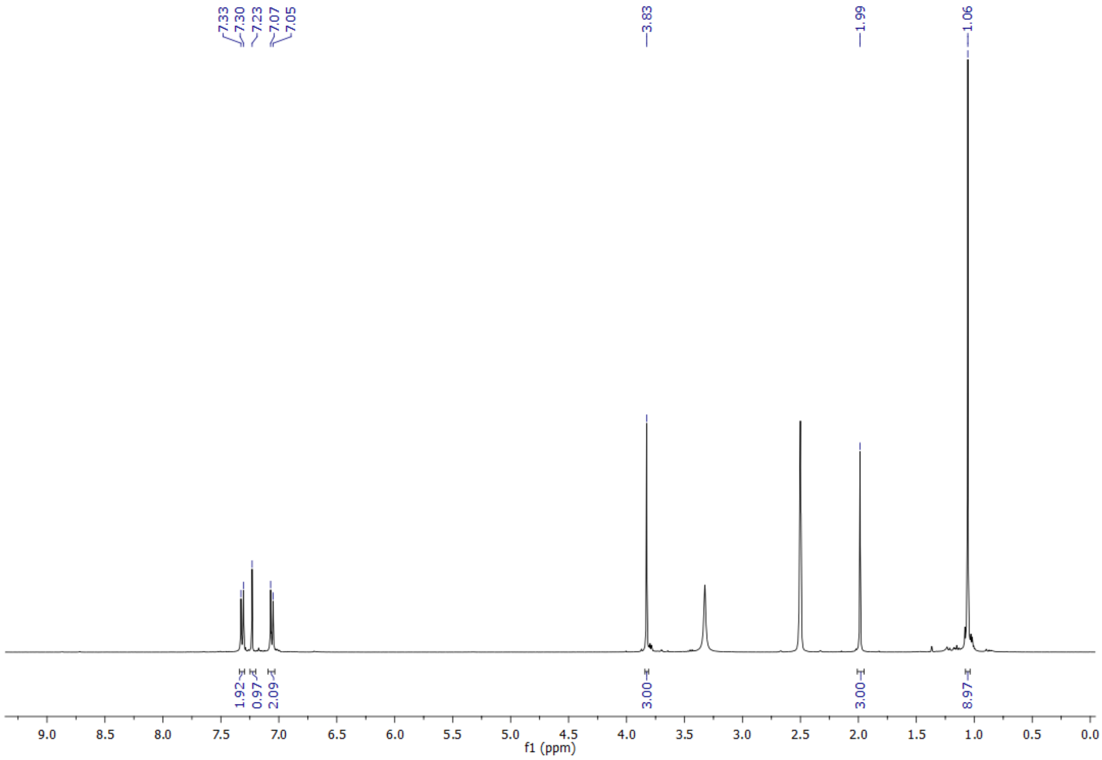

Figure S 87: ^{1}H-NMR (400 MHz, DMSO-d_6) of compound 29

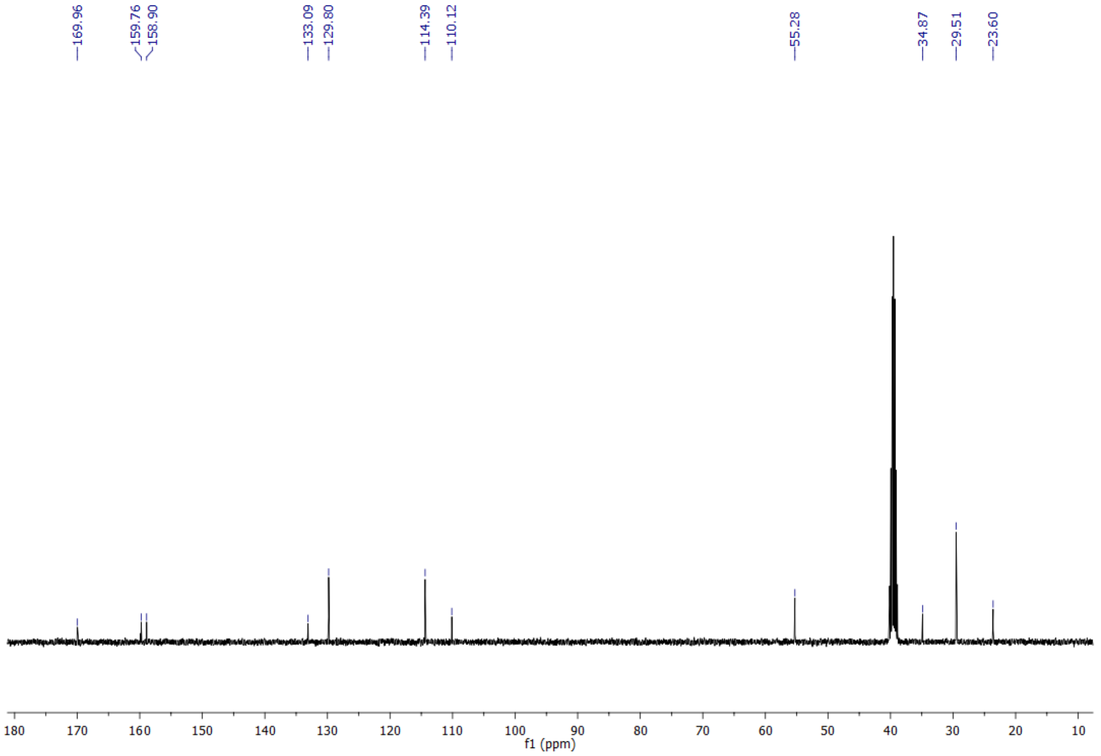

Figure S 88: ^{13}C-NMR (101 MHz, DMSO-d_6) of compound 29

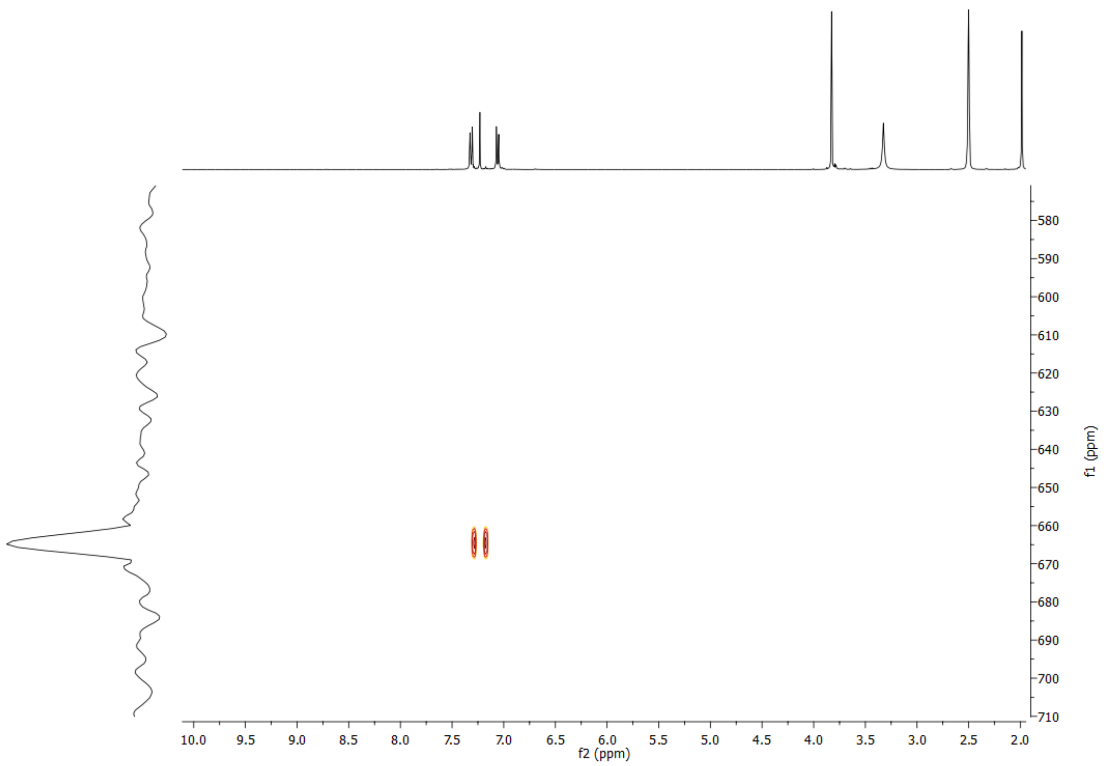

Figure S 89: ¹H-⁷⁷Se-HMBC (400 MHz, 76 MHz, DMSO-d₆) of compound 29

Compound 30

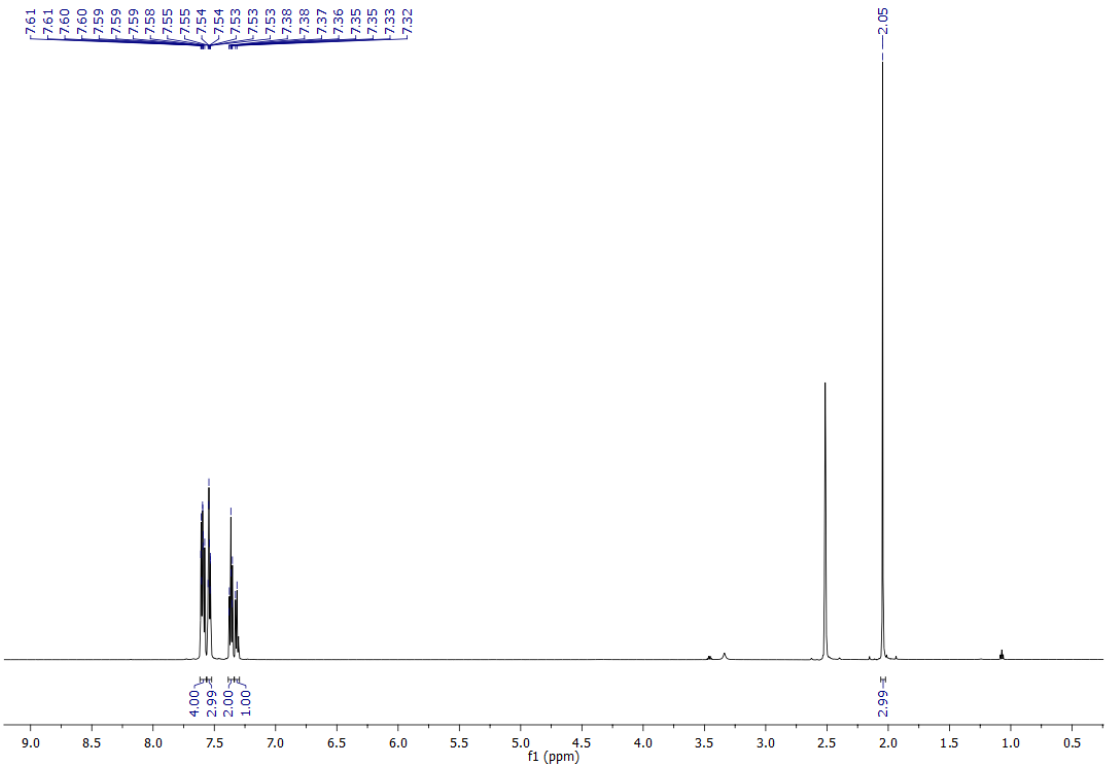

Figure S 90: ¹H-NMR (600 MHz, DMSO-d₆) of compound 30

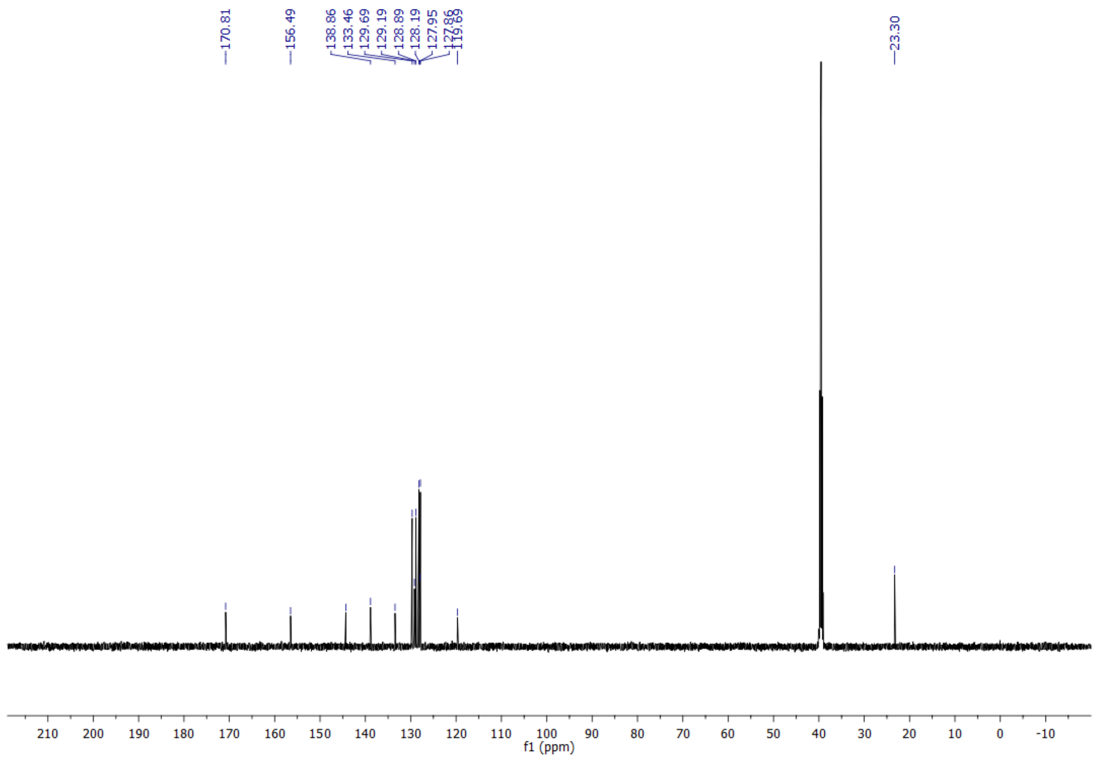

Figure S 91: ^{13}C-NMR (151 MHz, DMSO-d_6) of compound 30

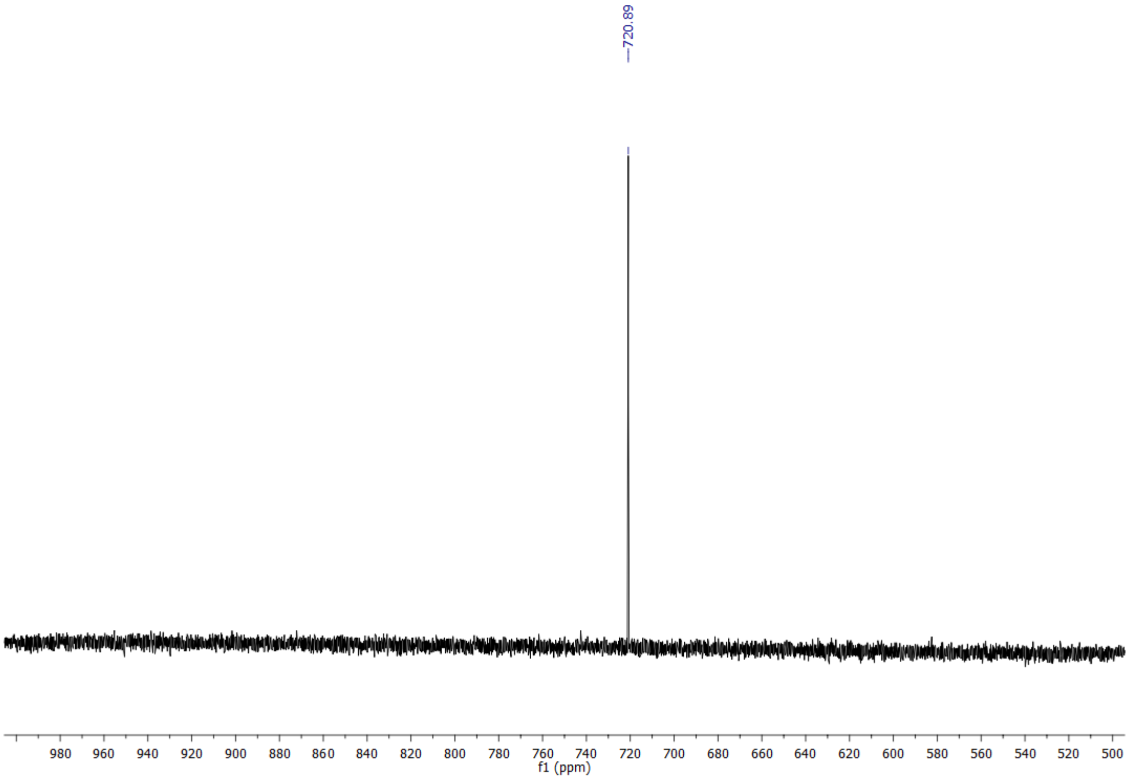

Figure S 92: ^{77}Se-NMR (114 MHz, DMSO-d_6) of compound 30

Compound 31

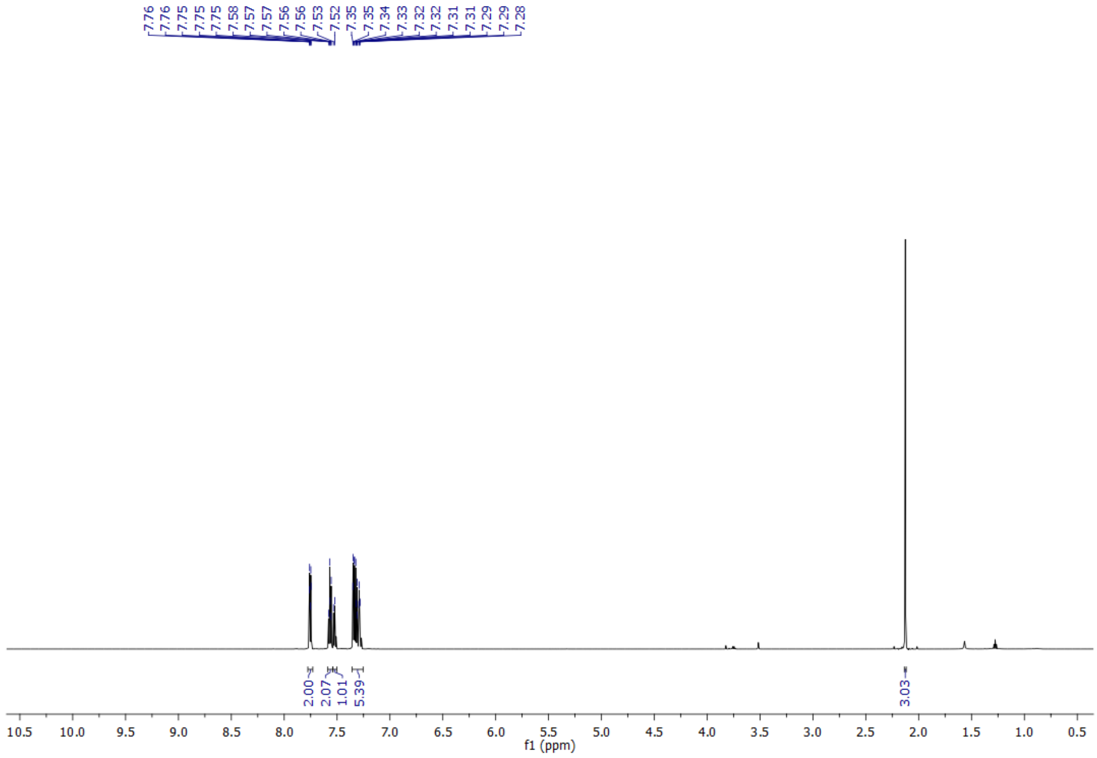

Figure S 93: ^{1}H-NMR (600 MHz, DMSO-d_6) of compound 31

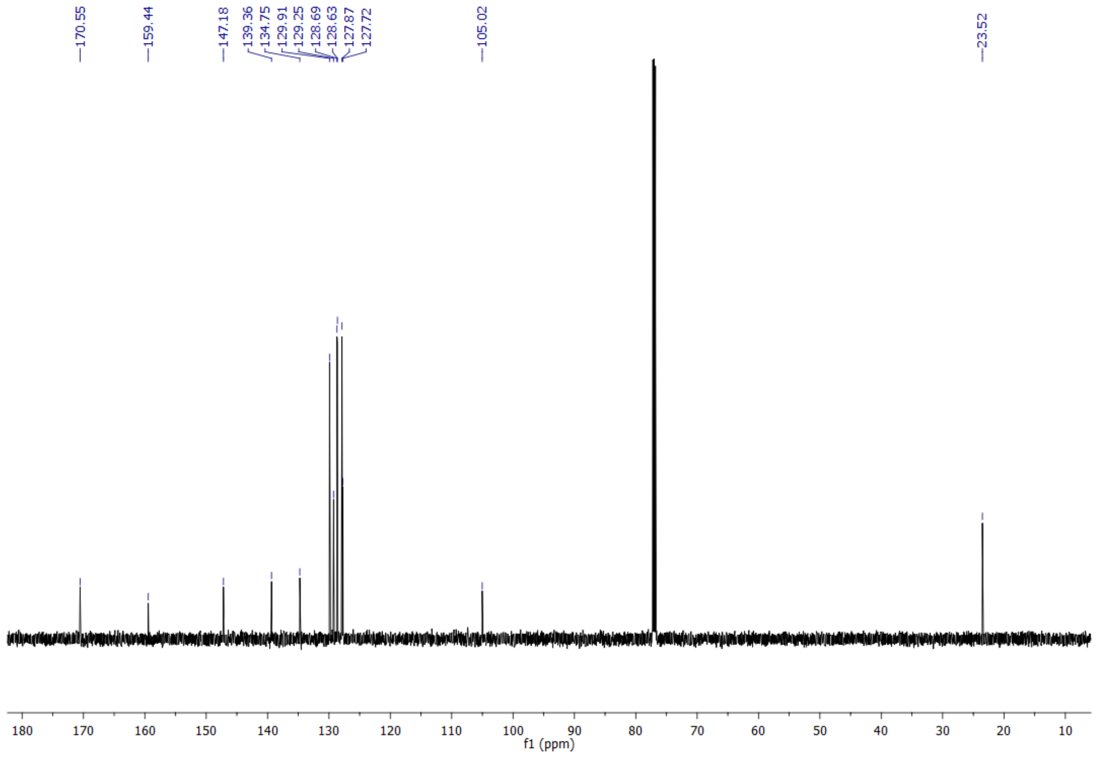

Figure S 94: ^{13}C-NMR (151 MHz, DMSO-d_6) of compound 31

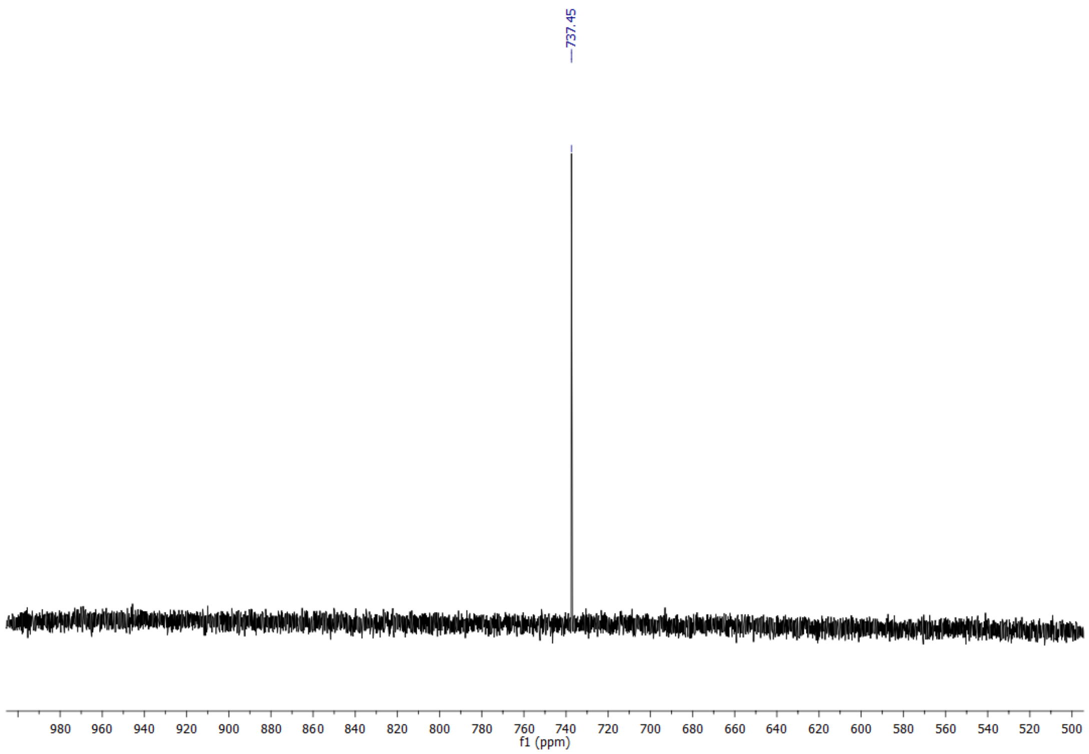

Figure S 95: ^{77}Se-NMR (114 MHz, DMSO-d_6) of compound 31

Compound 32

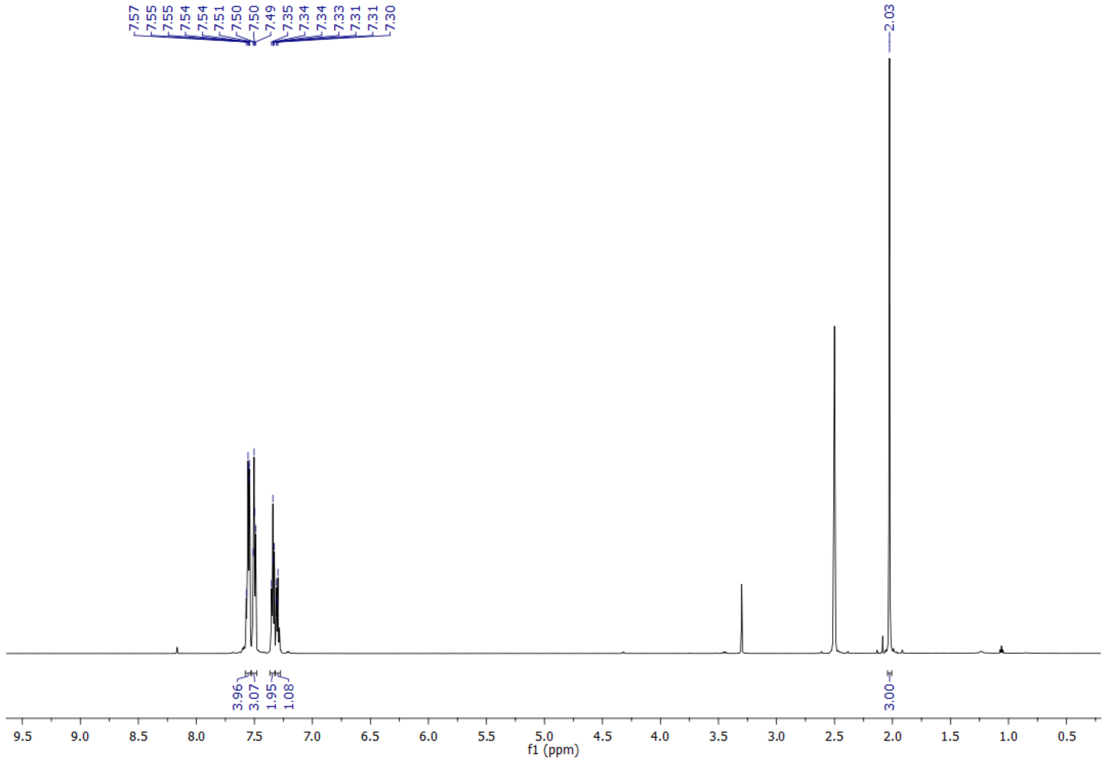

Figure S 96: ^{1}H-NMR (600 MHz, DMSO-d_6) of compound 32

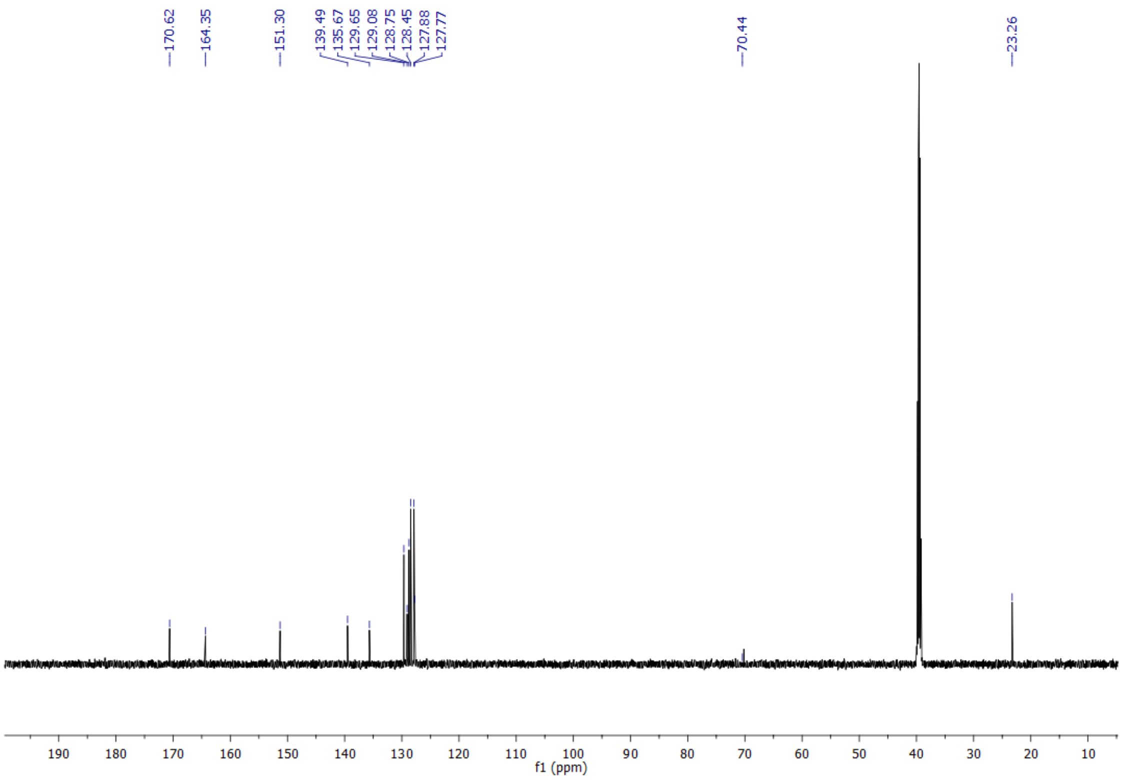

Figure S 97: ^{13}C-NMR (151 MHz, DMSO-d_6) of compound 32

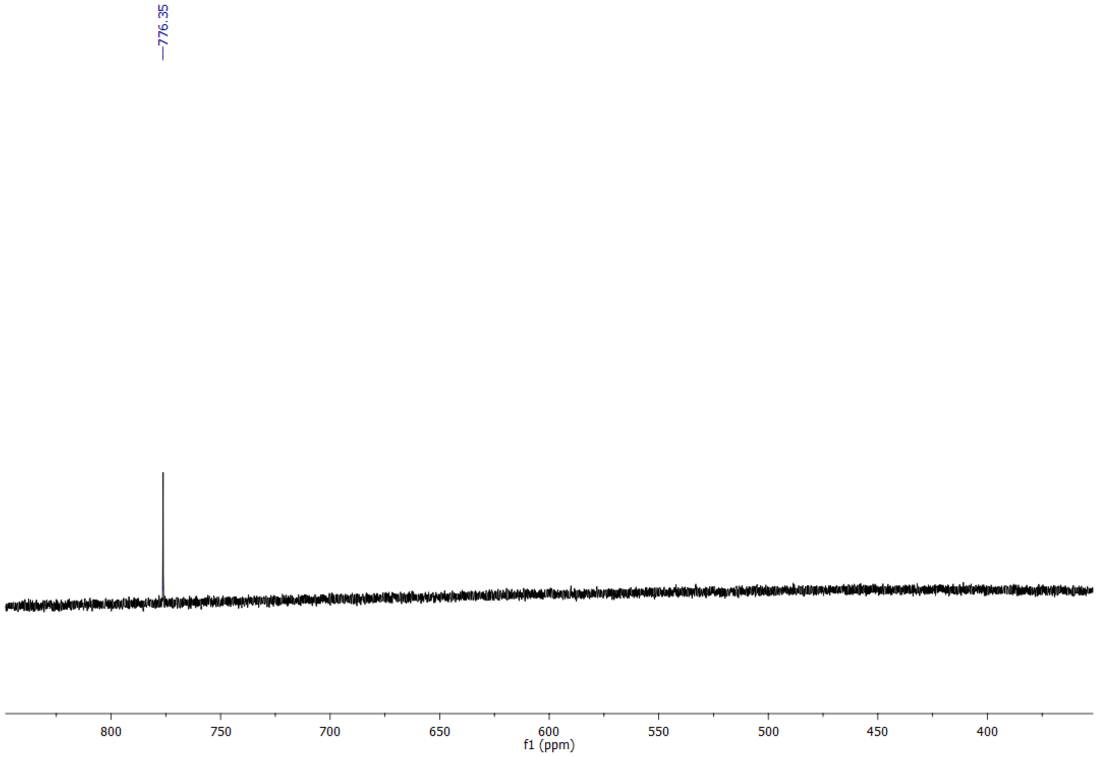

Figure S 98: ^{77}Se-NMR (114 MHz, DMSO-d_6) of compound 32

Compound 33

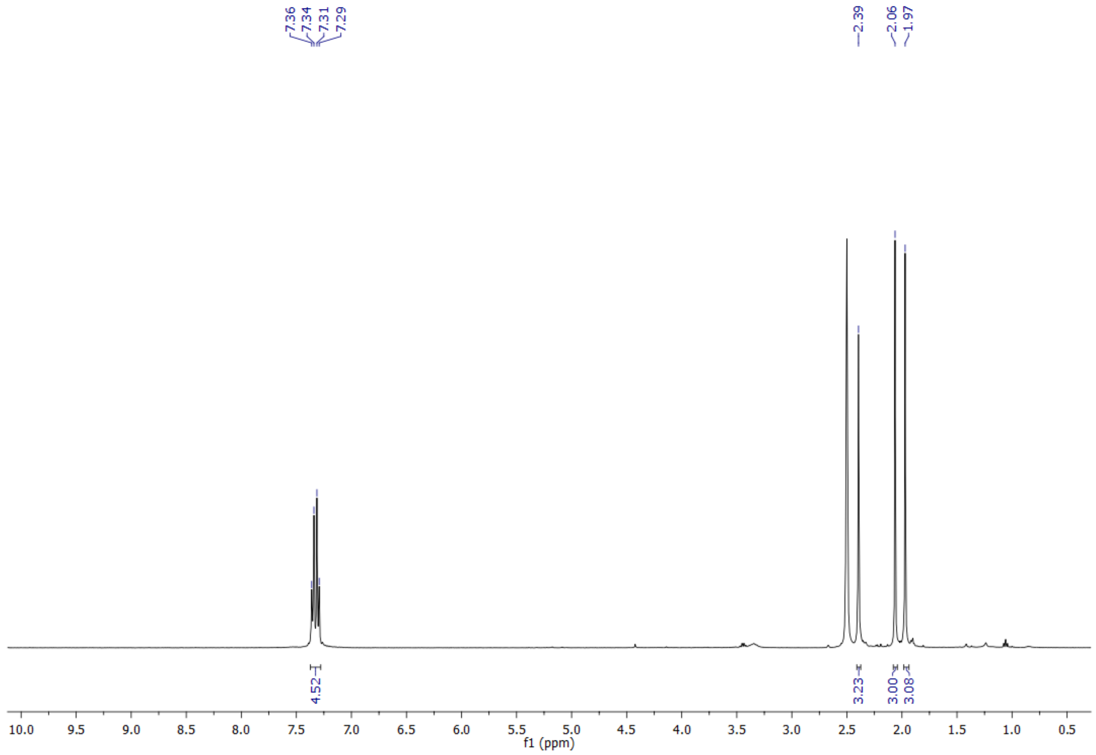

Figure S 99: ^{1}H-NMR (400 MHz, DMSO-d_6) of compound 33

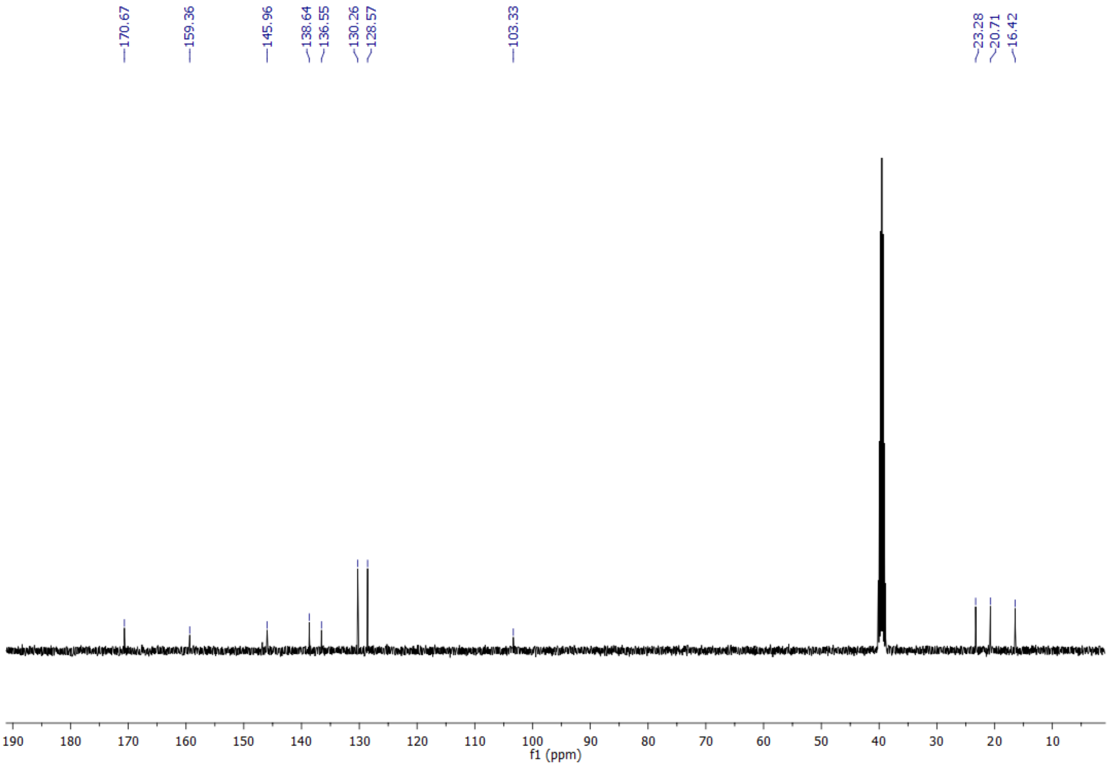

Figure S 100: ^{13}C-NMR (101 MHz, DMSO-d_6) of compound 33

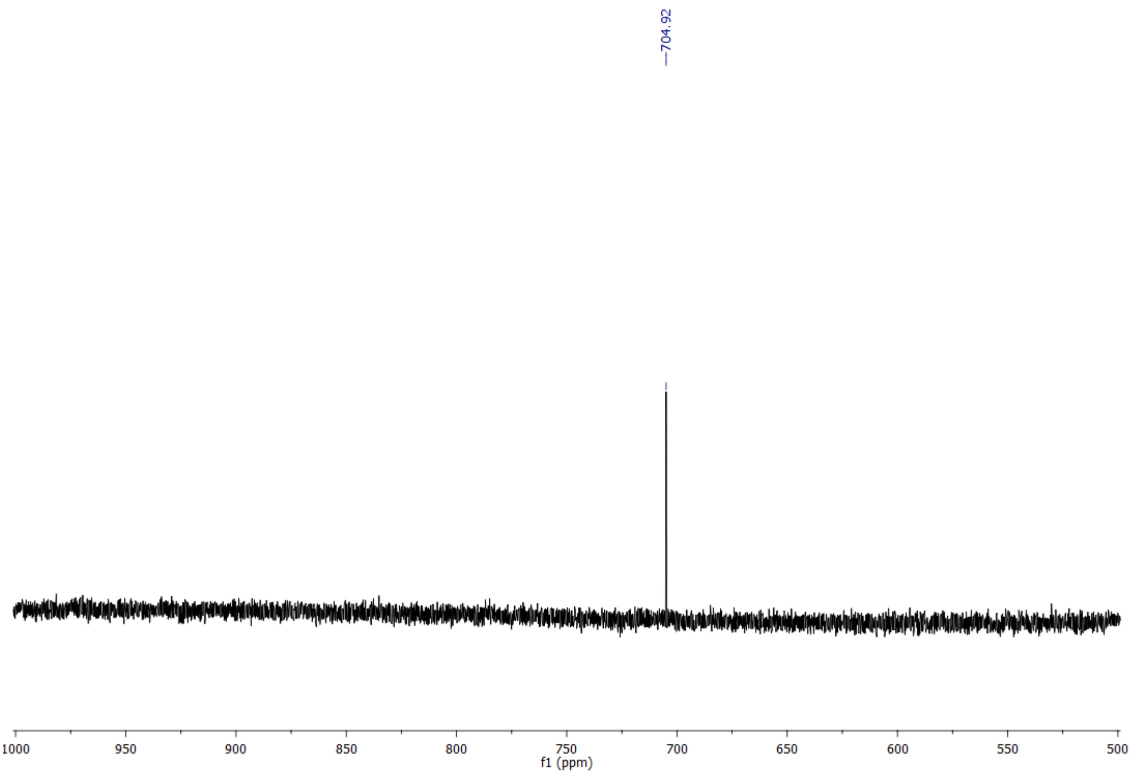

Figure S 101: ^{77}Se-NMR (76 MHz, DMSO-d_6) of compound 33

Compound 34

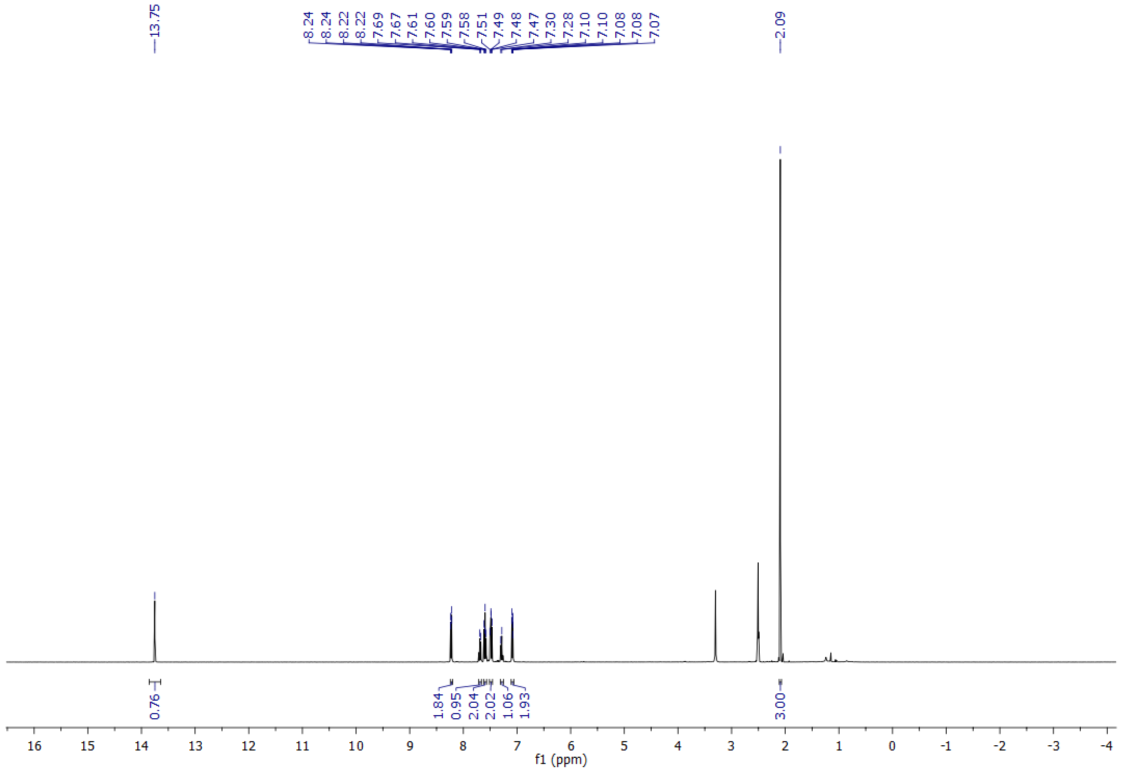

Figure S 102: ^{1}H-NMR (400 MHz, DMSO-d_6) of compound 34

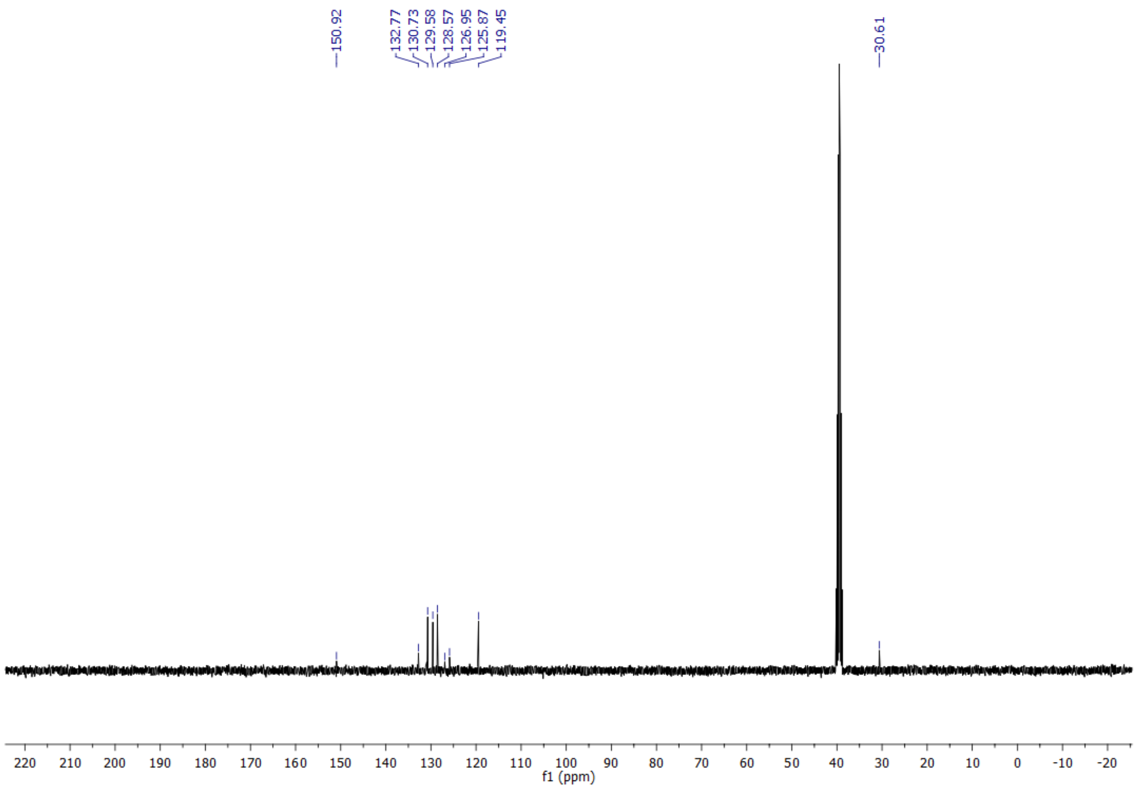

Figure S 103: ^{13}C-NMR (101 MHz, DMSO-d_6) of compound 34

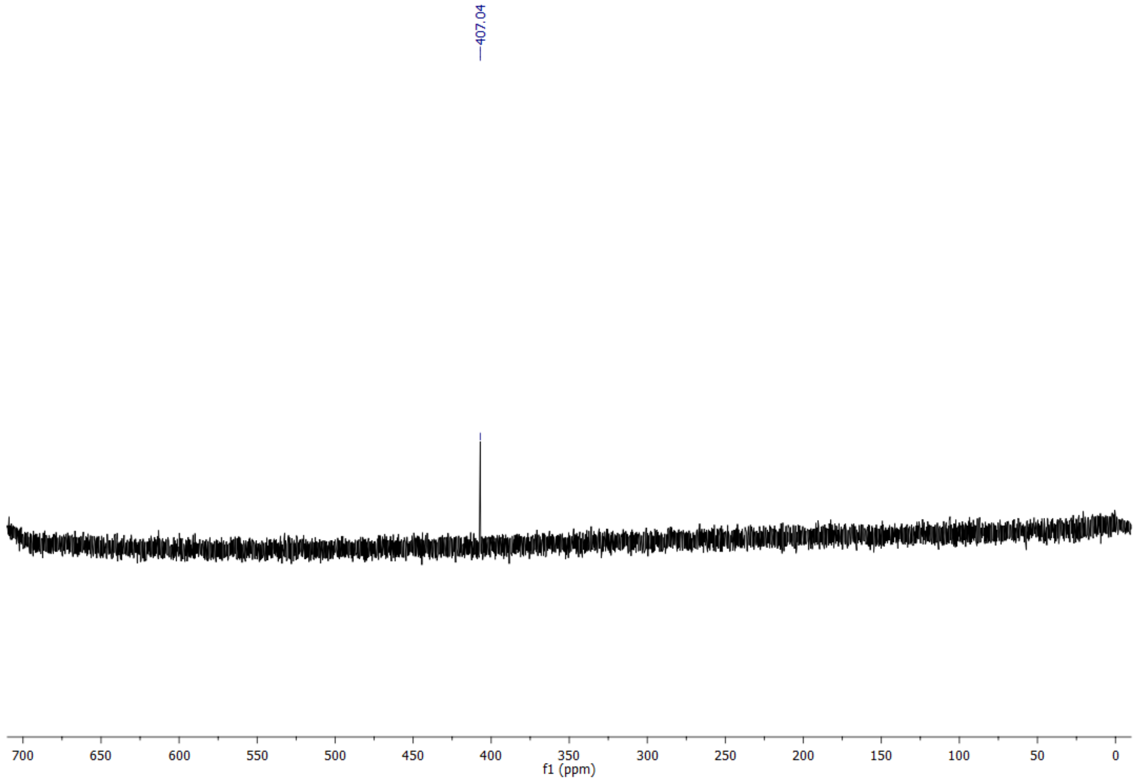

Figure S 104: ^{77}Se-NMR (76 MHz, DMSO-d_6) of compound 34

Compound 35

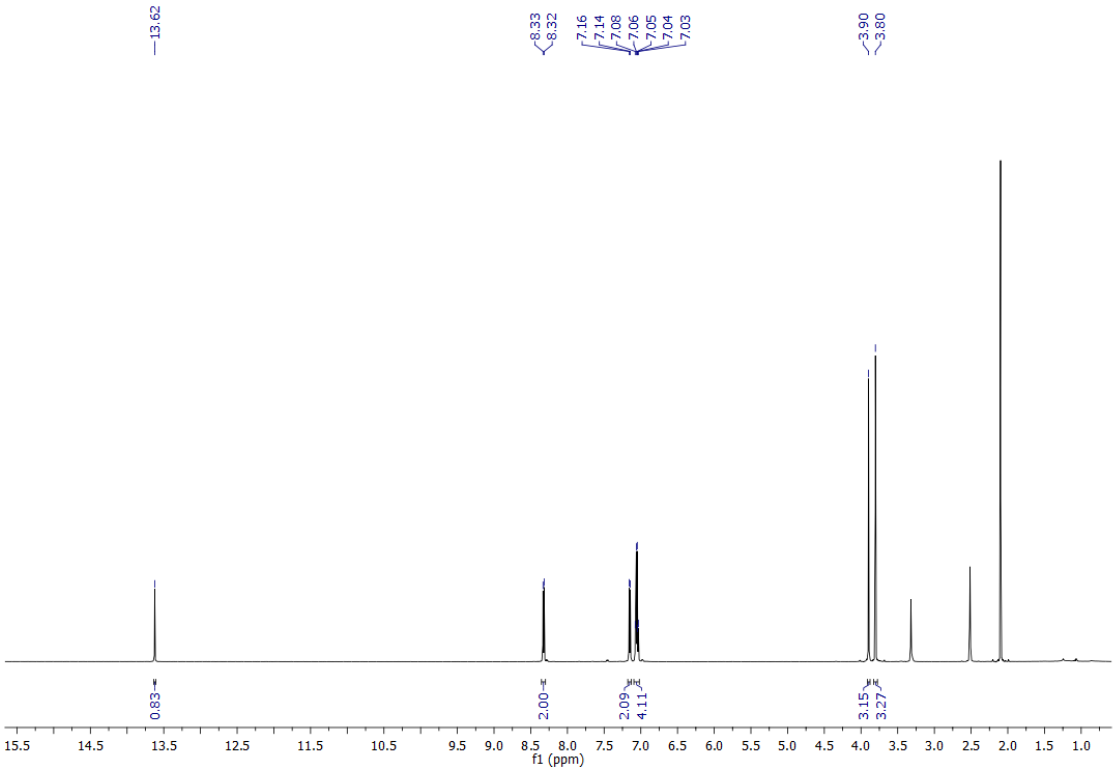

Figure S 105: ^{1}H-NMR (600 MHz, DMSO-d_6) of compound 35

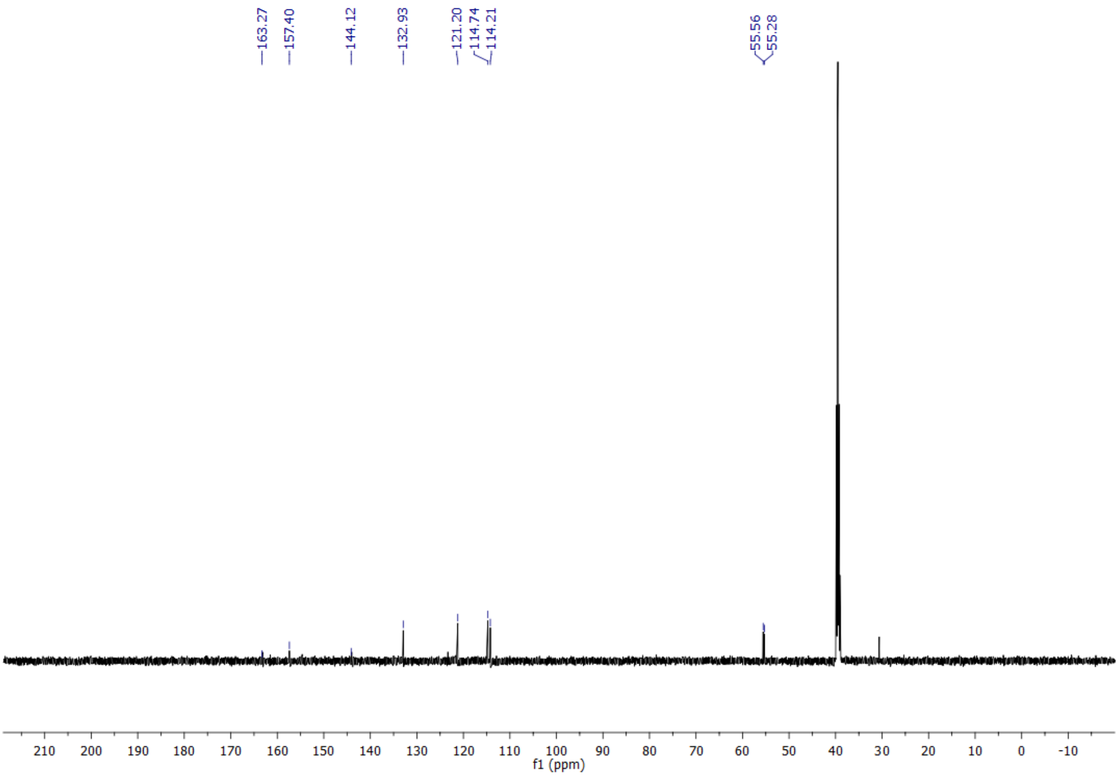

Figure S 106: ^{13}C-NMR (151 MHz, DMSO-d_6) of compound 35

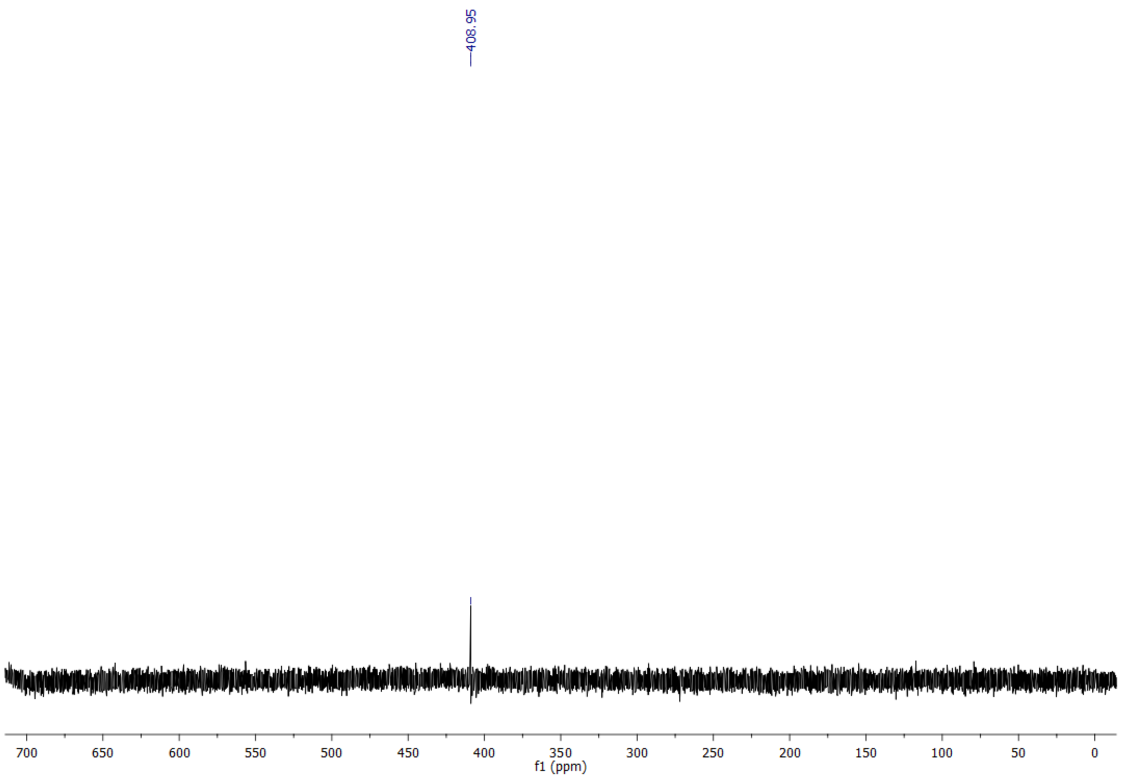

Figure S 107: ^{77}Se-NMR (114 MHz, DMSO-d_6) of compound 35

Compound 36

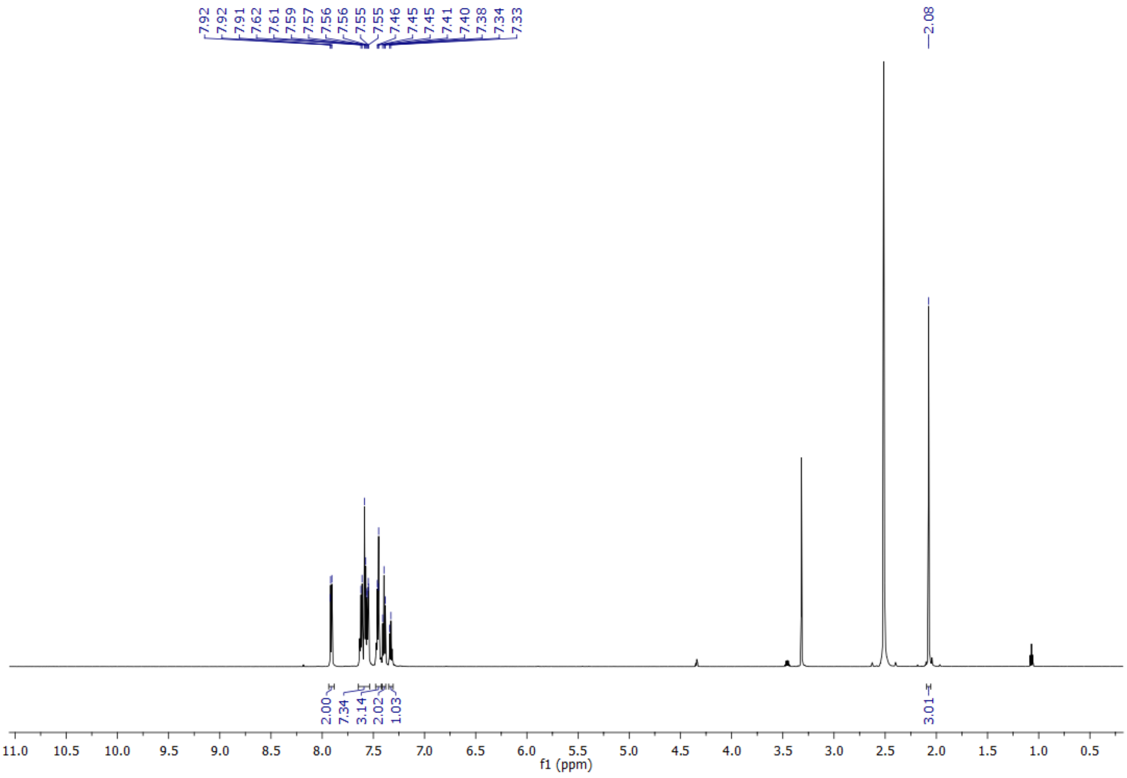

Figure S 108: ^{1}H-NMR (600 MHz, DMSO-d_6) of compound 36

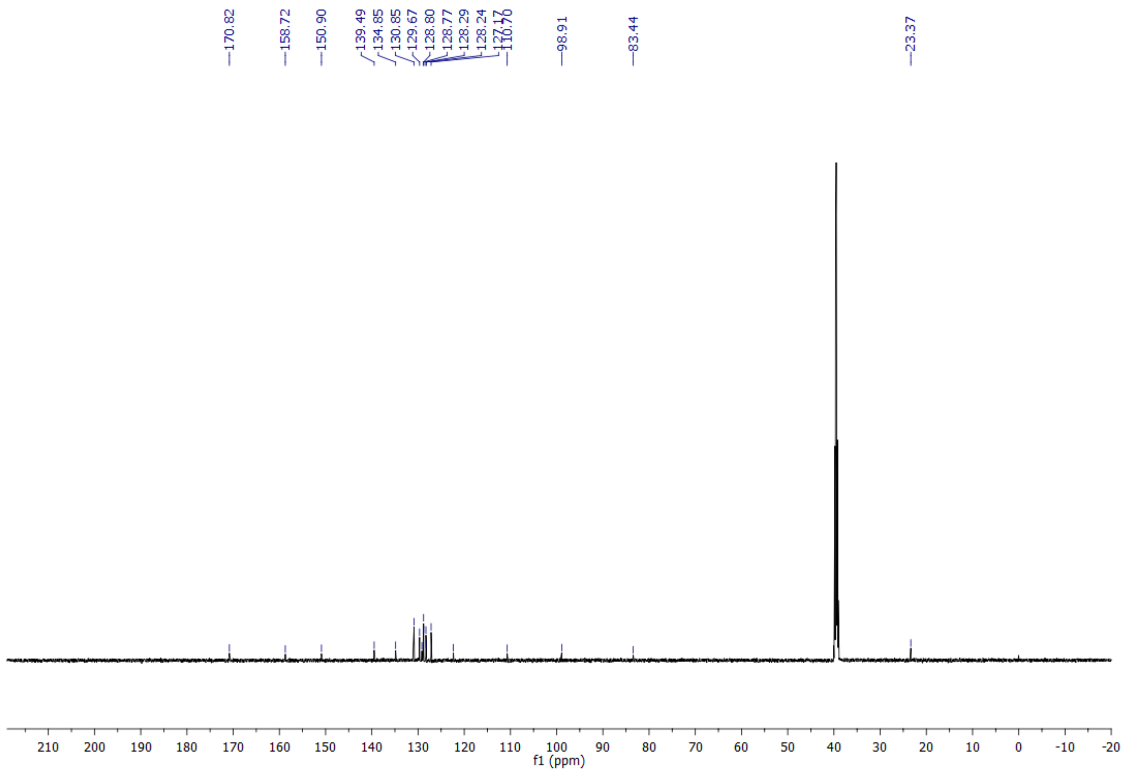

Figure S 109: ^{13}C-NMR (151 MHz, DMSO-d_6) of compound 36

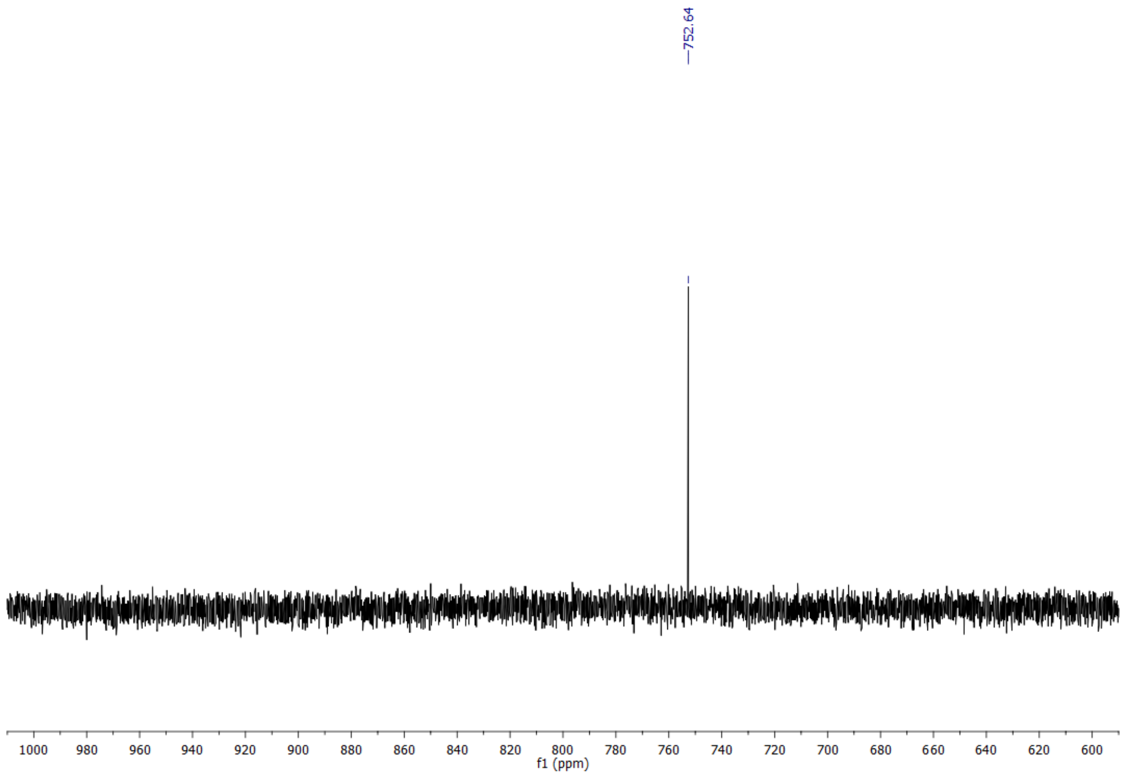

Figure S 110: ^{77}Se-NMR (76 MHz, DMSO-d_6) of compound 36

Compound 37

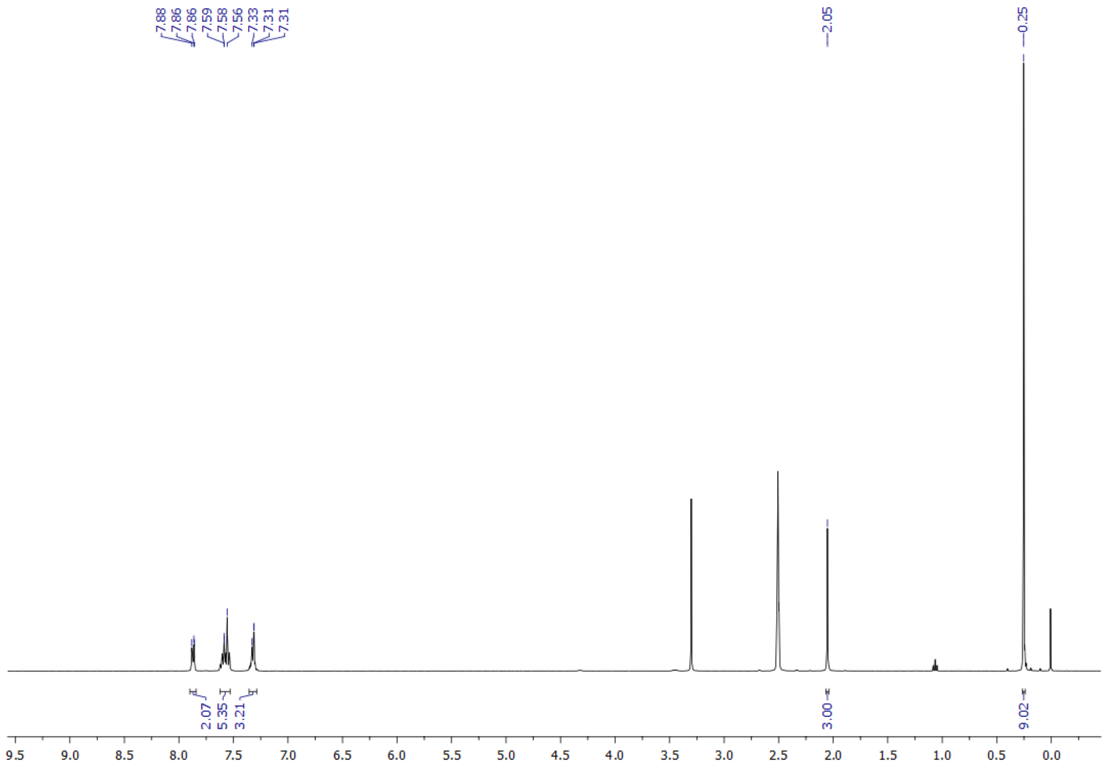

Figure S 111: ^{1}H-NMR (400 MHz, DMSO-d_6) of compound 37

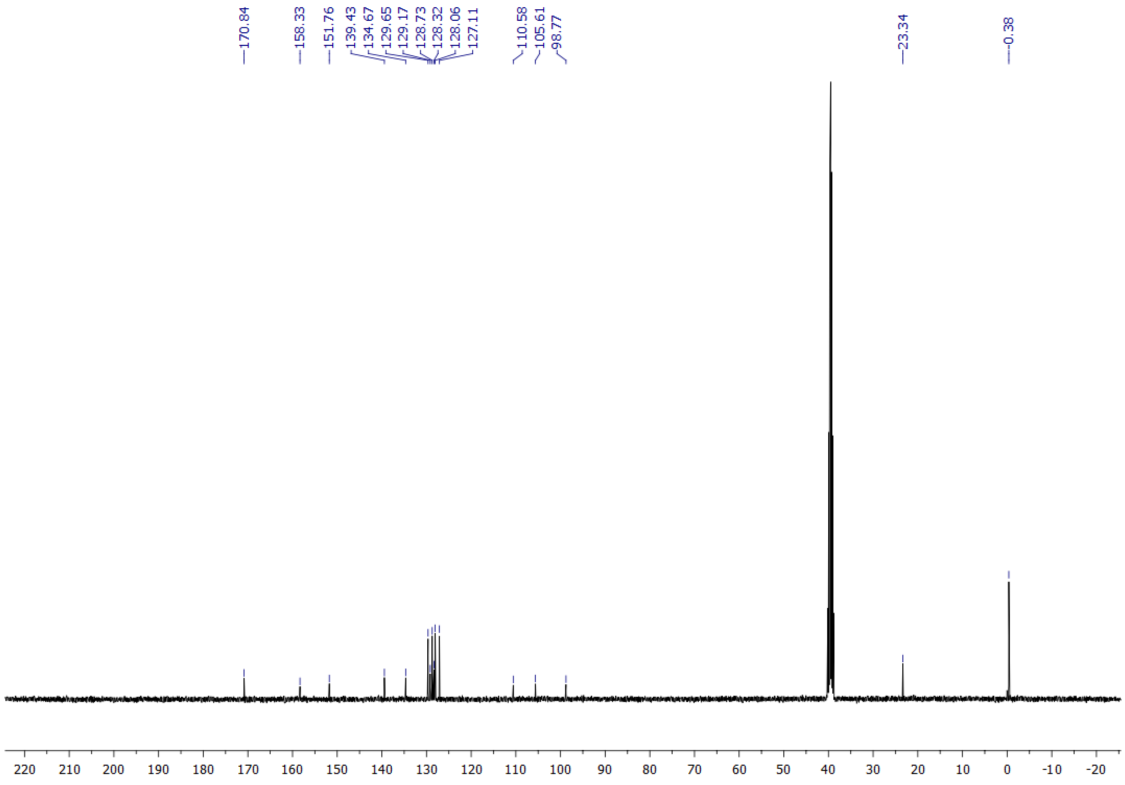

Figure S 112: ^{13}C-NMR (101 MHz, DMSO-d_6) of compound 37

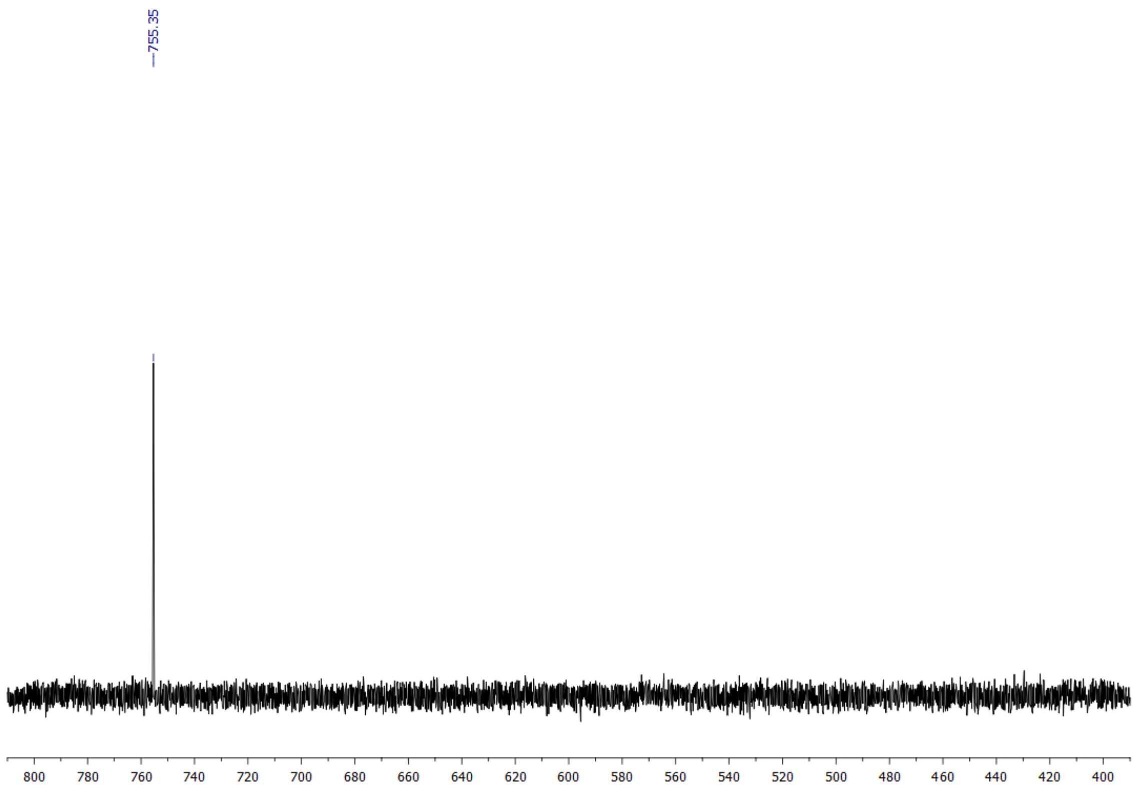

Figure S 113: ^{77}Se-NMR (76 MHz, DMSO) of compound 37

Compound 38

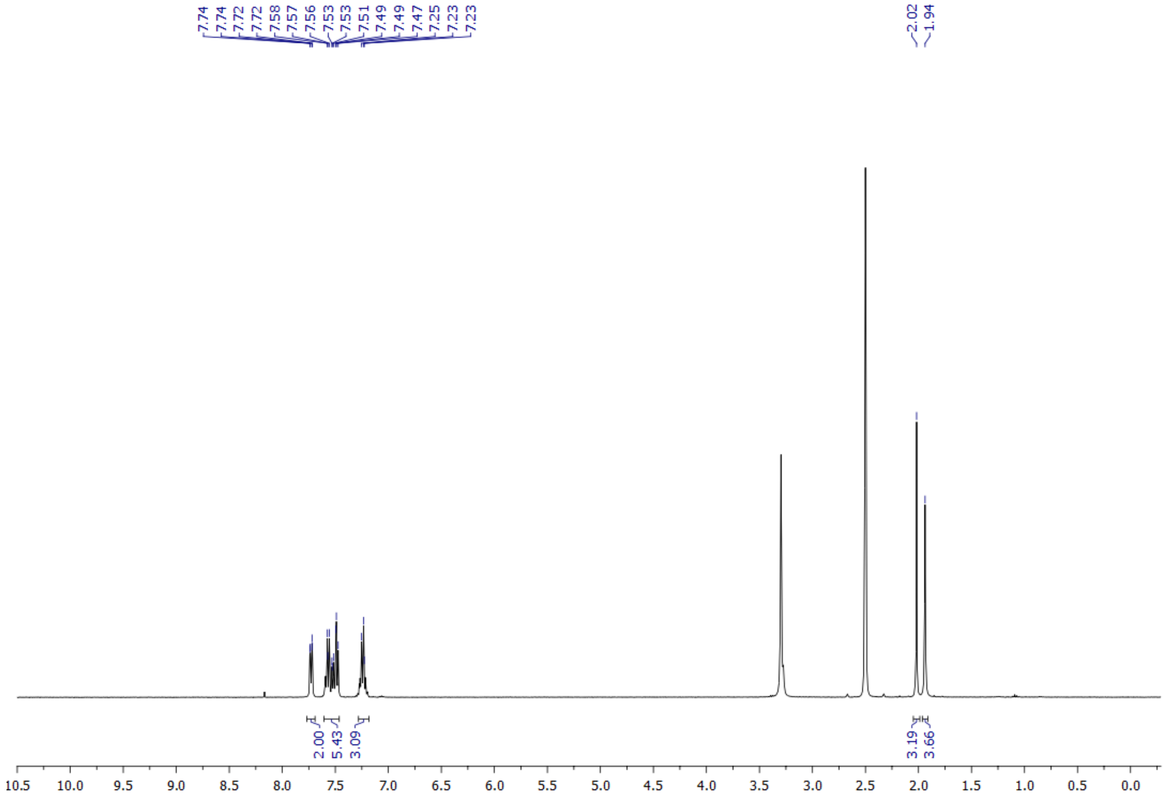

Figure S 114: ^{1}H-NMR (400 MHz, DMSO-d_6) of compound 38

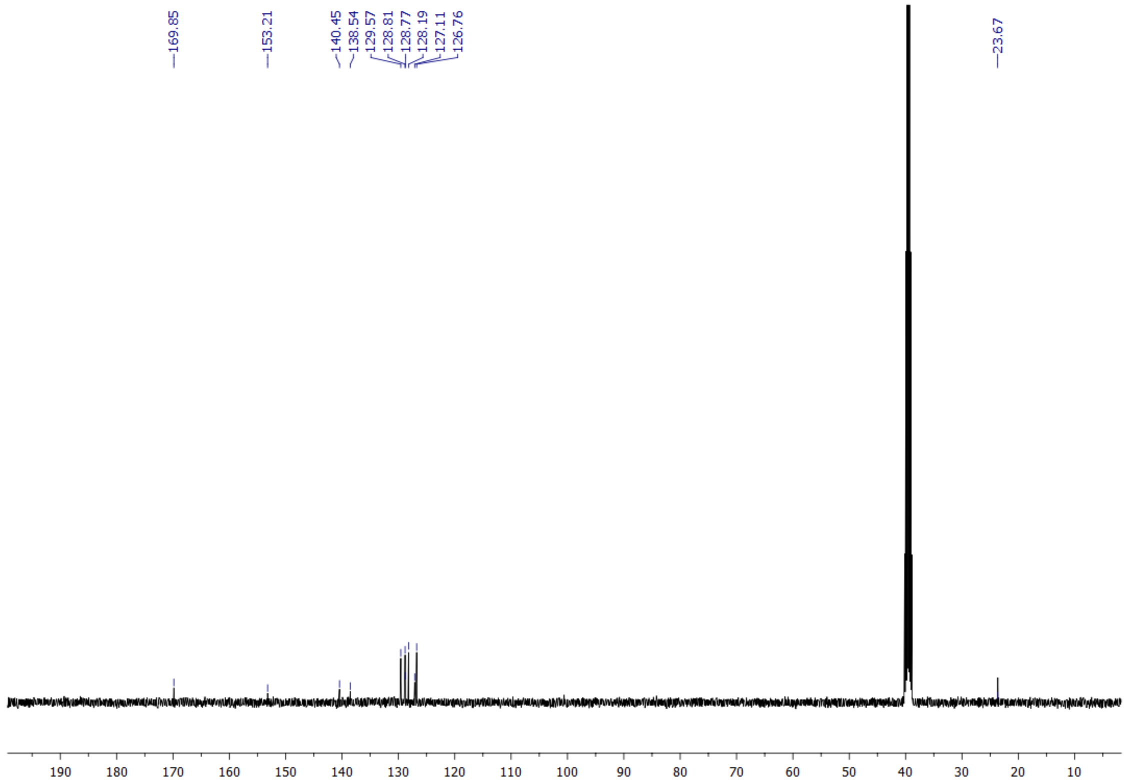

Figure S 115: ^{13}C-NMR (101 MHz, DMSO) of compound 38

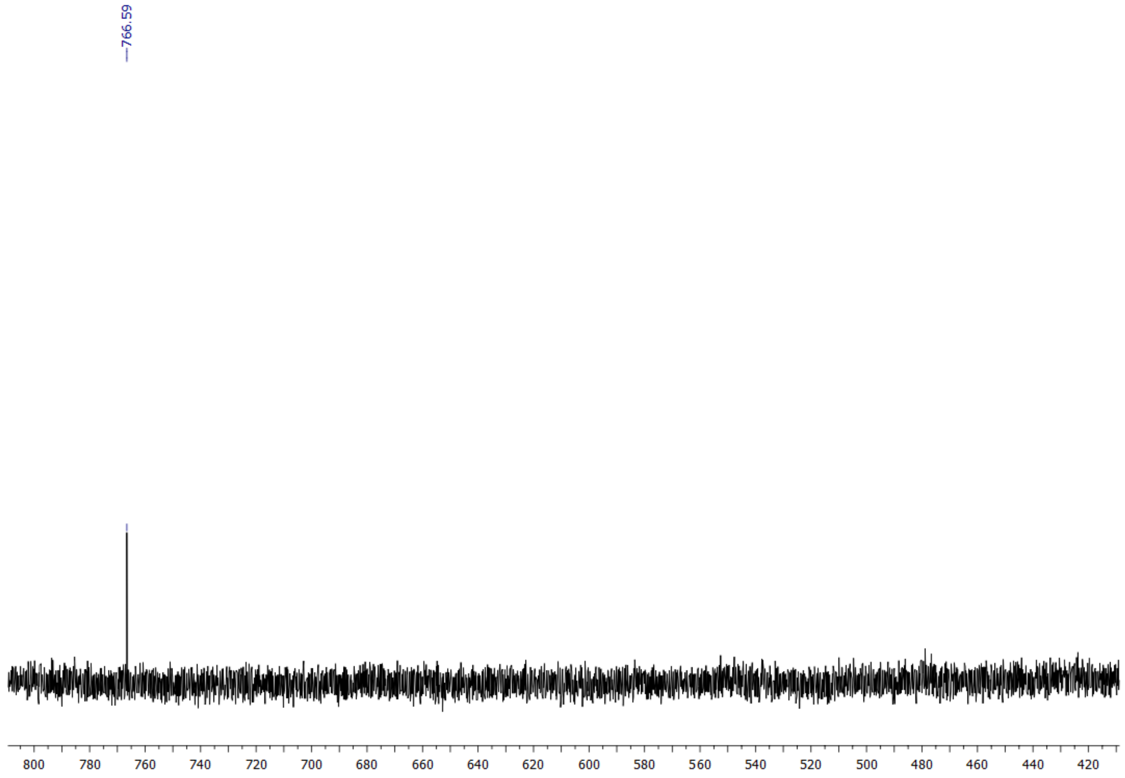

Figure S 116: ^{77}Se-NMR (76 MHz, DMSO) of compound 38

Compound 38S

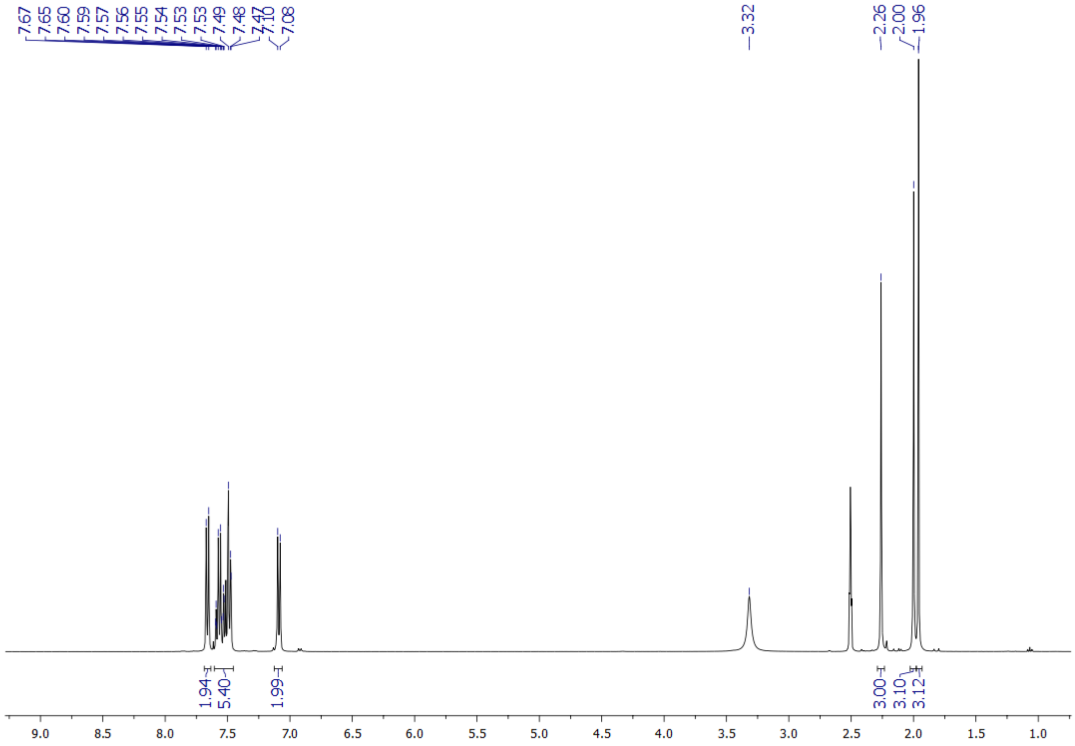

Figure S 117: ^{1}H-NMR (400 MHz, DMSO-d$_6$) of compound 38S

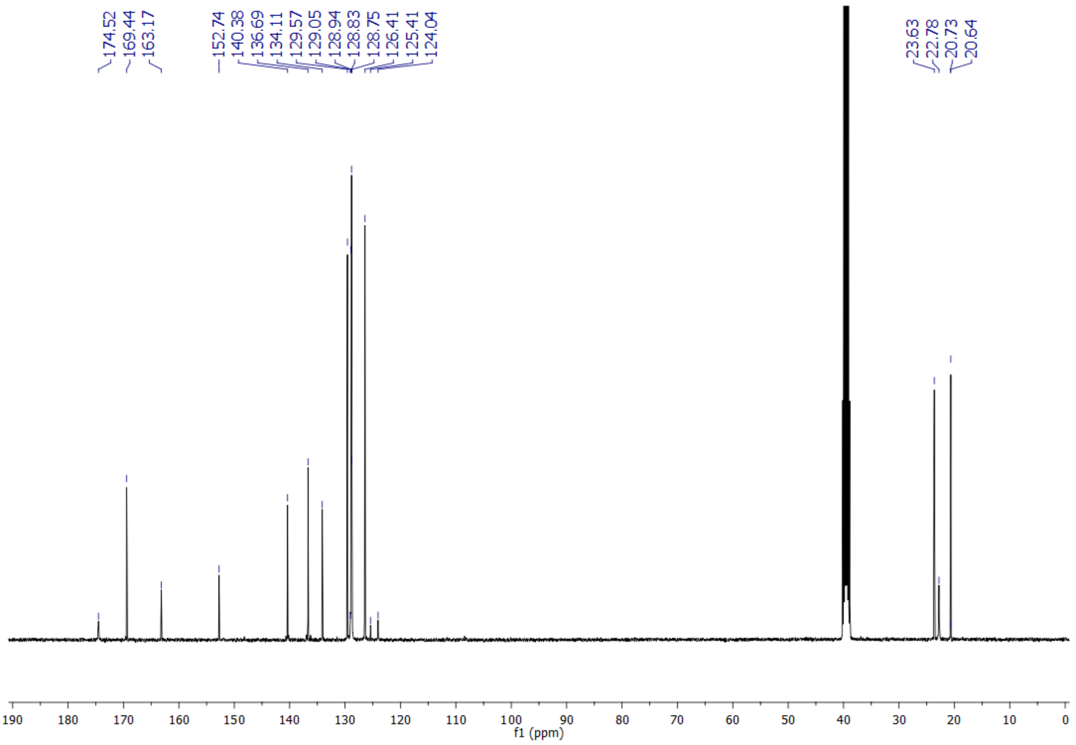

Figure S 118^{13}C-NMR (101 MHz, DMSO) of compound 38S

Compound 39

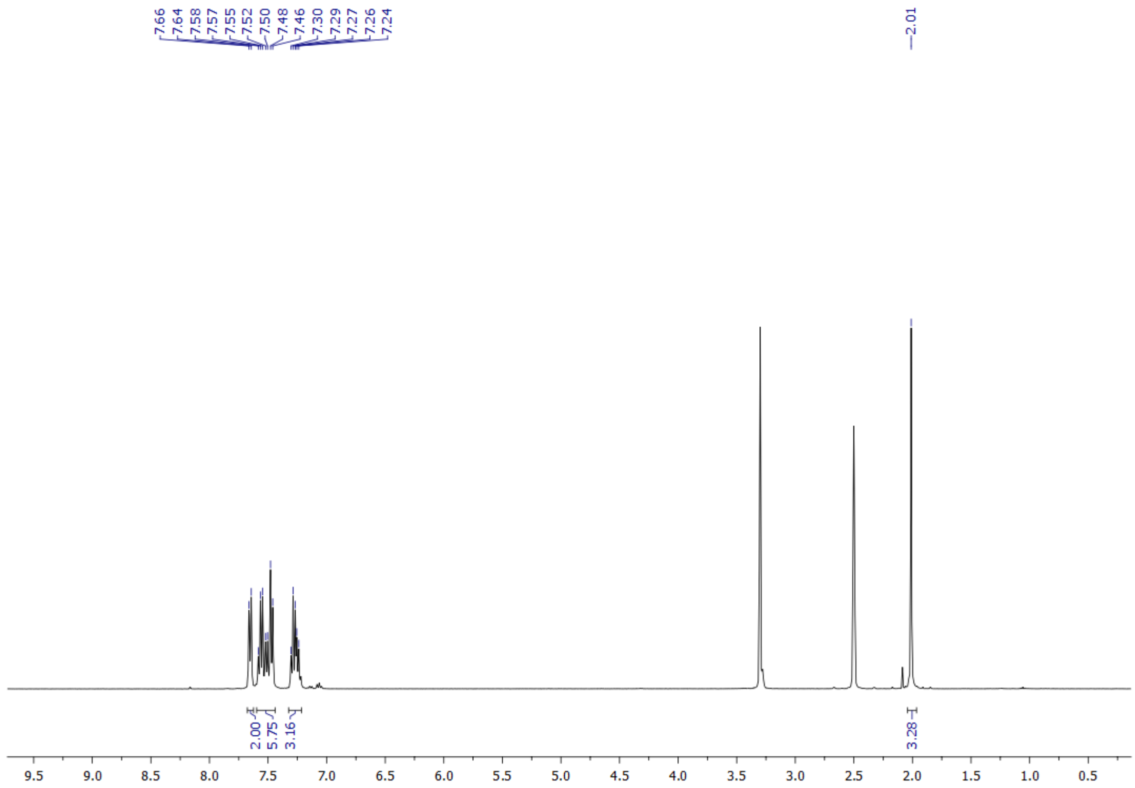

Figure S 119: ^{1}H-NMR (400 MHz, DMSO-d_6) of compound 39

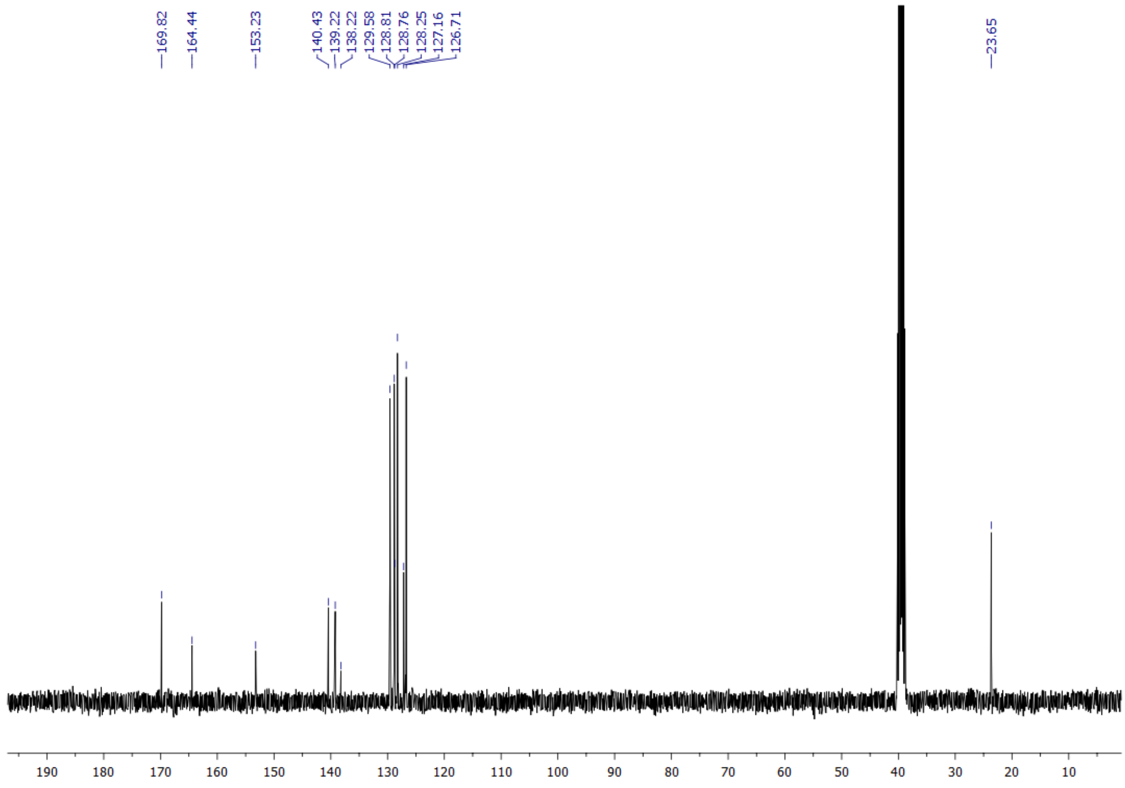

Figure S 120: ^{13}C-NMR (101 MHz, DMSO) of compound 39

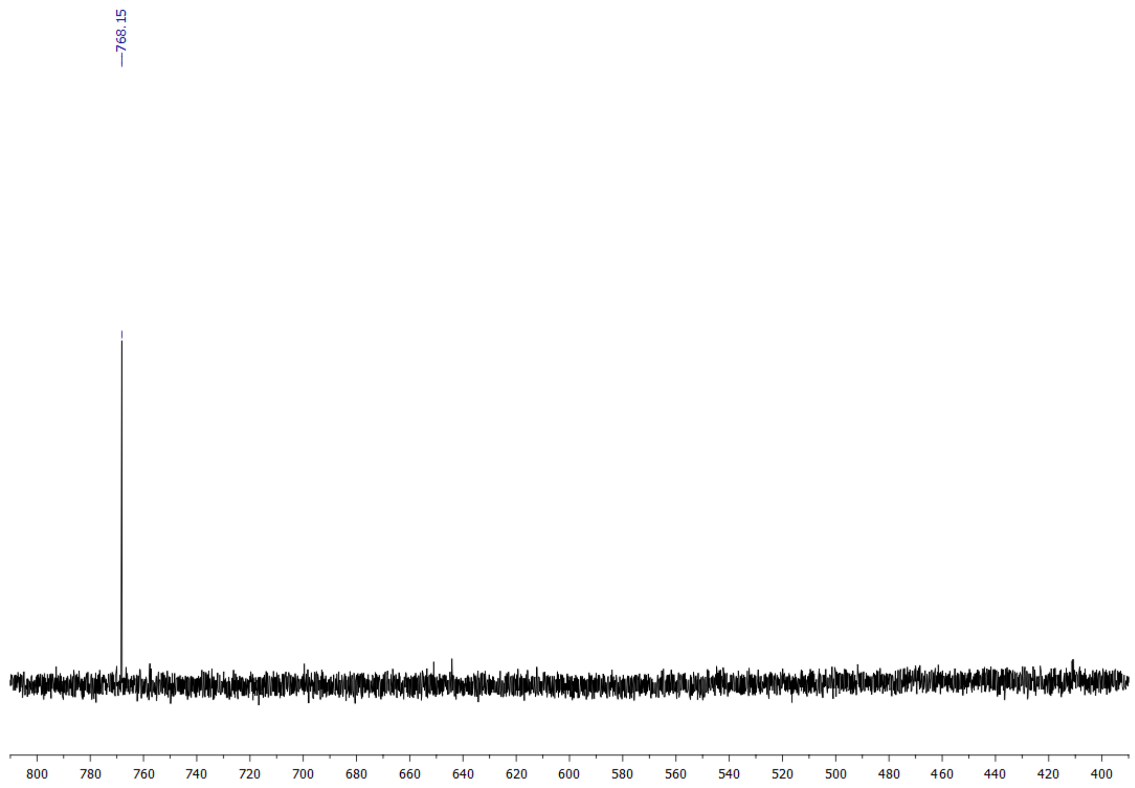

Figure S 121: ^{77}Se-NMR (76 MHz, DMSO) of compound 39

Compound 40

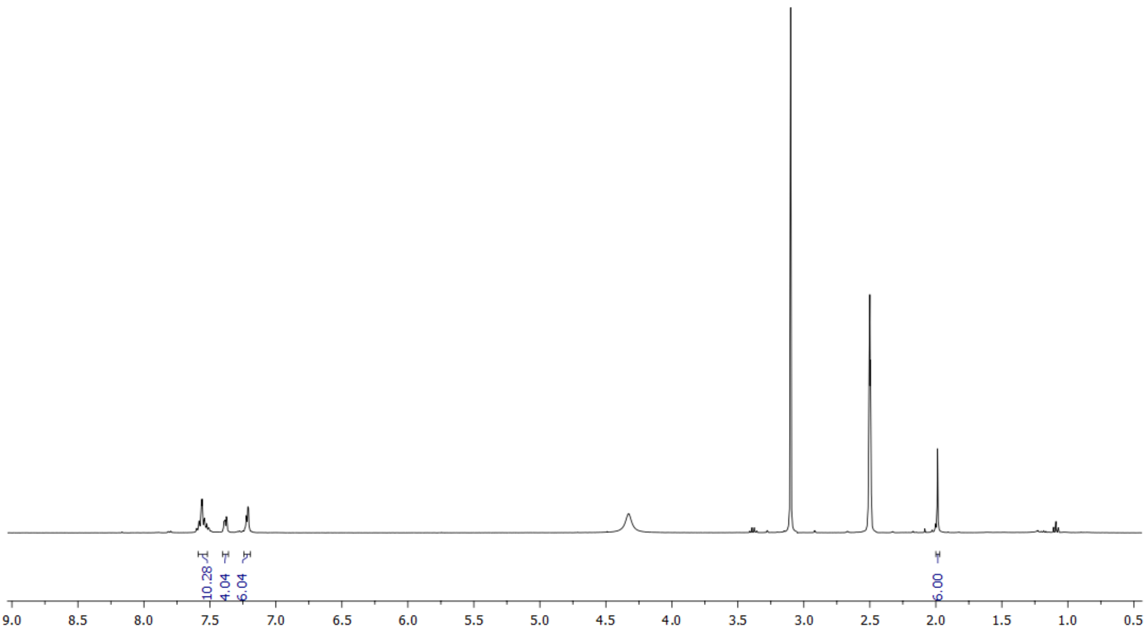

Figure S 122: : ^{1}H-NMR (400 MHz, DMSO-d_6) of compound 40

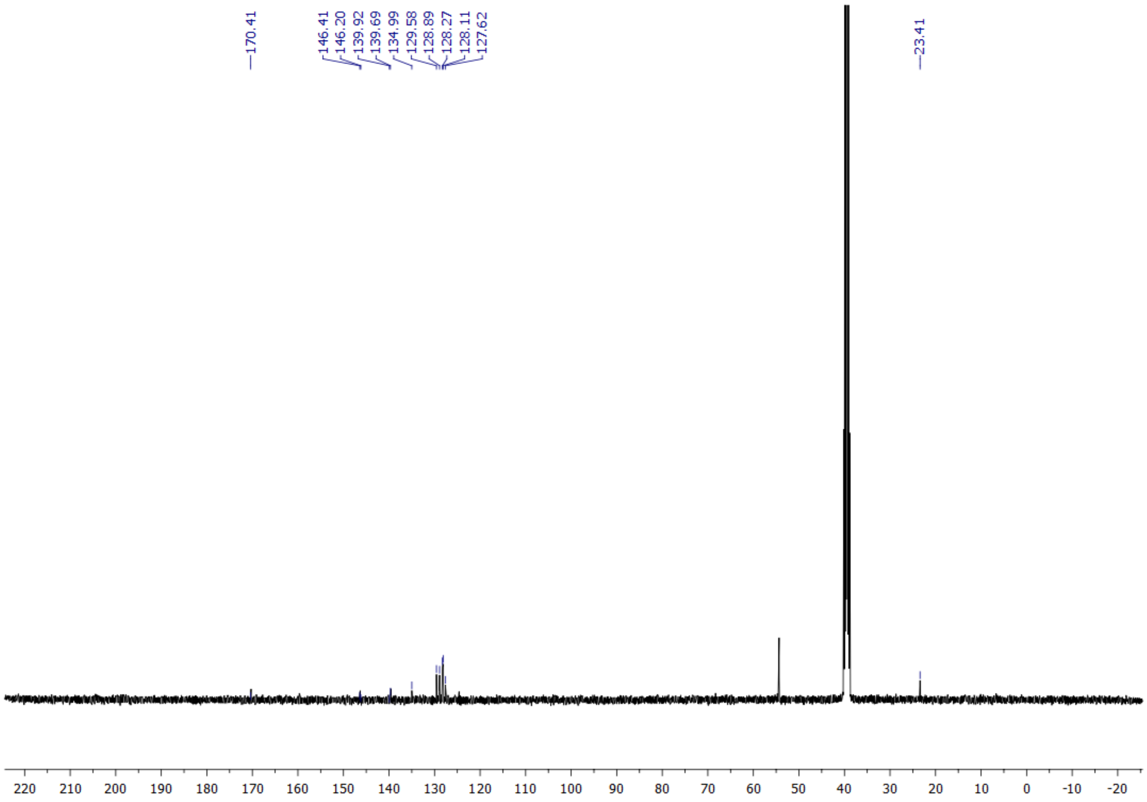

Figure S 123: ^{13}C-NMR (101 MHz, DMSO) of compound 40

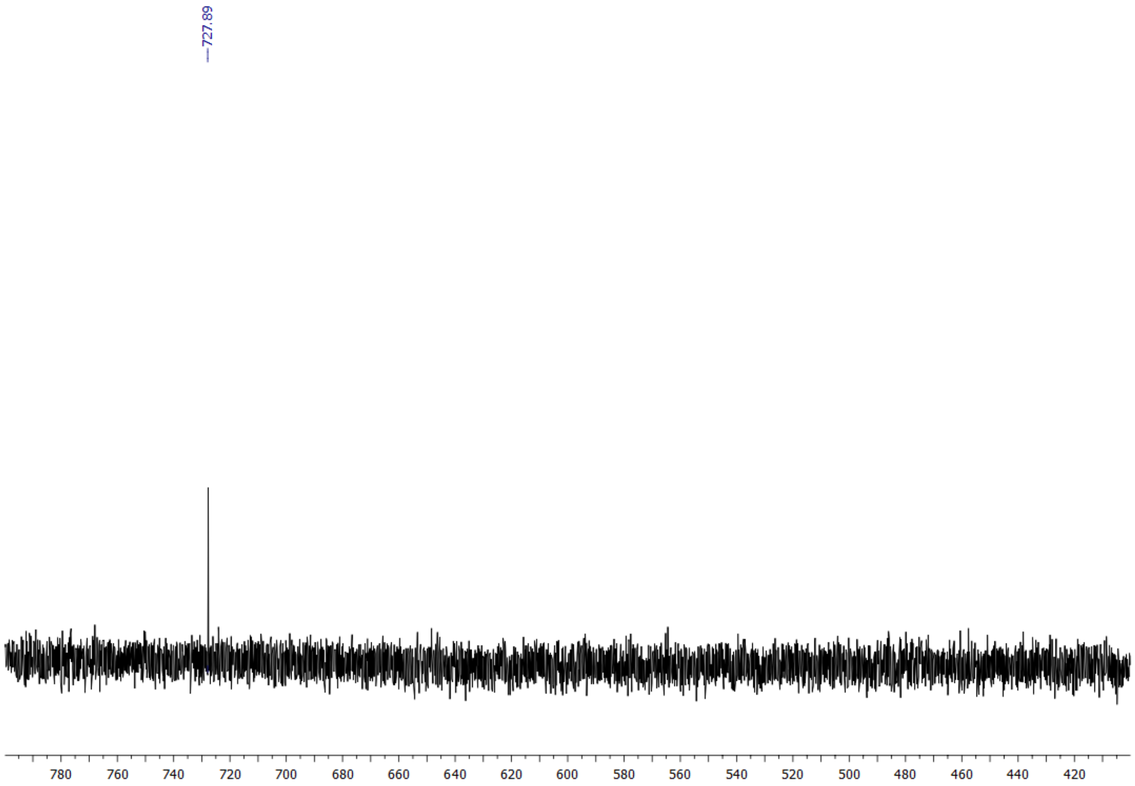

Figure S 124: ^{77}Se-NMR (76 MHz, DMSO) of compound 40

Table S 1: Crystallographic and refinement details

	**1c**	**2a**	**2b**	**2c**	**3**	**5**	**6a**	**9**
Identification code	2082322	2082323	2082320	2082340	2082337	2082329	2082333	2082310
Empirical formula	C_8H_7NOSe	$C_7H_8N_2Se$	$C_8H_{10}N_2Se$	$C_8 H_{10} N_2 O Se$	$C_{15}H_{12}N_2Se$	$C_{16}H_{14}N_2OSe$	$C_{42}H_{34}I_4N_4Se_2$	$C_{10} H_{10} N_2 Se$
Formula weight	212,11	199,11	213,14	229.14	299,23	329.25	1260,25	237.16
Temperature/K	150	149.95(10)	150(2)	100(2)	149.95(10)	293	293(2)	100(2)
Crystal system	triclinic	monoclinic	monoclinic	triclinic	monoclinic	monoclinic	triclinic	monoclinic
Space group	P-1	$P2_1/c$	$P2_1/n$	$P\bar{1}$	$P2_1/n$	$P2_1/c$	P-1	$P2_1/c$
a/Å	6.7205(8)	5.5760(4)	5.4997(2)	6.8563(7)	5.82360(10)	15.743(4)	9.0766(3)	8.9398(5)
b/Å	7.2682(6)	15.3311(12)	8.7494(3)	8.6186(9)	14.8625(4)	14.932(3)	9.3878(3)	13.5971(8)
c/Å	9.6442(10)	8.9604(7)	18.1394(6)	8.9293(9)	28.3043(7)	6.025(2)	26.4138(9)	8.0283(4)
α/°	75.195(8)	90	90	64.562(2)	90	90	85.946(3)	90
β/°	77.439(9)	92.257(7)	95.204(3)	71.488(2)	92.380(2)	92.28(3)	86.233(3)	98.000(3)
γ/°	63.877(10)	90	90	82.846(2)	90	90	82.743(3)	90
Volume/Å^3	405.92(8)	765.40(10)	869.25(5)	451.79(8)	2447.72(10)	1415.1(8)	2223.42(13)	966.39(9)
Z	2	4	4	2	8	4	2	4
ρ_{calc}g/cm^3	1,735	1,728	1,6286	1.684	1,624	1,545	1,882	1.630
μ/mm^{-1}	4,562	4,826	4,255	4.108	3,049	2,650	4,473	4.864
F(000)	208	392	423,8	228	1200	664.0	1188	472
Crystal size/mm^3	0.13 × 0.13 × 0.02	0.07 × 0.05 × 0.03	0.07 × 0.07 × 0.03	0.156 x 0.067 x 0.030	0.1 × 0.1 × 0.07	0.1 7× 0.14 × 0.05	0.1 × 0.06 × 0.04	0.267 x 0.052 x 0.026
2Θ range for data collection/°	6.35 to 58.812	5.268 to 58.732	5.18 to 58.86	2.643 to 33.137	5.118 to 58.77	5.854 to 66.47	4.704 to 58.732	5.964 to 72.062
Reflections collected	3494	6596	4053	13695	13416	7840	22080	17433
Independent reflections	1862 [R_{int} = 0.0245]	1840 [R_{int} = 0.0246]	1979 [R_{int} = 0.0320]	3450 [R_{int} = 0.0300]	5718 [R_{int} = 0.0278]	4808 [R_{int} = 0.0384]	10309 [R_{int} = 0.0268]	1879 [R_{int} = 0.1044]
Data/restraints/parameters	1862/0/101	1840/0/91	1979/0/101	3450 / 0 / 110	5718/0/325	4808/0/182	10309/0/469	1879 / 0 / 124
Goodness-of-fit on F^2	1,023	1,067	1,036	1,073	1,117	1.047	1,027	1,169
Final R indexes [I>=2σ (I)]	R_1 = 0.0279, wR_2 = 0.0587	R_1 = 0.0216, wR_2 = 0.0469	R_1 = 0.0335, wR_2 = 0.0678	R1 = 0.0181, wR2 = 0.0441	R_1 = 0.0363, wR_2 = 0.0754	R_1 = 0.612, wR_2 = 0.1552	R_1 = 0.0441, wR_2 = 0.0945	R1 = 0.0440, wR2 = 0.1097
Final R indexes [all data]	R_1 = 0.0351, wR_2 = 0.0619	R_1 = 0.0269, wR_2 = 0.0487	R_1 = 0.0559, wR_2 = 0.0770	R1 = 0.0204, wR2 = 0.0448	R_1 = 0.0534, wR_2 = 0.0819	R_1 = 0.1130, wR_2 = 0.2038	R_1 = 0.0655, wR_2 = 0.1043	R1 = 0.0737, wR2 = 0.1264
Largest diff. peak/hole / e Å^{-3}	0.42/-0.41	0.33/-0.29	0.42/-0.54	0.6/-0.3	0.47/-0.55	0.74/-0.98	2.35/-1.10	0.9/-1.2

	**10**	**HBr of 11**	**15**	**16**	**20**	**HBr of 20**	**21**	**22**
Identification code	2082330	2082321	2082324	2082331	2082326	2082311	2082325	2082327
Empirical formula	$C_9H_8N_2Se$	$C_{16}H_{15}BrN_2Se$	$C_{20}H_{16}N_2Se$	$C_{14}H_{18}N_2Se$	$C_{17}H_{16}N_2OSe$	$C_{17}H_{17}BrN_2OSe$	$C_{17}H_{16}N_2O_2Se$	$C_{22}H_{18}N_2OSe$
Formula weight	223,13	394,17	363,31	293,26	343,28	424.19	359,28	405,34
Temperature/K	293(2)	293	293	293(2)	293(2)	100(2)	293(2)	293
Crystal system	monoclinic	monoclinic	monoclinic	monoclinic	monoclinic	monoclinic	orthorhombic	monoclinic
Space group	$P2_1/n$	$P2_1/c$	$P2_1/c$	$P2_1/n$	$P2_1/c$	$P2_1/n$	$P2_12_12_1$	$P2_1/c$
a/Å	10.2757(10)	10.1315(7)	18.4368(11)	12.0668(8)	17.1141(12)	12.3164(16)	5.8504(6)	20.0700(15)
b/Å	7.4876(4)	16.5406(6)	15.0933(7)	5.9975(4)	14.9993(11)	8.9214(7)	14.9303(17)	15.2032(6)
c/Å	12.4378(9)	9.9513(7)	5.8505(3)	19.6436(10)	5.8375(3)	15.6592(8)	34.867(4)	5.8117(3)
α/°	90	90	90	90	90	90	90	90
β/°	113.119(10)	108.711(7)	97.977(5)	101.538(6)	90.174(6)	108.748(5)	90	91.309(6)
γ/°	90	90	90	90	90	90	90	90
Volume/$Å^3$	880.12(13)	1579.51(18)	1612.28(15)	1392.90(15)	1498.48(17)	1629.3(3)	3045.5(6)	1772.84(18)
Z	4	4	4	4	4	4	8	4
$\rho_{calc}g/cm^3$	1,684	1,658	1,497	1,398	1,522	1.729	1,567	1,519
μ/mm^{-1}	4,207	4,898	2,329	2,677	2,506	4.760	2,474	2,131
F(000)	440	776	736	600	696	840	1456	824
Crystal size/mm^3	0.06 × 0.04 × 0.01	0.11 × 0.03 × 0.02	0.08 × 0.08 × 0.05	0.1 × 0.07 × 0.03	0.11 × 0.09 × 0.04	0.07 × 0.06 × 0.06	0.08 × 0.05 × 0.03	0.15 × 0.09 × 0.03
2Θ range for data collection/°	6.504 to 58.596	4.908 to 59.098	5.214 to 58.868	6.592 to 66.622	4.76 to 58.97	2.664 to 33.157	4.674 to 59.054	4.864 to 58.87
Reflections collected	4228	7801	8388	7552	12900	33756	10985	9527
Independent reflections	2053 [R_{int} = 0.0252]	3648 [R_{int} = 0.0678]	3757 [R_{int} = 0.0287]	4758 [R_{int} = 0.0327]	3641 [R_{int} = 0.0954]	6206 [R_{int} = 0.0315]	6409 [R_{int} = 0.0267]	4183 [R_{int} = 0.0297]
Data/restraints/parameters	2053/0/109	3648/0/182	3757/0/209	4758/0/158	3641/0/192	6206 / 0 / 201	6409/0/402	4183/0/236
Goodness-of-fit on F^2	1,026	0,967	1,053	1,035	1,041	1.072	1,022	1,049
Final R indexes [I>=2σ (I)]	R_1 = 0.0429, wR_2 = 0.0867	R_1 = 0.0519, wR_2 = 0.1068	R_1 = 0.0448, wR_2 = 0.0869	R_1 = 0.0416, wR_2 = 0.0887	R_1 = 0.0647, wR_2 = 0.1626	R_1 = 0.0219, wR_2 = 0.0504	R_1 = 0.0429, wR_2 = 0.0689	R_1 = 0.0506, wR_2 = 0.0835
Final R indexes [all data]	R_1 = 0.0781, wR_2 = 0.1012	R_1 = 0.1036, wR_2 = 0.1455	R_1 = 0.0720, wR_2 = 0.0981	R_1 = 0.0819, wR_2 = 0.1093	R_1 = 0.1118, wR_2 = 0.2042	R_1 = 0.0281. wR_2 = 0.0530	R_1 = 0.0714, wR_2 = 0.0781	R_1 = 0.1048, wR_2 = 0.1001
Largest diff. peak/hole / e $Å^{-3}$	0.75/-0.54	0.54/-0.62	0.36/-0.33	0.41/-0.49	0.72/-0.56	0.6/-0.8	0.42/-0.36	0.30/-0.45
Flack parameter	-	-	-	-	-	-	0.360(12)	-

	**23**	**24**	**27**	**29**	**30**	**31**	**32**	**33**
Identification code	2082328	2083044	2082336	2082332	2082334	2082335	2082338	2082339
Empirical formula	$C_{20}H_{16}N_2OSe$	$C_{14}H_{18}N_2OSe$	$C_{17}H_{14}N_2OSe$	$C_{16}H_{20}N_2O_2Se$	$C_{17}H_{13}ClN_2OSe$	$C_{17}H_{13}BrN_2OSe$	$C_{17}H_{13}IN_2OSe$	$C_{13}H_{13}BrN_2OSe$
Formula weight	379,31	309,26	341,26	351,3	375,7	420,16	467,15	372,12
Temperature/K	293(2)	150.00(10)	149.95(10)	293(2)	149.95(10)	150.00(14)	300.3(6)	300(1)
Crystal system	monoclinic	monoclinic	monoclinic	monoclinic	orthorhombic	orthorhombic	orthorhombic	triclinic
Space group	$P2_1/c$	$P2_1/n$	$P2_1/c$	$P2_1/c$	Pbca	Pbca	Pbca	P-1
a/Å	18.260(2)	11.5370(7)	13.8857(4)	10.7463(7)	18.6466(10)	8.8441(3)	9.1064(3)	6.9604(4)
b/Å	15.0620(13)	6.1744(3)	13.5842(4)	23.3249(13)	8.6450(5)	18.7452(5)	18.7556(6)	10.2897(7)
c/Å	5.9240(5)	20.2819(11)	7.9397(2)	6.8845(4)	18.9116(11)	18.7293(5)	18.8939(5)	10.3584(6)
α/°	90	90	90	90	90	90	90	102.858(5)
β/°	93.860(10)	104.236(6)	91.449(3)	105.491(6)	90	90	90	98.936(5)
γ/°	90	90	90	90	90	90	90	95.700(5)
Volume/$Å^3$	1625.6(3)	1400.39(14)	1497.16(7)	1662.95(18)	3048.5(3)	3105.03(16)	3227.00(17)	707.55(8)
Z	4	4	4	4	8	8	8	2
$\rho_{calc}g/cm^3$	1,55	1,467	1,514	1,403	1,637	1,798	1,923	1,747
μ/mm^{-1}	2,318	2,672	2,508	2,264	2,641	4,995	4,243	5,467
F(000)	768	632	688	720	1504	1648	1792	364
Crystal size/mm^3	0.1 × 0.08 × 0.02	0.11 × 0.06 × 0.02	0.1 × 0.03 × 0.03	0.14 × 0.04 × 0.03	0.06 × 0.05 × 0.02	0.14 × 0.13 × 0.06	0.06 × 0.06 × 0.03	0.11 × 0.07 × 0.02
2Θ range for data collection/°	5.226 to 59.368	4.612 to 58.74	5.87 to 58.838	5.26 to 58.908	4.83 to 59.012	4.86 to 59.058	4.85 to 58.962	5.984 to 58.82
Reflections collected	9028	7297	7272	8872	10911	8329	10268	6200
Independent reflections	3838 [R_{int} = 0.0351]	3225 [R_{int} = 0.0319]	3472 [R_{int} = 0.0260]	3872 [R_{int} = 0.0329]	3613 [R_{int} = 0.0305]	3528 [R_{int} = 0.0298]	3856 [R_{int} = 0.0397]	3273 [R_{int} = 0.0283]
Data/restraints/parameters	3838/0/218	3225/0/167	3472/0/191	3872/0/195	3613/0/200	3528/0/200	3856/0/200	3273/0/166
Goodness-of-fit on F^2	1,047	1,043	1,067	1,038	1,018	1,077	1,103	1,016
Final R indexes [I>=2σ (I)]	R_1 = 0.0500, wR_2 = 0.1053	R_1 = 0.0340, wR_2 = 0.0732	R_1 = 0.0347, wR_2 = 0.0641	R_1 = 0.0435, wR_2 = 0.0866	R_1 = 0.0281, wR_2 = 0.0598	R_1 = 0.0349, wR_2 = 0.0631	R_1 = 0.0520, wR_2 = 0.1384	R_1 = 0.0428, wR_2 = 0.0872
Final R indexes [all data]	R_1 = 0.0968, wR_2 = 0.1325	R_1 = 0.0500, wR_2 = 0.0802	R_1 = 0.0524, wR_2 = 0.0693	R_1 = 0.0761, wR_2 = 0.1009	R_1 = 0.0423, wR_2 = 0.0651	R_1 = 0.0546, wR_2 = 0.0693	R_1 = 0.0717, wR_2 = 0.1505	R_1 = 0.0734, wR_2 = 0.1005
Largest diff. peak/hole / e $Å^{-3}$	0.35/-0.47	0.53/-0.41	0.33/-0.31	0.32/-0.52	0.45/-0.38	0.58/-0.54	1.24/-1.45	0.51/-0.43
Flack parameter	-	-	-	-	-	-	-	-

	35	36	37	38S	38	39Au	40	1x	1y
Identification code	2082312	2082313	2082315	2082319	2124335	2082316	2082317	2082318	2082314
Empirical formula	$C_{17} H_{15} N_3 O_3 Se$	$C_{25} H_{18} N_2 O Se$	$C_{22} H_{22} N_2 O SeSi$	$C_{20}H_{18}HgN_2O_3S$	$C_{19}H_{16}HgN_2O_3Se$	$C_{22}H_{27}AuCl_5N_3OSe$	$C_{34}H_{26}N_4O_2Se_2$	$C_{20}H_{15}N_3Se$	$C_{20}H_{15}N_3Se_2$
Formula weight	388.28	441.37	437.46	567.01	599.89	802.64	680.51	376.31	455.27
Temperature/K	100(2)	100(2)	150.00(10)	149.99(10)	100(2)	150.00(10)	150.00(10)	149.99(10)	100(2)
Crystal system	monoclinic	monoclinic	monoclinic	monoclinic	orthorhombic	triclinic	orthorhombic	monoclinic	monoclinic
Space group	$P2_1/n$	$P2_1/c$	$P2_1/c$	$P2_1/n$	$Pna2_1$	P-1	Fdd2	$P2_1/c$	$P2_1/c$
a/Å	6.5934(5)	10.4198(3)	9.7971(14)	16.6567(6)	6.707(6)	9.3257(3)	32.8581(13)	9.1913(5)	5.6550(7)
b/Å	15.4919(13)	20.7557(7)	9.649(5)	5.30189(19)	37.73(2)	15.8788(5)	19.1605(7)	15.8059(9)	21.4410(13)
c/Å	16.150(4)	9.4600(3)	22.573(5)	21.3913(11)	21.564(13)	19.2008(5)	9.2637(4)	11.5894(6)	14.4120(10)
α/°	90	90	90	90	90	89.872(2)	90	90	90
β/°	100.715(13)	96.5460(10)	94.575(18)	93.073(4)	90	87.129(2)	90	101.324(5)	94.671(5)
γ/°	90	90	90	90	90	82.845(2)	90	90	90
Volume/Å^3	1620.9(4)	2032.58(11)	2127.1(12)	1088.0	5456(7)	2817.57(15)	5832.2(4)	1650.89(16)	1741.6(3)
Z	4	4	4	4	12	4	8	4	4
ρ_{calc}g/cm^3	1.591	1.442	1.366	1.997	2.191	1.892	1.550	1.514	1.736
μ/mm^{-1}	2.337	1.866	1.835	8.293	7.401	7.008	2.575	2.280	2.954
F(000)	784	896	896	1088.0	3384.0	1544.0	2736	760.0	896.0
Crystal size/mm^3	0.16 x 0.02 x 0.01	0.118 × 0.072 × 0.041	0.12 × 0.04 × 0.02	0.09×0.07×0.02	0.05 × 0.025 × 0.01	0.06 × 0.05 × 0.02	0.08 × 0.07 × 0.06	0.13 × 0.06 × 0.05	0.06 × 0.01 × 0.01
2Θ range for data collection/°	2.884 to 32.029	2.779 to 31.225	5.302 to 58.682	6.046 to 59.094	0.942 to 25.122	4.796 to 58.92	5.04 to 70.148	5.204 to 58.486	2.976 to 53.808
Reflections collected	17003	54159	11082	10250	91571	27875	11284	8150	36538
Independent reflections	5623 [R_{int} = 0.0690]	6553 [R_{int} = 0.0353]	4978 [R_{int} = 0.0371]	10250 [R_{int} = 0.0543]	14592 [R_{int} = 0.0584]	12825 [R_{int} = 0.0295]	4827 [R_{int} = 0.0356]	3810 [R_{int} = 0.0219]	5668 [R_{int} = 0.0441]
Data/restraints/parameters	5623/0/220	6553/0/263	4978/0/248	10250/0/248	14592/1/709	12825/56/646	4827/1/191	3810/0/217	5668/0/226
Goodness-of-fit on F^2	1,034	1,159	1.045	1.045	1.042	1.027	1.042	1.024	1.054
Final R indexes [I>=2σ (I)]	R_1 = 0.0545, wR_2 = 0.1099	R_1 = 0.0445, wR_2 = 0.1187	R_1 = 0.0402. wR_2 = 0.0894	R_1 = 0.0392, wR_2 = 0.0893	R_1 = 0.0406. wR_2 = 0.1079	R_1 = 0.0306. wR_2 = 0.0641	R_1 = 0.0300. wR_2 = 0.0640	R_1 = 0.0284. wR_2 = 0.0605	R_1 = 0.0264. wR_2 = 0.0619
Final R indexes [all data]	R_1 = 0.0955,wR_2 = 0.1261	R_1 = 0.0551, wR_2 = 0.1224	R_1 = 0.0666. wR_2 = 0.1017	R_1 = 0.0555, wR_2 = 0.0926	R_1 = 0.0437. wR_2 = 0.1098	R_1 = 0.0426. wR_2 = 0.0689	R_1 = 0.0373. wR_2 = 0.0668	R_1 = 0.0374. wR_2 = 0.0643	R_1 = 0.0313. wR_2 = 0.0645
Largest diff. peak/hole / e Å^{-3}	0.6 /-1.6	1.9/-0.7	0.70/-0.48	1.48/-1.27	3.8/-1.2	2.25/-1.28	0.63/-0.28	0.35/-0.35	0.65/-0.76
Flack parameter	-	-	-	-	-	-	0.012(6)	-	-

Experimental details of the X-ray structure determinations

Diffraction data were collected at 150 K using a Rigaku Oxford Diffraction Gemini E Ultra diffractometer, equipped with an EOS CCD area detector and a four-circle kappa goniometer. For the data collection, the Mo source emitting graphite-monochromated Mo-Kα radiation (λ = 0.71073 Å) was used. Data integration, scaling and empirical absorption correction was carried out using the CrysAlis Pro program package [1]. Crystals of compound **38** were very small. Data was therefore collected at beamline P11 with an energy of 20 keV (λ = 0.619900 Å) at PETRA III DESY Hamburg. For data integration XDS [2] and for scaling as well as absorption correction SADABS was used [3]. The structures were solved using Direct Methods and refined by Full-Matrix-Least-Squares against F2 using SHELXT [4] and SHELXL [5]. Non-hydrogen atoms were refined anisotropically and hydrogen atoms were placed at idealized positions and refined using the riding model. All calculations were carried out using the Olex2 graphical interface [6].

References

1. *CrysAlisPro 40.53*, 40.53; Rigaku Oxford Diffraction Ltd.: Oxford (U.K.), 2019.
2. Kabsch, W. XDS. *Acta Cryst.* **2010**, *D66*, 125-132.
3. Sheldrick, G.M. *SADABS 2.03*; Universiät Göttingen, Germany: 2002.
4. Sheldrick, G.M. SHELXT - Integrated space-group and crystal-structure determination. *Acta Cryst.* **2015**, *A71*, 3-8.
5. Sheldrick, G.M. Crystal structure refinement with SHELXL. *Acta Cryst.* **2015**, *C71*, 3-8.
6. Dolomanov, O.V.; Bourhis, L.J.; Gildea, R.J.; Howard, J.A.K.; Puschmann, H. OLEX2: a complete structure solution, refinement and analysis program. *J. Appl. Cryst.* **2009**, *42*, 339-341.

Hit-Confirmation

Antimicrobial screening, Cytotoxicity & Haemolysis

Procedure and Materials

Content

1.0 Summary ... 3
1.1 Study ... 3
1.2 Assay Parameters ... 3
1.3 Outcomes ... 3
1.4 Structural Novelty ... 4
1.5 Publishing CO-ADD Data ... 4
2.0 Methods ... 5
2.1 Sample Preparation ... 5
2.2 Antibacterial Assay ... 5
2.2.1 Procedure ... 5
2.2.2 Analysis ... 5
2.3 Antifungal Assay ... 6
2.3.1 Procedure ... 6
2.3.2 Analysis ... 6
2.4 Cytotoxicity Assay ... 7
2.4.1 Procedure ... 7
2.4.2 Analysis ... 7
2.5 Haemolysis Assay ... 7
2.5.1 Procedure ... 7
2.5.2 Analysis ... 7
2.6 Antibiotic, Cytotoxic and Haemolytic Standards Preparation and Quality Control ... 8
3.0 Materials ... 9
3.1 Assay Materials ... 9
3.2 Standards ... 9
3.3 Microbial strains and cell lines ... 9
4.0 Controls ... 10
4.1 Antimicrobial susceptibility of tested strains ... 10
4.1.1 Antibiotic standards ... 10
4.1.2 Antifungal standard ... 10
4.2 Susceptibility profile of cell lines ... 10
4.3 Susceptibility profile of human washed red cells ... 11
4.4 Outcome ... 11

1.0 Summary

1.1 Study

Hit Confirmation of active compounds by whole cell growth inhibition assays was conducted as an 8-point dose response to determine the Minimum Inhibitory Concentration (MIC), in duplicate (n=2). The inhibition of growth is measured against those microorganisms that showed susceptibility to the compounds tested in the Primary Screen.

Included in the Hit Confirmation were 5 bacteria: *Escherichia coli*, *Klebsiella pneumoniae, Acinetobacter baumannii, Pseudomonas aeruginosa* and *Staphylococcus aureus*, and 2 fungi *Candida albicans* and *Cryptococcus neoformans*.

In addition to determining MIC, active compounds were counter screened for cytotoxicity against a human embryonic kidney cell line, HEK293, by determining their CC_{50} value. The compounds were also screened for haemolysis of human red blood cells.

1.2 Assay Parameters

Assay Parameters	Bacteria	Fungi	HEK293	Haemolysis
Test concentration	32 - 0.25 µg/mL or 20 – 0.15 µM ≤0.5% DMSO	32 - 0.25 µg/mL or 20 – 0.15 µM ≤0.5% DMSO	32 - 0.25 µg/mL or 20 – 0.15 µM ≤0.5% DMSO	32 - 0.25 µg/mL or 20 – 0.15 µM ≤0.5% DMSO
QC	Duplicate (n=2) Control MIC: Pass	Duplicate (n=2) Control MIC: Pass	Duplicate (n=2) Control CC_{50}: Pass	Duplicate (n=2) Control HC_{10}: Pass
Plates	Non-Binding Surface (NBS), 384-well plate	Non-Binding Surface (NBS), 384-well plate	TC, 384-well black wall/clear bottom	Polypropylene 384-well and polystyrene 384-well plates
Media	Cation-adjusted Mueller Hinton broth	Yeast Nitrogen Base	DMEM supplemented with 10% FBS	0.9% NaCl
Read Out	OD_{600}	OD_{630} Resazurin $OD_{600-570}$	Resazurin $F_{560/590}$	OD_{405}

1.3 Outcomes

Hit Confirmation outcomes are detailed in individual Project reports, personalised for each Project Submission for each CO-ADD user.

Please see your data sheet with file extension **P0XXX_HC_data.xlsx,** for example CO-ADD Project **P0100, P0100_HC_data.xlsx**

1.4 Structural Novelty

As per the T&C's of CO-ADD, structures for all submitted compounds for antimicrobial screening should be disclosed to CO-ADD following Primary Screening. Without structures for all submitted compounds, Hit Confirmation assays will not be triggered.

If you have not already done so, please **provide CO-ADD with the chemical structure** of the full sample set in this study (both for compounds showing activity and those that do not), which will allow CO-ADD to filter out future samples with the same, or highly similar structure. In addition, please **notify CO-ADD** if you agree to publish the data (*i.e.* structures and activity) in the public bioactive database ChEMBL (www.ebi.ac.uk/chembl/). CO-ADD aims to increase the public knowledge of antimicrobial research, including data about non-active compounds.

All confirmed hits, without cytotoxicity or haemolytic activity, will be considered for further Hit-Validation, after a detailed analysis of structure-activity relationship and antimicrobial novelty, within CO-ADD samples, as well as, within public antimicrobial activity databases, like ChEMBL (www.ebi.ac.uk/chembl/).

1.5 Publishing CO-ADD Data

If you wish to publish data provided by CO-ADD, we kindly ask that you acknowledge CO-ADD appropriately with the following reference:

Helping Chemists Discover New Antibiotics
M.A. Blaskovich, J. Zuegg, A.G. Elliott, M.A. Cooper
ACS Infect. Dis., **2015**, 1(7), 285-287.
DOI: 10.1021/acsinfecdis.5b00044; PMID: 27622818

as well as an acknowledgement for the funding of CO-ADD:

"The antimicrobial screening performed by CO-ADD (The Community for Antimicrobial Drug Discovery) was funded by the Wellcome Trust (UK) and The University of Queensland (Australia)."

Please advise CO-ADD at your earliest convenience that you have used provided data for publication purposes. This information is extremely helpful in keeping track of the outputs from the CO-ADD initiative and supports the program in renewed funding possibilities to continue CO-ADD as a free screening service available to the academic community.

CO-ADD also asks, that where possible you publish your data in an Open Access journals.

2.0 Methods

2.1 Sample Preparation

Samples were provided by the collaborator and stored frozen at -20 °C. Samples were prepared in DMSO and water to a final testing concentration of 32 µg/mL or 20 µM (unless otherwise indicated in the data sheet) and serially diluted 1:2 fold for 8 times. Each sample concentration was prepared in 384-well plates, non-binding surface plate (**NBS;** Corning 3640) for each bacterial/fungal strain, tissue-culture treated (**TC-treated;** Corning 3712/3764) black for mammalian cell types and polypropylene 384-well (**PP**; Corning 3657) for haemolysis assays, all in duplicate (n=2), and keeping the final DMSO concentration to a maximum of 0.5%. All the sample preparation was done using liquid handling robots.

Compounds that showed notable solubility issues during stock solution preparation are detailed in the **Data sheet** for the individual Project.

2.2 Antibacterial Assay

2.2.1 Procedure

All bacteria were cultured in Cation-adjusted Mueller Hinton broth (**CAMHB**) at 37 °C overnight. A sample of each culture was then diluted 40-fold in fresh broth and incubated at 37 °C for 1.5-3 h. The resultant mid-log phase cultures were diluted (CFU/mL measured by OD_{600}), then added to each well of the compound containing plates, giving a cell density of 5×10^5 CFU/mL and a total volume of 50 µL. All the plates were covered and incubated at 37 °C for 18 h without shaking.

2.2.2 Analysis

Inhibition of bacterial growth was determined measuring absorbance at 600 nm (OD_{600}), using a Tecan M1000 Pro monochromator plate reader. The percentage of growth inhibition was calculated for each well, using the negative control (media only) and positive control (bacteria without inhibitors) on the same plate as references.

The percentage of growth inhibition was calculated for each well, using the negative control (media only) and positive control (bacteria without inhibitors) on the same plate. The MIC was determined as the lowest concentration at which the growth was fully inhibited, defined by an inhibition ≥ 80%. In addition, the maximal percentage of growth inhibition is reported as D_{Max}, indicating any compounds with partial activity.

Hits were classified by MIC ≤ 16 µg/mL or MIC ≤ 10 µM in either replicate (n=2 on different plates).

2.3 Antifungal Assay

2.3.1 Procedure

Fungi strains were cultured for 3 days on Yeast Extract-Peptone Dextrose (**YPD**) agar at 30 °C. A yeast suspension of 1 x 10^6 to 5 x 10^6 CFU/mL (as determined by OD_{530}) was prepared from five colonies. The suspension was subsequently diluted and added to each well of the compound-containing plates giving a final cell density of fungi suspension of 2.5 $\times 10^3$ CFU/mL and a total volume of 50 µL. All plates were covered and incubated at 35 °C for 36 h without shaking.

2.3.2 Analysis

Growth inhibition of *C. albicans* was determined measuring absorbance at 630 nm (OD_{630}), while the growth inhibition of *C. neoformans* was determined measuring the difference in absorbance between 600 and 570 nm ($OD_{600-570}$), after the addition of resazurin (0.001% final concentration) and incubation at 35 °C for 2 h. The absorbance was measured using a Biotek Multiflo Synergy HTX plate reader.

In both cases, the percentage of growth inhibition was calculated for each well, using the negative control (media only) and positive control (fungi without inhibitors) on the same plate. The MIC was determined as the lowest concentration at which the growth was fully inhibited, defined by an inhibition ≥ 80% for *C. albicans* and an inhibition ≥ 70% for *C. neoformans.* Due to a higher variance in growth and inhibition, a lower threshold was applied to the data for *C. neoformans*. In addition, the maximal percentage of growth inhibition is reported as D_{Max}, indicating any compounds with marginal activity.

Hits were classified by MIC ≤ 16 µg/mL or MIC ≤ 10 µM in either replicate (n=2 on different plates).

2.4 Cytotoxicity Assay

2.4.1 Procedure

HEK293 cells were counted manually in a Neubauer haemocytometer and then plated in the 384-well plates containing the compounds to give a density of 5000 cells/well in a final volume of 50 µL. **DMEM** supplemented with **10% FBS** was used as growth media and the cells were incubated together with the compounds for 20 h at 37 °C in 5% CO_2.

2.4.2 Analysis

Cytotoxicity (or cell viability) was measured by fluorescence, ex: 560/10 nm, em: 590/10 nm ($F_{560/590}$), after addition of 5 µL of 25 µg/mL resazurin (2.3 µg/mL final concentration) and after incubation for further 3 h at 37 °C in 5% CO_2. The fluorescence intensity was measured using a Tecan M1000 Pro monochromator plate reader, using automatic gain calculation.

CC_{50} (concentration at 50% cytotoxicity) were calculated by curve fitting the inhibition values *vs.* log(concentration) using a sigmoidal dose-response function, with variable fitting values for bottom, top and slope. In addition, the maximal percentage of cytotoxicity is reported as D_{Max}, indicating any compounds with partial cytotoxicity.

The curve fitting was implemented using Pipeline Pilot's dose-response component, resulting in similar values to curve fitting tools such as GraphPad's Prism and IDBS's XlFit. Any value with > indicate sample with no activity (low D_{Max} value) or samples with CC_{50} values above the maximum tested concentration (higher D_{Max} value).

Cytotoxic samples were classified by $CC_{50} \leq 32$ µg/mL or $CC_{50} \leq 10$ µM in either replicate (n=2 on different plates). In addition, samples were flagged as partial cytotoxic if $D_{Max} \geq 50\%$, even with CC_{50} > the maximum tested concentration.

2.5 Haemolysis Assay

2.5.1 Procedure

Human whole blood was washed three times with 3 volumes of 0.9% NaCl and then resuspended in same to a concentration of 0.5 x 10^8 cells/mL, as determined by manual cell count in a Neubauer haemocytometer. The washed cells were then added to the 384-well compound-containing plates for a final volume of 50 µL. After a 10 min shake on a plate shaker the plates were then incubated for 1 h at 37 °C. After incubation, the plates were centrifuged at 1000*g* for 10 min to pellet cells and debris, 25 µL of the supernatant was then transferred to a polystyrene 384-well assay plate.

2.5.2 Analysis

Haemolysis was determined by measuring the supernatant absorbance at 405 mm (OD_{405}). The absorbance was measured using a Tecan M1000 Pro monochromator plate reader.

HC_{10} and HC_{50} (concentration at 10% and 50% haemolysis, respectively) were calculated by curve fitting the inhibition values *vs*. log(concentration) using a sigmoidal dose-response function with variable fitting values for top, bottom and slope. In addition, the maximal

percentage of haemolysis is reported as D_{Max}, indicating any compounds with partial haemolysis.

The curve fitting was implemented using Pipeline Pilot's dose-response component, resulting in similar values to curve fitting tools such as GraphPad's Prism and IDBS's XlFit. Any value with > indicate sample with no activity (low D_{Max} value) or samples with HC_{10} values above the maximum tested concentration (higher D_{Max} value).

Haemolysis samples were classified by HC_{10} ≤ 32 µg/mL or HC_{10} ≤ 10 µM in either replicate (n=2 on different plates). In addition, samples were flagged as partial haemolytic if D_{Max} ≥ 50%, even with HC_{10} > the maximum tested concentration.

2.6 Antibiotic, Cytotoxic and Haemolytic Standards Preparation and Quality Control

Colistin and Vancomycin were used as positive bacterial inhibitor standards for Gram-negative and Gram-positive bacteria, respectively. Fluconazole was used as a positive fungal inhibitor standard for *C. albicans and C. neoformans.* Tamoxifen was used as a positive cytotoxicity standard. Melittin was used as a positive haemolytic standard.

Each antibiotic standard was provided in 4 concentrations, with 2 above and 2 below its MIC or CC_{50} value, and plated into the first 8 wells of column 23 of the 384-well NBS plates. Tamoxifen and melittin was used in 8 concentrations in 2 fold serial dilutions with 50 µg/mL highest concentration.

The quality control (QC) of the assays was determined by Z'-Factor, calculated from the Negative (media only) and Positive Controls (bacterial, fungal or cell culture without inhibitor), and the Standards. Plates with a Z'-Factor of ≥ 0.4 and Standards active at the highest and inactive at the lowest concentration, were accepted for further data analysis.

3.0 Materials

3.1 Assay Materials

Material	*Code*	*Brand/Supplier*	*Cat No.*
Compound preparation plate, Polypropylene	PP	Corning	3364
Assay Plates – Antimicrobial Non-binding surface	NBS 384w	Corning	3640
Assay Plates – Cytotoxicity Tissue culture treated	Black/Clear bottom 384w	Corning	3712
Assay Plates - Haemolysis	PP-Haem	Corning	3657
Reading Plates - Haemolysis	Clear 384w	Corning	3680
Growth media - bacteria	CAMHB	Bacto Laboratories	212322
Culture agar - fungi	YPD	Becton Dickinson	242720
Growth media - fungi	YNB	Becton Dickinson	233520
Resazurin		Sigma-Aldrich	R7017
Dulbecco's Modified Eagle Medium	DMEM	Life Technologies	11995-073
Foetal Bovine Serum	FBS	Bovogen	FFBS-500
0.9% NaCl	Saline	Baxter	AHF7124

3.2 Standards

Sample Name	*Sample ID*	*Full MW*	*Stock Conc. (mg/mL)*	*Solvent*	*Source*
Colistin - Sulfate	MCC_000094:02	1400.63	10.0	DMSO	Sigma; C4461
Vancomycin - HCL	MCC_000095:02	1485.71	10.0	DMSO	Sigma; 861987
Fluconazole	MCC_008383:01	306.27	2.56	DMSO	Sigma; F8929
Tamoxifen	MCC_000096:01	371.50	10	DMSO	Sigma; T5648
Melittin	MCC_008868:02	2846.46	10	Water	Sigma: M2272

3.3 Microbial strains and cell lines

ID	*Batch*	*Organism*	*Strain*	*Description*
GN_001	02	*Escherichia coli*	ATCC 25922	FDA control strain
GN_003	02	*Klebsiella pneumoniae*	ATCC 700603	MDR
GN_034	02	*Acinetobacter baumannii*	ATCC 19606	Type strain
GN_042	02	*Pseudomonas aeruginosa*	ATCC 27853	Quality control strain
GP_020	02	*Staphylococcus aureus*	ATCC 43300	MRSA
FG_001	01	*Candida albicans*	ATCC 90028	CLSI reference
FG_002	01	*Cryptococci neoformans*	ATCC 208821	H99, Type strain
MA_007	02	*Homo sapiens* embryonic kidney cells	ATCC CRL-1573	HEK 293
HA_150	-	*Homo sapiens*	ARCBS 5400 00150	Whole blood

4.0 Controls

4.1 Antimicrobial susceptibility of tested strains

Values are the average of ≥ 6 independent biological replicates. All values are within the expected range as per CLSI guidelines.

4.1.1 Antibiotic standards

MIC determined by BMD method, CA-MHB, Corning 3640 384 NBS plates		**GN_001:02** ***Escherichia coli*** FDA Control ATCC 25922	**GN_003:02** ***Klebsiella pneumophila*** ESBL ATCC 700603	**GN_034:02** ***Acinetobacter baumannii*** Type strain ATCC 19606	**GN_042:02** ***Pseudomonas aeruginosa*** QC strain ATCC 27853
Compound	Compound Type	MIC (µg/mL)			
Colistin - sulfate	Antibiotic	**0.125**	**0.25**	**0.25**	**0.25**

MIC determined by BMD method, CA-MHB, Corning 3640 384 NBS plates		**GP_020:02** ***Staphylococcus aureus*** MRSA ATCC 43300
Compound	Compound Type	MIC (µg/mL)
Vancomycin - HCl	Antibiotic	**1**

4.1.2 Antifungal standard

MIC determined by BMD method, YNB, Corning 3640 384 NBS plates		**FG_001:02** ***Candida albicans*** CLSI reference ATCC 90028	**FG_002:02** ***Cryptococcus neoformans H99*** Type strain ATCC 208821
Compound	Compound Type	MIC (µg/mL)	
Fluconazole	Antifungal	**0.125**	**8**

4.2 Susceptibility profile of cell lines

Values are the average of > 6 independent biological replicates. CC_{50} is the concentration at 50% cytotoxicity.		**MA_007 HEK293 ATCC CRL-1573**	
		CC_{50} (µg/mL)	
Compound	Compound Type	Average	Stdev
Tamoxifen	PKC inhibitor	**9**	**2.2**

4.3 Susceptibility profile of human washed red cells

Values are the average of > 6 independent biological replicates. HC_{10} and HC_{50} are the concentrations at 10% and 50% haemolysis, respectively.		**HA_150 Human Whole blood ARCBS 00150**			
		HC_{10} (μg/mL)		HC_{50} (μg/mL)	
Compound	Compound Type	Average	Stdev	Average	Stdev
Melittin	Haemolytic peptide	**2.7**	**0.9**	**8.5**	**2.5**

4.4 Outcome

All standard compound controls displayed inhibitory values within the expected range for each assay type and each organism tested. For further information please contact the CO-ADD team at support@co-add.org.

Strain ID	***Species***	***Standard positive inhibitor control***	***Pass/Fail***
GN_001:02	*E. coli*	Colistin	Pass
GN_003:02	*K. pneumoniae* (MDR)	Colistin	Pass
GN_034:02	*A. baumannii*	Colistin	Pass
GN_042:02	*P. aeruginosa*	Colistin	Pass
GP_020:02	*S. aureus* (MRSA)	Vancomycin	Pass
FG_001:01	*C. albicans*	Fluconazole	Pass
FG_002:01	*C. neoformans* (H99)	Fluconazole	Pass
MA_007:02	*Homo sapiens* HEK293	Tamoxifen	Pass
HA_150	*Homo sapiens*	Melittin	Pass

Developmental Therapeutics Program<br>One Dose Mean Graph	NSC: D-811317 / 1	Conc: 1.00E-5 Molar	Test Date: Dec 17, 2018
	Experiment ID: 1812OS48		Report Date: Jan 14, 2019

Panel/Cell Line	Growth Percent
**Leukemia**	
CCRF-CEM	79.91
HL-60(TB)	100.45
K-562	90.03
MOLT-4	72.12
RPMI-8226	87.67
SR	88.43
**Non-Small Cell Lung Cancer**	
A549/ATCC	94.81
EKVX	79.22
HOP-62	65.67
HOP-92	76.55
NCI-H226	87.41
NCI-H23	87.99
NCI-H322M	80.66
NCI-H460	97.56
NCI-H522	70.28
**Colon Cancer**	
COLO 205	108.42
HCC-2998	102.47
HCT-116	87.45
HCT-15	95.13
HT29	101.88
KM12	90.66
SW-620	99.21
**CNS Cancer**	
SF-268	97.83
SF-295	89.03
SF-539	98.19
SNB-19	96.16
SNB-75	88.47
U251	92.23
**Melanoma**	
LOX IMVI	97.89
MALME-3M	92.97
M14	94.79
MDA-MB-435	100.13
SK-MEL-2	93.34
SK-MEL-28	108.03
SK-MEL-5	102.68
UACC-257	101.46
UACC-62	83.91
**Ovarian Cancer**	
IGROV1	82.69
OVCAR-3	106.33
OVCAR-4	79.15
OVCAR-5	96.73
OVCAR-8	97.93
NCI/ADR-RES	101.12
SK-OV-3	99.95
**Renal Cancer**	
786-0	101.40
A498	70.63
ACHN	96.53
CAKI-1	58.36
SN12C	91.38
TK-10	121.05
UO-31	66.10
**Prostate Cancer**	
PC-3	89.80
DU-145	102.51
**Breast Cancer**	
MCF7	75.92
MDA-MB-231/ATCC	70.16
HS 578T	95.59
BT-549	96.33
T-47D	61.88
MDA-MB-468	80.54
Mean	90.22
Delta	31.86
Range	62.69

Mean Growth Percent - Growth Percent

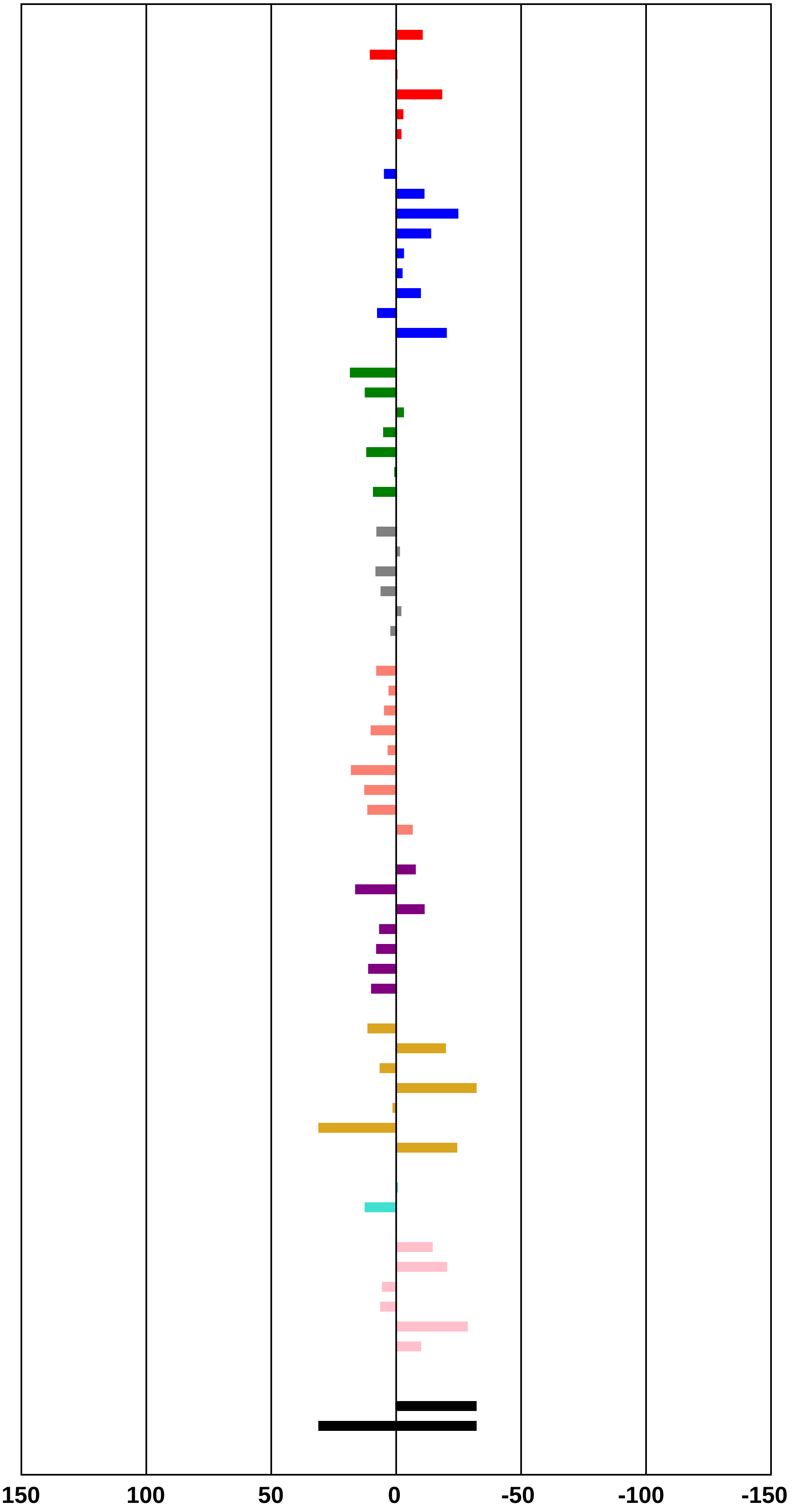

Developmental Therapeutics Program	NSC: D-811318 / 1	Conc: 1.00E-5 Molar	Test Date: Dec 17, 2018
One Dose Mean Graph	Experiment ID: 1812OS48		Report Date: Jan 14, 2019

Panel/Cell Line	Growth Percent
Leukemia	
CCRF-CEM	95.46
HL-60(TB)	100.73
K-562	91.18
MOLT-4	85.52
RPMI-8226	86.49
SR	89.72
Non-Small Cell Lung Cancer	
A549/ATCC	92.17
EKVX	76.66
HOP-62	69.99
HOP-92	68.68
NCI-H226	86.09
NCI-H23	88.19
NCI-H322M	79.16
NCI-H460	105.03
NCI-H522	62.84
Colon Cancer	
COLO 205	105.60
HCC-2998	99.04
HCT-116	84.50
HCT-15	93.76
HT29	89.03
KM12	88.63
SW-620	98.80
CNS Cancer	
SF-268	91.96
SF-295	96.19
SF-539	89.45
SNB-19	98.32
SNB-75	82.44
U251	88.52
Melanoma	
LOX IMVI	93.37
MALME-3M	88.89
M14	97.24
MDA-MB-435	110.24
SK-MEL-2	100.59
SK-MEL-28	104.10
SK-MEL-5	90.15
UACC-257	99.03
UACC-62	70.31
Ovarian Cancer	
IGROV1	74.85
OVCAR-3	108.82
OVCAR-4	82.82
OVCAR-5	95.84
OVCAR-8	97.79
NCI/ADR-RES	96.00
SK-OV-3	90.22
Renal Cancer	
786-0	92.05
A498	63.24
ACHN	85.54
CAKI-1	62.86
SN12C	91.20
TK-10	65.53
UO-31	50.75
Prostate Cancer	
PC-3	84.46
DU-145	94.10
Breast Cancer	
MCF7	86.30
MDA-MB-231/ATCC	66.20
HS 578T	95.87
BT-549	93.83
T-47D	60.79
MDA-MB-468	96.75
Mean	87.69
Delta	36.94
Range	59.49

Mean Growth Percent - Growth Percent

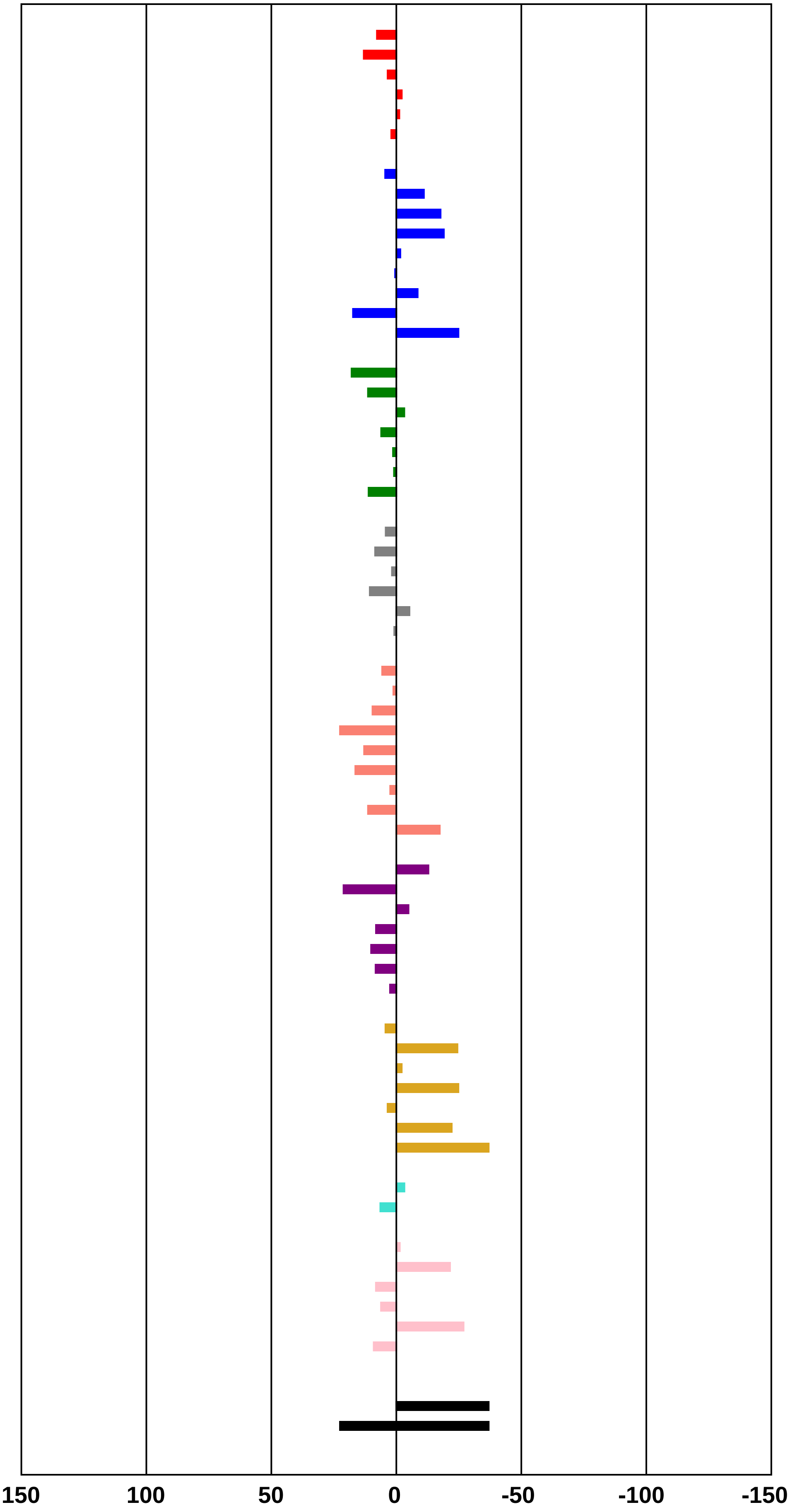

Developmental Therapeutics Program

One Dose Mean Graph

NSC: D-811319 / 1	Conc: 1.00E-5 Molar	Test Date: Dec 17, 2018
Experiment ID: 1812OS48		Report Date: Jan 14, 2019

Panel/Cell Line	Growth Percent
Leukemia	
CCRF-CEM	109.90
HL-60(TB)	99.66
K-562	101.00
MOLT-4	97.31
RPMI-8226	90.40
SR	91.23
Non-Small Cell Lung Cancer	
A549/ATCC	89.21
EKVX	95.07
HOP-62	58.75
HOP-92	104.87
NCI-H226	99.92
NCI-H23	91.70
NCI-H322M	67.94
NCI-H460	87.60
NCI-H522	78.32
Colon Cancer	
COLO 205	121.09
HCC-2998	96.89
HCT-116	100.72
HCT-15	94.79
HT29	85.75
KM12	96.33
SW-620	103.65
CNS Cancer	
SF-268	102.83
SF-295	50.21
SF-539	94.59
SNB-19	91.57
SNB-75	85.72
U251	98.69
Melanoma	
LOX IMVI	100.28
MALME-3M	89.27
M14	100.95
MDA-MB-435	96.66
SK-MEL-2	111.00
SK-MEL-28	101.51
SK-MEL-5	101.82
UACC-257	95.66
UACC-62	93.15
Ovarian Cancer	
IGROV1	98.39
OVCAR-3	109.27
OVCAR-4	91.26
OVCAR-5	99.43
OVCAR-8	103.39
NCI/ADR-RES	96.55
SK-OV-3	104.18
Renal Cancer	
786-0	81.53
A498	77.19
ACHN	103.94
CAKI-1	87.93
SN12C	97.51
TK-10	89.72
UO-31	89.94
Prostate Cancer	
PC-3	94.60
DU-145	107.34
Breast Cancer	
MCF7	88.42
MDA-MB-231/ATCC	97.68
HS 578T	104.13
BT-549	107.71
T-47D	94.02
MDA-MB-468	90.67
Mean	94.76
Delta	44.55
Range	70.88

Mean Growth Percent - Growth Percent

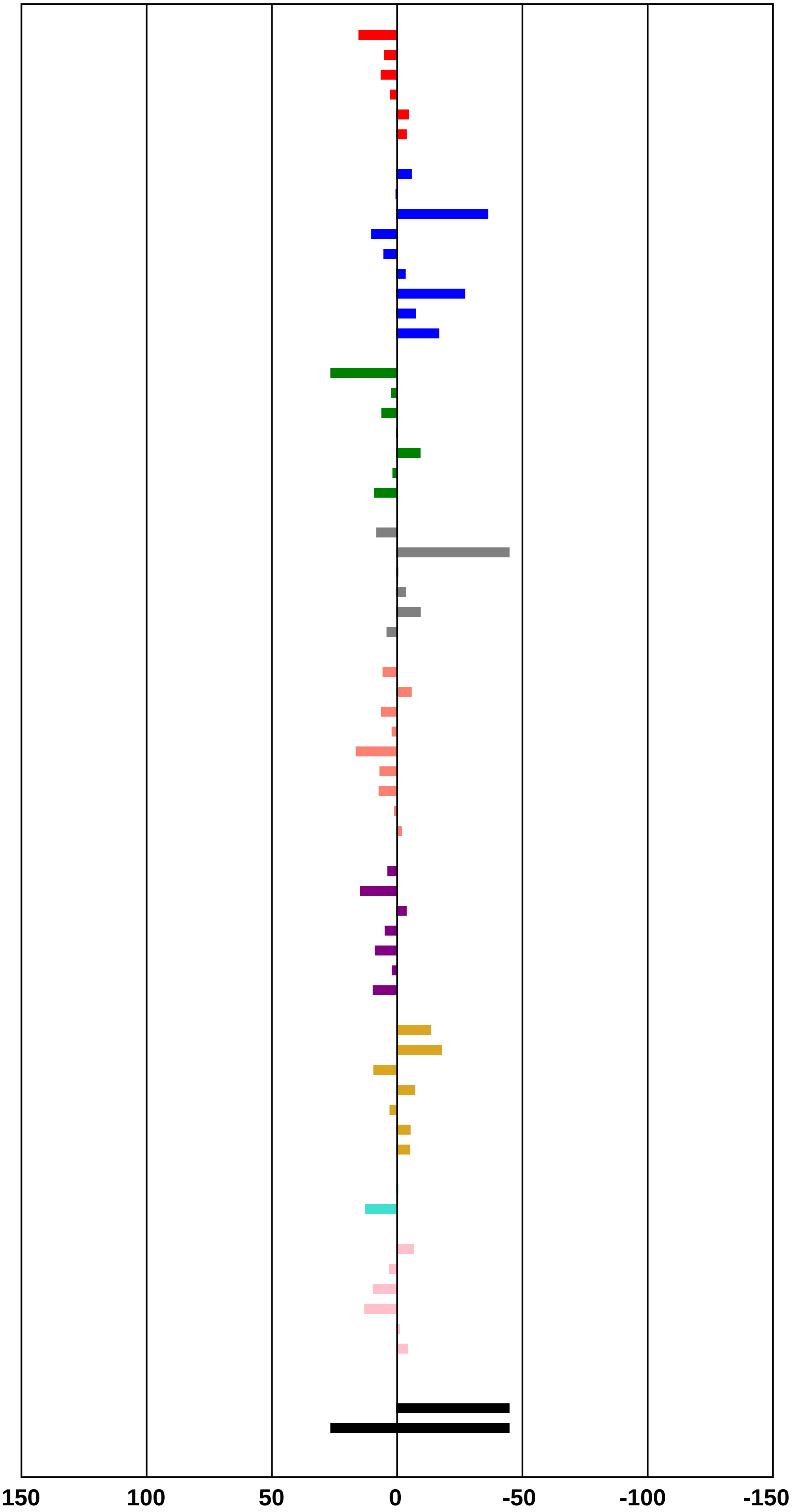

Developmental Therapeutics Program

One Dose Mean Graph

**NSC:** D-811320 / 1	**Conc:** 1.00E-5 Molar	**Test Date:** Dec 17, 2018
**Experiment ID:** 1812OS48		**Report Date:** Jan 14, 2019

Panel/Cell Line	Growth Percent
**Leukemia**	
CCRF-CEM	91.64
HL-60(TB)	99.14
K-562	91.63
MOLT-4	72.23
RPMI-8226	83.30
SR	76.91
**Non-Small Cell Lung Cancer**	
A549/ATCC	95.20
EKVX	68.04
HOP-62	68.31
HOP-92	66.95
NCI-H226	79.81
NCI-H23	81.56
NCI-H322M	83.57
NCI-H460	100.05
NCI-H522	74.40
**Colon Cancer**	
COLO 205	107.53
HCC-2998	98.06
HCT-116	84.83
HCT-15	92.47
HT29	110.99
KM12	90.76
SW-620	96.38
**CNS Cancer**	
SF-268	92.81
SF-295	97.77
SF-539	89.16
SNB-19	95.27
SNB-75	88.17
U251	89.20
**Melanoma**	
LOX IMVI	87.04
MALME-3M	79.89
M14	89.76
MDA-MB-435	103.60
SK-MEL-2	102.56
SK-MEL-28	102.08
SK-MEL-5	94.62
UACC-257	88.36
UACC-62	77.08
**Ovarian Cancer**	
IGROV1	59.60
OVCAR-3	89.18
OVCAR-4	84.59
OVCAR-5	93.80
OVCAR-8	95.47
NCI/ADR-RES	90.57
SK-OV-3	84.45
**Renal Cancer**	
786-0	92.07
A498	73.68
ACHN	84.62
CAKI-1	63.16
SN12C	87.20
TK-10	102.31
UO-31	48.56
**Prostate Cancer**	
PC-3	99.71
DU-145	99.89
**Breast Cancer**	
MCF7	76.04
MDA-MB-231/ATCC	74.78
HS 578T	99.16
BT-549	95.23
T-47D	62.35
MDA-MB-468	79.70
Mean	86.90
Delta	38.34
Range	62.43

Mean Growth Percent - Growth Percent

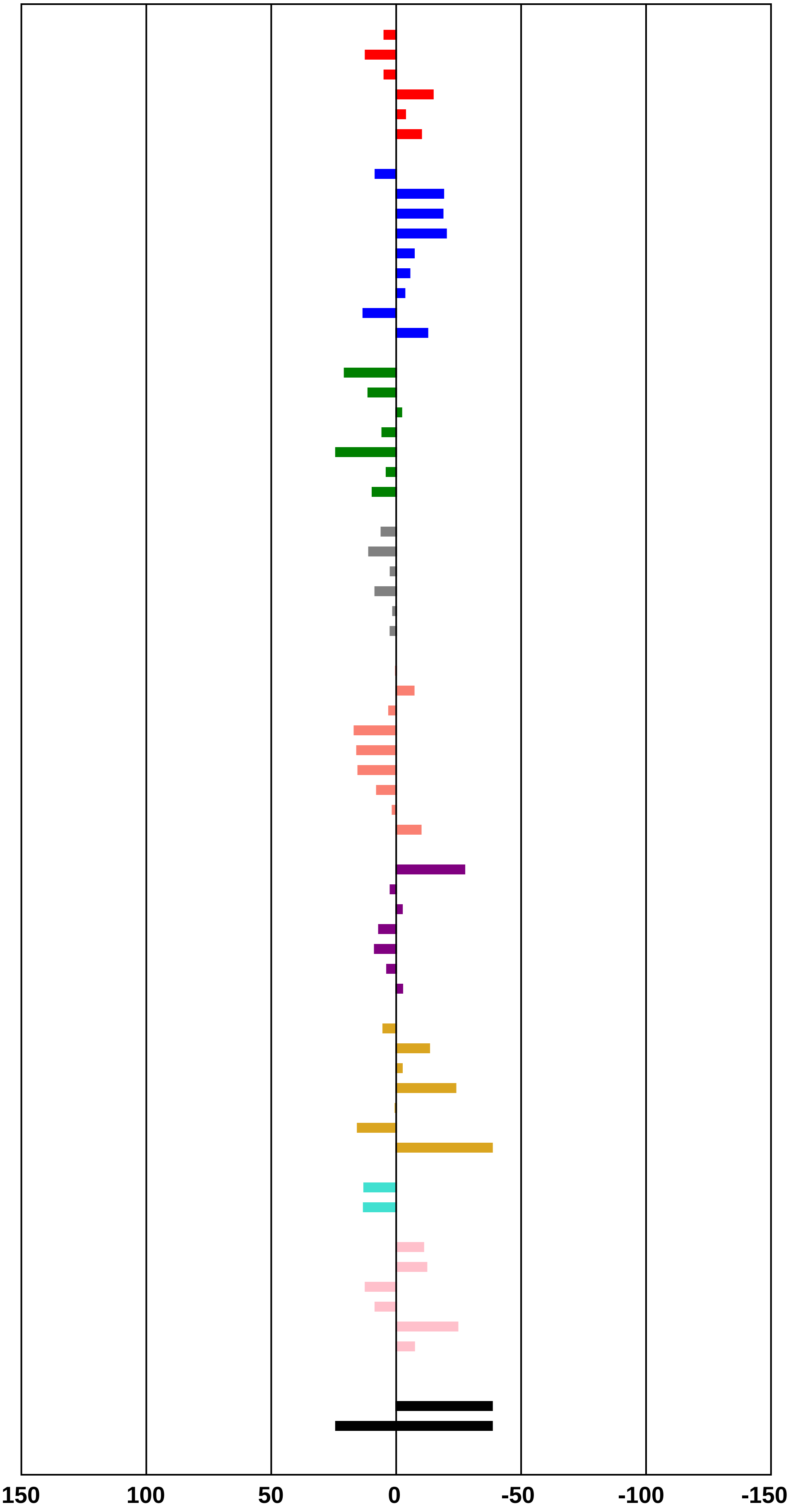

Developmental Therapeutics Program

One Dose Mean Graph

**NSC:** D-811321 / 1	**Conc:** 1.00E-5 Molar	**Test Date:** Dec 17, 2018
**Experiment ID:** 1812OS48		**Report Date:** Jan 14, 2019

Panel/Cell Line	Growth Percent
**Leukemia**	
CCRF-CEM	102.00
HL-60(TB)	103.11
K-562	87.77
MOLT-4	80.06
RPMI-8226	97.84
SR	89.86
**Non-Small Cell Lung Cancer**	
A549/ATCC	89.33
EKVX	64.00
HOP-62	93.29
HOP-92	62.66
NCI-H226	76.36
NCI-H23	81.26
NCI-H322M	73.72
NCI-H460	95.55
NCI-H522	75.71
**Colon Cancer**	
COLO 205	113.36
HCC-2998	106.79
HCT-116	84.70
HCT-15	96.56
HT29	106.60
KM12	88.91
SW-620	95.65
**CNS Cancer**	
SF-268	101.71
SF-295	93.58
SF-539	91.37
SNB-19	102.49
SNB-75	110.78
U251	95.98
**Melanoma**	
LOX IMVI	86.37
MALME-3M	84.18
M14	92.00
MDA-MB-435	102.76
SK-MEL-2	100.41
SK-MEL-28	99.75
SK-MEL-5	88.81
UACC-257	87.12
UACC-62	75.64
**Ovarian Cancer**	
IGROV1	62.23
OVCAR-3	100.07
OVCAR-4	78.67
OVCAR-5	88.61
OVCAR-8	95.36
NCI/ADR-RES	94.73
SK-OV-3	97.07
**Renal Cancer**	
786-0	101.75
A498	67.87
ACHN	88.24
CAKI-1	64.52
SN12C	87.45
TK-10	116.30
UO-31	60.21
**Prostate Cancer**	
PC-3	85.09
DU-145	97.62
**Breast Cancer**	
MCF7	83.49
MDA-MB-231/ATCC	54.95
HS 578T	96.64
BT-549	99.49
T-47D	63.38
MDA-MB-468	84.03
Mean	88.91
Delta	33.96
Range	61.35

Mean Growth Percent - Growth Percent

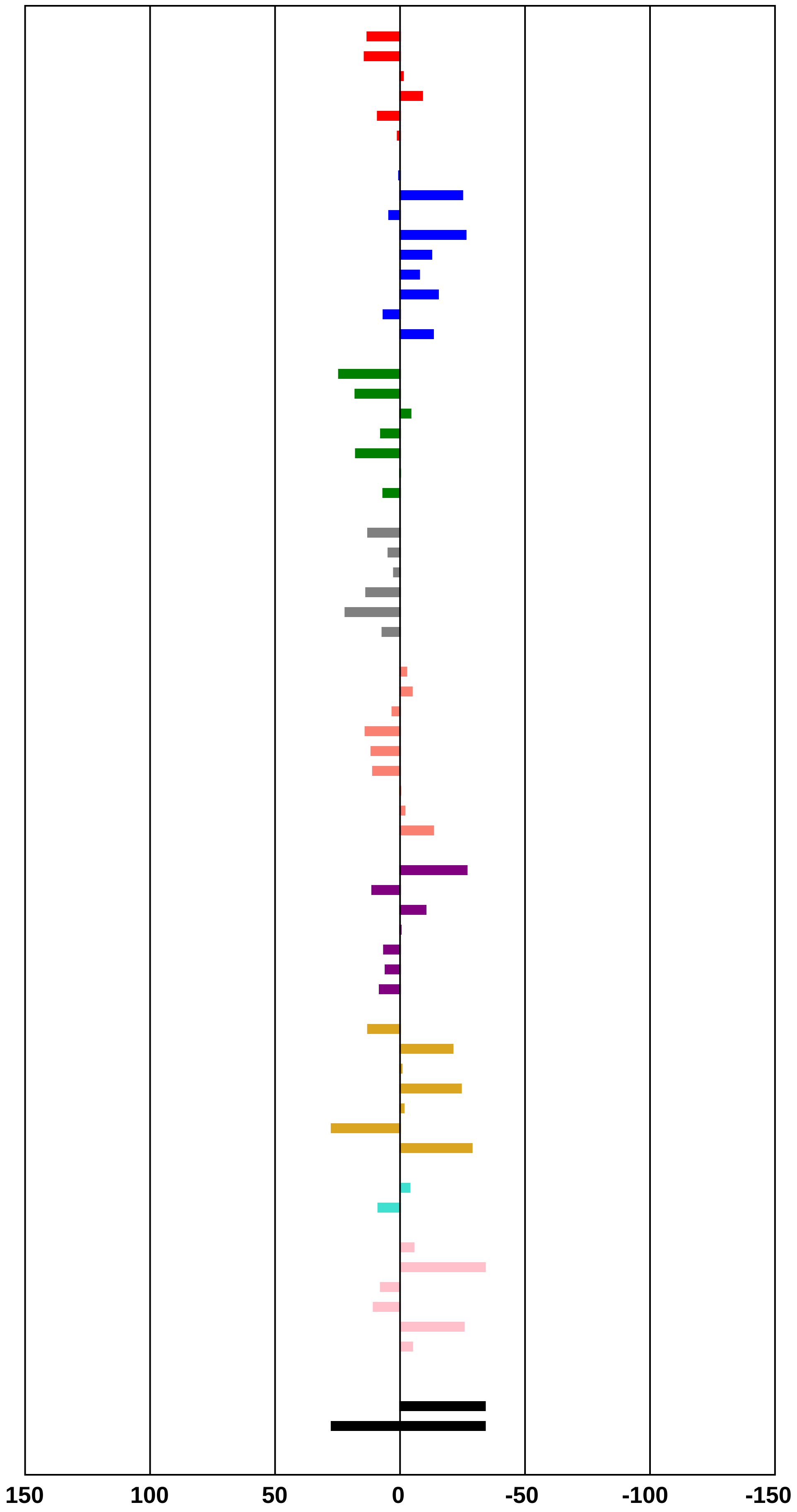

Developmental Therapeutics Program

One Dose Mean Graph

NSC: D-811323 / 1 | **Conc:** 1.00E-5 Molar | **Test Date:** Dec 17, 2018

Experiment ID: 1812OS48 | **Report Date:** Jan 14, 2019

Panel/Cell Line	Growth Percent
**Leukemia**	
CCRF-CEM	83.72
HL-60(TB)	102.87
K-562	80.39
MOLT-4	79.60
RPMI-8226	80.01
SR	89.30
**Non-Small Cell Lung Cancer**	
A549/ATCC	64.38
EKVX	75.86
HOP-62	39.51
HOP-92	80.55
NCI-H226	92.91
NCI-H23	89.09
NCI-H322M	42.51
NCI-H460	63.23
NCI-H522	76.11
**Colon Cancer**	
COLO 205	109.92
HCC-2998	101.69
HCT-116	75.91
HCT-15	92.25
HT29	101.20
KM12	76.45
SW-620	95.36
**CNS Cancer**	
SF-268	99.62
SF-295	42.41
SF-539	90.36
SNB-19	74.28
SNB-75	91.08
U251	76.02
**Melanoma**	
LOX IMVI	95.62
MALME-3M	83.11
M14	94.74
MDA-MB-435	96.14
SK-MEL-2	106.16
SK-MEL-28	98.28
SK-MEL-5	94.15
UACC-257	89.00
UACC-62	81.01
**Ovarian Cancer**	
IGROV1	80.70
OVCAR-3	96.53
OVCAR-4	70.44
OVCAR-5	95.43
OVCAR-8	101.12
NCI/ADR-RES	100.80
SK-OV-3	76.58
**Renal Cancer**	
786-0	93.68
A498	56.82
ACHN	92.30
CAKI-1	71.80
SN12C	90.18
TK-10	111.88
UO-31	71.52
**Prostate Cancer**	
PC-3	74.56
DU-145	92.69
**Breast Cancer**	
MCF7	91.86
MDA-MB-231/ATCC	80.60
HS 578T	100.94
BT-549	96.71
T-47D	39.50
MDA-MB-468	81.09
Mean	84.28
Delta	44.78
Range	72.38

Mean Growth Percent - Growth Percent

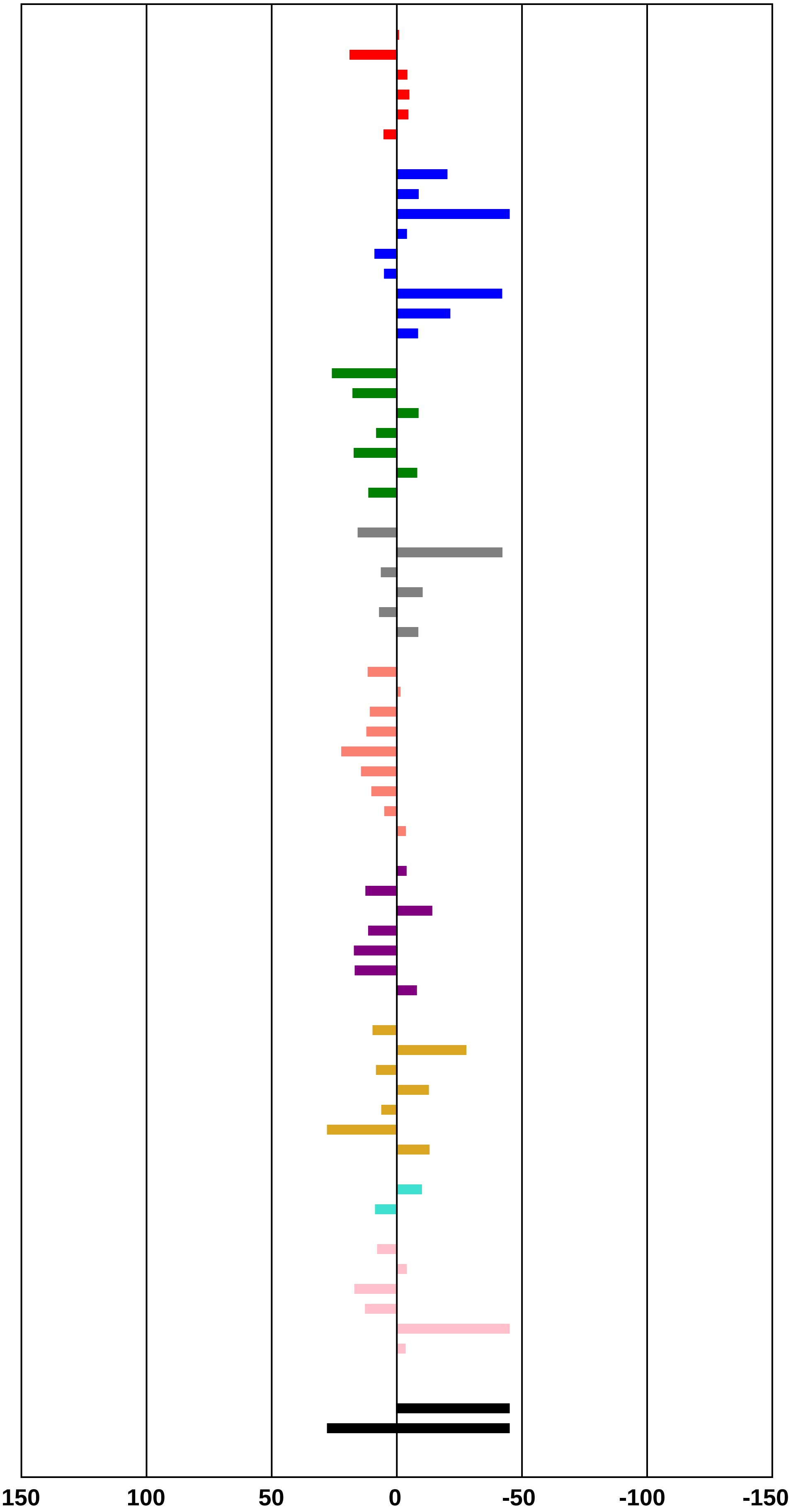

Developmental Therapeutics Program

One Dose Mean Graph

**NSC:** D-811324 / 1	**Conc:** 1.00E-5 Molar
**Test Date:** Dec 17, 2018	**Experiment ID:** 1812OS48
**Report Date:** Jan 14, 2019	

Panel/Cell Line	Growth Percent
Leukemia	
CCRF-CEM	101.14
HL-60(TB)	101.89
K-562	80.07
MOLT-4	76.22
RPMI-8226	86.57
SR	79.63
Non-Small Cell Lung Cancer	
A549/ATCC	87.52
EKVX	70.28
HOP-62	69.85
HOP-92	59.79
NCI-H226	81.39
NCI-H23	87.22
NCI-H322M	69.81
NCI-H460	100.38
NCI-H522	68.60
Colon Cancer	
COLO 205	103.75
HCC-2998	86.51
HCT-116	86.31
HCT-15	86.93
HT29	101.74
KM12	71.80
SW-620	98.77
CNS Cancer	
SF-268	95.71
SF-295	92.99
SF-539	93.74
SNB-19	91.72
SNB-75	83.00
U251	84.45
Melanoma	
LOX IMVI	93.45
MALME-3M	78.61
M14	87.29
MDA-MB-435	104.42
SK-MEL-2	104.80
SK-MEL-28	98.78
SK-MEL-5	89.25
UACC-257	98.59
UACC-62	78.74
Ovarian Cancer	
IGROV1	68.78
OVCAR-3	93.48
OVCAR-4	75.60
OVCAR-5	96.75
OVCAR-8	96.32
NCI/ADR-RES	97.33
SK-OV-3	86.45
Renal Cancer	
786-0	92.99
A498	55.92
ACHN	83.95
CAKI-1	55.67
SN12C	86.21
TK-10	83.51
UO-31	46.17
Prostate Cancer	
PC-3	83.57
DU-145	98.41
Breast Cancer	
MCF7	61.04
MDA-MB-231/ATCC	64.26
HS 578T	89.63
BT-549	87.35
T-47D	6.30
MDA-MB-468	49.80
Mean	82.90
Delta	76.60
Range	98.50

Mean Growth Percent - Growth Percent

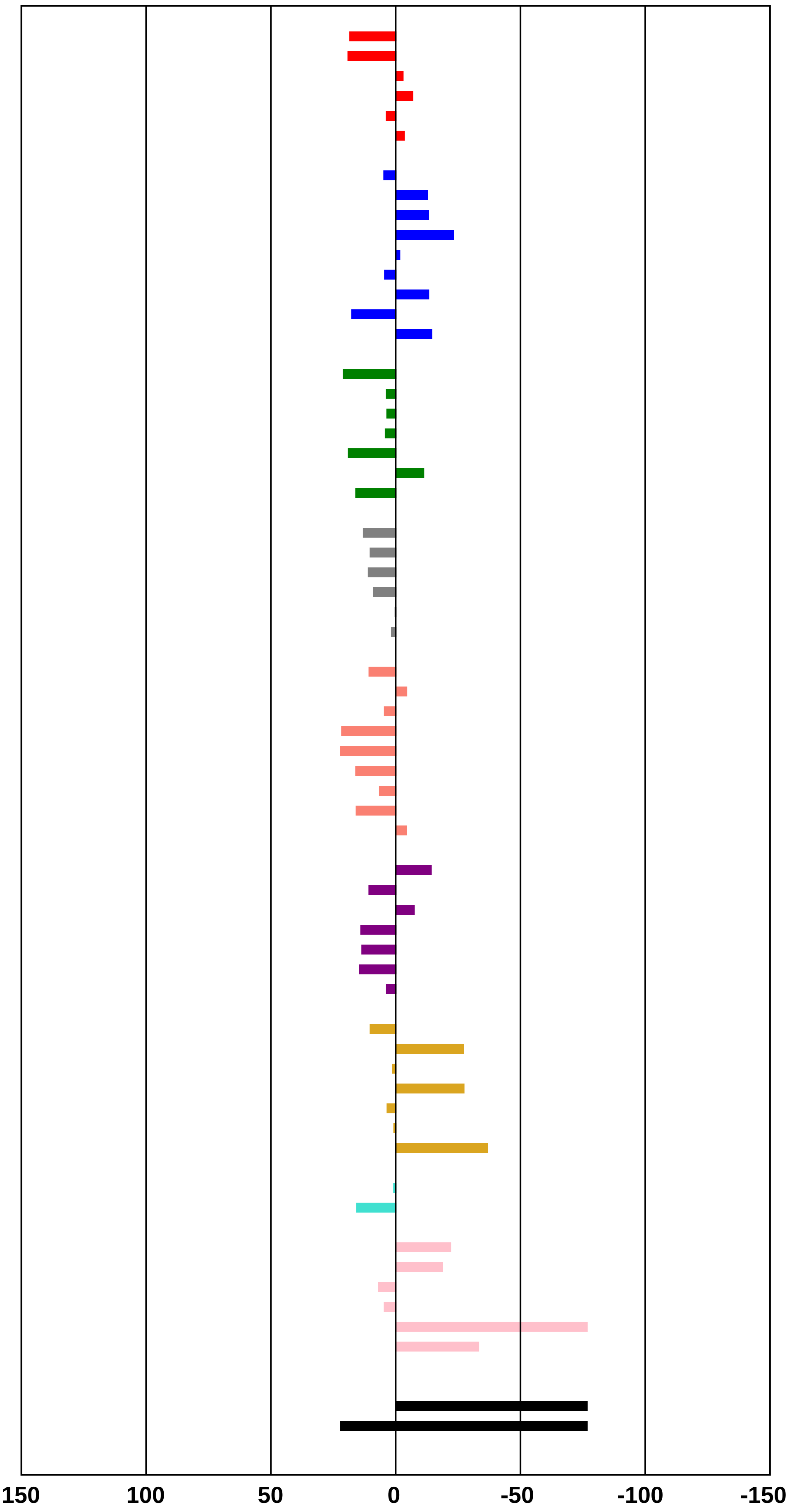

Developmental Therapeutics Program	NSC: D-811325 / 1	Conc: 1.00E-5 Molar	Test Date: Dec 17, 2018
One Dose Mean Graph	Experiment ID: 1812OS48		Report Date: Jan 14, 2019

Panel/Cell Line	Growth Percent
Leukemia	
CCRF-CEM	99.27
HL-60(TB)	96.18
K-562	84.15
MOLT-4	75.75
RPMI-8226	79.70
SR	82.82
Non-Small Cell Lung Cancer	
A549/ATCC	92.13
EKVX	67.65
HOP-62	72.29
HOP-92	63.23
NCI-H226	79.43
NCI-H23	85.87
NCI-H322M	80.00
NCI-H460	94.88
NCI-H522	75.50
Colon Cancer	
COLO 205	111.26
HCC-2998	97.72
HCT-116	92.32
HCT-15	94.82
HT29	103.41
KM12	82.30
SW-620	95.27
CNS Cancer	
SF-268	96.25
SF-295	91.56
SF-539	85.39
SNB-19	93.97
SNB-75	72.71
U251	93.86
Melanoma	
LOX IMVI	88.08
MALME-3M	76.89
M14	90.79
MDA-MB-435	101.57
SK-MEL-2	94.12
SK-MEL-28	102.84
SK-MEL-5	87.41
UACC-257	96.66
UACC-62	73.59
Ovarian Cancer	
IGROV1	66.77
OVCAR-3	90.01
OVCAR-4	73.93
OVCAR-5	104.09
OVCAR-8	97.94
NCI/ADR-RES	95.30
SK-OV-3	87.60
Renal Cancer	
786-0	93.89
A498	54.62
ACHN	83.84
CAKI-1	61.30
SN12C	86.06
TK-10	133.32
UO-31	45.79
Prostate Cancer	
PC-3	84.91
DU-145	96.88
Breast Cancer	
MCF7	64.55
MDA-MB-231/ATCC	59.50
HS 578T	89.53
BT-549	91.42
T-47D	67.17
MDA-MB-468	63.78
Mean	85.49
Delta	39.70
Range	87.53

Mean Growth Percent - Growth Percent

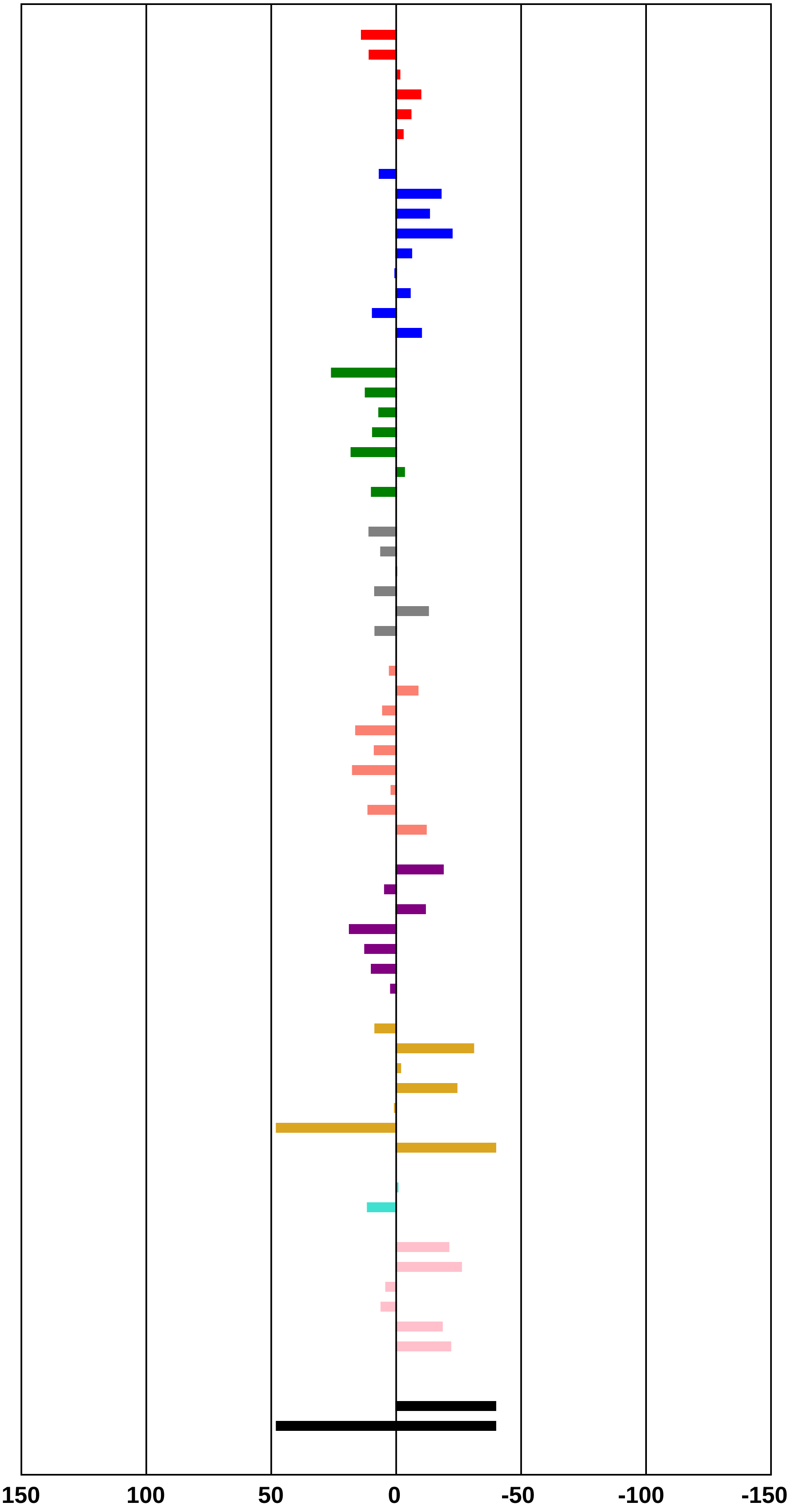

**Developmental Therapeutics Program**	**NSC:** D-811326 / 1	**Conc:** 1.00E-5 Molar	**Test Date:** Dec 17, 2018
**One Dose Mean Graph**	**Experiment ID:** 1812OS48		**Report Date:** Jan 14, 2019

Panel/Cell Line	Growth Percent
Leukemia	
CCRF-CEM	102.72
HL-60(TB)	102.13
K-562	103.91
MOLT-4	105.29
RPMI-8226	91.77
SR	93.65
Non-Small Cell Lung Cancer	
A549/ATCC	103.33
EKVX	83.84
HOP-62	81.66
HOP-92	90.78
NCI-H226	86.59
NCI-H23	93.55
NCI-H322M	87.95
NCI-H460	105.44
NCI-H522	80.12
Colon Cancer	
COLO 205	108.26
HCC-2998	102.09
HCT-116	99.40
HCT-15	94.68
HT29	105.88
KM12	92.06
SW-620	105.37
CNS Cancer	
SF-268	99.46
SF-295	101.34
SF-539	94.76
SNB-19	102.63
SNB-75	83.11
U251	105.46
Melanoma	
LOX IMVI	99.71
MALME-3M	87.55
M14	99.54
MDA-MB-435	109.01
SK-MEL-2	102.06
SK-MEL-28	102.28
SK-MEL-5	99.17
UACC-257	105.52
UACC-62	93.64
Ovarian Cancer	
IGROV1	81.22
OVCAR-3	110.02
OVCAR-4	92.84
OVCAR-5	102.71
OVCAR-8	110.27
NCI/ADR-RES	100.91
SK-OV-3	97.10
Renal Cancer	
786-0	97.67
A498	87.54
ACHN	97.56
CAKI-1	90.67
SN12C	99.58
TK-10	116.66
UO-31	74.81
Prostate Cancer	
PC-3	95.25
DU-145	101.93
Breast Cancer	
MCF7	67.54
MDA-MB-231/ATCC	83.03
HS 578T	101.88
BT-549	101.54
T-47D	58.29
MDA-MB-468	77.84
Mean	95.81
Delta	37.52
Range	58.37

Mean Growth Percent - Growth Percent

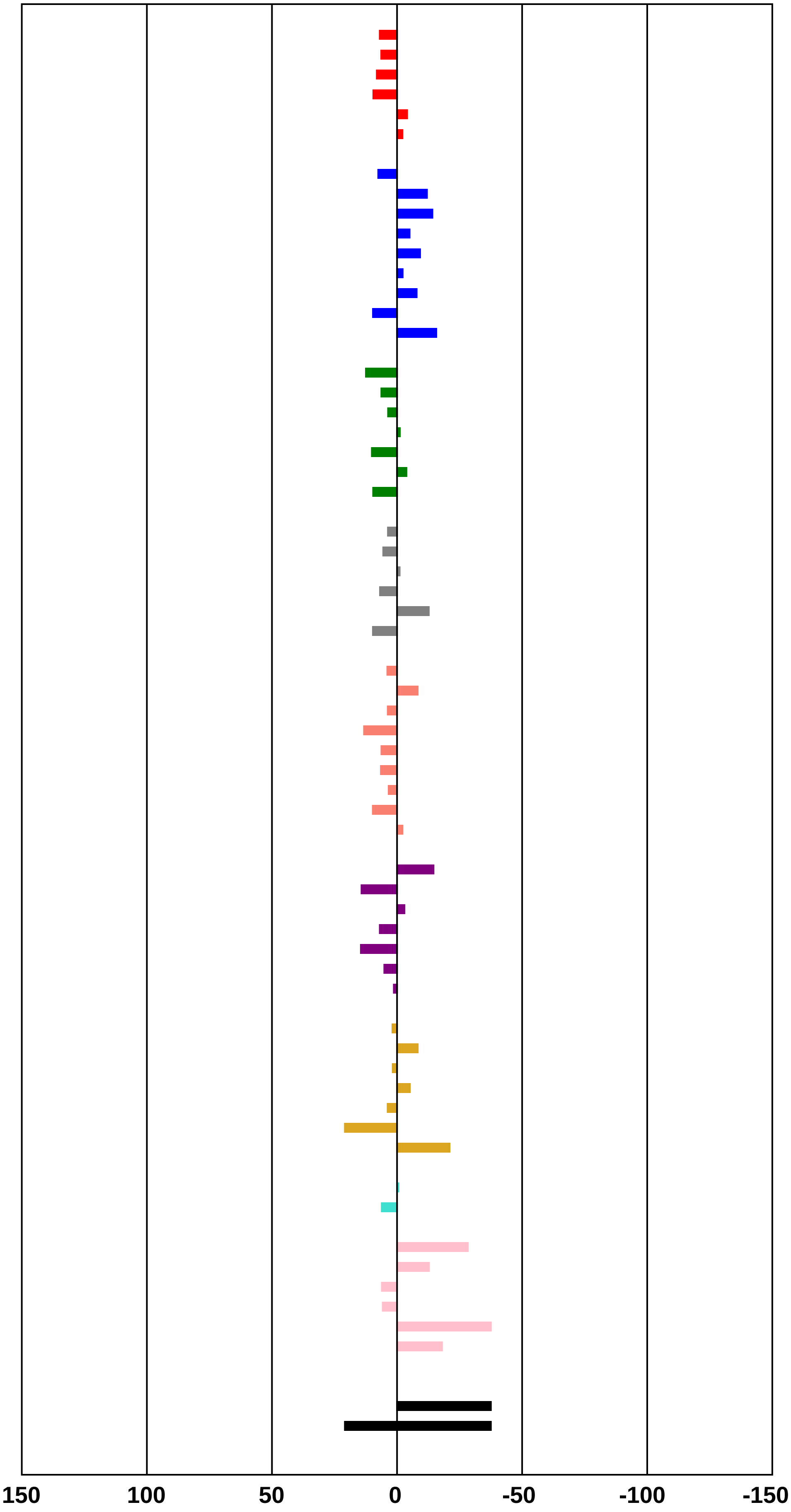

Developmental Therapeutics Program One Dose Mean Graph	NSC: D-811327 / 1	Conc: 1.00E-5 Molar	Test Date: Dec 17, 2018
	Experiment ID: 1812OS48		Report Date: Jan 14, 2019

Panel/Cell Line	Growth Percent
**Leukemia**	
CCRF-CEM	99.35
HL-60(TB)	101.50
K-562	104.27
MOLT-4	86.30
RPMI-8226	90.36
SR	96.16
**Non-Small Cell Lung Cancer**	
A549/ATCC	91.04
EKVX	73.25
HOP-62	77.48
HOP-92	92.12
NCI-H226	81.29
NCI-H23	81.39
NCI-H322M	80.78
NCI-H460	102.09
NCI-H522	88.77
**Colon Cancer**	
COLO 205	110.29
HCC-2998	98.29
HCT-116	96.53
HCT-15	90.30
HT29	109.45
KM12	93.73
SW-620	99.42
**CNS Cancer**	
SF-268	99.92
SF-295	99.33
SF-539	94.12
SNB-19	103.43
SNB-75	86.71
U251	102.20
**Melanoma**	
LOX IMVI	95.20
MALME-3M	88.00
M14	93.20
MDA-MB-435	105.28
SK-MEL-2	106.39
SK-MEL-28	107.70
SK-MEL-5	103.11
UACC-257	91.65
UACC-62	84.95
**Ovarian Cancer**	
IGROV1	65.58
OVCAR-3	99.30
OVCAR-4	93.56
OVCAR-5	96.36
OVCAR-8	97.78
NCI/ADR-RES	96.42
SK-OV-3	91.18
**Renal Cancer**	
786-0	103.46
A498	68.71
ACHN	93.82
CAKI-1	73.72
SN12C	93.07
TK-10	117.73
UO-31	63.51
**Prostate Cancer**	
PC-3	94.34
DU-145	113.22
**Breast Cancer**	
MCF7	74.05
MDA-MB-231/ATCC	77.01
HS 578T	93.35
BT-549	106.34
T-47D	51.08
MDA-MB-468	67.85
Mean	92.15
Delta	41.07
Range	66.65

Mean Growth Percent - Growth Percent

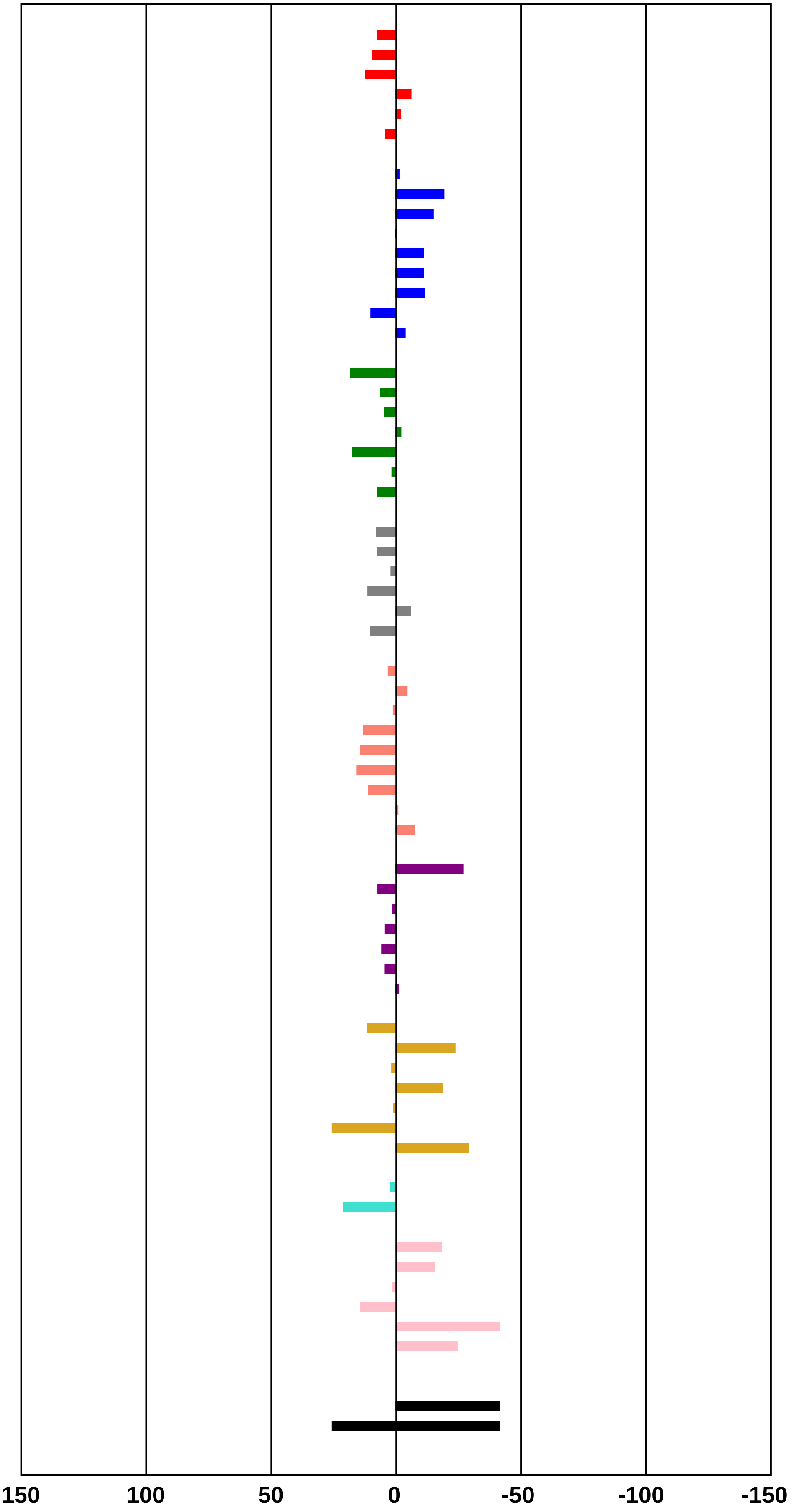

Supplement: Supplementary file 1 [file molecules-26-07695-s001.zip › molecules-1514916-supplementary.pdf]
